# Supplementary material for: Foldamers controlled by functional triamino acids: structural investigation of α/γ-hybrid oligopeptides
Source: Commun Chem. 2024 May 25;7:114. doi: 10.1038/s42004-024-01201-7 (PMC11128005; doi:10.1038/s42004-024-01201-7)
Supplement: Supplementary file 2 — Supplementary information [file 42004_2024_1201_MOESM2_ESM.pdf]

Supplementary Information

Foldamers Controlled by Functional Triamino Acids: Structural Investigation of α/γ-Hybrid Oligopeptides

David Just[1], Vladimír Palivec[1], Kateřina Bártová[1], Lucie Bednárová[1], Markéta Pazderková[1], Ivana Císařová[2], Hector Martinez-Seara[1]*, Ullrich Jahn[1]*

[1] Institute of Organic Chemistry and Biochemistry, Czech Academy of Sciences, Flemingovo náměstí 2, 16610 Prague 6, Czech Republic

[2] Department of Inorganic Chemistry, Faculty of Science, Charles University in Prague, Hlavova 2030/8, 12843 Prague 2, Czech Republic

*Corresponding author e-mails:

ullrich.jahn@uochb.cas.cz

hseara@gmail.com

Table of contents

1. **Summary schemes of the studied foldamers**……S3
2. **Synthesis**……S4
3. **NMR experiments**……S8
   a) **Comparison of selected spectral regions of hexamers 6 and octamers 8**……S8
   b) **Concentration dependence**……S13
   c) **DMSO titration**……S16
   d) **Temperature dependence**……S19
   e) **AcOH titration**……S22
   f) **HCl addition**……S25
   g) **Comparison of spectral regions of peptides 6R^{H}AG-Ac and 6SDabG-Ac at different pH**……S27
   h) **2D ROESY experiments**……S29
4. **ECD and IR/VCD spectra**……S37
5. **Molecular dynamics and calculations**……S44
6. **X-Ray crystallographic data**……S51
7. **General experimental information**……S58
8. **General procedures**……S58
9. **Experimental data and characterisation**……S60
10. **Supplementary References**……S99

Supplementary Methods

1) Summary schemes of the studied foldamers

Fmoc-6GR^{P}A
Ac-6GR^{P}A

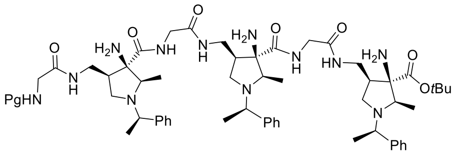

Ac-6GR^{H}A

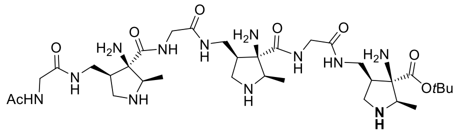

Fmoc-6AR^{P}A
Ac-6AR^{P}A

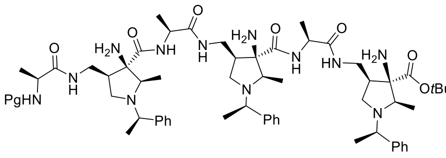

Ac-6AR^{H}A

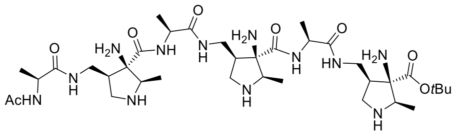

Fmoc-6AS^{P}A
Ac-6AS^{P}A

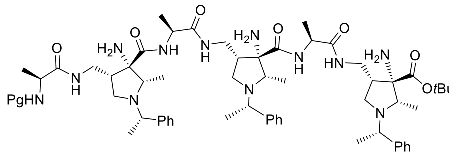

Ac-6AS^{H}A

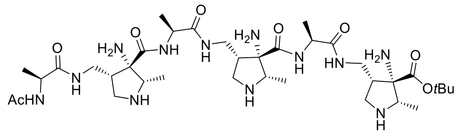

Ac-6GSDab

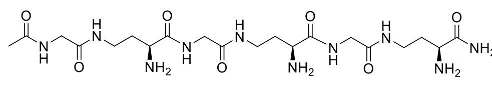

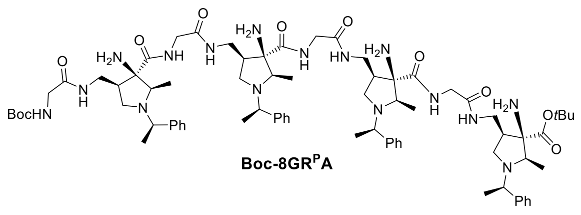

Boc-8GR^{P}A

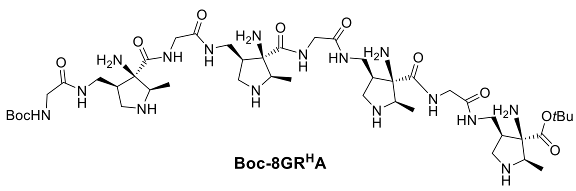

Boc-8GR^{H}A

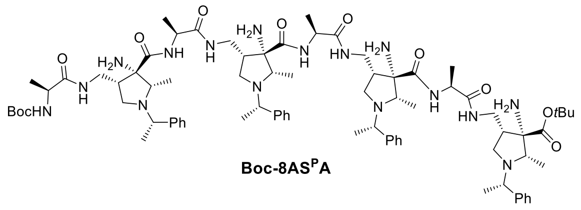

Boc-8AS^{P}A

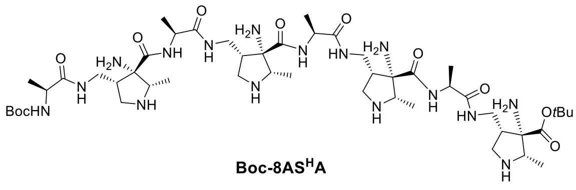

Boc-8AS^{H}A

2) Synthesis

a) GR^{P}A series

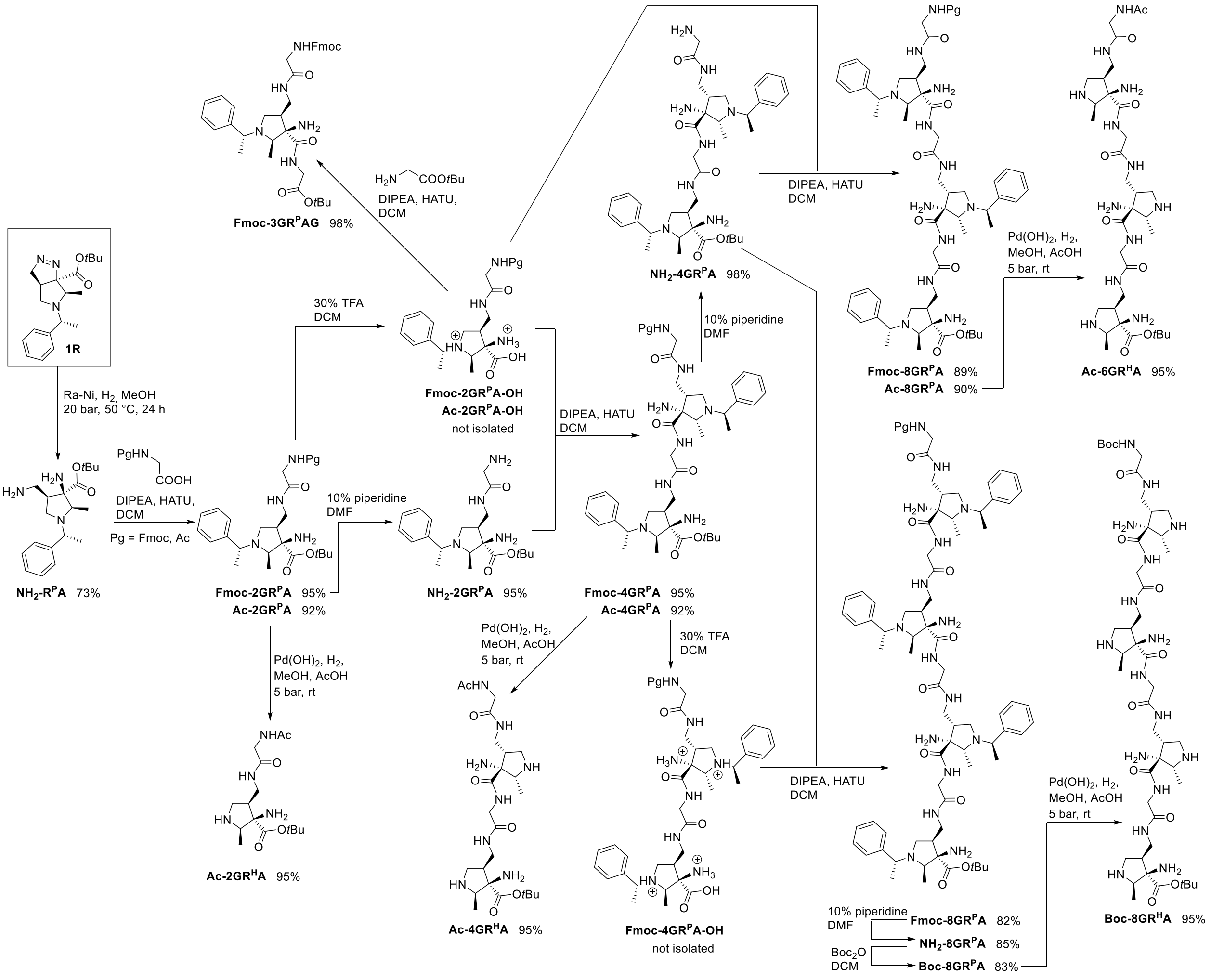

Figure S1. Synthetic sequence of the **GR^{P}A** foldamer series.

The monomeric building block **NH$_2$-R^{P}A** was prepared by Ra-Ni mediated hydrogenative reduction of pyrazolinopyrrolidine **1R** that was prepared according to the literature.[1] Peptide coupling of **NH$_2$-R^{P}A** with Fmoc-protected glycine provided dimer **Fmoc-2GR^{P}A**, which served as building blocks for oligopeptide assembly by a convergent *tert*-butyl ester-Fmoc amine strategy. Deprotection of the Fmoc group by piperidine/DMF in one part of **Fmoc-2GR^{P}A** furnished free amino dipeptides **NH$_2$-2GR^{P}A**, whereas liberation of the *tert*-butyl esters unit by TFA/DCM in the other part of **Fmoc-2GR^{P}A** provided carboxylic acid building blocks **Fmoc-2GR^{P}A-OH**. Tetramer **Fmoc-4GR^{P}A** was obtained in excellent yields by coupling of **NH$_2$-2GR^{P}A** and **Fmoc-2GR^{P}A-OH** using HATU as activator. Similar splitting of **Fmoc-4GR^{P}A** and individual deprotection as in **Fmoc-2GR^{P}A** set the stage for further oligomer assembly. Hexamer **Fmoc-6GR^{P}A** was obtained in very good yield by HATU-mediated coupling from **NH$_2$-4GR^{P}A** and **Fmoc-2GR^{P}A-OH**, whereas octamer **Fmoc-8GR^{P}A** was prepared by

coupling of **NH$_2$-4GR^{P}A** and **Fmoc-4GR^{P}A-OH**. However, the Fmoc group is not compatible with free pyrrolidine units in the oligomers on hydrogenolytic removal of the phenylethyl groups. Therefore, the dimer, tetramer, and hexamer were prepared as *N*-acetyl-protected derivatives **Ac-2GR^{P}A**, **Ac-4GR^{P}A,** and **Ac-6GR^{P}A** using an identical strategy with Ac-protected glycine, whereas *N*-Boc protection of **NH$_2$-8GR^{P}A** proved to be better for octamer **Boc-8GR^{P}A**. Finally, hydrogenolysis of the phenylethyl groups catalyzed by Pearlman's catalyst furnished dimer **Ac-2GR^{H}A**, tetramer **Ac-4GR^{H}A**, and hexamer **Ac-6GR^{H}A**, as well as octamer **Boc-8GR^{H}A** in quantitative yields.

b) AS^{P}A series

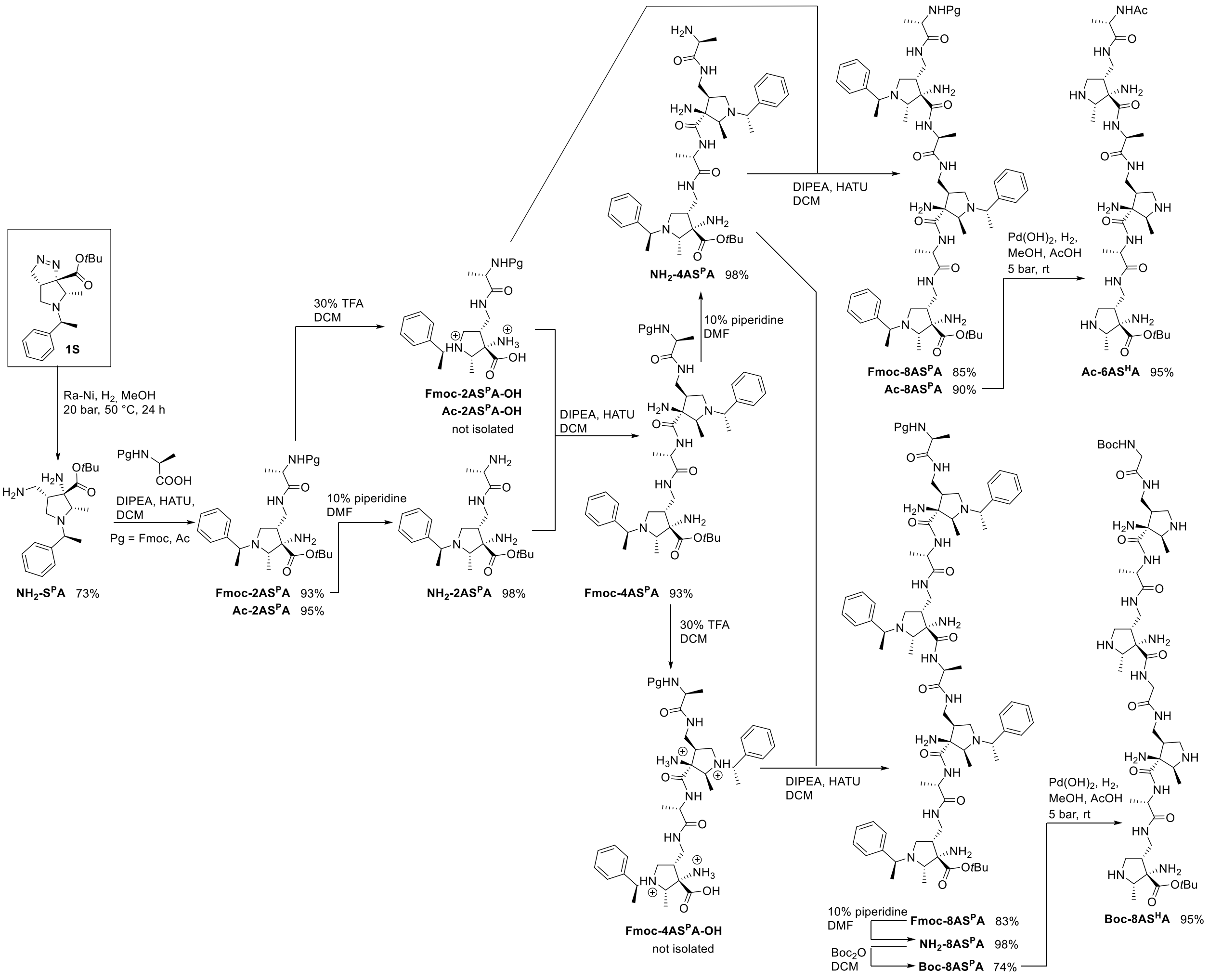

Figure S2. Synthetic sequence of the **AS^{P}A** foldamer series.

Similarly, the monomeric building block **NH$_2$-S^{P}A** was prepared through Ra-Ni mediated hydrogenative reduction of pyrazolinopyrrolidine **1S**, prepared as described in the literature.[1] Peptide coupling of **NH$_2$-S^{P}A** with Fmoc-protected L-alanine yielded dimer **Fmoc-2AS^{P}A**, serving as the foundation for oligopeptide assembly via the same convergent *tert*-butyl ester-Fmoc amine

approach. Removal of the Fmoc group by piperidine/DMF from one part of **Fmoc-2AS^{P}A** provided free amino dipeptides **NH₂-2AS^{P}A**, while liberation of the *tert*-butyl esters unit by TFA/DCM from the other part of **Fmoc-2AS^{P}A** generated carboxylic acid building blocks **Fmoc-2AS^{P}A-OH**. Tetramer **Fmoc-4AS^{P}A** was furnished in excellent yields by coupling of **NH₂-2AS^{P}A** and **Fmoc-2AS^{P}A-OH** using HATU. A similar process splitting of **Fmoc-4AS^{P}A** and individual deprotection as seen in **Fmoc-2AS^{P}A** paved the way for further oligomer assembly. Hexamer **Fmoc-6AS^{P}A** was obtained in very good yield by peptide coupling of **NH₂-4AS^{P}A** and **Fmoc-2AS^{P}A-OH**, while octamer **Fmoc-8AS^{P}A** was prepared via peptide coupling of **NH₂-4AS^{P}A** and **Fmoc-4AS^{P}A-OH**. Again, due to the incompatibility of the Fmoc group with free pyrrolidine units, the hexamer was prepared as *N*-acetyl-protected derivative **Ac-6AS^{P}A** using an identical strategy with *N*-acetyl-protected L-alanine, whereas *N*-Boc protection of **NH₂-8AS^{P}A** proved to be better for octamer **Boc-8AS^{P}A**. Finally, hydrogenolysis of the phenylethyl groups catalyzed by Pearlman's catalyst provided hexamer **Ac-6AS^{H}A**, as well as octamer **Boc-8AS^{H}A** in quantitative yields.

c) AR^{P}A series

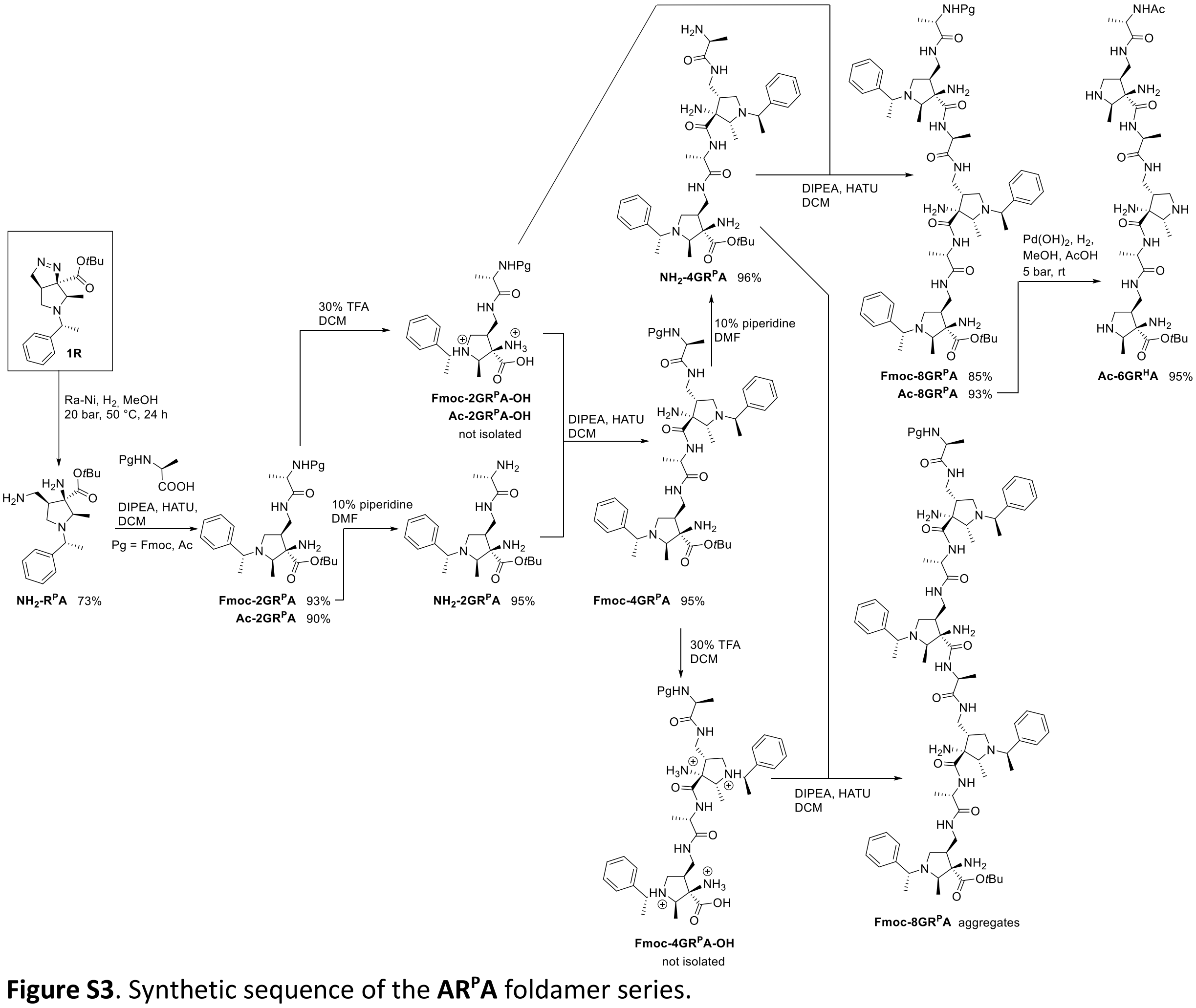

Figure S3. Synthetic sequence of the **AR^{P}A** foldamer series.

The synthetic sequence of the **AR^{P}A** foldamer series followed the identical strategy as used for the **GR^{P}A** foldamer series using Fmoc-protected L-alanine or *N*-acetyl-protected L-alanine. Hexamer **Fmoc-6AR^{P}A** and octamer **Fmoc-8AR^{P}A** were obtained in very good yields. Notably, octamer **Fmoc-8AR^{P}A** irreversibly formed insoluble aggregates on evaporation, which disabled further investigations. Hydrogenolysis of the phenylethyl groups catalyzed by Pearlman's catalyst provided hexamer **Ac-6AR^{H}A**.

3) NMR experiments

a) Comparison of selected spectral regions of hexamers 6 and octamers 8

Peptides **Fmoc-6^{P}** and **Boc-8GR^{P}A**, **Boc-8AS^{P}A** were compared for their amide resonances in $CDCl_3$ (Figure S4). Hexamers **Fmoc-6^{P}** and octamers **Boc-8GR^{P}A**, **Boc-8AS^{P}A** displayed amide N3-H, N5-H, and N7-H resonances in the range between 7.8-8.3 ppm indicating strong H-bonding. All even amide N-H resonances were found in the 7.0-7.8 ppm region indicating weakly hydrogen bonded amides. The *N*-terminal carbamate N-H resonance is located upfield at ca. 6.5 ppm for **Fmoc-6GR^{P}A** and **Fmoc-6AS^{P}A** suggesting only weak participation in hydrogen bonding, which strengthens as the peptide length increases as documented by the downfield shift of the N-terminal N-H signal from 5.5 ppm for dimers **Fmoc-2GR^{P}A** and **Fmoc-2AS^{P}A** via 5.9 ppm and 6.6 ppm for **Fmoc-4GR^{P}A** and **Fmoc-4AS^{P}A**, respectively. In contrast, in the **Fmoc-AR^{P}A**-series *N*-terminal N-H resonances are located at ca. 5.7 ppm for all peptide lengths, indicating no interactions with other parts of the molecules. The N2-H resonance of **Fmoc-6AR^{P}A** was not detectable at r.t. (*cf*. Figure S19).

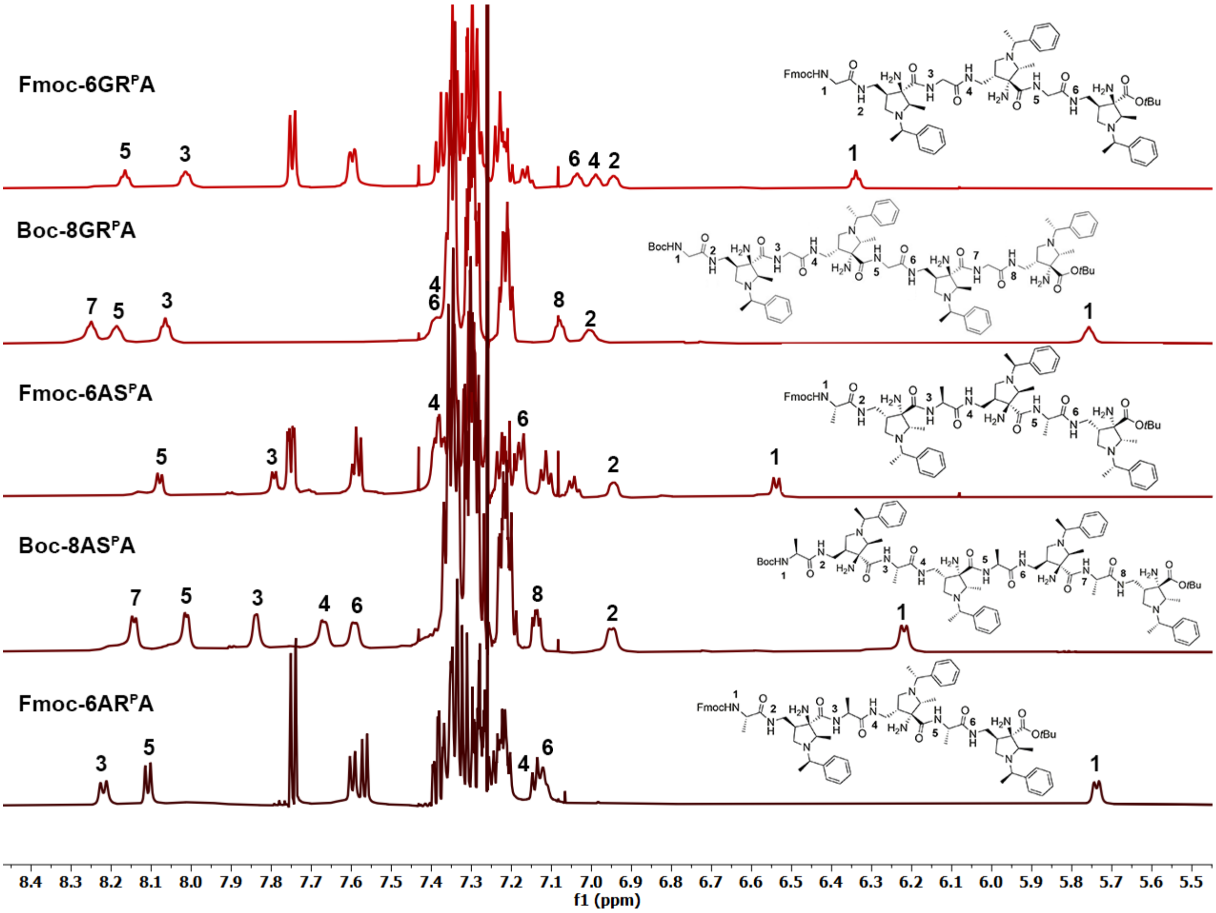

Figure S4. Comparison of hexamers **Fmoc-6^{P}** and octamers **Boc-8GR^{P}A**, **Boc-8AS^{P}A** in $CDCl_3$ in the amide proton region from 5.5 to 8.5 ppm.

Peptides **Fmoc-6^P** and **Boc-8GR^{P}A**, **Boc-8AS^{P}A** were compared for their CHα resonances at α-amino acid residues in $CDCl_3$ (Figure S5). For **Boc-8GR^{P}A**, the α-protons of peripheral glycine α-residues Gly(1) and Gly(7) have chemical shifts ranging from 3.6-3.8 ppm and 4.0 ppm, similar to peripheral α-residues Gly(1) and Gly(5) of hexamer **Fmoc-6GR^{P}A** with ranges from 3.6-3.8 ppm and 3.9-4.0 ppm. The α-protons of the internal glycine units Gly(3) and Gly(5) for octamer **Boc-8GR^{P}A** are in contrast overlapped in narrow ranges of 3.50-3.55 and 4.03-4.08 ppm, hinting to a similar chemical and magnetic environment. The chemical shifts are comparable to internal glycine unit Gly(3) of hexamer **Fmoc-6GR^{P}A** signals of which were found at 3.40 and 4.08 ppm.

Similarly, for the alanine-containing octamer **Boc-8AS^{P}A** the α-protons of peripheral alanine α-residues Ala(1) and Ala(7) have chemical shifts at 4.16 and 4.26 ppm, similar to those of peripheral α-residues Ala(1) and Ala(5) of hexamer **Fmoc-6AS^{P}A** at 4.20 and 4.35 ppm. The α-protons of the internal alanine units Ala(3) and Ala(5) for octamer **Boc-8AS^{P}A** are overlapped at 4.34 ppm comparable to the internal alanine unit Ala(3) of hexamer **Fmoc-6AS^{P}A** signal at 4.28 ppm. Resonances of hexamer **Fmoc-6AR^{P}A** were identified at 4.40 and 4.23 ppm for terminal alanine α-residues Ala(1) and Ala(5), while the internal alanine unit Ala(3) displayed a signal at 4.12 ppm.

This indicates similarity of the solution structures of hexamers **Fmoc-6GR^{P}A, Fmoc-6AS^{P}A** and octamers **Boc-8GR^{P}A, Boc-8AS^{P}A**, whereas hexamer **Fmoc-6AR^{P}A** displayed different behavior. Unfortunately, octamer **Fmoc-8AR^{P}A** aggregated and irreversibly precipitated after formation, which prevented gaining structural information in solution.

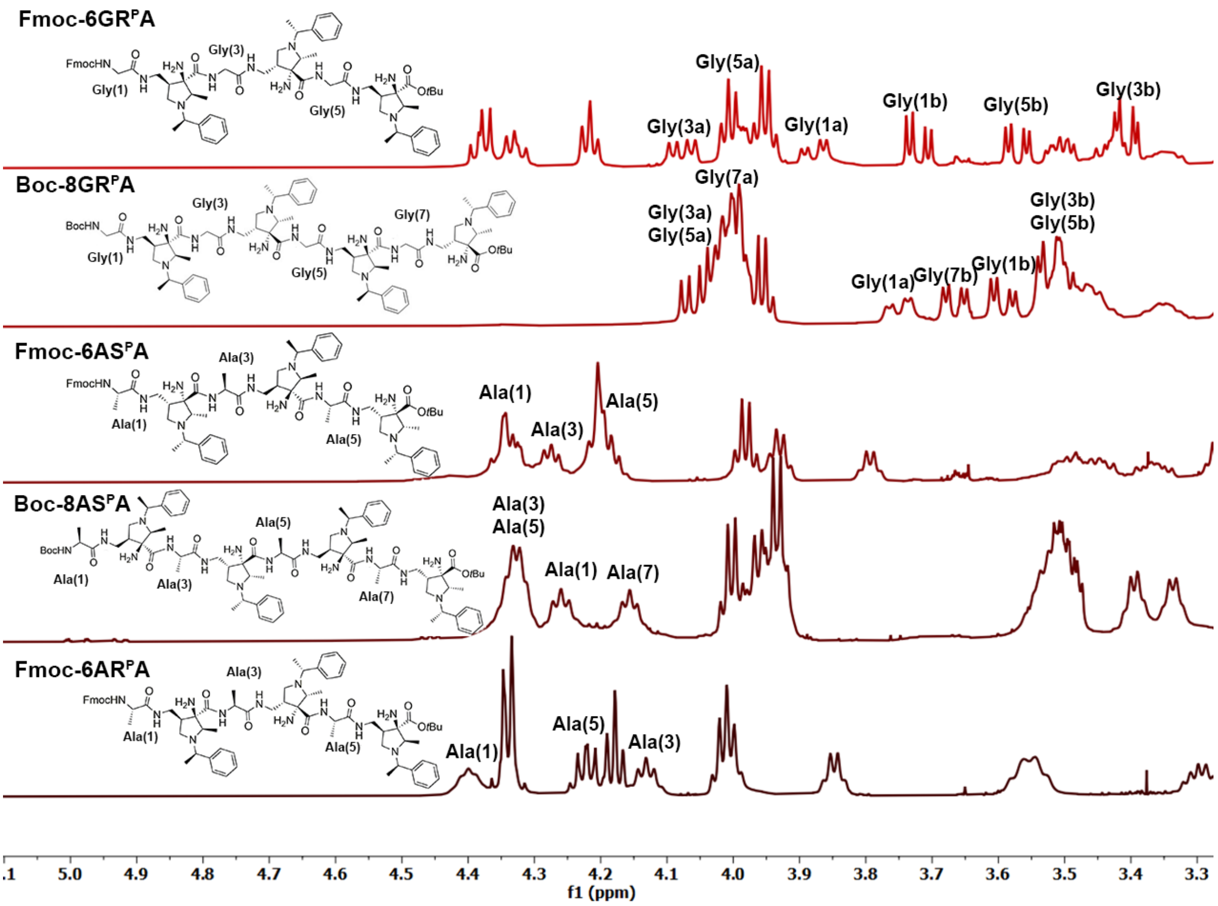

Figure S5. Comparison of hexamers **Fmoc-6^P** and octamers **Boc-8GR^PA**, **Boc-8AS^PA** in $CDCl_3$ in the CHα region of α-amino acid residues in the range from 3.3 to 5.1 ppm.

Peptides **Ac-6^{H}** and **Boc-8GR^{H}A**, **Boc-8AS^{H}A**, and **Ac-6GSDab** were compared for their amide resonances (Figure S6). Because of their increased basicity compared to **Fmoc-6^{P}** and **Ac-6^{P}**, the amide N-H signals were not detectable; hence, the pH had to be adjusted to approximately 4.5 by addition of CD_3COOD. All six amide N-H protons of **Ac-6^{H}** and all eight N-H protons of **Boc-8GR^{H}A** and **Boc-8AS^{H}A** display chemical shifts $\delta > 8$ ppm. Hexamer **Ac-6GR^{H}A** showed amide proton resonances of N3-H and N5-H at 8.7 ppm, whereas the other amide protons were found in the range of 8.1-8.3 ppm, suggesting interactions with the adjacent NH_2 groups. Similarly, octamer **Boc-8GR^{H}A** showed the same distribution of all amides except for the N-terminal N1-H resonance, which is attached to a chemically different protecting group. The spectral similarities of both hexamer **Ac-6GR^{H}A** and octamer **Boc-8GR^{H}A** imply their similar spatial structural arrangement. Hexamer **Ac-6AS^{H}A** displayed amide proton resonances N3-H and N5-H at 8.5 ppm, which are less distinct from the other amide protons founded in the range of 8.2-8.4 ppm. Correspondingly, octamer **Boc-8AS^{H}A** showed a very similar distribution as hexamer **Ac-6AS^{H}A**, thus similar spatial structural arrangements can be expected. Hexamer **Ac-6AR^{H}A** displayed even less distinction having all amide N-H resonances in the range of 8.2-8.4 ppm. Surprisingly, hexamer **Ac-6GSDab** showed amide resonances N3-H and N5-H at 8.9 ppm, whereas the other amide protons were found in the range of 8.2-8.4 ppm, suggesting that the 5-membered NH(*i*)···NH_2(*i*–1) interaction likely also exists in this truncated peptide despite the protic conditions.

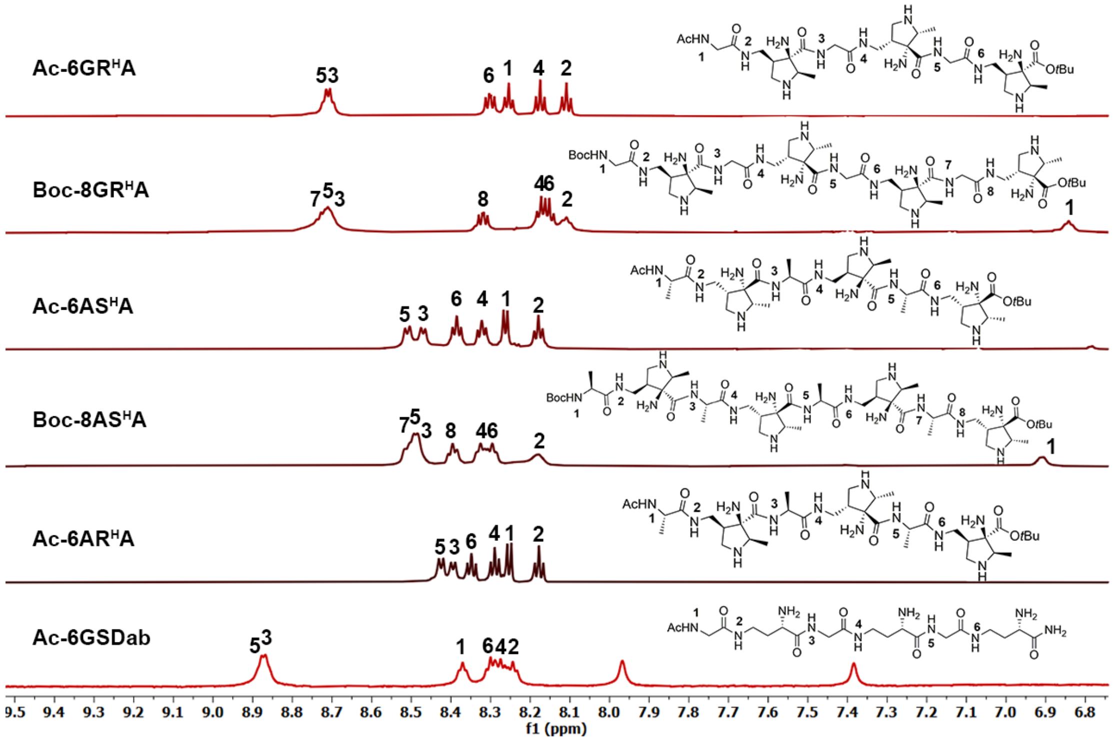

Figure S6. Comparison of hexamers **Ac-6^{H}** and **Ac-6GSDab**, and octamers **Boc-8GR^{H}A**, **Boc-8AS^{H}A** in D_2O/H_2O 9:1 acidified with CD_3COOD to pH of 4.5 in the amide proton region in the range from 6.8 to 9.5 ppm.

Peptides **Ac-6^H** were compared for their CHα resonances at the α-amino acid positions under acidic aqueous conditions (Figure S7). For **Ac-6GR^HA**, the α-protons of glycine α-residues Gly(3) and Gly(5) have chemical shifts of their AB systems in the range from 3.85 ppm to 3.9-4.0 ppm, the N-terminal Gly(1) was found at 3.8 ppm as a single doublet. Although they are much less distinct than those of glycine-containing hexamer **Fmoc-6GR^PA** the different location of the AB system protons still hints to a defined spatial arrangement, which likely gains certain flexibility at the N-terminus. For hexamer **Ac-6AS^HA**, the α-protons of alanine α-residues displayed a narrower resonance distribution ranging from 4.2-4.4 ppm, whereas hexamer **Ac-6AR^HA** exhibits resonances between the same alanine residues in an even narrower range at 4.2 ppm. This effect can be a consequence of a reduced tendency to form an ordered secondary structure; alternatively, the peptide might feature a different secondary structure arrangement. In contrast, the octamers **Boc-8GR^HA**, and **Boc-8AS^HA** provided ^{1}H NMR spectra, which are complex and do not allow further structural characterization other than demonstrating their identity.

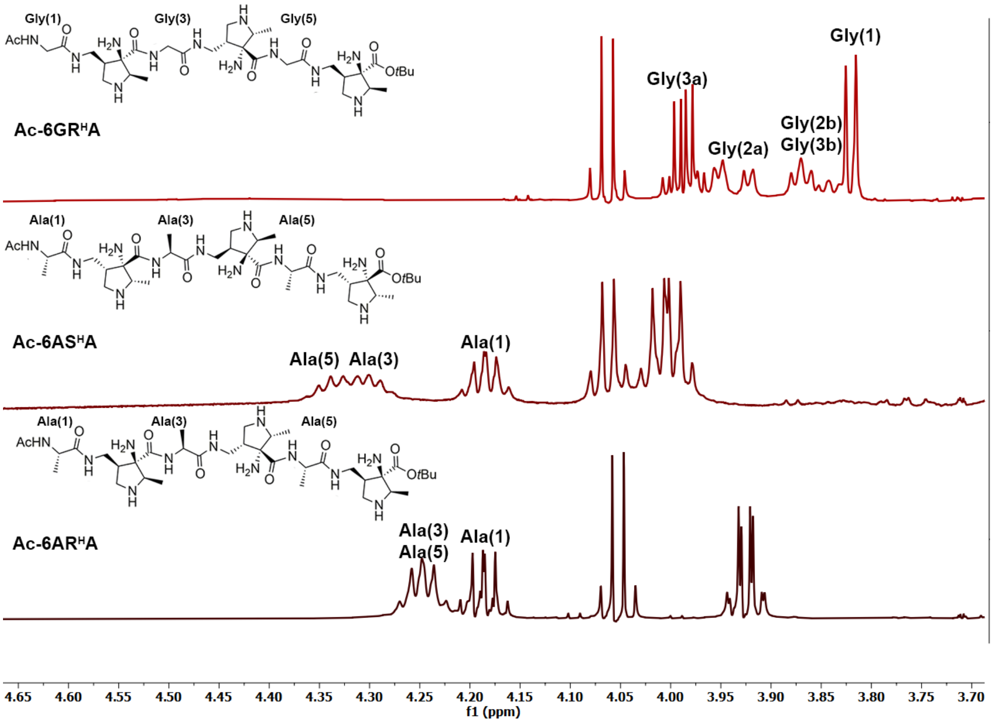

Figure S7. Comparison of hexamers **Ac-6^H** in D_2O/H_2O 9:1 acidified with CD_3COOD to pH of 4.5 in the CHα region of α-amino acid residues in the range from 3.7 to 4.6 ppm.

b) Concentration dependence

Peptides **Fmoc-6^P^** and **Boc-8GR^P^A** were dissolved in $CDCl_3$ (500 µL, 0.03% TMS) at concentrations of 0.2 mM, 1 mM, 5 mM and 25 mM for **Fmoc-6^P^**, 0.1 mM, 0.6 mM, 3 mM, 15 mM and 70 mM for **Boc-8GR^P^A**, respectively, and the NMR spectra were recorded at 24 °C (Figures S8-S11).

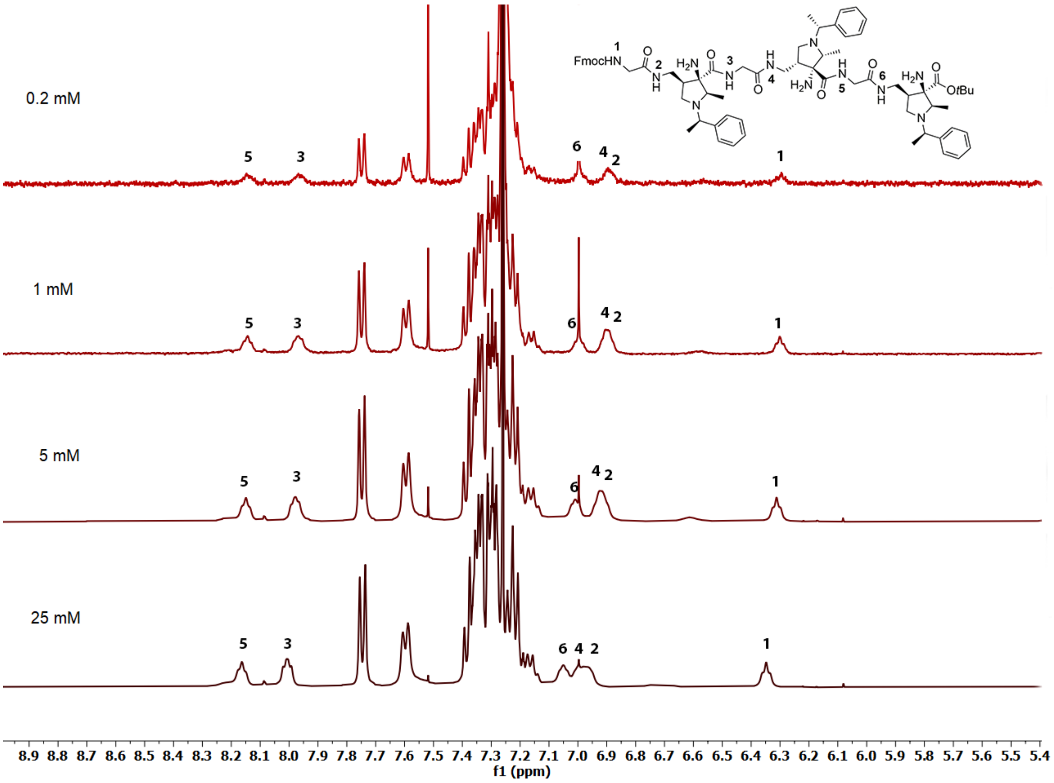

Figure S8. Concentration dependence of Fmoc(Gly-(*R*,*R*,*R*,*R*)^{P}AAMP)$_3$O*t*Bu (**Fmoc-6GR^P^A**) in $CDCl_3$.

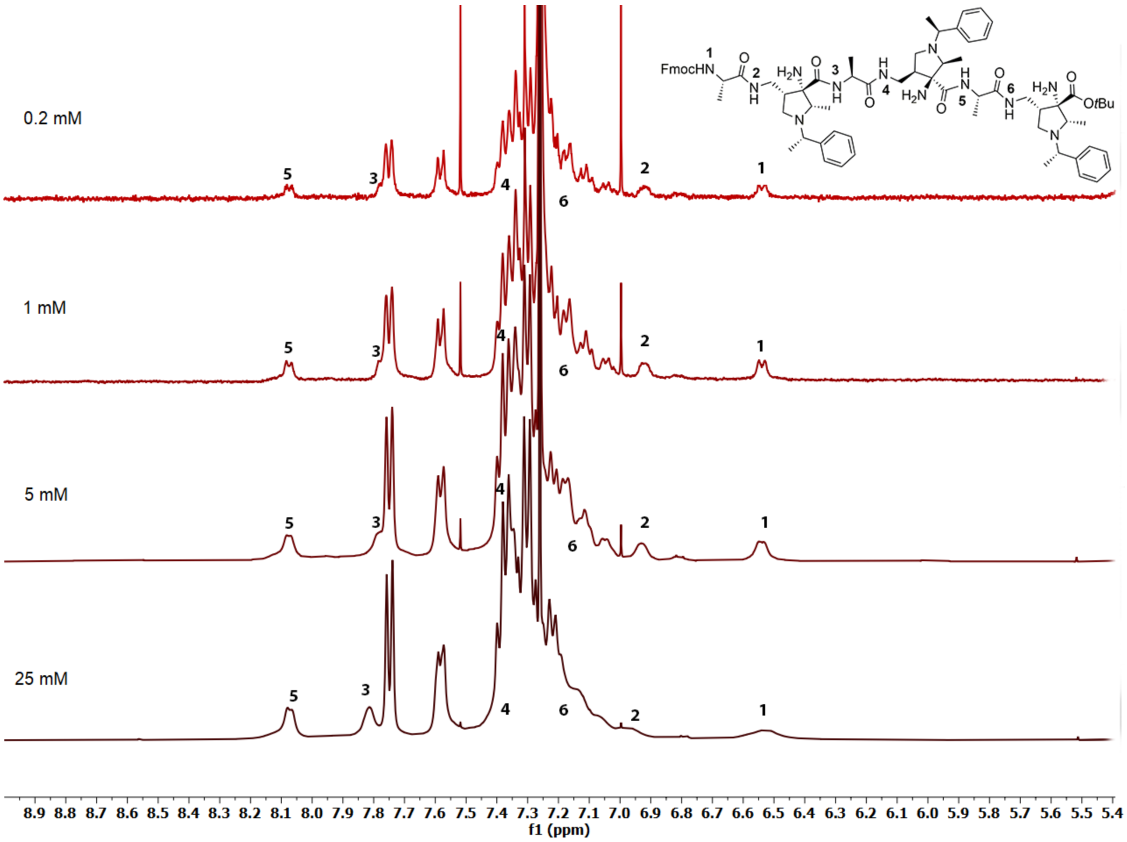

Figure S9. Concentration dependence of Fmoc(Ala-(*S*,*S*,*S*,*S*)[P]AAMP)$_3$O*t*Bu (**Fmoc-6AS[P]A**) in $CDCl_3$.

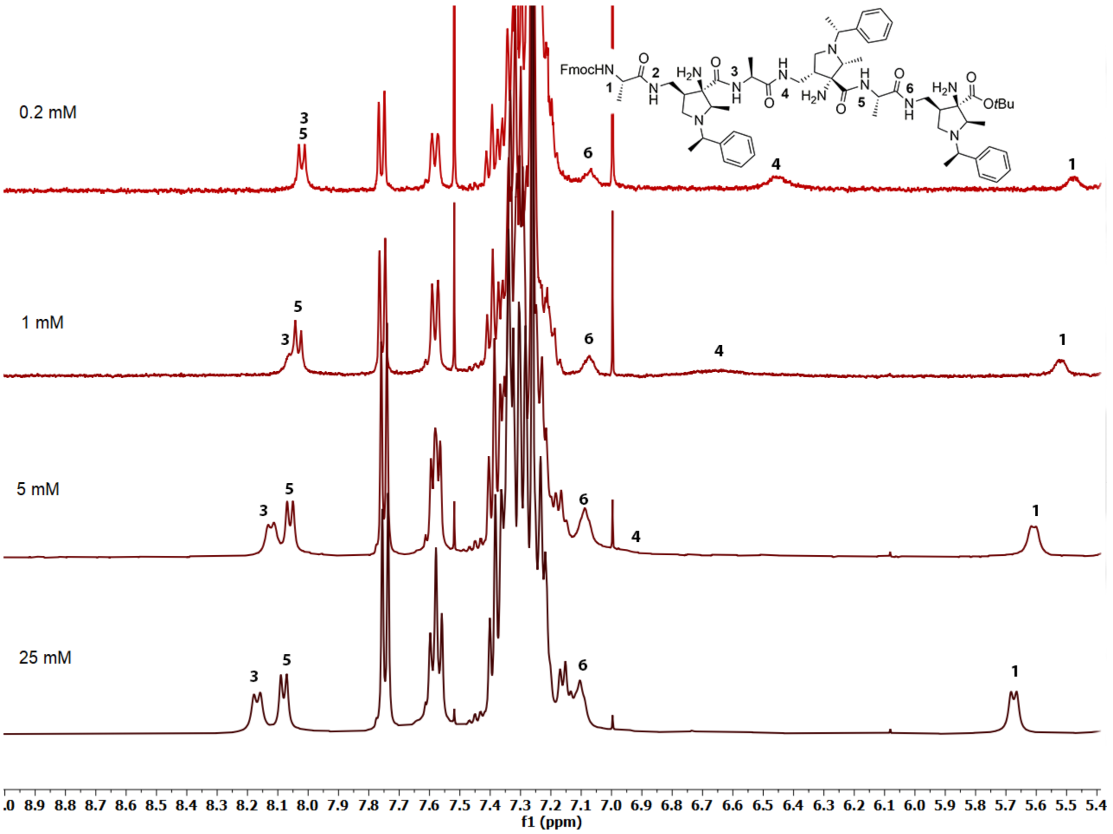

Figure S10. Concentration dependence of Fmoc(Ala-(*R*,*R*,*R*,*R*)[P]AAMP)$_3$O*t*Bu (**Fmoc-6AR[P]A**) in $CDCl_3$.

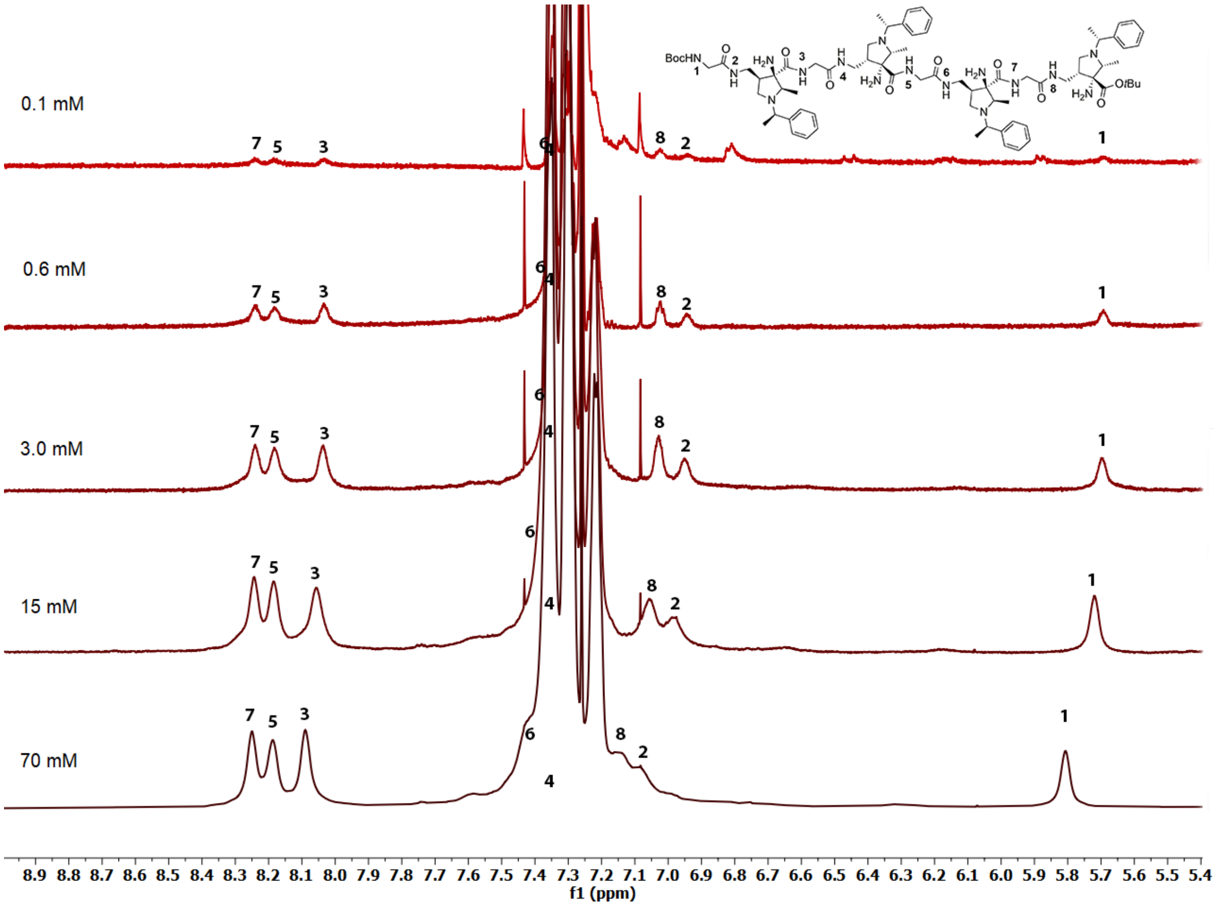

Figure S11. Concentration dependence of Boc(Gly-(*R*,*R*,*R*,*R*)^{P}AAMP)$_4$O*t*Bu (**Boc-8GR^{P}A**) in $CDCl_3$.

Peptides **Fmoc-6GR^{P}A**, **Fmoc-6AS^{P}A**, and **Boc-8GR^{P}A** displayed no significant changes over the used concentration range, suggesting concentration-independent solution structures (Figures S8,S9,S11). In contrast, peptide **Fmoc-6AR^{P}A** proved to be influenced by concentration changes; amide protons N3-H and N5-H overlapped at lower concentrations (<1mM) (Figure S10). The central N4-H proton exhibited a significant downfield shift over the concentration range, and additionally, it was significantly broadened with increasing concentration until it completely disappeared at the highest concentrations (25 mM). The NH-2 proton was too broad to be detectable at all. This behavior of peptide **Fmoc-6AR^{P}A** implies the formation of concentration-dependent intermolecular interactions; nevertheless, the presence of intramolecular hydrogen bond network involving both N3-H and N5-H is also evident.

c) DMSO Titration

Peptides **Fmoc-6^P** and **Boc-8GR^{P}A**, **Boc-8AS^{P}A** were dissolved to a 15 mM solution in $CDCl_3$ (500 µL, 0.03% TMS). DMSO-d_6 (5-100 µL) was injected and the NMR tube was shaken to ensure homogeneity (Figures S12-S16).

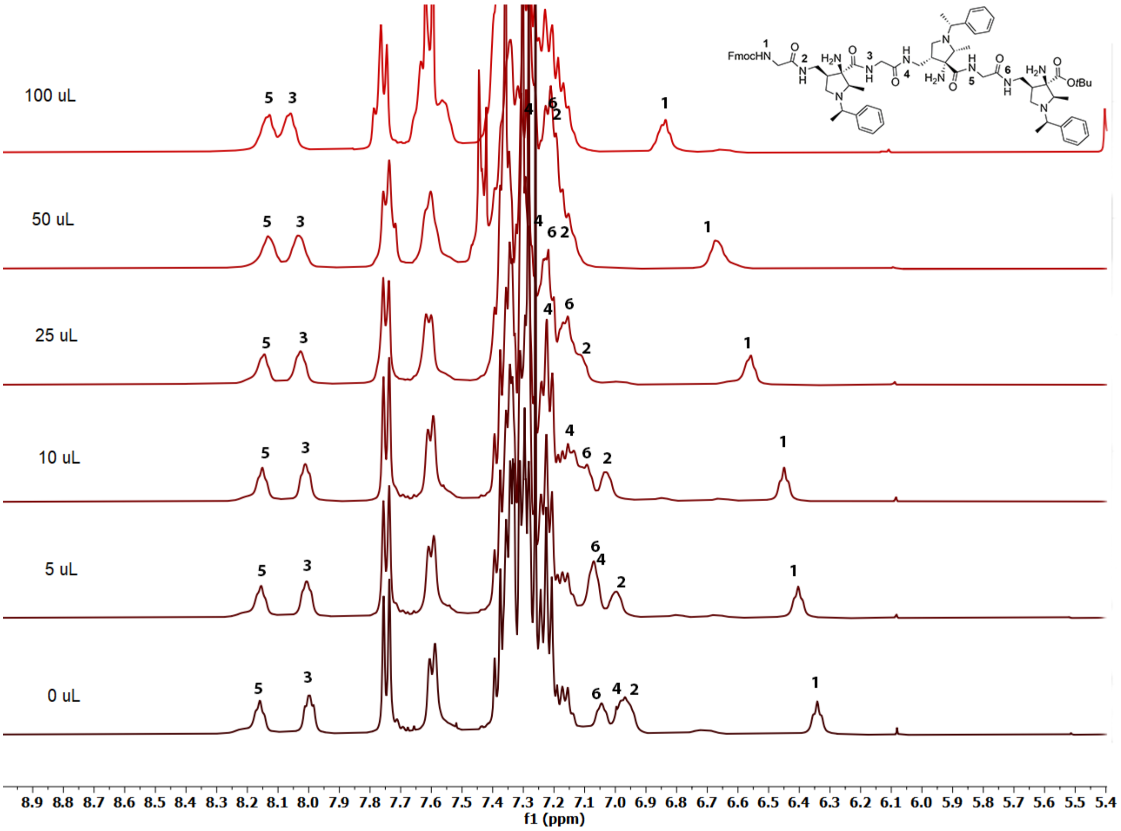

Figure S12. Titration of a 15 mM $CDCl_3$ solution of Fmoc(Gly-(*R*,*R*,*R*,*R*)^{P}AAMP)$_3$O*t*Bu (**Fmoc-6GR^{P}A**) by DMSO-d_6.

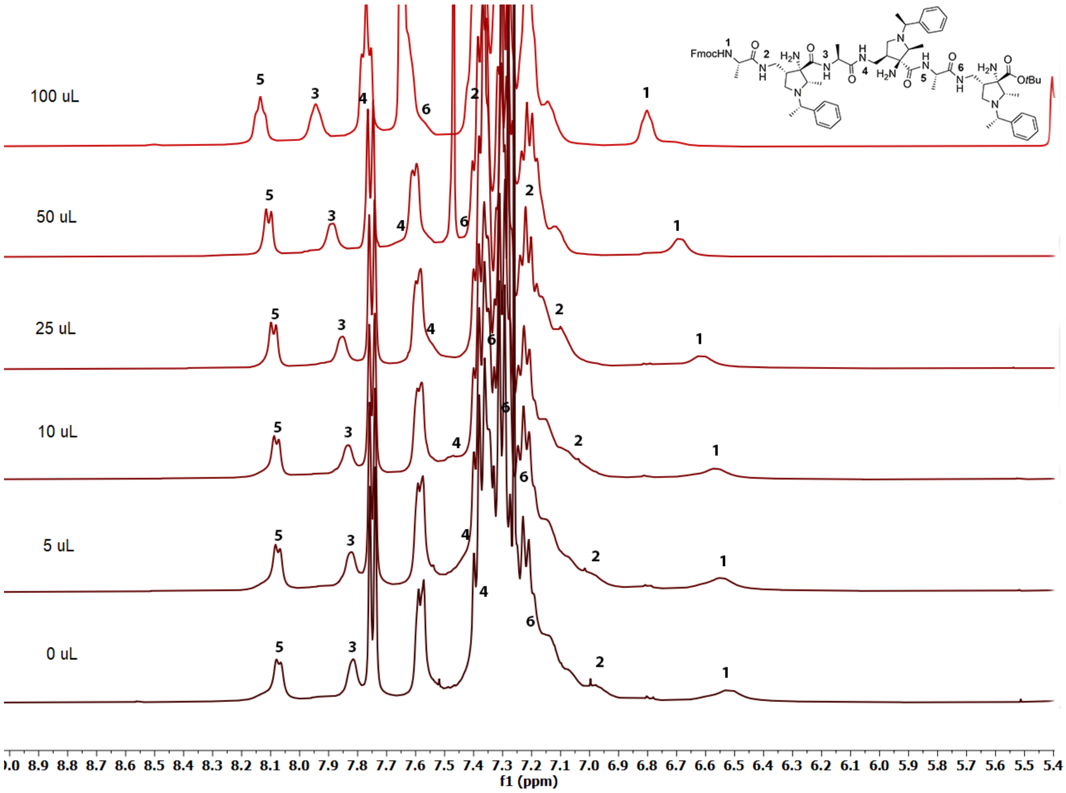

Figure S13. Titration of a 15 mM $CDCl_3$ solution of Fmoc(Ala-(*S*,*S*,*S*,*S*)^{P}AAMP)$_3$O*t*Bu (**Fmoc-6AS^{P}A**) by DMSO-d$_6$.

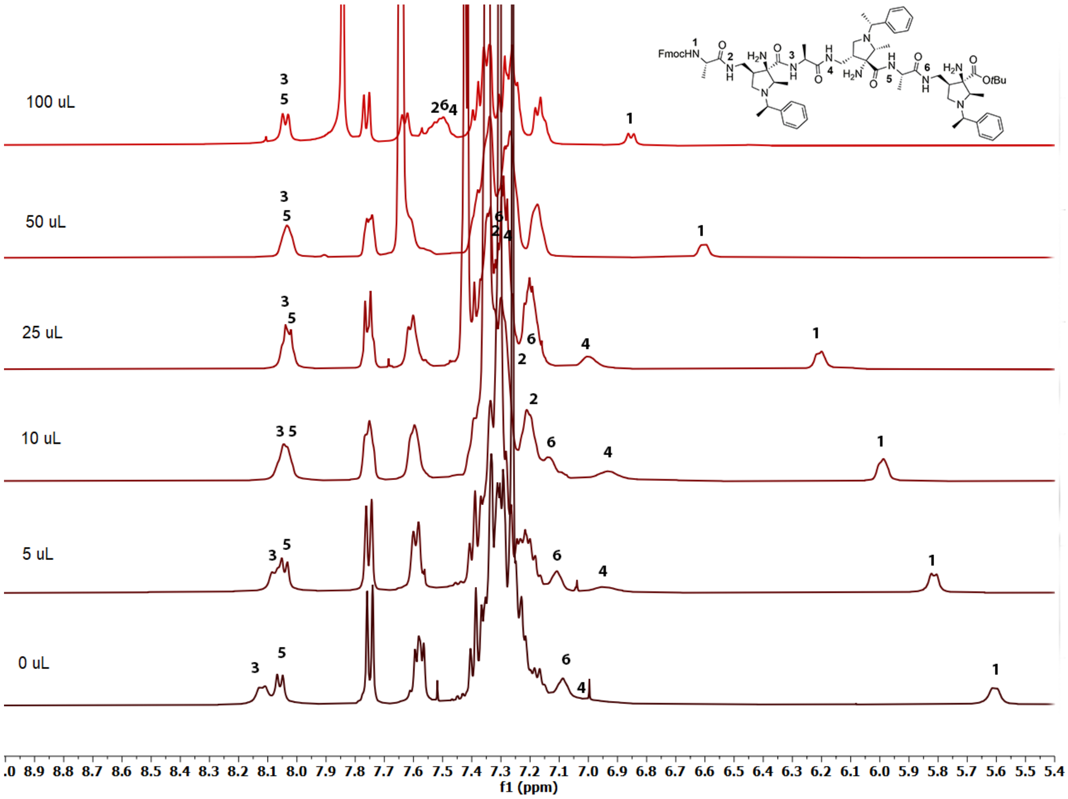

Figure S14. Titration of a 15 mM $CDCl_3$ solution of Fmoc(Ala-(*R*,*R*,*R*,*R*)^{P}AAMP)$_3$O*t*Bu (**Fmoc-6AR^{P}A**) by DMSO-d$_6$.

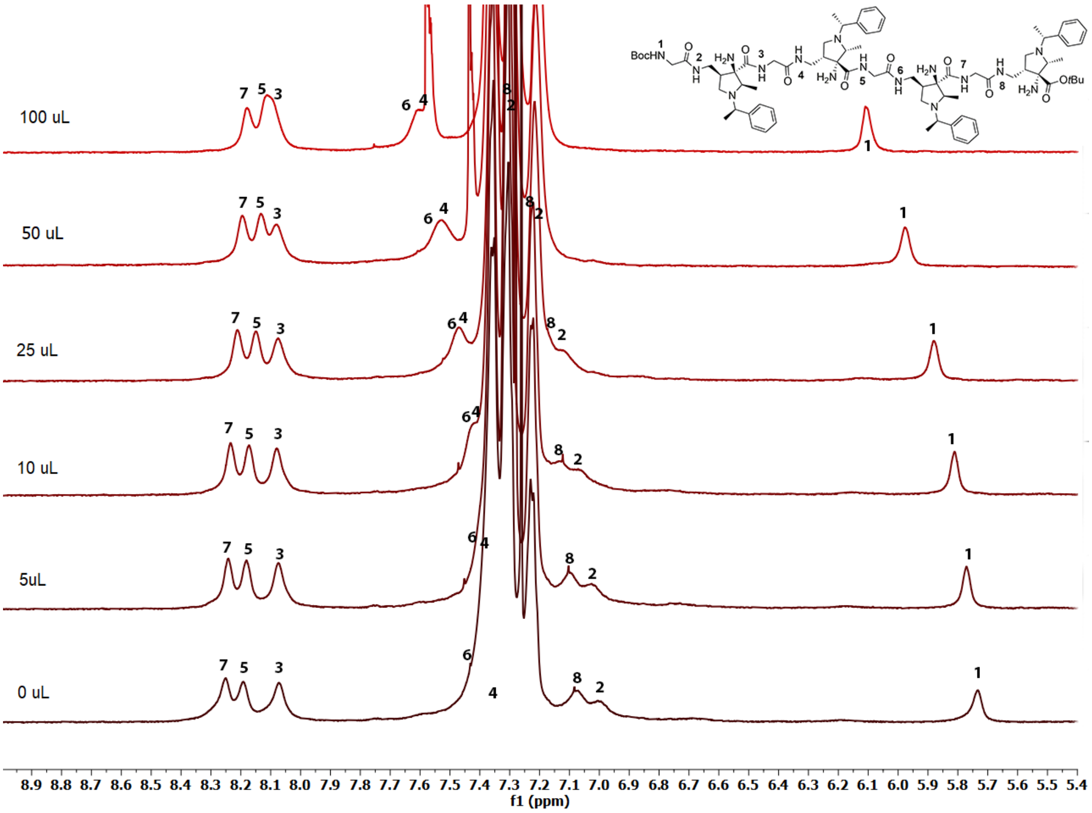

Figure S15. Titration of a 15 mM $CDCl_3$ solution of Boc(Gly-(*R*,*R*,*R*,*R*)^{P}AAMP)$_4$O*t*Bu (**Boc-8GR^{P}A**) by DMSO-d_6.

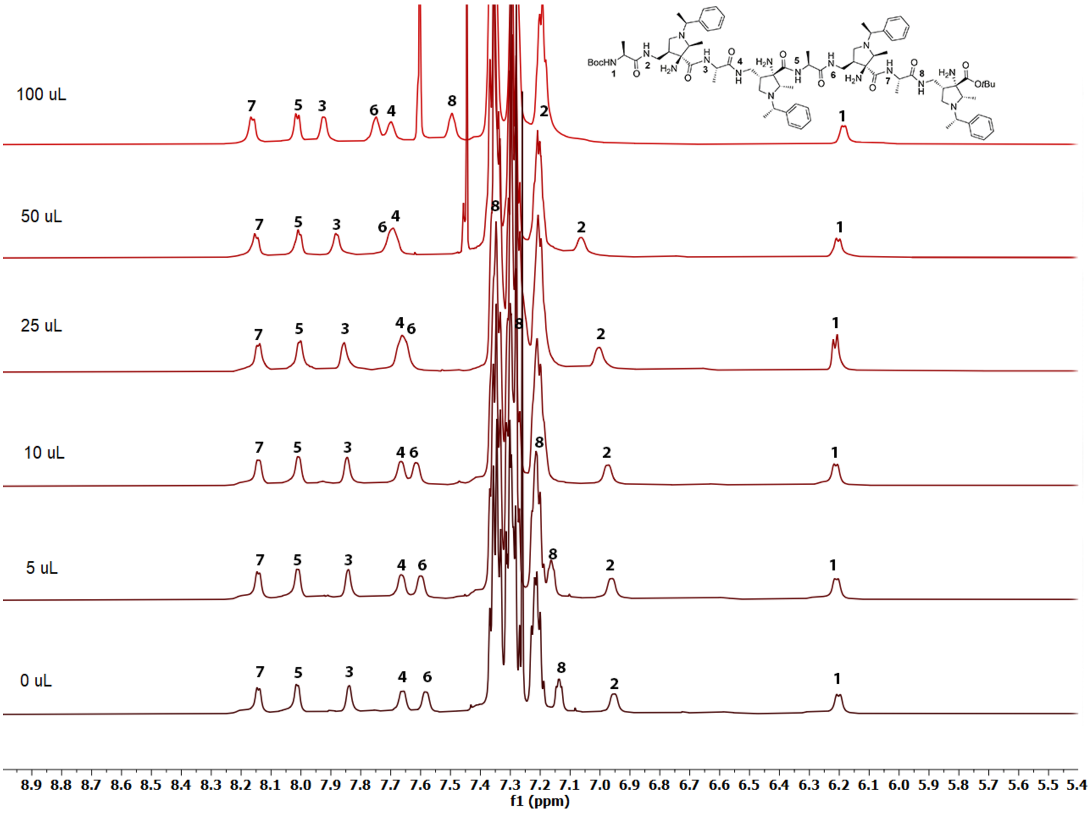

Figure S16. Titration a of 15 mM $CDCl_3$ solution of Boc(Ala-(*S*,*S*,*S*,*S*)^{P}AAMP)$_4$O*t*Bu (**Boc-8AS^{P}A**) by DMSO-d_6.

Peptides **Fmoc-6GR^{P}A** and **Fmoc-6AS^{P}A** exhibited only slight downfield shifts of amide proton chemical shifts during titration by DMSO (Figures S12,S13). Most affected amide protons are those being not adjacent to the free α-amino groups; however, the observed shifts do not indicate a significant change in the peptide secondary structure. Additionally, octapeptides **Boc-8GR^{P}A** and **Boc-8AS^{P}A** showed even less spectral changes during DMSO titration, suggesting increasing structural stability with increasing peptide length (Figures S15,S16). Conversely, peptide **Fmoc-6AR^{P}A** proved to be significantly affected during titration by DMSO, which is manifested as a large 2 ppm downfield shift with an exponential trend of the NH-1 resonance, the local minimum of the chemical shift of the NH-4 proton at the addition of 10 μL of DMSO, and the appearance of the NH-2 proton resonance only after addition of larger than 10 μL DMSO (Figure S14). These results indicate highly dynamic secondary arrangements at the N-terminal and central regions in hexamer **Fmoc-6AR^{P}A**.

d) Temperature dependence

Peptides **Fmoc-6^P** and **Boc-8GR^{P}A**, **Boc-8AS^{P}A** were dissolved to a 15 mM solution in $CDCl_3$ (500 μL, 0.03% TMS) for temperature dependence experiments (Figures S17-S21). The temperature dependence was measured from –53 to 27 °C for hexamers and from –53 to 47 °C for octamers.

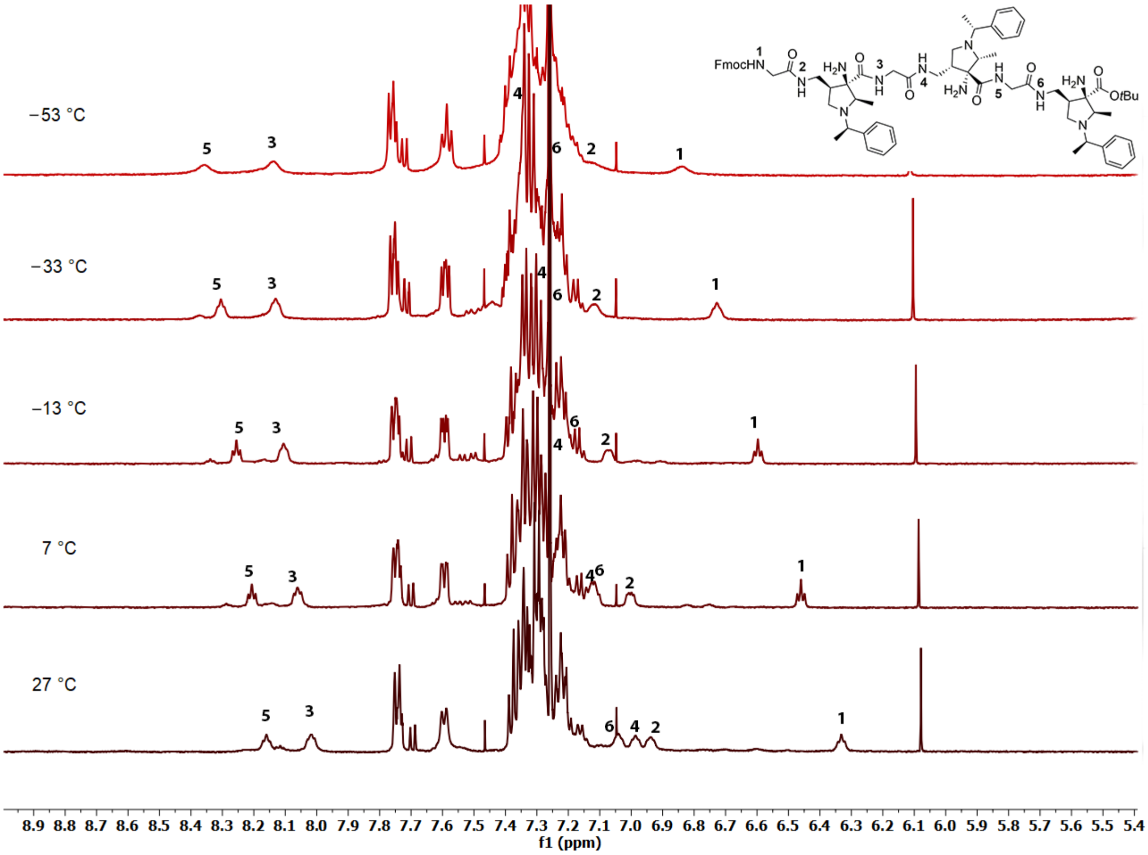

Figure S17. Temperature dependence of amide resonances in a 15 mM $CDCl_3$ solution of Fmoc(Gly-(*R*,*R*,*R*,*R*)^{P}AAMP)$_3$O*t*Bu (**Fmoc-6GR^{P}A**).

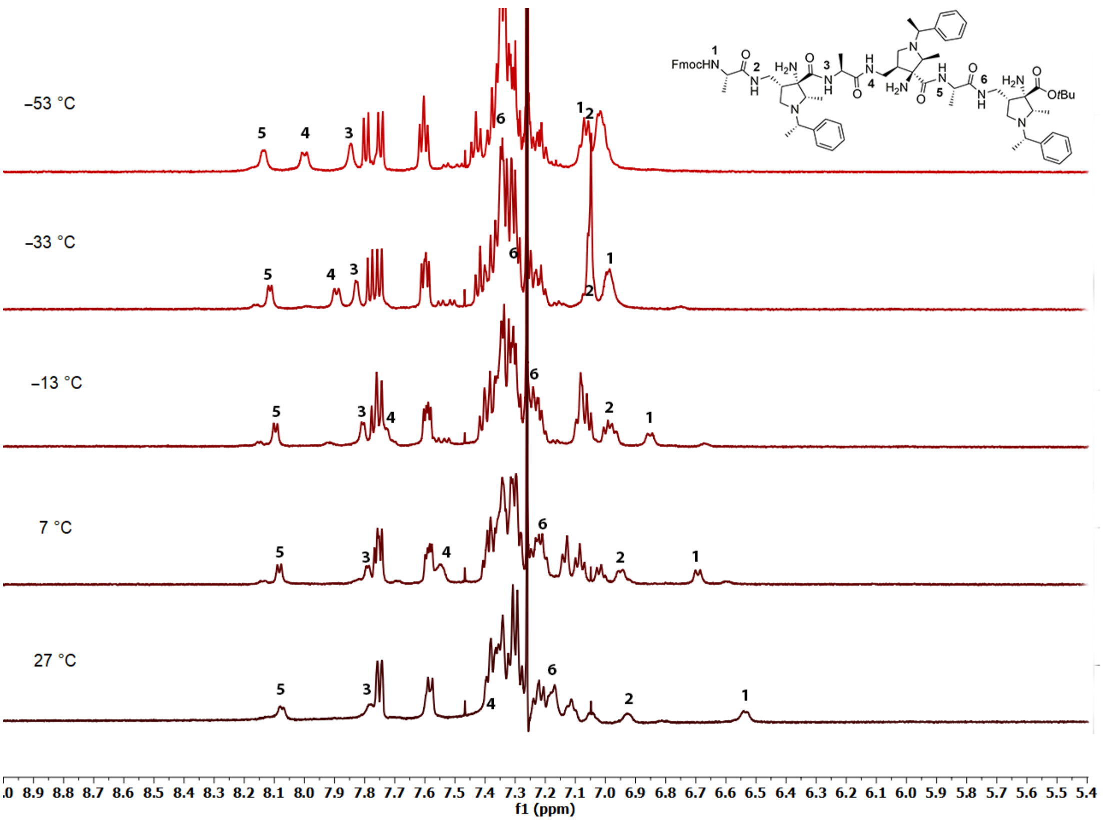

Figure S18. Temperature dependence of amide resonances in a 15 mM $CDCl_3$ solution of Fmoc(Ala-(*S*,*S*,*S*,*S*)^{P}AAMP)$_3$O*t*Bu (**Fmoc-6AS^{P}A**).

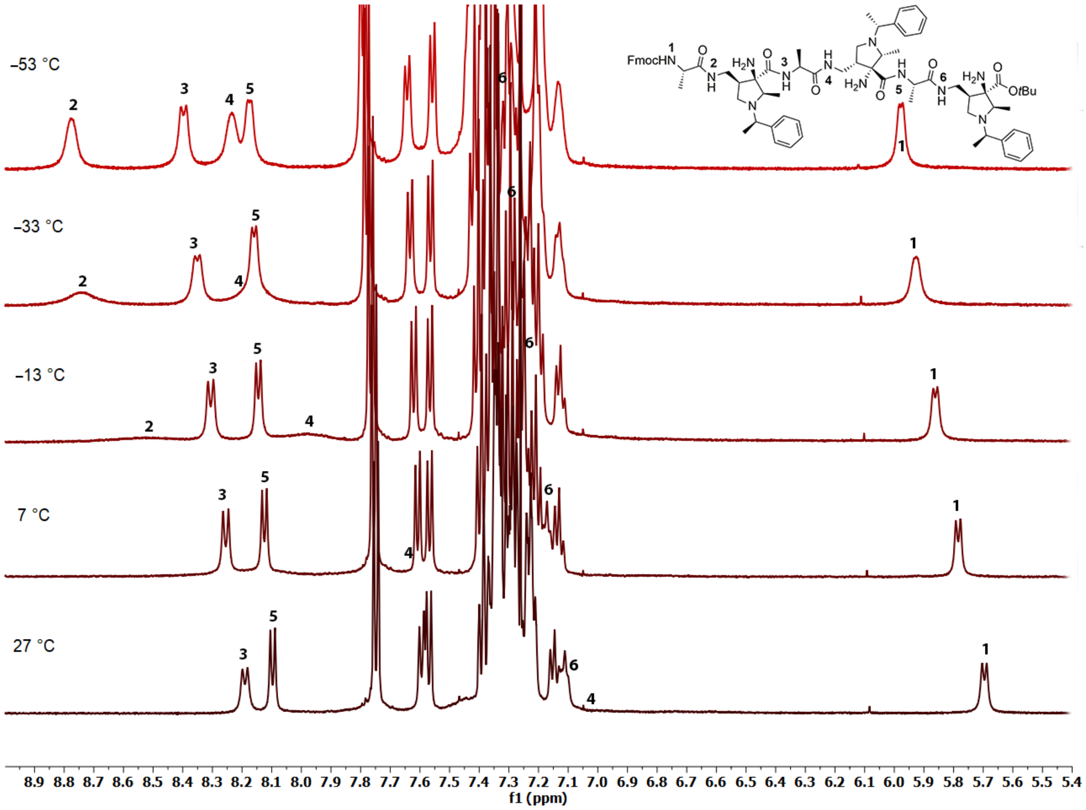

Figure S19. Temperature dependence of amide resonances in a 15 mM $CDCl_3$ solution of Fmoc(Ala-(*R*,*R*,*R*,*R*)^{P}AAMP)$_3$O*t*Bu (**Fmoc-6AR^{P}A**).

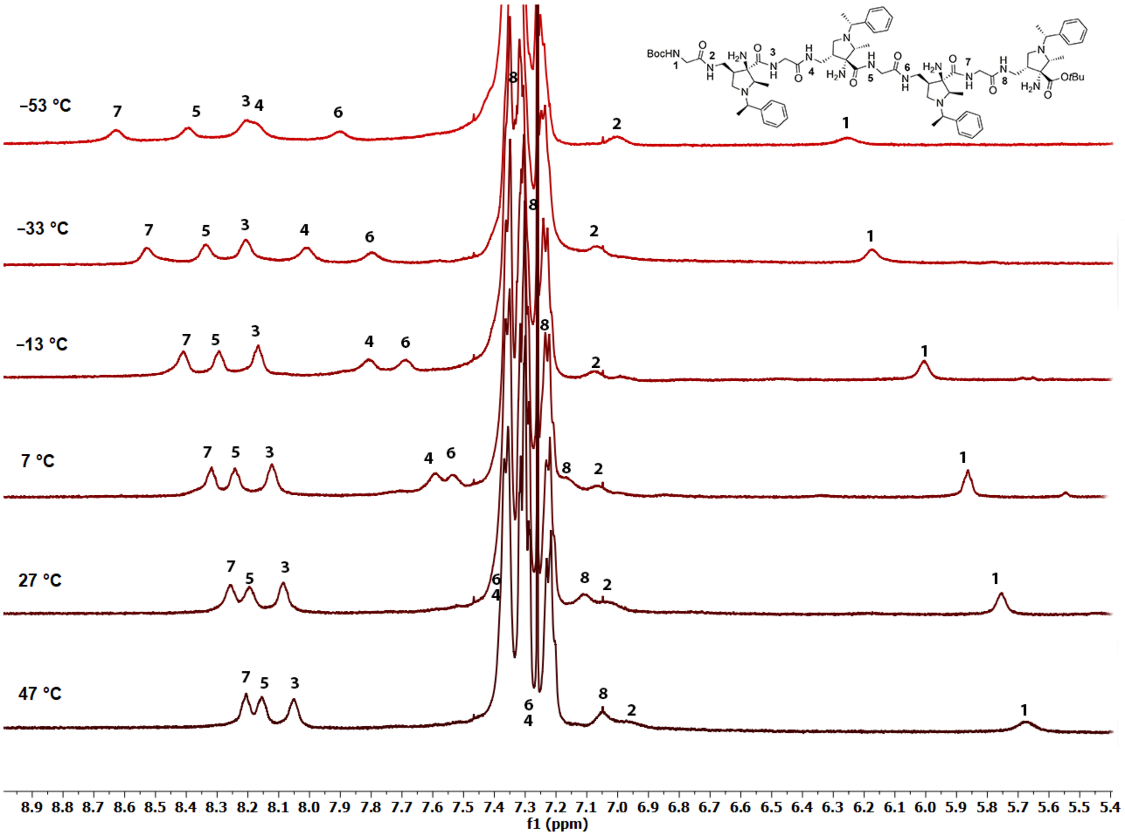

Figure S20. Temperature dependence of amide resonances in a 15 mM $CDCl_3$ solution of Boc(Gly-(*R*,*R*,*R*,*R*)^{P}AAMP)$_4$O*t*Bu (**Boc-8GR^{P}A**).

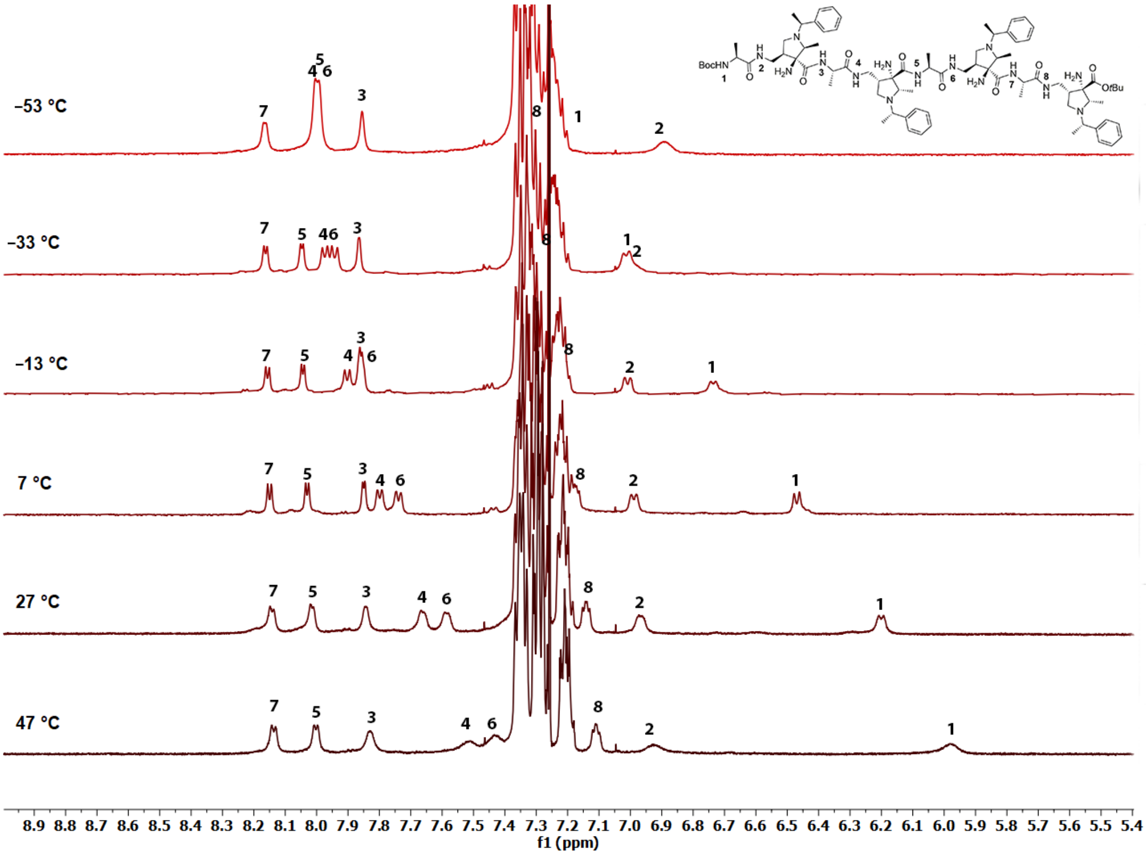

Figure S21. Temperature dependence of amide resonances in a 15 mM $CDCl_3$ solution of Boc(Ala-(*S*,*S*,*S*,*S*)^{P}AAMP)$_4$O*t*Bu (**Boc-8AS^{P}A**).

Peptides **Fmoc-6GR^{P}A**, **Fmoc-6AS^{P}A**, and **Boc-8GR^{P}A**, **Boc-8AS^{P}A** showed only small N-H proton upfield chemical shift changes by maximally 0.5 ppm overall deviation as the temperature increased, suggesting only slight temperature dependence (Figures S17,S18,S20,S21). In contrast, peptide **Fmoc-6AR^{P}A** showed a dramatic upfield shift of N2-H and N4-H simultaneously with signal broadening as the temperature increased (Figure S19). Additionally, the N2-H proton completely disappeared at higher temperatures (7-27 °C). These large changes on N-H proton chemical shifts imply significant conformational changes.

e) AcOH Titration

Peptides **Boc-8GR^{P}A**, **Boc-8AS^{P}A** were dissolved to a 15 mM solution in $CDCl_3$ (500 µL, 0.03% TMS) dried over 3Å molecular sieves. AcOH (1-8 equivalents) was injected, and the NMR tube was shaken to ensure homogeneity (Figures S22-S24).

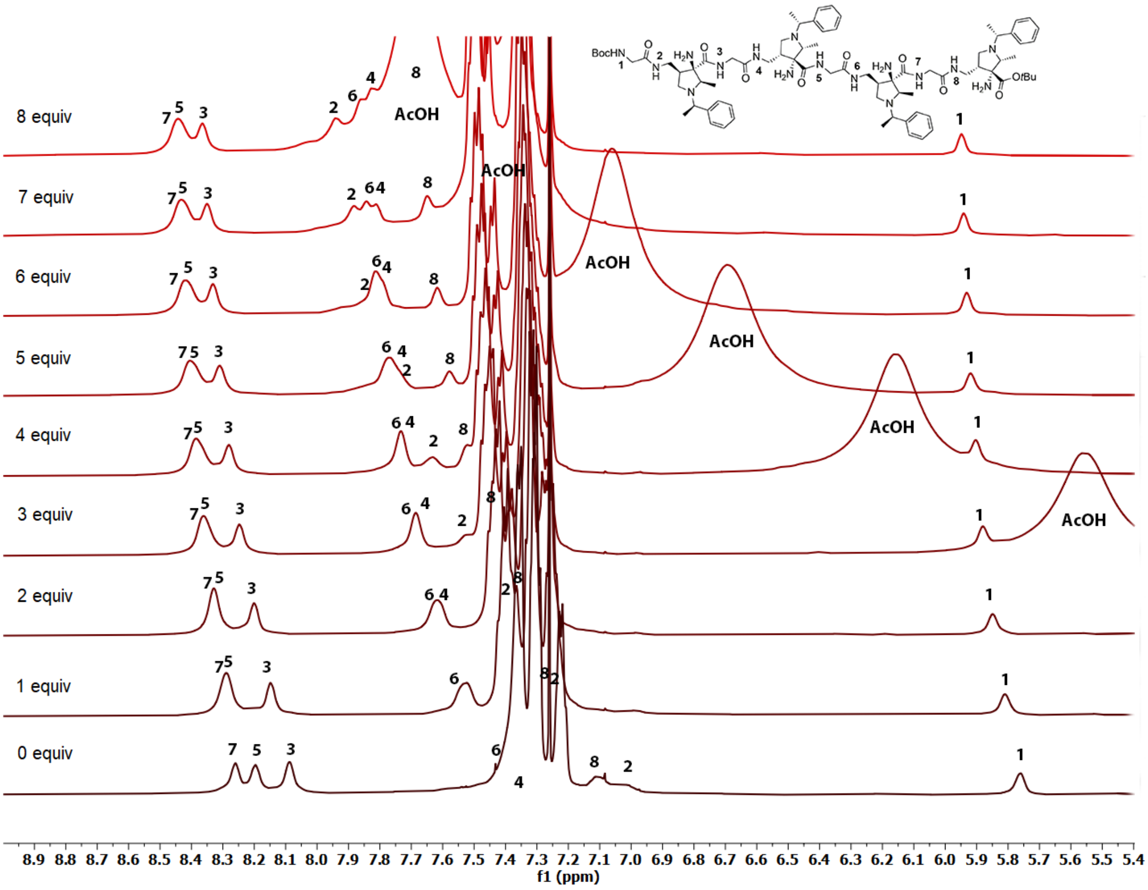

Figure S22. Titration of a 15 mM $CDCl_3$ solution of Boc(Gly-(*R*,*R*,*R*,*R*)^{P}AAMP)$_4$O*t*Bu (**Boc-8GR^{P}A**) by AcOH.

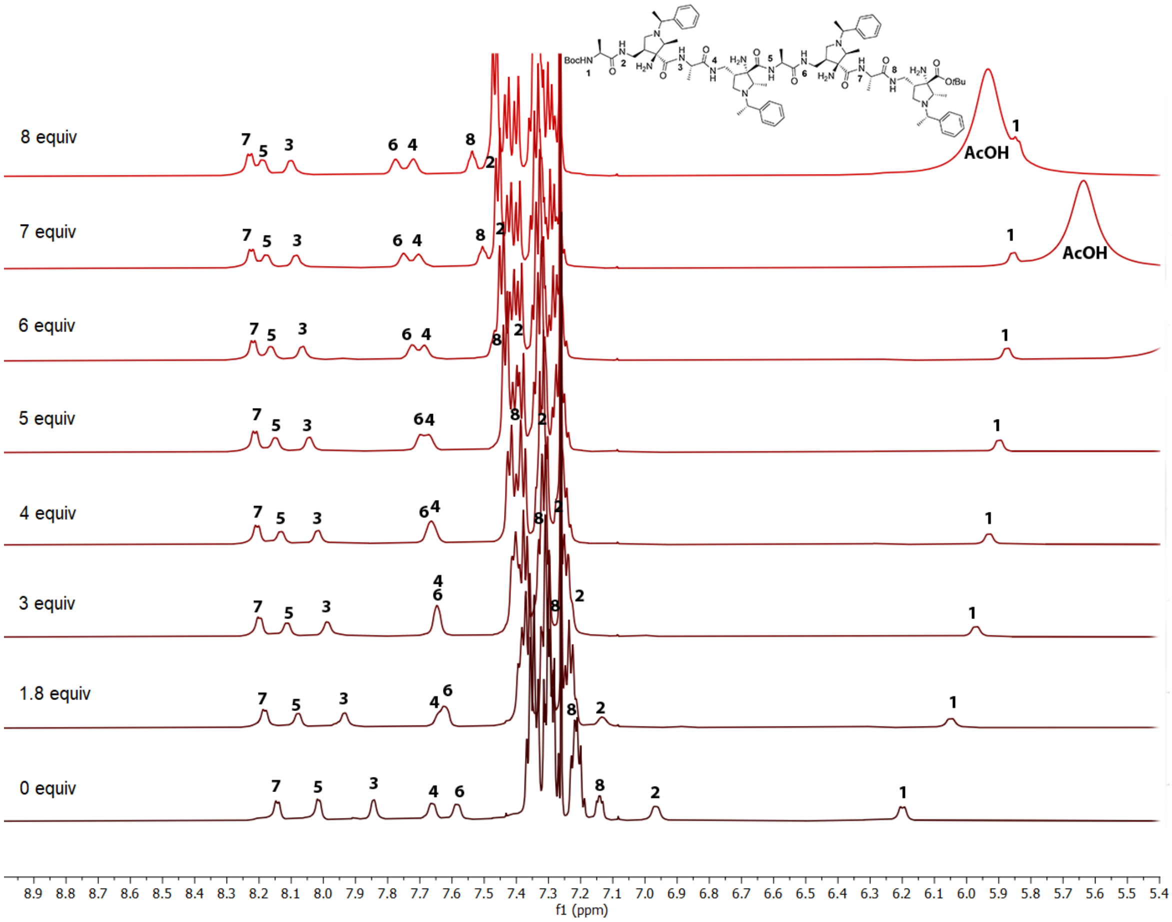

Figure S23. Titration of a 15 mM $CDCl_3$ solution of Boc(Ala-(*S*,*S*,*S*,*S*)^{P}AAMP)$_4$O*t*Bu (**Boc-8AS^{P}A**) by AcOH.

Amide protons N3-H, N5-H, and N7-H adjacent to NH_2 groups showed minimal deviation during titration by AcOH for both peptides **Boc-8GR^{P}A**, **Boc-8AS^{P}A**, implying the weak basicity of free primary amino group since their protonation should affect the intramolecular five-membered NH(*i*)···NH_2(*i*–1) interaction (Figures S22,S23).

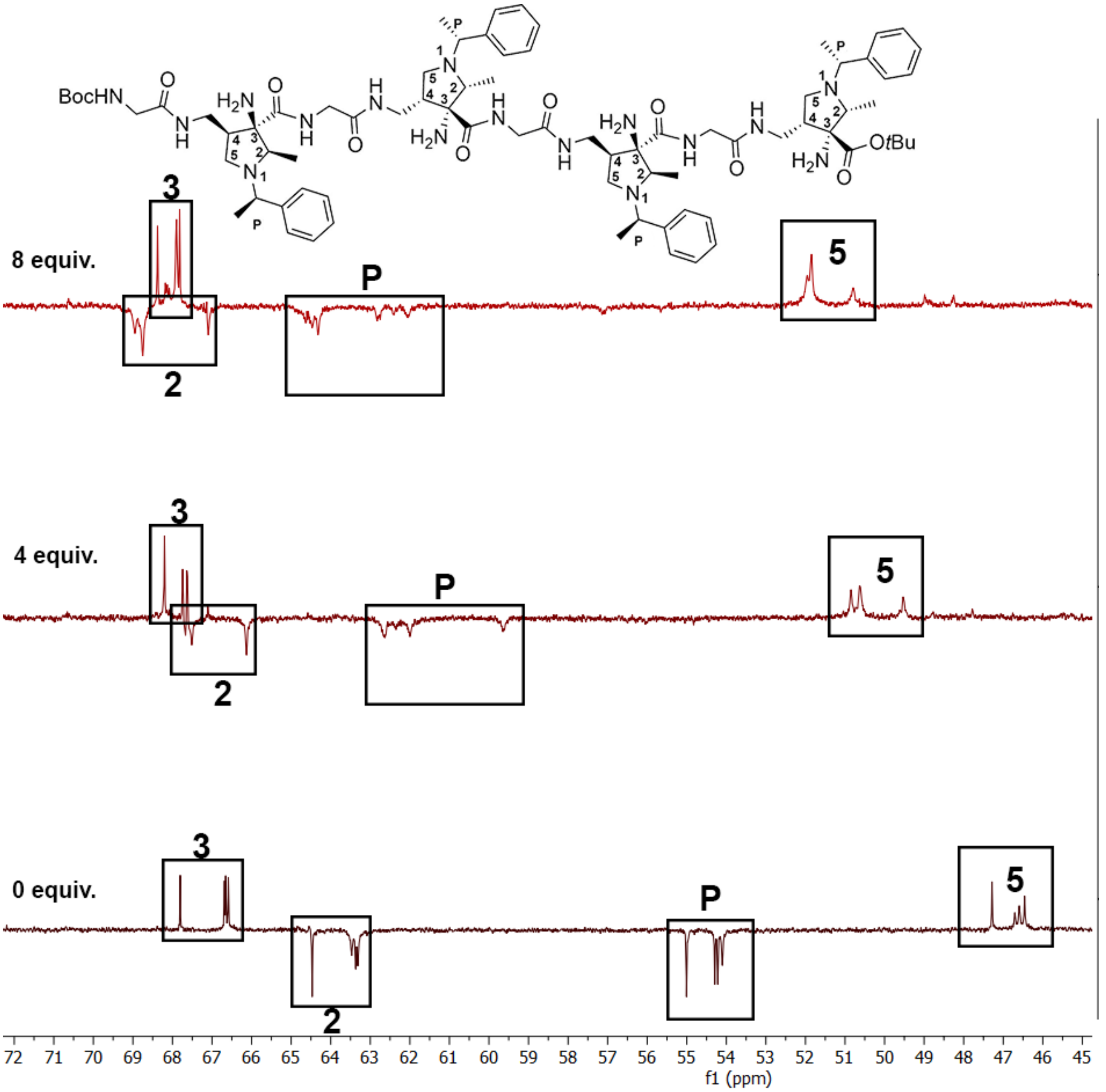

Figure S24. Comparison of ^{13}C NMR spectra of Boc(Gly-(*R*,*R*,*R*,*R*)^{P}AAMP)$_4$O*t*Bu (**Boc-8GR^{P}A**) in $CDCl_3$ after addition of 4 and 8 equivalents of AcOH.

All carbon atoms neighboring the pyrrolidine nitrogen atoms displayed a significant downfield shift after addition of four and eight equivalents of acetic acid (Figures S24). Carbon atoms C-5 were found as a set of signals in the range of 46.4-47.3 ppm; they were downfield shifted to 49.5-50.8 ppm after addition of the first four equivalents of AcOH and to 50.8-51.9 ppm after another four equivalents of AcOH. Carbon atoms C-2 were found as a set of signals in the range of 64.4-63.3 ppm; they were downfield shifted to 67.6-66.1 ppm after addition 4 equiv. AcOH and to 68.8-67.1 ppm with 8 equiv. of AcOH. The CH carbon atoms of the phenylethyl (PE) groups were found as a set of signals in the range of 55.0-54.1 ppm; they were downfield shifted to 62.6-59.6 ppm with 4 equiv. of AcOH and to 64.4-62.0 ppm with 8 equiv. of AcOH, respectively. Additionally, these signals experienced significant

broadening on addition of four equiv. AcOH. In contrast, the carbon atoms bearing the free primary amino group showed an only negligible downfield shift from 67.8-66.6 ppm to 68.2-67.7 ppm after the first four equivalents of AcOH and no further shift after the addition of a total of eight equivalents of AcOH. Moreover, these signals remained sharp on addition of of AcOH. This spectral behavior implies a selective protonation at the pyrrolidine nitrogen atoms, while the free primary amino groups are not protonated.

f) HCl addition

Peptides **Boc-8GR^{P}A**, **Boc-8AS^{P}A** were dissolved to a 15 mM solution in $CDCl_3$ (500 µL), HCl (6 µL, 7.5 mmol, 1.25 M solution in MeOH) was added and the mixture was concentrated *in vacuo* (Figure S25,S26). The resulting salt was redissolved in $CDCl_3$ (500 µL, 0.03% TMS). To rule out potential peptide decomposition by HCl solution, the solution was reextracted with saturated $NaHCO_3$ solution after NMR acquisition. The original peptides were fully recovered.

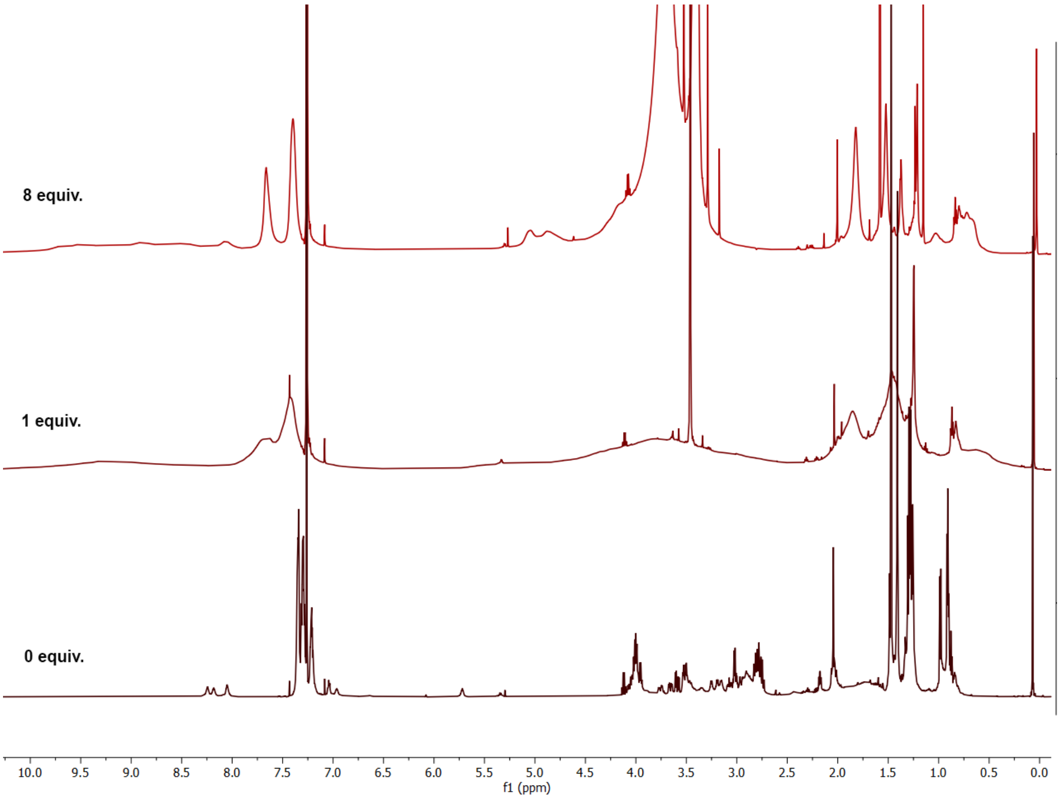

Figure S25. Addition of a HCl solution (1 equiv. in MeOH) to a 15 mM $CDCl_3$ solution of Boc(Gly-(*R*,*R*,*R*,*R*)^{P}AAMP)$_4$O*t*Bu (**Boc-8GR^{P}A**).

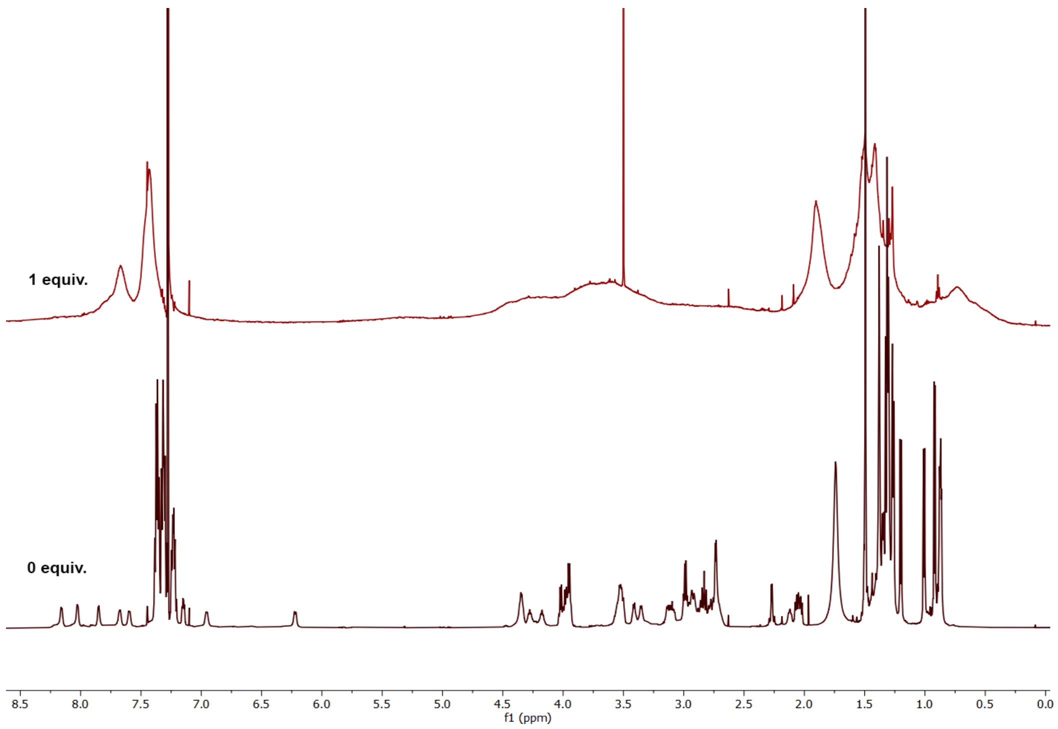

Figure S26. Addition of a HCl solution (1 equiv. in MeOH) to a 15 mM $CDCl_3$ solution of Boc(Ala-(*S*,*S*,*S*,*S*)^{P}AAMP)$_4$O*t*Bu (**Boc-8AS^{P}A**).

Peptides **Boc-8GR^{P}A**, **Boc-8AS^{P}A** showed strong broadening of all signals after addition of HCl solution (1 equiv. in MeOH). To rule out that the spectral broadening is a result of rapid proton exchange, further HCl (4 and 8 equiv. in MeOH) was added; however, although the ^{1}H NMR spectrum changed, the unstructured broad signals remained indicating destruction of the secondary structure.

g) Comparison of spectral regions of peptides Ac-6GR^{H}A and Ac-6SGDab at different pH

Peptides **Ac-6GR^{H}A** and **Ac-6SGDab** were compared with respect to their chemical shifts in aqueous solution at different pH.

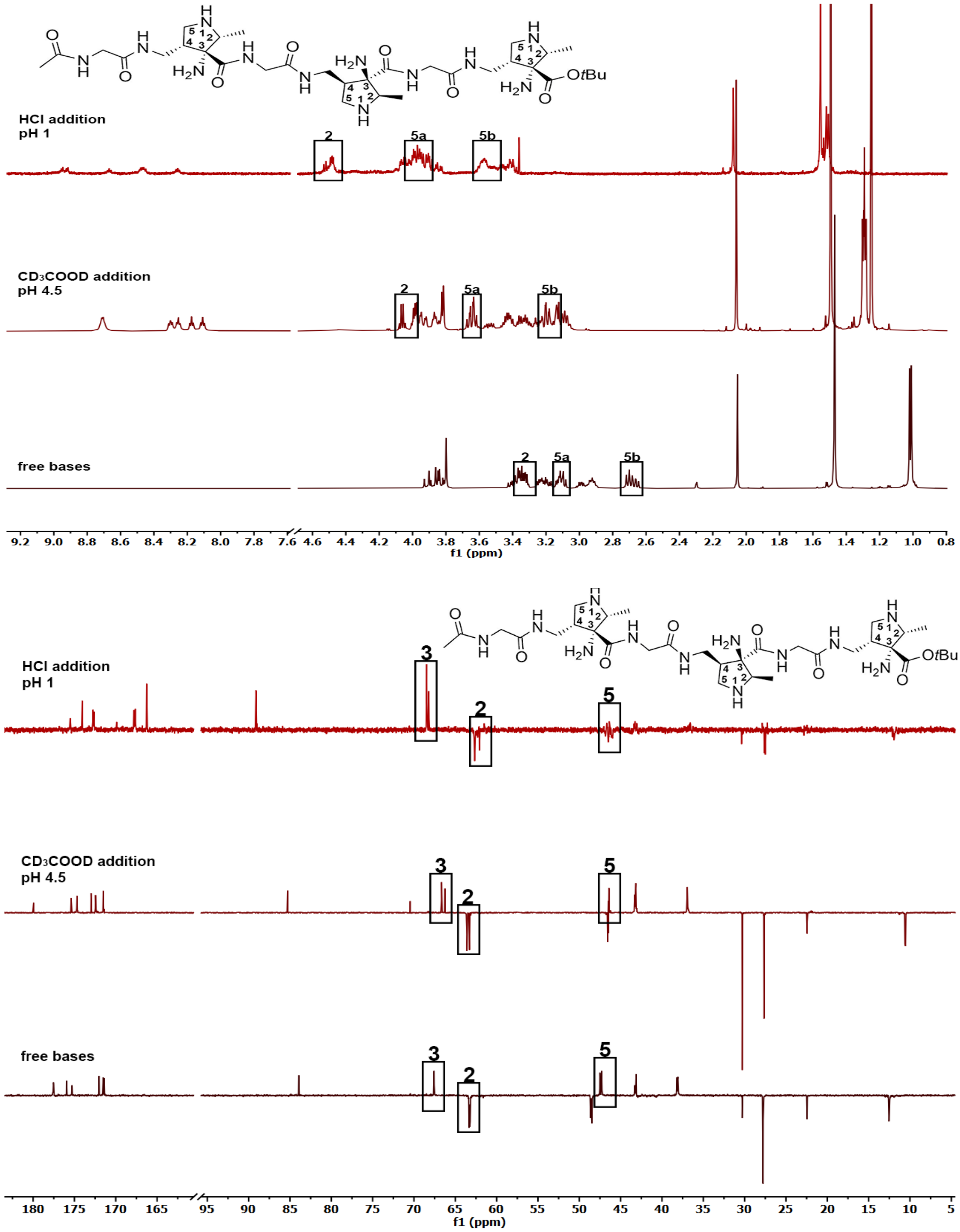

Figure S27. Acidification of a 15 mM 9:1 H_2O/D_2O solution of Boc(Gly-(*R*,*R*,*R*)AAMP)$_3$O*t*Bu (**Ac-6GR^{H}A**) by CD_3COOD and HCl and comparison of the ^{1}H NMR and ^{13}C NMR spectra.

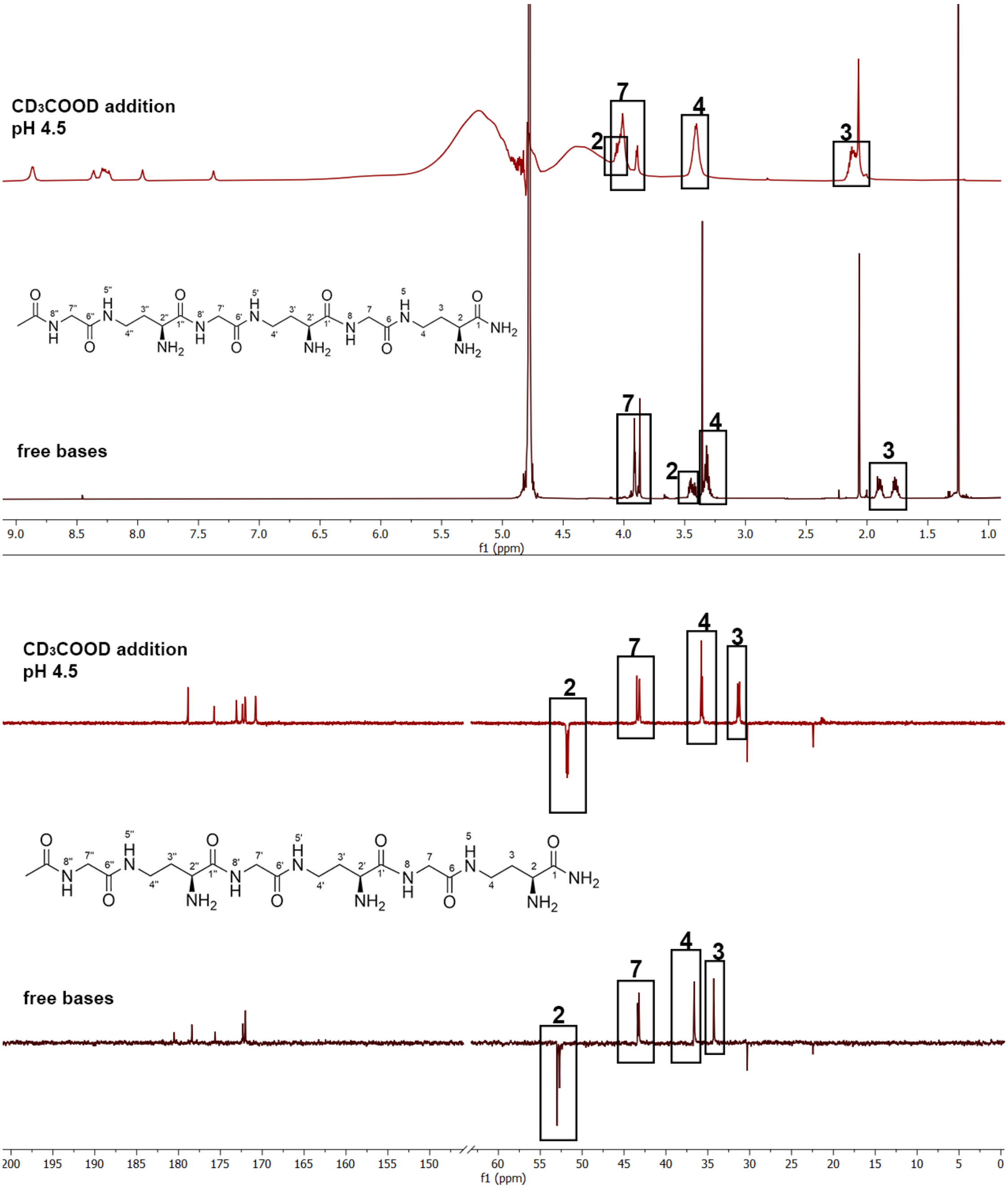

Figure S28. Acidification of a 5 mM 9:1 H_2O/D_2O solution of AcNH(Gly-L-Dab)$_3$NH$_2$ (**Ac-6SGDab**) by CD_3COOD and comparison of the ^1H NMR and ^{13}C NMR spectra.

Although the ^1H NMR spectra of **Ac-6GR^{H}A** showed a downfield shift of the CH protons adjacent to the pyrrolidine nitrogen atoms at pH 4.5 and 1 under aqueous conditions suggesting their protonation, the ^{13}C NMR spectra did not exhibit a significant chemical shift change of the adjacent carbon atoms. Similarly, ^1H NMR spectra of **Ac-6SGDab** showed a downfield shift of CH protons adjacent to the amino groups at pH 4.5, whereas the ^{13}C NMR spectra remained similar at both pH values.

h) 2D NMR ROESY experiments

All ROESY NMR data were processed with MestreNova 14.2.0 using default processing parameters and COSY-like symmetrisation. Positive cross-peaks are depicted as red contours, negative cross-peaks as blue contours.

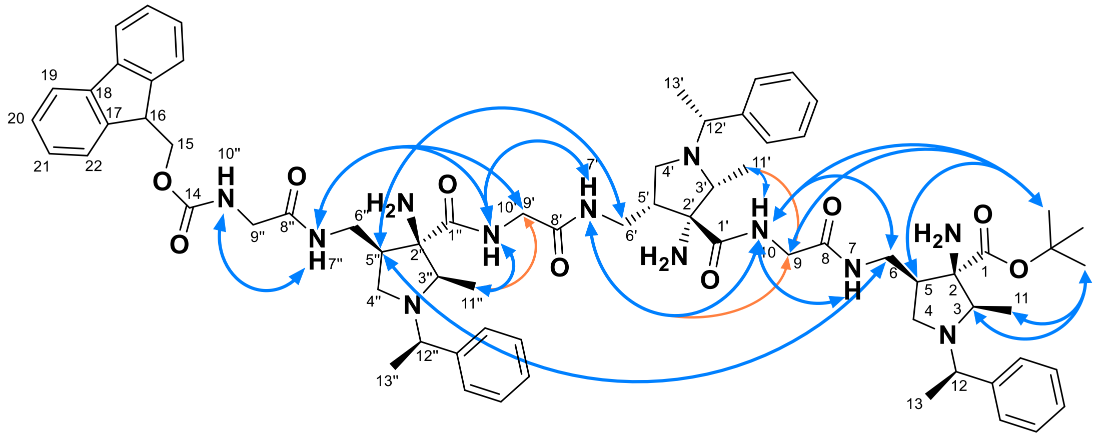

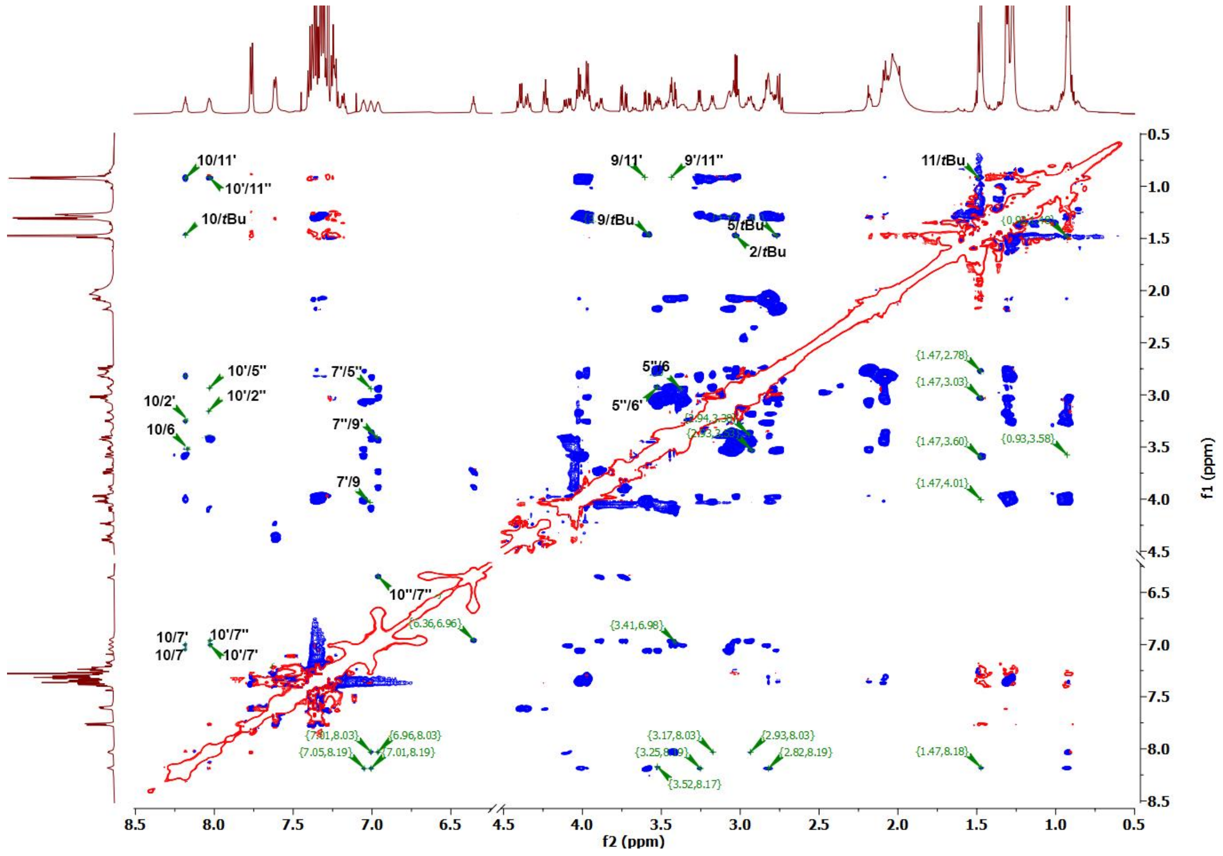

Figure S29. 2D ROESY spectrum of a 15mM $CDCl_3$ solution of Fmoc(Gly-(*R*,*R*,*R*,*R*)^{P}AAMP)$_3$O*t*Bu (**Fmoc-6GR^{P}A**).

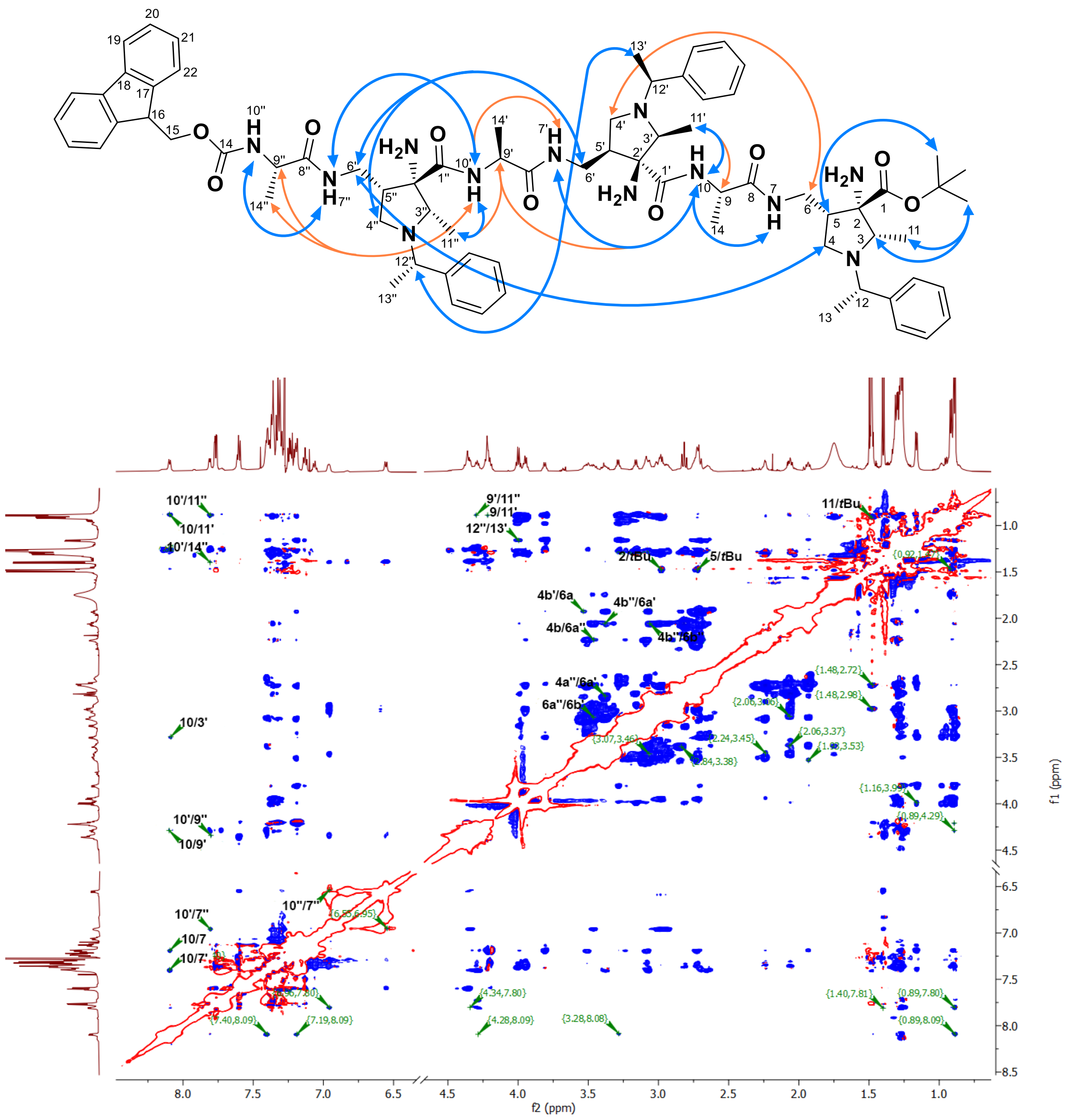

Figure S30. 2D ROESY spectrum of a 15mM $CDCl_3$ solution of Fmoc(Ala-(*S*,*S*,*S*,*S*)^{P}AAMP)$_3$O*t*Bu (**Fmoc-6AS^{P}A**).

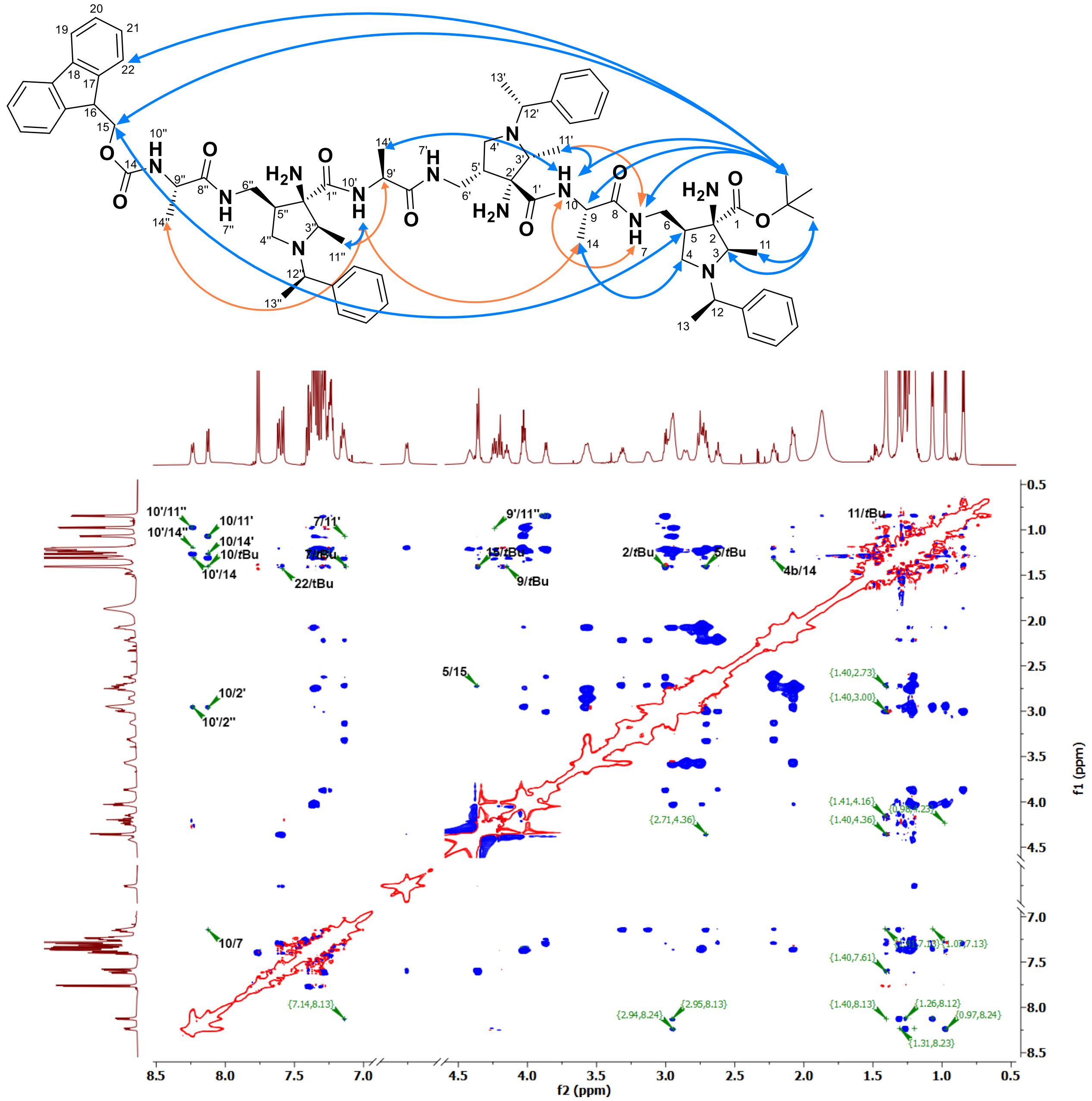

Figure S31. 2D ROESY spectrum of a 15mM $CDCl_3$ solution of Fmoc(Ala-(*R*,*R*,*R*,*R*)^{P}AAMP)$_3$O*t*Bu (**Fmoc-6AR^{P}A**).

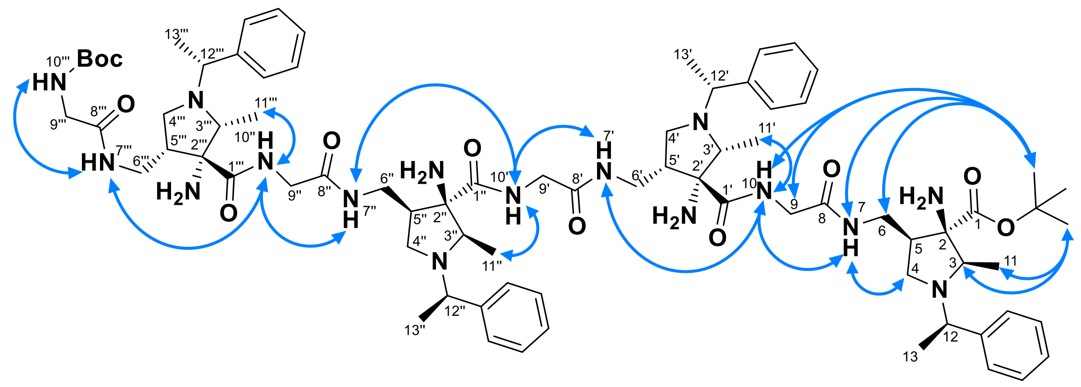

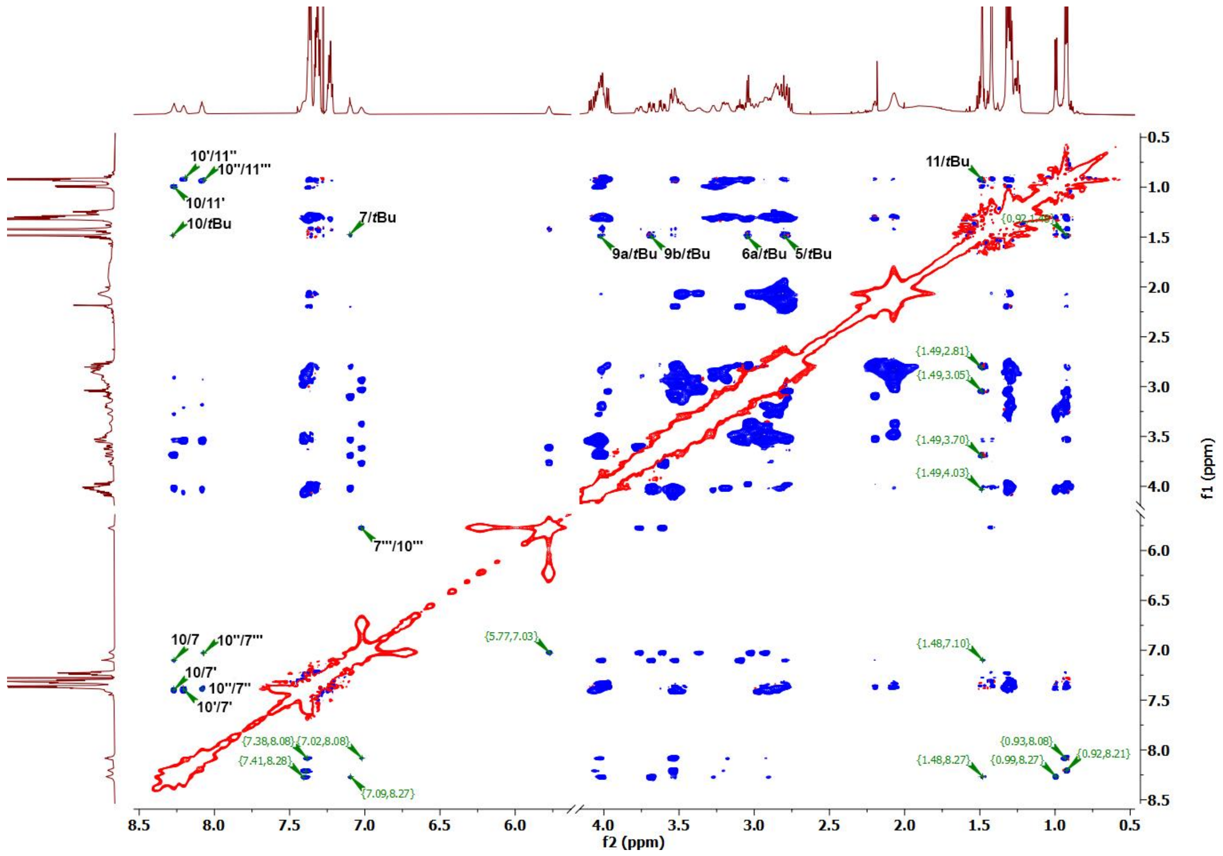

Figure S32. 2D ROESY spectrum of a 10mM $CDCl_3$ solution of Boc(Gly-(*R*,*R*,*R*,*R*)[P]AAMP)$_4$O*t*Bu (**Boc-8GR[P]A**).

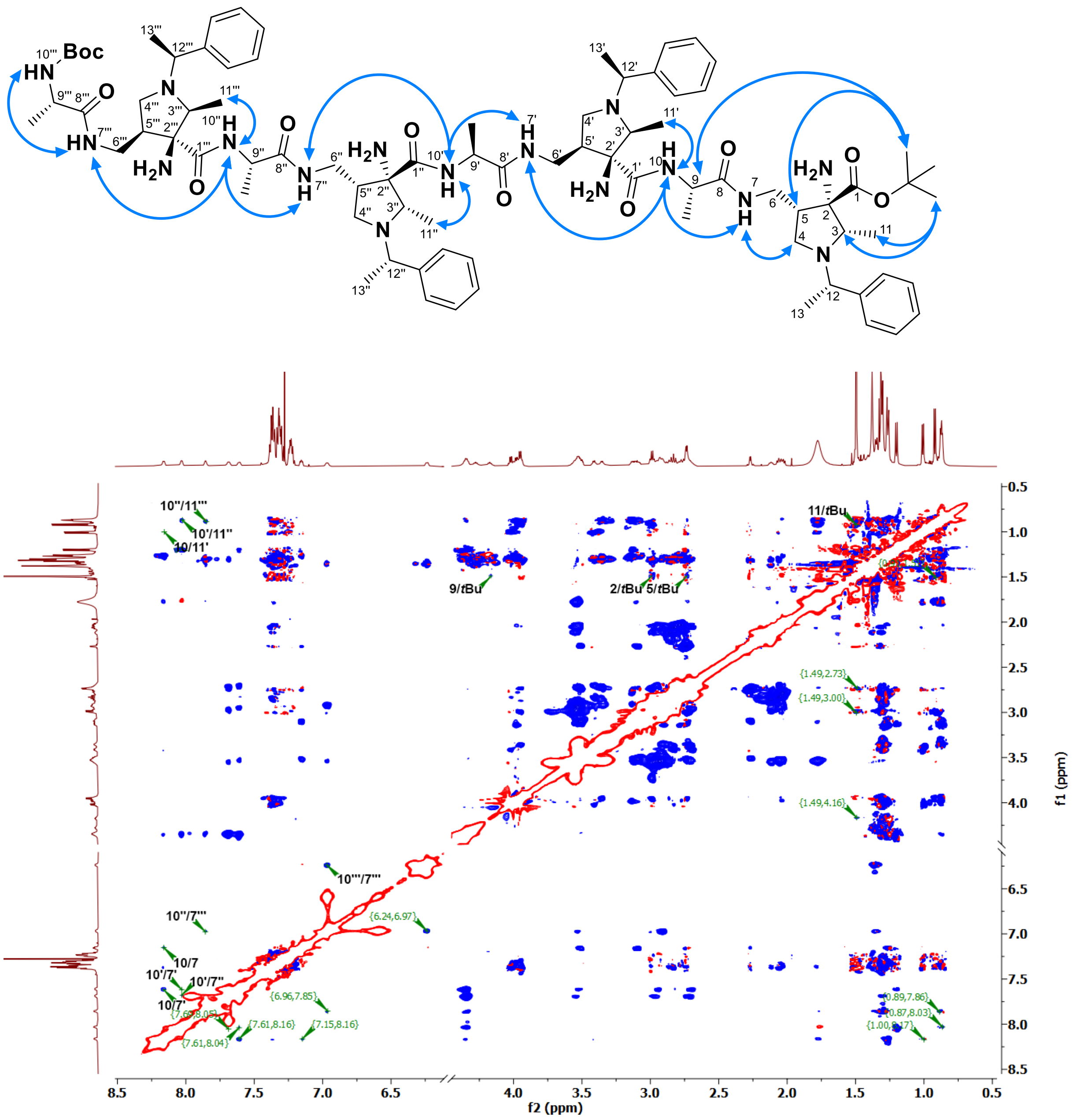

Figure S33. 2D ROESY spectrum of a 15mM $CDCl_3$ solution of Boc(Ala-(*S*,*S*,*S*,*S*)^{P}AAMP)$_4$O*t*Bu (**Boc-8AS^{P}A**).

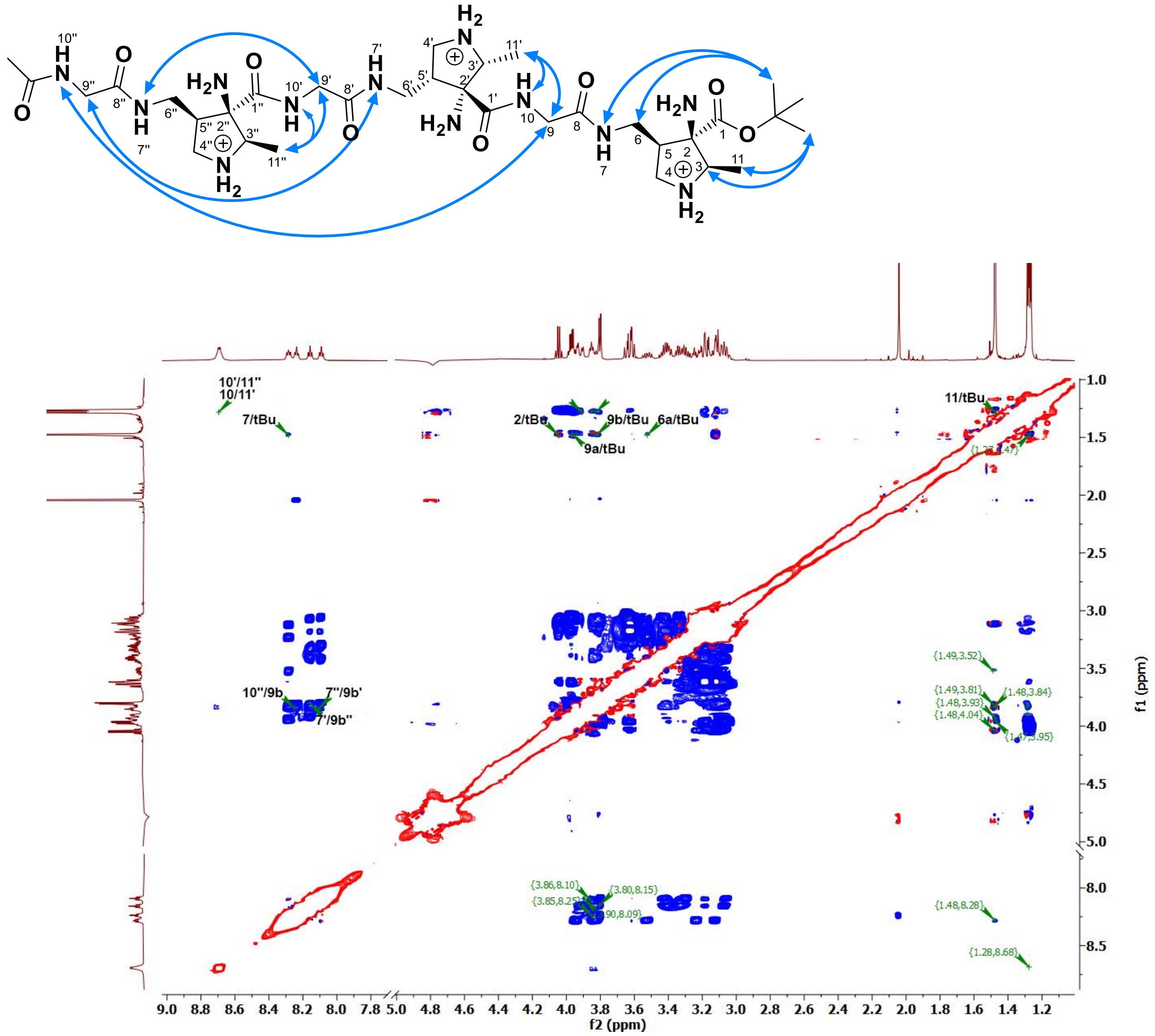

Figure S34. 2D ROESY spectrum of a 25mM H_2O/D_2O 9:1 solution of Ac(Gly-(*R*,*R*,*R*,*R*)AAMP)$_3$O*t*Bu (**Ac-6GR^{H}A**) acidified by CD_3COOD to pH 4.5.

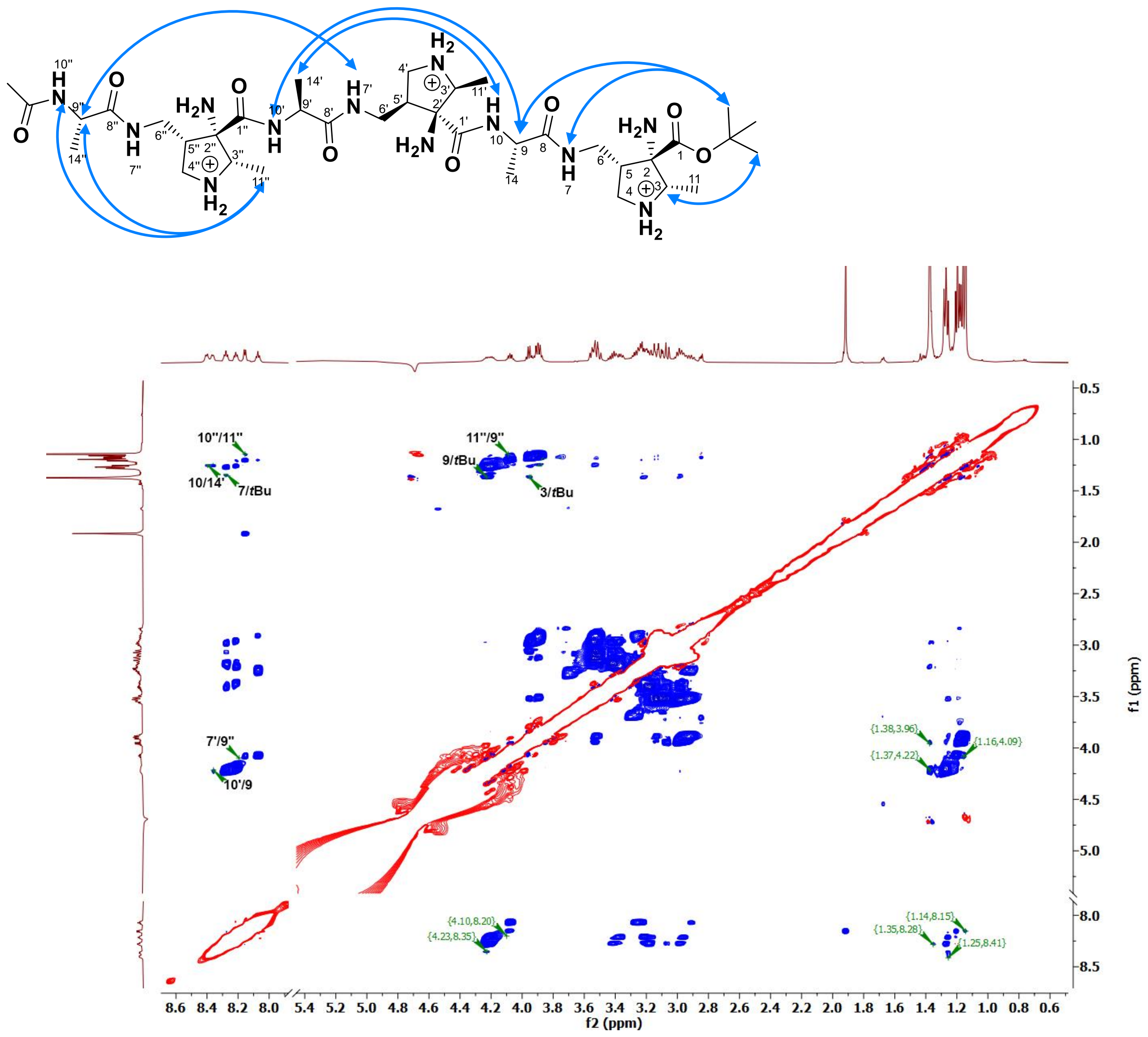

Figure S35. 2D ROESY spectrum of a 25mM H_2O/D_2O 9:1 solution of Ac(Ala-(*S*,*S*,*S*,*S*)AAMP)$_3$O*t*Bu (**Ac-6AS^{H}A**) acidified by CD_3COOD to pH 4.5.

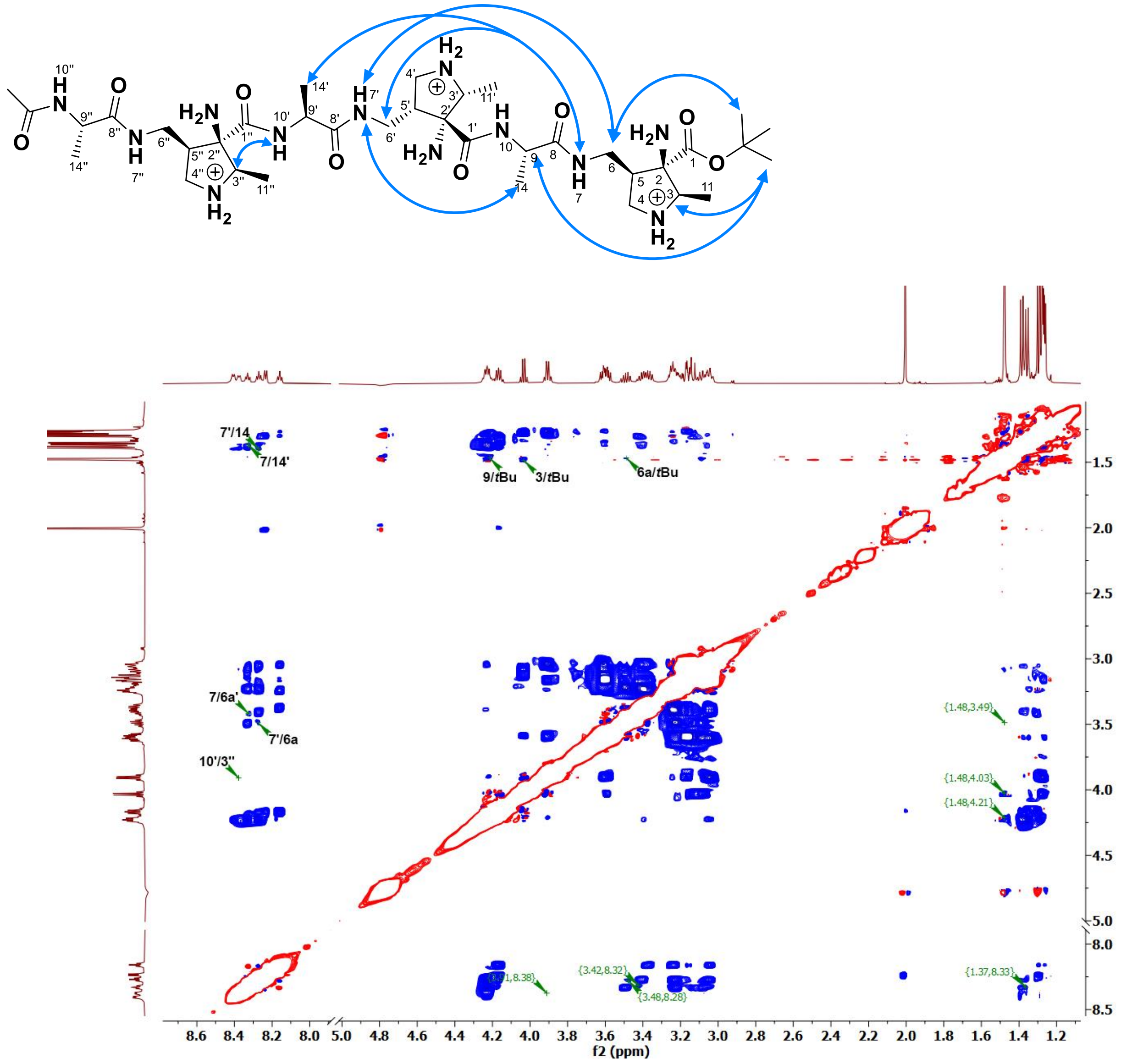

Figure S36. 2D ROESY spectrum of a 25mM H_2O/D_2O 9:1 solution of Ac(Ala-(*R*,*R*,*R*,*R*)AAMP)$_3$O*t*Bu (**Ac-6AR^{H}A**) acidified by CD_3COOD to pH 4.5.

4) ECD and IR/VCD spectra

ECD spectroscopy: ECD experiments were carried out on a J-1500 spectropolarimeter (Jasco, Tsukuba, Japan). The spectra were collected from 180 to 280 nm at room temperature in 0.01 cm cylindrical quartz cells at 1 mM concentration in H_2O, TFE or MeOH using the following setup: 5 nm/min speed, 16 s time constant, 1 nm spectral bandwidth, 2 scans, 0.5 nm steps. After baseline subtraction, the final data were expressed as molar ellipticities θ ($deg \cdot cm^2 \cdot dmol^{-1}$) per residue.

IR spectroscopy: Solutions in MeOH and $CHCl_3$ were measured on a Nicolet 6700 spectrometer (Thermo Fisher Scientific, Waltham, MA, USA) using a standard mid-IR source, KBr beamsplitter and DTGS detector (2 cm^{-1} spectral resolution, Happ–Genzel apodization function, 124 scans) in the 4000–1000 cm^{-1} spectral range. The cell compartment was purged by dry nitrogen during all measurements. A CaF_2 cell with 0.025 mm path length (SpeCac) was used for samples dissolved in MeOH and a NaCl cell with 0.1 mm path length (SpeCac) was used for samples dissolved in $CHCl_3$. For both methods solvent scans were subtracted as background. The baseline was corrected using a linear function. Final IR spectra were normalized to amide I intensity maxima.

VCD spectroscopy: VCD spectra were recorded on a commercial dual source VCD spectrometer (Chiral*IR-2X*™, BioTools, Inc., U.S.A.) working in a dual PEM mode using two ZnSe photoelastic modulators (36.996 and 37.02 kHz, Hinds Instruments, Inc., U.S.A.). The VCD data were collected for ~12 hours (12 blocks of 6000 scans each at 8 cm^{-1} resolution) at room temperature. A CaF_2 cell with 0.025 mm path length (SpeCac) was used for samples dissolved in MeOH and a NaCl cell with 0.1 mm path length (SpeCac) was used for samples dissolved in $CHCl_3$. For both methods solvent scans were subtracted as background. The baseline was corrected using a linear function. Final IR spectra were normalized to amide I intensity maxima. The VCD spectra were smoothened with a second-order Savitzky-Golay filter using a 9 point window and normalized to amide I maxima in the corresponding IR spectra. Numerical data treatment was carried out using the Grams/AI software (Thermo Electron, Waltham, MA, USA).

Foldamers **Ac-xGR^{H}A** (x = 2,4,6) and **Boc-8GR^{H}A** dissolved in MeOH were studied using ECD spectroscopy (Figure S37). The ECD spectrum of foldamer **Ac-2GR^{H}A** was characterized by two negative spectral bands below ~190 nm (Θ ~ 12500 degcm^{2}dmol^{-1}) and ~222 nm (Θ ~ 4000 degcm^{2}dmol^{-1}). For foldamer **Ac-4GR^{H}A**, the spectrum showed an ECD broad negative spectral band at ~194 nm slightly higher intensity (Θ ~ 15000 degcm^{2}dmol^{-1}) accompanied by a positive spectral band at ~222 nm of rather low intensity (Θ ~ 1500 degcm^{2}dmol^{-1}). Hexamer **Ac-6GR^{H}A** and octamer **Boc-8GR^{H}A** are, in contrast, characterized by a large negative maximum at ~203 nm for **Ac-6GR^{H}A** and at ~198 nm for **Boc-8GR^{H}A** with intensity about two times higher compared to the shorter foldamer (Θ ~ 29000 degcm^{2}dmol^{-1}) accompanied by a negative shoulder at ~222 nm. The increase of intensity observed for **Ac-6GR^{H}A** and **Boc-8GR^{H}A** could be interpreted in terms of forming of self-organized secondary structure.

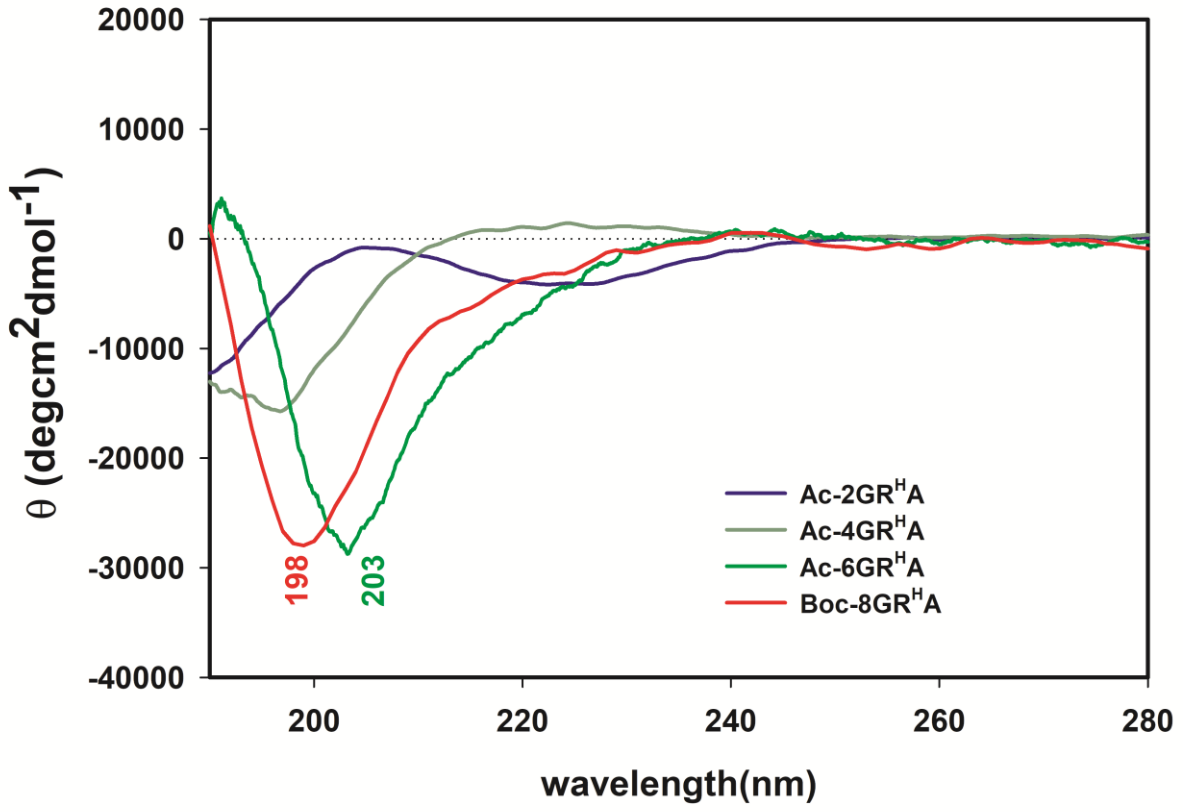

Figure S37: ECD spectra of the **GR^{H}A** foldamer series in MeOH. **Ac-2GR^{H}A** (blue), **Ac-4GR^{H}A** (dark green), **Ac-6GR^{H}A** (green), and **Boc-8GR^{H}A** (red).

Foldamers **Ac-xGR^{P}A** (x = 2,4,6) and **Boc-8GR^{P}A** including **NH_2-R^{P}A** were examined in $CHCl_3$ solution using VCD/IR spectroscopy (Figure S38), allowing evaluation of the spectral contribution of **NH_2-R^{P}A** unit to the spectral pattern of foldamers VCD/IR spectra (black line). The increase of foldamer's length and forming of self-organized secondary structure could be followed using spectral changes mainly in the amide I spectral region (~1640-1690 cm^{-1}). In contrast to the VCD spectra of foldamers **Ac-xGR^{P}A** (x = 2,4,6) and **Boc-8GR^{P}A**, only small spectral changes are observed in their IR spectra. For **Ac-2GR^{P}A**, a positive spectral band of low intensity at ~1661 cm^{-1} was observed. The general spectral pattern observed for foldamers **Ac-4GR^{P}A**, **Ac-6GR^{P}A**, and **Boc-8GR^{P}A** in the amide I spectral region is a positive couplet, whereas subtle spectral differences for foldamers **Ac-4GR^{P}A**, **Ac-6GR^{P}A**, and **Boc-8GR^{P}A** reflect formation of self-organized secondary structure (1680 cm^{-1} (-)/1656 cm^{-1} (+) for **Ac-4GR^{P}A**, (1679 cm^{-1} (-)/1647 cm^{-1} (+) for **Ac-6GR^{P}A**, (1673 cm^{-1} (-)/1642 cm^{-1} (+) for **Ac-8GR^{P}A**). The conservative character of the couplet observed for foldamers **Ac-6GR^{P}A** and **Boc-8GR^{P}A** might hint at the formation of a right-handed helical structure**.**

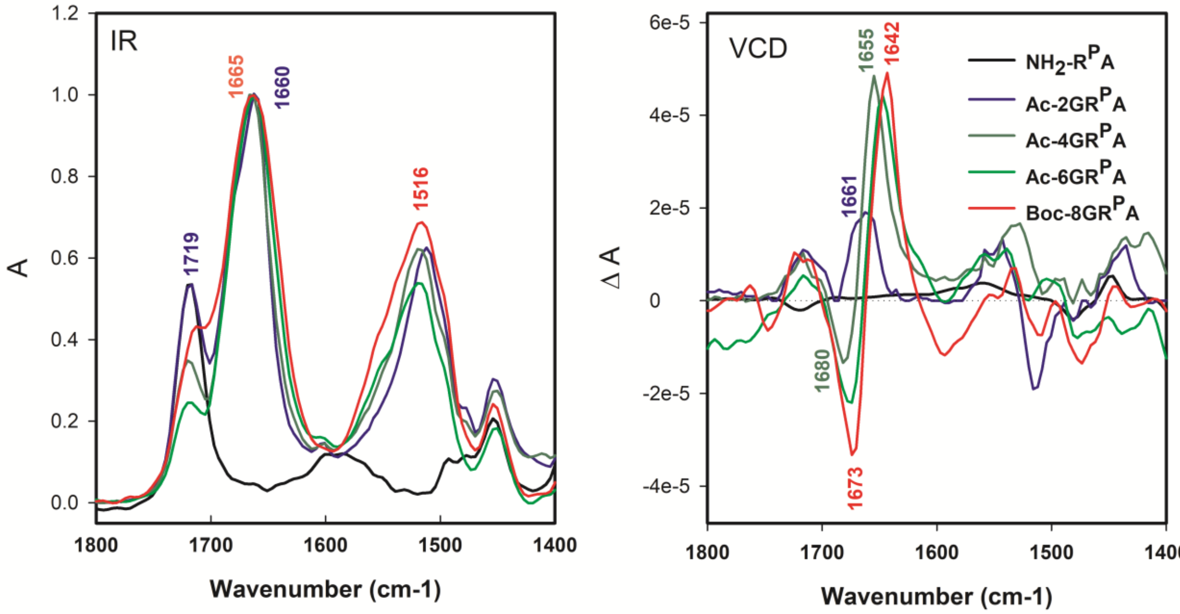

Figure S38: IR/VCD spectra of the **GR^{P}A** foldamer series in chloroform. **NH_2-R^{P}A** (black), **Ac-2GR^{P}A** (blue), **Ac-4GR^{P}A** (olive), **Ac-6GR^{P}A** (green) and **Boc-8GR^{P}A** (red).

Table S1: Summary of all IR/VCD values

Compound	IR<br>Amide I (cm^{-1})	IR<br>Amide II (cm^{-1})	VCD<br>Amide I (cm^{-1})
**Ac-2GR^{P}A**	1662	1512	1661(+)
**Ac-2GR^{H}A**	1660	1552	1646(-)
**Ac-4GR^{P}A**	1664	1519	1680(-) 1655(+)
**Ac-4GR^{H}A**	1659	1549	1659(-) 1637(+)
**Ac-6GR^{P}A**	1662	1519	1674(–) 1649(+)
**Ac-6GS^{P}A**	1663	1520	1673(+) 1648(–)
**Ac-6AS^{P}A**	1658	1514	1668(+) 1642(–)
**Ac-6AR^{P}A**	1657	1513	1689(+)[a] 1658(–) 1640(+)[a]
**Ac-6GR^{H}A**	1657	1548	1662(–) 1636(+)
**Ac-6AS^{H}A**	1653	1550, 1522	1672(+) 1640(–)
**Ac-6AR^{H}A**	1656	1554, 1520	1683(+)[a] 1655(–) 1609(+)[a]
**Boc-8GR^{P}A**	1665	1516	1672(–) 1643(+)
**Boc-8AS^{P}A**	1652	1516	1668(+) 1638(–)
**Boc-8GR^{H}A**	1652	1540	1660(–) 1635(+)
**Boc-8AS^{H}A**	1652	1550	1665(+) 1638(–)

[a] low-intensity bands

IR spectra of compounds **Ac-6GR^{P}A**, **Ac-6GS^{P}A**, **Ac-6AS^{P}A**, and **Boc-8GR^{P}A, Boc-8AS^{P}A** displayed two strong bands at ~3320 cm^{-1} and ~3370 cm^{-1}, implying that the peptides feature similarly hydrogen-bonded N-H groups. The shoulder at ~3450 cm^{-1} was assigned to the NH_2 functional group. In contrast, the bands of **Ac-6AR^{P}A** are more structured with a variable intensity suggesting different degree of N-H hydrogen bonding.

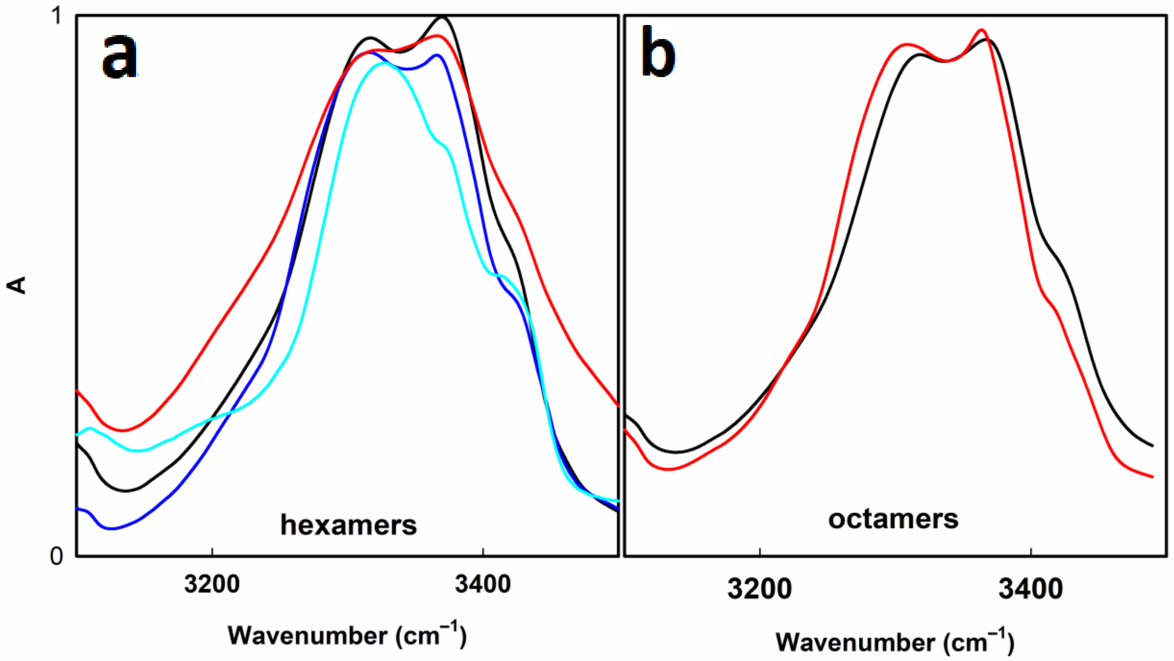

Figure S39. NH Region of the IR spectrum of oligopeptides in chloroform. **a** hexamers **Ac-6GR^{P}A** (black), **Ac-6AS^{P}A** (blue), **Ac-6AR^{P}A** (cyan), **Ac-6GS^{P}A** (red); **b** octamers **Boc-8GR^{P}A** (black), **Boc-8AS^{P}A** (blue).

The stability of **Ac-6GSDab** in MeOH solution is limited, even when using three times lower concentrations compared to the VCD/IR measurements of previous samples. The IR spectrum measured just after the sample dissolution exhibited an amide I band at ~1661 cm^{-1} and an amide II band at ~1550 cm^{-1}. In the amide I spectral region, a shoulder at ~1636 cm^{-1} emerged after 12 hours of measurement. Subsequently, an intensity increase of this shoulder accompanied by a shift of the amide I band to 1670 cm^{-1} and an intensity increase of the amide II band occurred. The observed spectral changes indicate the formation of β-turn/β-sheet structures.

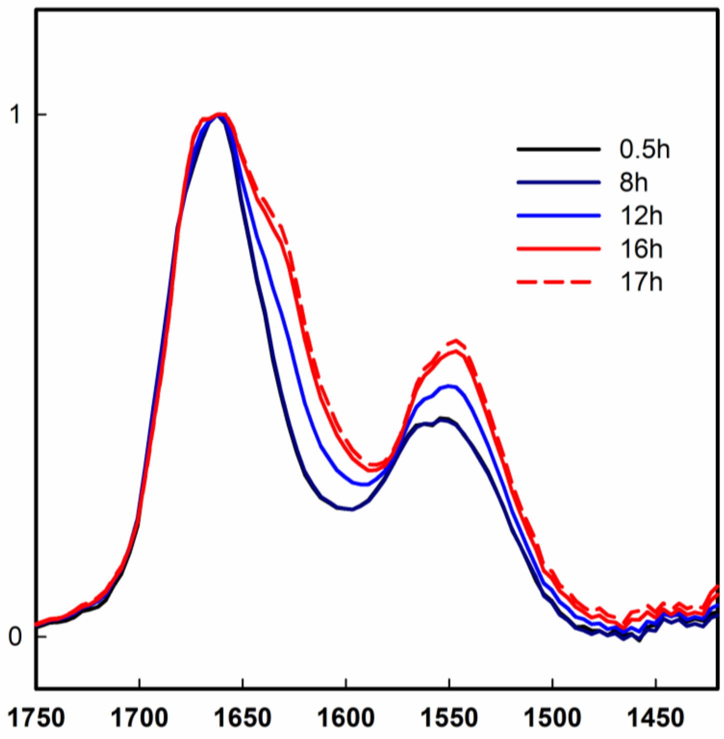

Figure S40: IR spectra of **Ac-6GSDab** in MeOH collected after 0.5 h (black), 8 h (dark blue) 12 h (blue), 16 h (red), 17 h (dashed red) after the sample dissolution (sample concentration 15 mM).

The time scale of 12 h, when the sample probably does not change largely its structure, did not allow to obtain reasonable VCD spectra. However, an enormous intensity increase of the VCD signal within the following 16 hours of measurement hints to the formation of chiral sample aggregates.[2] The comparison of compounds **Ac-6GR^{H}A** and **Ac-6GSDab** shows that the AAMP units seem to be not only important for secondary structure formation but also for the overall sample stability in solution.

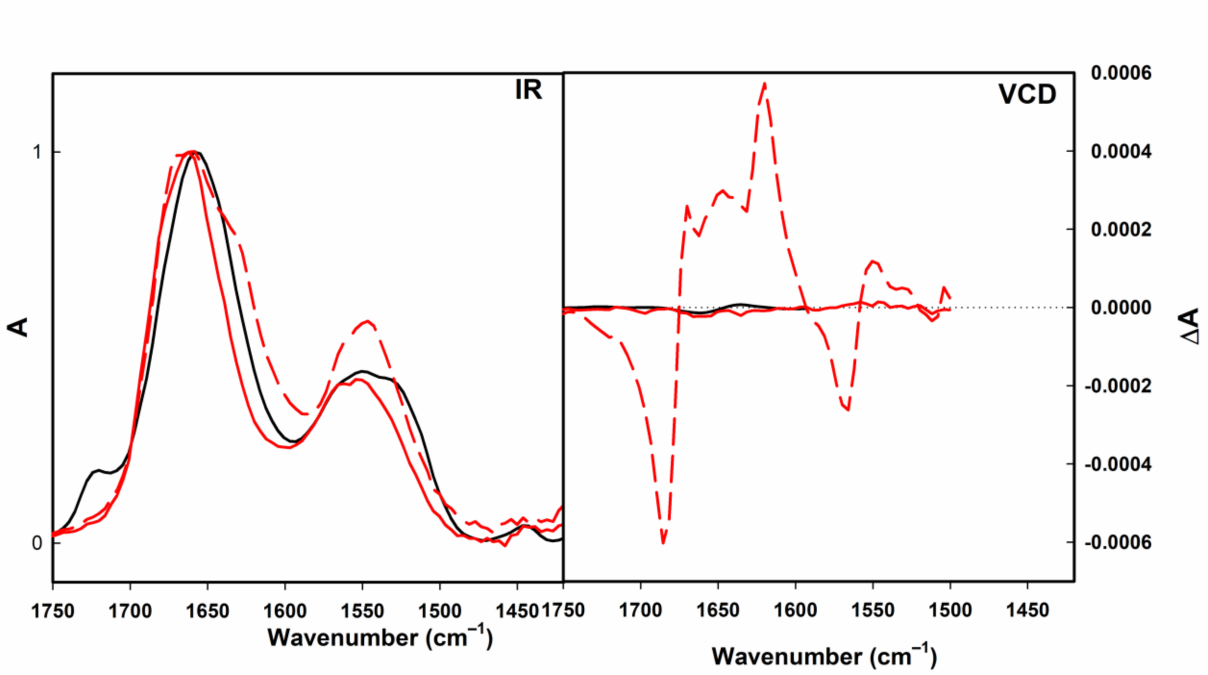

Figure S41: IR and VCD spectra of **Ac-6GR^{H}A** in MeOH (sample concentration 50 mM) (black) and **Ac-6GSDab** in MeOH (sample concentration 15 mM) collected after 0.5 h (red), and 16 h (dashed red) after dissolution.

5) Molecular dynamic and calculations

General

All simulations were performed using Gromacs 2016[3] and Gaussian 2016[4] program packages. Molecular dynamics simulations were performed in an NpT ensemble where a single solute molecule was placed in a 5x5x5 nm box, and the remainder of the volume was filled with solvent molecules. In the case of hexamer **Ac-6GR^{H}A** in water, which is sixfold positively charged moiety, neutralized the box with 6 acetate molecules. We used CHARMM-GUI to generate force field parameters for the hexamers/octamer/acetate and TIP3P force field parameters to describe water molecules.[5] The long-range electrostatics were modeled using PME with a 1 nm cutoff, after which the interactions were shifted to zero. The Van der Waals interactions were also treated with 1 nm cutoff, and long-range dispersion correction for energy and pressure was applied. All hydrogen-containing bonds were constrained using LINCS.[6] A V-rescale thermostat[7] and Berendsen barostat[8] were used to keep the temperature and pressure at ambient conditions (300K and 1 bar) using a time constant 1 and 5 ps^{-1}, respectively. The MD simulation of **Ac-6GR^{H}A** at 8000 K for enhanced sampling was performed in an NVT ensemble, where only the solute molecule was tempered at 8000 K, while the solvent was kept at 300 K, using a thermostat time constant of 0.001 ps^{-1}. All stereochemical centers were restrained during the high-temperature simulation, and the resulting structures were then relaxed in a 10 ps MD simulation. Low temperature (300 K) simulations used 2 fs time step while the high temperature used 0.1 fs time steps.

Used models for comparison to experimental data:

Fmoc(Gly-(*R*,*R*,*R*,*R*)^P^AAMP)₃O*t*Bu (6R^P^AG-Fmoc) in CDCl₃

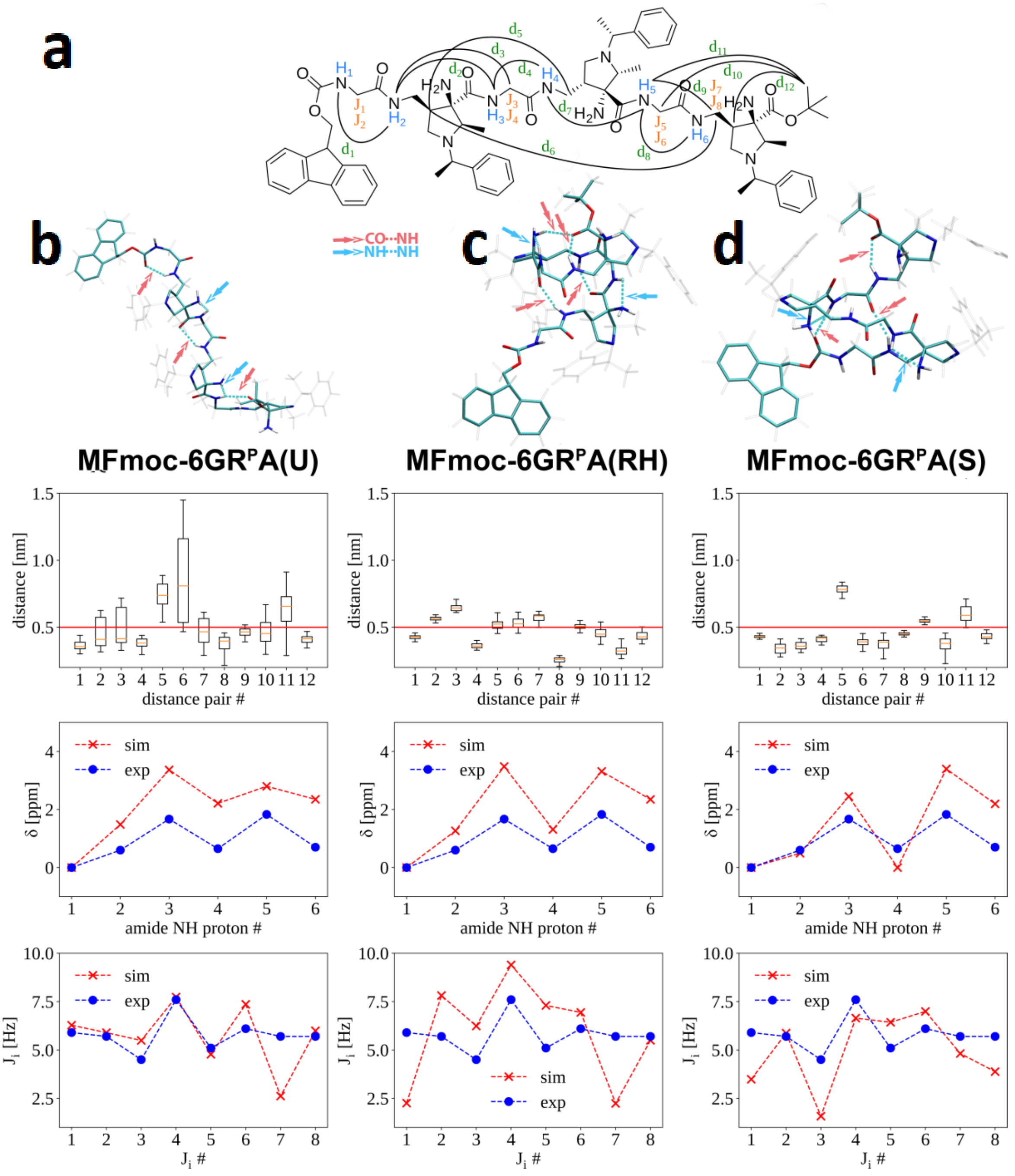

Figure S42: Representative structures of **Fmoc-6GR^P^A** and comparative analysis of experimental and simulated data. **a** Experimental long-range NOEs d_i observed amide proton chemical shifts δ_i, and spin-spin couplings J_i of **Fmoc-6GR^P^A** used for comparing to simulation data. **b** Representative model of unrestrained structure **MFmoc-6GR^P^A**(U). Representative models of **c** right-handed helical structure **MFmoc-6GR^P^A**(RH) and **d** left-handed staircase-like structure **d MFmoc-6GR^P^A**(S) resulting from unrestrained MD simulation. Comparison of simulated NOEs, amide proton chemical shifts, and

spin-spin coupling constants (top, middle, and bottom, respectively) with comparison to experimental observables. In the structures hydrogen bonds for these representative conformers are shown in dashed cyan lines. Blue (NH-NH) and red (NH-CO) arrows indicate the nature of the hydrogen bond. The red line in NOEs represents the 0.5 nm observable threshold.

Three models (**MFmoc-6GR^{P}A(U)**/ **MFmoc-6GR^{P}A(RH)**/ **MFmoc-6GR^{P}A(S)**) were used to compare to experimental data (Figure S42a). Model **MFmoc-6GR^{P}A(U)** was obtained by performing a 10000 ns (U)nrestrained MD simulation from which 100 structures were extracted using a uniform sampling rate (every 100 ns) (Figure S42b). The average model **MFmoc-6GR^{P}A**(U) provides relatively good agreement with observed amide proton chemical shifts and spin-spin coupling constants; however, the observed NOEs show significant deviations from the distance threshold of 0.5 nm, especially the pyrrolidine-pyrrolidine contacts at d5 and d6 deviate considerably.

Then we visually scanned this initial unrestrained MD simulation for potentially stable structural motifs representative for left-handed and right-handed helical structures. Two prominent structures, one for each helix chirality, were selected. Both structures were found to be present in unrestrained simulation for a significant amount of time, i.e., 15% (right) and 29% (left) of simulation time based on RMSD measurement (backbone atoms) using 0.3 nm cutoff for differentiation. The two structures were subsequently used as a starting point for a 300 ps unrestrained simulation for environment relaxation with 10 ps sampling time, yielding 30 representative structures per model. Model **MFmoc-6GR^{P}A(RH)** (Figure S42c) corresponds to the right-handed helix like-structure, while model **MFmoc-6GR^{P}A(S)** (Figure S42d) forms a left-handed staircase-like structure. These two models are further discussed in the main text.

Boc(Gly-(*R*,*R*,*R*,*R*)^{P}AAMP)$_3$O*t*Bu (Boc-8GR^{P}A) in CDCl$_3$

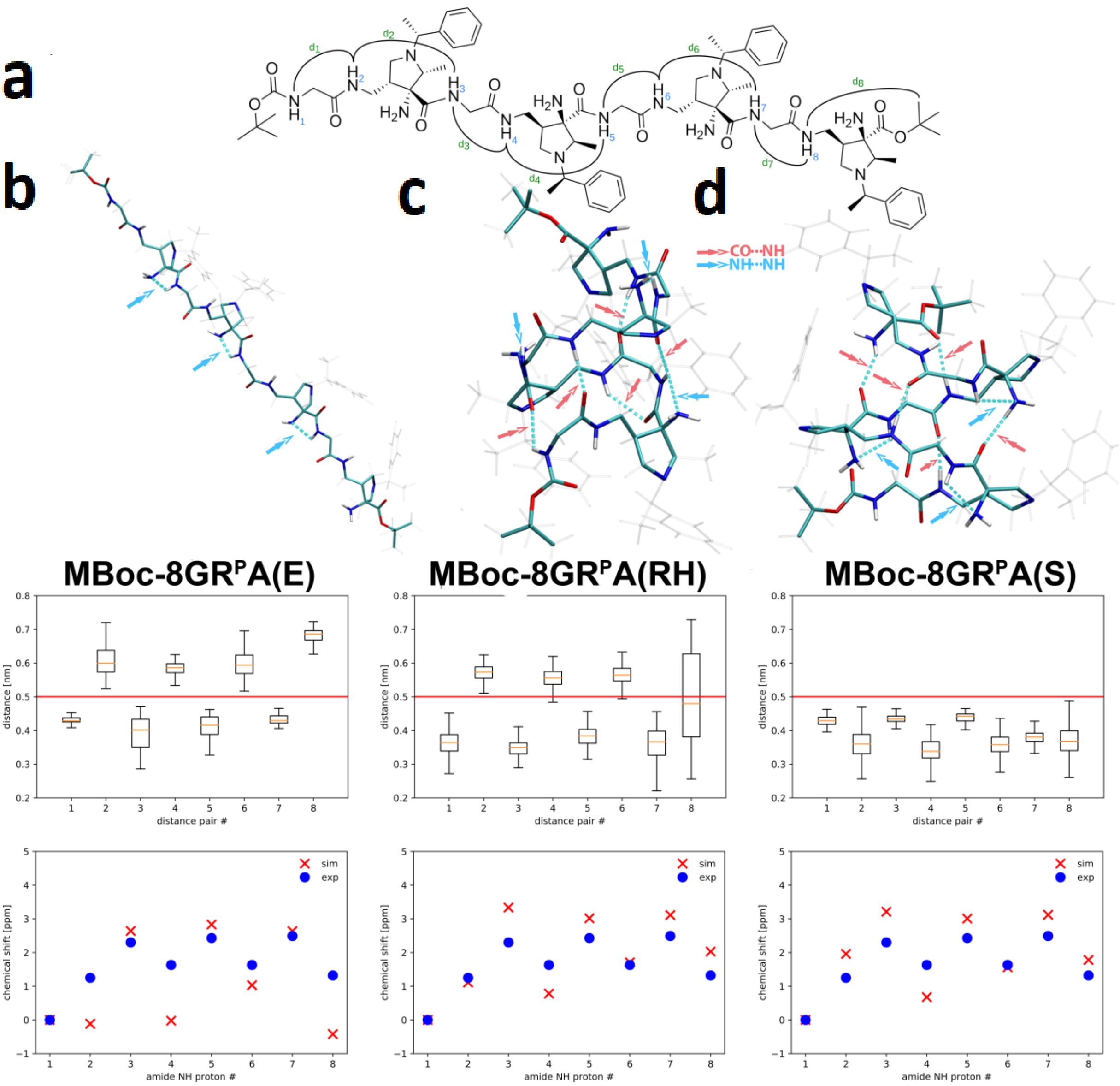

Figure S43. Representative structures of **Boc-8GR^{P}A** and comparative analysis of experimental and simulated data. **a** Experimental long-range NOEs d_i (*cf.* Figure 5D) and amide proton chemical shifts δ_i of **Boc-8GR^{P}A** used for comparing simulation data. **b** Extended structure **MBoc-8GR^{P}A**(E). **c** Right-handed helical structure **MBoc-8GR^{P}A**(RH) **d** Left-handed staircase-like structure **MBoc-8GR^{P}A**(S). Comparison of simulated to experimental NOEs and NH amide proton chemical shifts (top and bottom), respectively. In the structures hydrogen bonds for these representative conformers are shown in dashed cyan lines. Blue (NH-NH) and red (NH-CO) arrows indicate the nature of the hydrogen bond. The red line in NOEs represents the 0.5 nm observable threshold.

Three models (**MBoc-8GR^{P}A(E)/MBoc-8GR^{P}A(RH)/MBoc-8GR^{P}A(S)**) were used to compare to experimental data (Figure S43a). Model **MBoc-8GR^{P}A(E)** was obtained by performing a 2 ns

simulation (36 representative structures) while restraining the **Boc-8GR^{P}A** molecule in (E)xtended position using lower walls restrain on end atoms (4.5 nm) as developed in Plumed[9] (Figure S43b). For this model, half of the simulated NOE distances are above the observable limits (> 0.5 nm), and the N2-H, N4-H, and N8-H amide proton chemical shifts are too small compared to the experimental data indicating the formation of a secondary structure. Models **MBoc-8GR^{P}A(RH)** (Figure S38c) and **MBoc-8GR^{P}A(S)** (Figure S43d) were obtained by performing an unbiased 4500 ns MD simulation (yielding ~115000 structures) and scanning the obtained structures for any structural motifs similar to those studied for **MFmoc-6GR^{P}A**. We found two structural motifs that are very similar to **MFmoc-6GR^{P}A(RH)** and **MFmoc-6GR^{P}A(S)** models. These structures were found present for 51% (**MFmoc-6GR^{P}A(RH)**) and 4% (**MFmoc-6GR^{P}A(S)**) of simulation time based on RMSD measurement (backbone atoms) using 0.3 nm cutoff for differentiation. We selected these two structures (**MBoc-8GR^{P}A(RH)** – right-handed helix-like structure; **MBoc-8GR^{P}A(S)** – left-handed staircase-like structure) and used them as a starting point for a 300 ps simulation with 10 ps sampling time, yielding 30 representative structures per model.

AcNH(Gly-(*R*,*R*,*R*)AAMP)$_3$O*t*Bu (6R^{H}AG-Ac) in water

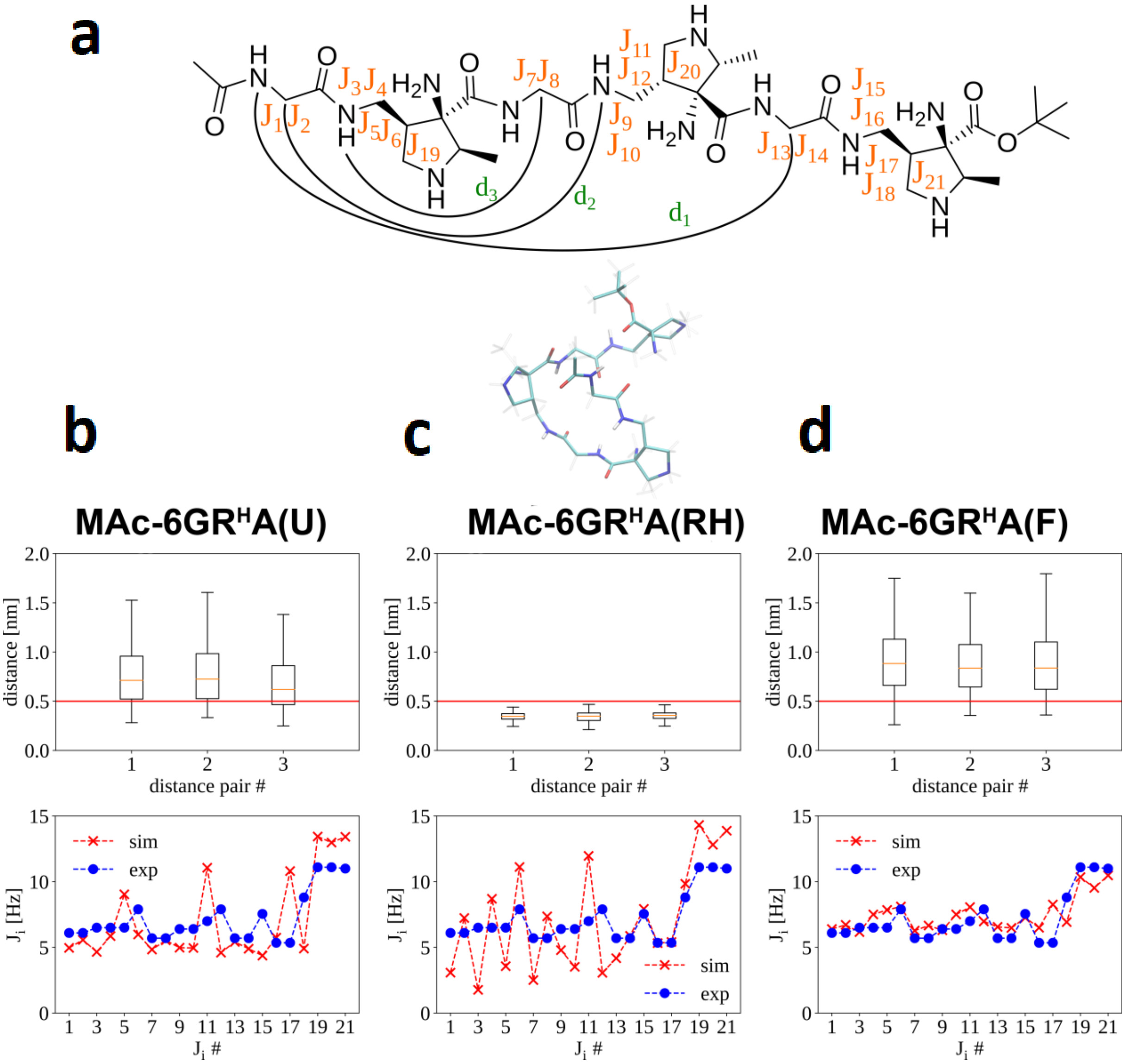

Figure S44. Representative structures of **Ac-6GR^{H}A** and comparative analysis of experimental and simulated data. **a** Experimental long-range NOEs d_i and spin-spin couplings J_i obtained of **Ac-6GR^{H}A** used for comparing to simulation data; Simulated and experimental NOE (top) and NH amide proton chemical shift (bottom) data for **b** Unrestrained model **MAc-6GR^{H}A**(U); **c** Folded model **MAc-6GR^{H}A**(RH) obtained after selecting simulated structures fulfilling all experimental NOEs, i.e., distance < 0.5 nm; **d** Data for unstructured foldamer **MAc-6GR^{H}A**(F) by artificially imposing 8000 K on the foldamer while keeping the solvent water at 300 K (enhanced sampling). The red line in NOEs represents the 0.5 nm observable threshold.

Three models (**MAc-6GR^{H}A(U)/MAc-6GR^{H}A(RH)/MAc-6GR^{H}A(F)**) were used to compare to the experimental data in aqueous solution (Figure S44a). At first, model **MAc-6GR^{H}A(U)** was obtained by performing a 5500 ns (U)nrestricted MD simulation from which 110 structures were extracted using a

uniform sampling rate (every 50 ns) (Figure S44b). Its NOE distances are above the observable threshold limit of 0.5 nm and the coupling constants scatter considerably. The second model **MAc-6GR^{H}A(RH)** was obtained by filtering all obtained structures from the unrestricted MD simulation (55000 structures) using experimental NOE distance restraints (0.5 nm cutoff), obtaining 103 structures (Figure S44c). The NOE data understandably correspond well to the experimental data, but the values of the coupling constants considerably deviate from the experimental values. The main but not only reason may be the full protonation state of the simulation model used. However, NMR spectral investigations at different pH suggest only partial protonation due to changes with pH (Supplementary Figure S27). To further test the effect of foldamer flexibility, an MD simulation in aqueous solutions using a large nonphysical temperature for the solute was performed. This model achieves a fully conformationally flexible state. This model **MAc-6GR^{H}A(F)** intends to enhance the sampling by forcing the foldamer to an non-physical 8000 K simulation while the solvent was kept at 300K for 200 ps, yielding 200 structures (Figure S44d). The model possesses all NOEs above the 0.5 nm threshold, but interestingly, the coupling constants fit well to the experimental results. These data suggest that folding in **Ac-6GR^{H}A** occurs, but the overall structure is more flexible in aqueous solution and several conformations are likely populated.

Calculation of ^{1}H NMR shifts of hexamer/octamer in $CDCl_3$

Structures obtained from MD simulations were stripped of all solvent molecules, and the solvent was further modeled by means of continuum solvation.[10] The structures were then optimized using 10 optimization steps at the B3LYP/6-311++G** level of theory. Subsequently, the ^{1}H isotropic nuclear magnetic shielding constants were calculated using the LC-BLYP/pcS2[11] level of theory using the CSGT method. The values obtained from all representative structures were averaged and transformed to ^{1}H chemical shifts by referencing the values to the NH_1 amide proton.

Calculation of J_i spin-spin couplings in water

Structures obtained from MD simulations were stripped of all solvent molecules and the solvent was further modeled by means of continuum solvation.[10] The molecular geometry was not further optimized at the QM level, and the spin-spin coupling constants was directly calculated. The spin-spin coupling constants were calculated using mPW1PW91/pc-J2[12] level of theory, and only Fermi contact spin-spin terms were included.

6) X-Ray crystallographic data

Diffraction data sets of **Fmoc-2GR^{P}A** were collected on a Bruker D8 VENTURE Kappa Duo diffractometer with a PHOTON100 detector with micro-focus sealed tube CuKα (λ = 1.54178 Å) x-ray source IμS at 130K. Diffraction data sets of **NH_2-4GR^{P}A** were collected on a Bruker D8 VENTURE Kappa Duo diffractometer with a PHOTONIII detector with micro-focus sealed tube CuKα (λ = 1.54178 Å) x-ray source IμS at 120K. The structures were solved by direct methods (XT[13a]) and refined by full matrix least squares based on F^2 (SHELXL2018[13b]). The hydrogen atoms on carbon were calculated into idealized positions and fixed during refinement (riding model) with assigned temperature factors either $H_{iso}(H) = 1.2\ U_{eq}$(pivot atom) or $H_{iso}(H) = 1.5\ U_{eq}$ (pivot atom) for methyl groups. Hydrogen atoms on oxygen were found on the difference Fourier map and refined under rigid body assumption. The determination of the absolute configuration of both crystals is based on known chirality of the carbon atoms preserved during synthesis. One of the phenylethyl moieties of **NH_2-4GR^{P}A** is disordered over two positions triggering disorder also in one of the water molecules. The crystallographic data are summarized in Table S2.

Table S2: Crystal data, data collection and refinement parameters for **Fmoc-2GR^{P}A** and **NH_2-4GR^{P}A**.

**Compound**	**Fmoc-2GR^{P}A**	**NH_2-4GR^{P}A**
CCDC	2142444	2142445
Formula	$C_{36}H_{44}N_4O_5$	$C_{38}H_{58}N_8O_5 \cdot 2.343\ H_2O$
M.w.	612.75	749.13
Temperature [K]	130	120
Crystal system	Orthorhombic	Monoclinic
Space group [No.]	$P2_12_12_1$ (No. 19)	$P2_1$ (No 4)
a [Å]	9.8349 (6)	16.1650 (7)
b [Å]	36.465 (2)	6.5283 (3)
c [Å]	9.2883 (5)	20.0911 (10)
β [°]		102.817 (3)
Z	4	2
V [Å^3]	3331.0 (3)	2067.38 (17)
D_x [g cm^{-3}]	1.222	1.203
Crystal size [mm]	0.49 × 0.21 × 0.13	0.27 × 0.08 × 0.04
Crystal shape	Prism	Prism
μ [mm^{-1}]	0.66	0.68
ϑ_{max} [°]	63.8	63.0
Measured reflections	19223	21703
Independent diffractions (R_{int} ^{a})	5466 (0.046)	6802 (0.057)
Observed diffract. [I>2σ(I)]	5237	5310
T_{min}, T_{max}	0.72, 0.92	0.84, 0.97
No. of parameters	411	490
w_1, w_2 ^{b}	0.0331, 4.7048	0.0832, 0
Absolute structure param. (Flack)	-0.14 (15)	0.1 (2)
R^c [$F^2 > 2\sigma(F^2)$]	0.064	0.051
$wR(F^2)$ for all data	0.155	0.142
GOF ^{d}	1.16	1.04
Residual electron density [e/Å^3]	0.52, -0.28	0.31, -0.20

^{a} $R_{int} = \sum|F_o^2 - F_{o,mean}^2| / \sum F_0^2$, ^{b} $R(F) = \sum||F_o| - |F_c|| / \sum|F_o|$, ^{c} $wR(F^2) = [\sum(w(F_o^2 - F_c^2)^2)/(\sum(F_o^2)^2)]^{1/2}$, weighting scheme: $w = [\sigma^2(F_o^2) + (w_1P)^2 + w_2P)^2]^{-1}$, where $P = [\max(F_o^2, 0) + 2F_c^2]^{1/3}$, ^{d} $S = [\sum(w(F_o^2 - F_c^2)^2)/N_{diffrs} - N_{params})]^{1/2}$.

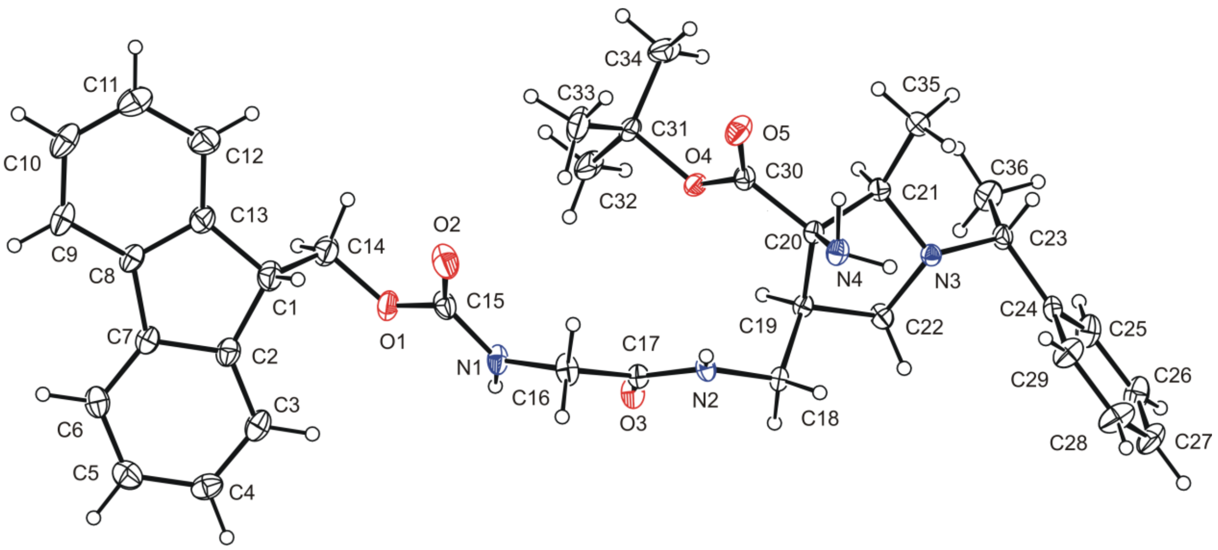

Figure S45. View on the molecule of **Fmoc-2GR^{P}A**. The displacement elipsoids are drawn at 30% probability level.

Table S3: Torsion angles values (°) for **Fmoc-2GR^{P}A.**

C14—C1—C2—C3	58.1 (8)	N2—C18—C19—C22	179.5 (4)
C13—C1—C2—C3	179.1 (6)	C18—C19—C20—N4	29.2 (5)
C14—C1—C2—C7	-122.0 (5)	C22—C19—C20—N4	-94.7 (4)
C13—C1—C2—C7	-1.1 (6)	C18—C19—C20—C30	-94.0 (5)
C7—C2—C3—C4	-1.2 (8)	C22—C19—C20—C30	142.1 (4)
C1—C2—C3—C4	178.7 (6)	C18—C19—C20—C21	147.9 (4)
C2—C3—C4—C5	0.5 (9)	C22—C19—C20—C21	23.9 (4)
C3—C4—C5—C6	0.1 (9)	C22—N3—C21—C35	171.4 (4)
C4—C5—C6—C7	0.0 (9)	C23—N3—C21—C35	-56.8 (5)
C5—C6—C7—C2	-0.6 (8)	C22—N3—C21—C20	48.4 (4)
C5—C6—C7—C8	-178.0 (5)	C23—N3—C21—C20	-179.9 (4)
C3—C2—C7—C6	1.2 (8)	N4—C20—C21—N3	75.2 (4)
C1—C2—C7—C6	-178.7 (5)	C30—C20—C21—N3	-167.7 (4)
C3—C2—C7—C8	179.1 (5)	C19—C20—C21—N3	-43.4 (4)
C1—C2—C7—C8	-0.7 (6)	N4—C20—C21—C35	-46.8 (6)
C6—C7—C8—C13	-179.9 (6)	C30—C20—C21—C35	70.3 (5)
C2—C7—C8—C13	2.5 (6)	C19—C20—C21—C35	-165.5 (4)
C6—C7—C8—C9	3.9 (10)	C21—N3—C22—C19	-33.2 (5)
C2—C7—C8—C9	-173.8 (6)	C23—N3—C22—C19	-164.6 (4)

Table S3 (continued).

C13—C8—C9—C10	0.7 (8)	C18—C19—C22—N3	-122.0 (4)
C7—C8—C9—C10	176.6 (5)	C20—C19—C22—N3	4.1 (5)
C8—C9—C10—C11	2.8 (9)	C22—N3—C23—C24	-57.8 (5)
C9—C10—C11—C12	-4.3 (10)	C21—N3—C23—C24	175.9 (4)
C10—C11—C12—C13	2.2 (10)	C22—N3—C23—C36	70.9 (6)
C11—C12—C13—C8	1.4 (10)	C21—N3—C23—C36	-55.4 (6)
C11—C12—C13—C1	-174.3 (6)	N3—C23—C24—C25	134.7 (5)
C9—C8—C13—C12	-2.9 (9)	C36—C23—C24—C25	5.9 (7)
C7—C8—C13—C12	-179.6 (5)	N3—C23—C24—C29	-49.2 (6)
C9—C8—C13—C1	173.5 (5)	C36—C23—C24—C29	-178.0 (5)
C7—C8—C13—C1	-3.2 (7)	C29—C24—C25—C26	0.6 (9)
C14—C1—C13—C12	-58.1 (9)	C23—C24—C25—C26	176.7 (5)
C2—C1—C13—C12	178.6 (6)	C24—C25—C26—C27	0.5 (9)
C14—C1—C13—C8	125.9 (5)	C25—C26—C27—C28	-0.9 (10)
C2—C1—C13—C8	2.6 (6)	C26—C27—C28—C29	0.2 (10)
C15—O1—C14—C1	-75.6 (6)	C25—C24—C29—C28	-1.2 (9)
C2—C1—C14—O1	-62.4 (6)	C23—C24—C29—C28	-177.4 (6)
C13—C1—C14—O1	-177.9 (5)	C27—C28—C29—C24	0.8 (10)
C16—N1—C15—O2	-4.3 (9)	C31—O4—C30—O5	5.0 (7)
C16—N1—C15—O1	176.1 (4)	C31—O4—C30—C20	-170.2 (4)
C14—O1—C15—O2	-0.5 (8)	N4—C20—C30—O5	35.1 (6)
C14—O1—C15—N1	179.1 (4)	C19—C20—C30—O5	160.7 (5)
C15—N1—C16—C17	-132.6 (5)	C21—C20—C30—O5	-85.1 (6)
C18—N2—C17—O3	-0.6 (7)	N4—C20—C30—O4	-149.6 (4)
C18—N2—C17—C16	175.4 (4)	C19—C20—C30—O4	-24.0 (6)
N1—C16—C17—O3	-18.1 (7)	C21—C20—C30—O4	90.2 (5)
N1—C16—C17—N2	165.7 (4)	C30—O4—C31—C32	-175.4 (5)
C17—N2—C18—C19	82.5 (5)	C30—O4—C31—C33	-55.9 (6)
N2—C18—C19—C20	59.4 (5)	C30—O4—C31—C34	66.9 (6)

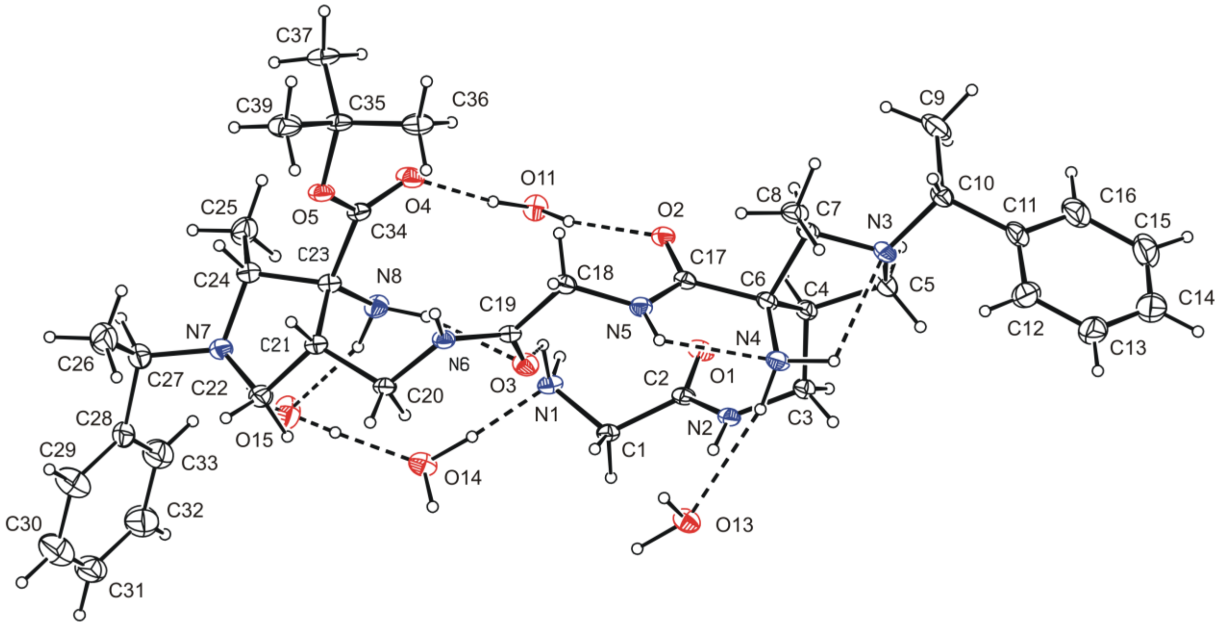

Figure S46. View on the molecule of **NH_2-4GR^{P}A**. The displacement ellipsoids are drawn on 20% probability level to enable clear atom numbering. Only one position of disordered phenylethyl group is displayed for clarity.

Table S4: Hydrogen-bond geometry for **NH_2-4GR^{P}A**.

D—H···*A*	*D*—H (Å)	H···*A* (Å)	*D*···*A* (Å)	*D*—H···*A* (°)
N1—H11*N*···O3	0.97	2.26	2.900 (5)	123
N4—H42*N*···O13*W*^{ii}	0.99	2.51	3.477 (5)	167
N4—H41*N*···N3	1.05	2.40	2.849 (5)	104
N5—H5···N4	0.88	2.22	2.660 (5)	111
N8—H82*N*···O3	0.98	2.30	3.260 (5)	164
N8—H81*N*···O15*W*	1.02	2.29	3.269 (11)	163
O11*W*—H12*W*···O2	0.94	1.91	2.797 (4)	156
O11*W*—H11*W*···O4	0.99	1.77	2.751 (4)	168
O14*W*—H22*W*···N1^{iii}	1.07	1.71	2.767 (5)	169

Symmetry codes: (i) *x*, *y*-1, *z*; (ii) *x*, *y*+1, *z*; (iii) -*x*+1, *y*-1/2, -*z*+1; (iv) -*x*+1, *y*+1/2, -*z*+1.

Table S5: Torsion angles values (°) for **NH_2-4GR^{P}A**.

C3—N2—C2—O1	-0.3 (6)	C27—N7—C22—C21	-131.8 (4)
C3—N2—C2—C1	177.9 (3)	C20—C21—C22—N7	-150.9 (4)
N1—C1—C2—O1	-33.8 (5)	C23—C21—C22—N7	-26.4 (5)
N1—C1—C2—N2	148.0 (3)	C20—C21—C23—N8	46.9 (5)
C2—N2—C3—C4	76.1 (5)	C22—C21—C23—N8	-74.7 (4)
N2—C3—C4—C5	174.6 (4)	C20—C21—C23—C34	-79.5 (4)
N2—C3—C4—C6	56.4 (5)	C22—C21—C23—C34	158.9 (4)
C7—N3—C5—C4	-21.9 (4)	C20—C21—C23—C24	163.4 (4)
C10—N3—C5—C4	-154.4 (4)	C22—C21—C23—C24	41.8 (4)
C3—C4—C5—N3	-133.4 (4)	C22—N7—C24—C25	149.8 (4)
C6—C4—C5—N3	-9.0 (4)	C27—N7—C24—C25	-78.9 (5)
C5—C4—C6—N4	-82.1 (4)	C22—N7—C24—C23	27.6 (5)
C3—C4—C6—N4	40.2 (5)	C27—N7—C24—C23	158.9 (4)
C5—C4—C6—C17	147.1 (4)	N8—C23—C24—N7	77.5 (4)
C3—C4—C6—C17	-90.6 (4)	C34—C23—C24—N7	-161.5 (4)
C5—C4—C6—C7	34.1 (4)	C21—C23—C24—N7	-42.5 (4)
C3—C4—C6—C7	156.4 (4)	N8—C23—C24—C25	-44.1 (5)
C10—N3—C7—C8	-60.6 (5)	C34—C23—C24—C25	77.0 (5)
C5—N3—C7—C8	167.2 (4)	C21—C23—C24—C25	-164.1 (4)
C10—N3—C7—C6	175.8 (4)	C22—N7—C27—C28B	-61.2 (10)
C5—N3—C7—C6	43.7 (4)	C24—N7—C27—C28B	170.6 (9)
N4—C6—C7—N3	74.9 (4)	C22—N7—C27—C26	58.7 (6)
C17—C6—C7—N3	-165.0 (3)	C24—N7—C27—C26	-69.4 (6)
C4—C6—C7—N3	-46.9 (4)	C22—N7—C27—C28A	-74.0 (6)
N4—C6—C7—C8	-47.6 (5)	C24—N7—C27—C28A	157.9 (5)
C17—C6—C7—C8	72.5 (4)	N7—C27—C28A—C29A	134.3 (5)
C4—C6—C7—C8	-169.4 (4)	C26—C27—C28A—C29A	4.5 (8)
C7—N3—C10—C11	163.5 (4)	N7—C27—C28A—C33A	-47.5 (7)
C5—N3—C10—C11	-68.7 (6)	C26—C27—C28A—C33A	-177.4 (5)
C7—N3—C10—C9	-65.4 (6)	C33A—C28A—C29A—C30A	0.0
C5—N3—C10—C9	62.3 (6)	C27—C28A—C29A—C30A	178.2 (7)
N3—C10—C11—C16	126.5 (5)	C28A—C29A—C30A—C31A	0.0
C9—C10—C11—C16	-3.3 (8)	C29A—C30A—C31A—C32A	0.0
N3—C10—C11—C12	-54.9 (6)	C30A—C31A—C32A—C33A	0.0
C9—C10—C11—C12	175.3 (5)	C31A—C32A—C33A—C28A	0.0

Table S5 (continued).

C16—C11—C12—C13	1.3 (8)	C29A—C28A—C33A—C32A	0.0
C10—C11—C12—C13	-177.4 (5)	C27—C28A—C33A—C32A	-178.2 (7)
C11—C12—C13—C14	-0.2 (9)	N7—C27—C28B—C29B	109.7 (11)
C12—C13—C14—C15	-2.0 (10)	C26—C27—C28B—C29B	-16.8 (12)
C13—C14—C15—C16	2.9 (10)	N7—C27—C28B—C33B	-63.8 (11)
C12—C11—C16—C15	-0.4 (8)	C26—C27—C28B—C33B	169.7 (8)
C10—C11—C16—C15	178.3 (5)	C33B—C28B—C29B—C30B	0.0
C14—C15—C16—C11	-1.7 (9)	C27—C28B—C29B—C30B	-173.0 (16)
C18—N5—C17—O2	-4.1 (6)	C28B—C29B—C30B—C31B	0.0
C18—N5—C17—C6	170.1 (3)	C29B—C30B—C31B—C32B	0.0
N4—C6—C17—O2	-171.6 (4)	C30B—C31B—C32B—C33B	0.0
C7—C6—C17—O2	70.0 (5)	C31B—C32B—C33B—C28B	0.0
C4—C6—C17—O2	-38.5 (5)	C29B—C28B—C33B—C32B	0.0
N4—C6—C17—N5	14.0 (5)	C27—C28B—C33B—C32B	174.2 (13)
C7—C6—C17—N5	-104.3 (4)	C35—O5—C34—O4	1.8 (6)
C4—C6—C17—N5	147.1 (4)	C35—O5—C34—C23	-178.0 (3)
C17—N5—C18—C19	94.0 (4)	N8—C23—C34—O4	5.4 (6)
C20—N6—C19—O3	4.2 (6)	C21—C23—C34—O4	134.1 (4)
C20—N6—C19—C18	-178.4 (3)	C24—C23—C34—O4	-115.4 (5)
N5—C18—C19—O3	-33.0 (6)	N8—C23—C34—O5	-174.7 (3)
N5—C18—C19—N6	149.5 (4)	C21—C23—C34—O5	-46.1 (5)
C19—N6—C20—C21	-116.2 (4)	C24—C23—C34—O5	64.4 (5)
N6—C20—C21—C22	172.7 (4)	C34—O5—C35—C38	-177.9 (4)
N6—C20—C21—C23	56.0 (5)	C34—O5—C35—C36	-60.0 (5)
C24—N7—C22—C21	-0.8 (5)	C34—O5—C35—C37	63.8 (5)

7) General experimental information

Synthesis: Reactions not involving aqueous conditions were performed in flame-dried glassware under an argon atmosphere. Solvents and additives were dried prior to use according to standard procedures. TLC analyses were performed on POLYGRAM SIL G/UV254 plates. Chromatographic separations were carried out on silica gel 60 (Fluka, 230-400 mesh) either manually or on a CombiFlash® NextGen 300+ instrument.

Mass spectroscopy: ESI mass spectra were obtained on Thermo Fisher Scientific LCQ Fleet spectrometer, sample concentration approx. 1 µg/mL, spray voltage pos. mode: 3.3 kV. HRMS spectra were measured on Waters Q-Tof micro spectrometer, resolution: 100000. For multiply charged compounds, the nominal mass is given, and the value of the most abundant monoisotopic mass is stated in parentheses.

NMR spectroscopy: ^{1}H and ^{13}C NMR spectra were recorded on a Bruker Avance III™ 400, 500 or 600 spectrometers operating at 400, 500 or 600 MHz for ^{1}H NMR and 100.1, 125.7 or 150.9 MHz for ^{13}C NMR. Temperature-dependent spectra were recorded on a Bruker Avance II™ 500 MHz instrument. Full assignment of ^{1}H and ^{13}C signals was achieved by a combination of 2D experiments (^{1}H,^{1}H-COSY; ^{1}H-^{13}C HMBC; ^{1}H-^{13}C HSQC). Spatial long-range contacts were determined by ^{1}H,^{1}H-ROESY experiments. $CDCl_3$ was dried over 3Å molecular sieves prior to the measurements. Measurements in H_2O/D_2O were performed using selective presaturation to supress the H_2O signal.

IR spectroscopy: IR spectra of the synthetized compounds were measured on a Bruker AlPHA-FT-IR spectrometer (4 cm^{-1} spectral resolution, Happ–Genzel apodization function, 64 scans) as neat samples using an ATR device equipped with a diamond crystal in the 4000–600 cm^{-1} spectral range.

8) General procedures

tert-Butyl (1*R*,5*S*,8*R*,1'*R*)-8-methyl-7-(1-phenylethyl)-2,3,7-triazabicyclo[3.3.0]oct-2-ene-1-carboxylate **1S** or **1R** were prepared according to the literature.[1] Their spectral and physical data match those reported.

A) Peptide coupling

HATU (418 mg, 1.10 mmol) and subsequently pyrrolidine **NH$_2$-R^{P}A** or **NH$_2$-S^{P}A** (400 mg, 1.20 mmol) were added to a stirred solution of *N*-protected amino acid (1.00 mmol) and *N*,*N*-diisopropylethylamine (0.35 mL, 2.00 mmol) in DCM (20 mL) at r.t. and the reaction mixture was stirred for 2 h. After evaporation of the solvent *in vacuo*, the residue was dissolved in a 1:1 EtOAc/Et_2O mixture (25 mL) and the resulting solution was washed with saturated $NaHCO_3$ solution

(25 mL) and 5% LiCl solution (5×25 mL). The combined organic layers were dried over anhydrous Na_2SO_4 and evaporated *in vacuo*. Purification conditions are described at the individual compounds.

B) *tert*-Butyl ester deprotection and subsequent peptide coupling

TFA (2 mL) was added dropwise to a stirred solution of *tert*-butyl ester **Fmoc-2^P, Ac-2^P** or **Fmoc-4^P** (1.00 mmol) in DCM (4 mL). The reaction mixture was stirred overnight or until judged complete by TLC analysis and concentrated *in vacuo*. The crude product was redissolved in DCM (20 mL) and *N*,*N*-diisopropylethylamine (0.7 mL, 4.00 mmol for **Fmoc-2^P, Ac-2^P** or 1.0 mL, 6.00 mmol for **Fmoc-4^P**) was dropwise added, followed by free amines **NH_2-2^P** or **NH_2-4^P** (1.10 mmol) and HATU (456 mg, 1.20 mmol). The mixture was stirred for 2 h. After evaporation of the solvent *in vacuo*, the residue was dissolved in a 1:1 EtOAc/Et_2O mixture (25 mL) and the resulting solution was washed with saturated $NaHCO_3$ solution (25 mL) and 5% LiCl solution (5×25 mL). The combined organic layers were dried over anhydrous Na_2SO_4 and evaporated *in vacuo*. Purification conditions are described at the individual compounds.

C) Amine Deprotection

Fmoc-Protected peptide **Fmoc-2^P**, **Fmoc-4^P** or **Fmoc-8GR^{P}A**, **Fmoc-8AS^{P}A** (0.5 mmol) was dissolved in 20% piperidine solution in DMF (2.5 mL) and the mixture was stirred at r.t. for 10 min. The solvent was evaporated, and the crude product was purified by column chromatography. Purification conditions are described at the individual compounds.

D) Phenylethyl group deprotection

Protected peptide **Ac-6^P** or **Boc-8GR^{P}A**, **Boc-8AS^{P}A** (0.02 mmol) was dissolved in MeOH (0.5 mL), a drop of AcOH and Pearlman's catalyst (Aldrich, 0.4 mg, 0.16 µmol) were added, and the mixture was hydrogenated at 5 bar H_2 pressure at 25 °C for 12 h. The reaction mixture was filtered through celite® (1 cm) and washed with MeOH (10 mL). The filtrate was concentrated *in vacuo*, redissolved in water and neutralized through a plug of DOWEX® 1X8 chloride form, which was made basic by 0.1 M NaOH solution before, to yield the unprotected peptide as free base.

E) Boc protection of the amine

Deprotected peptide **NH_2-8GR^{P}A, NH_2-8AS^{P}A** (0.075 mmol) was dissolved in CH_2Cl_2 (1.5 mL) and di-*tert*-butyl dicarbonate (18 mg, 0.082 mmol) was added. The reaction mixture was stirred at r.t. for 30 min and evaporated providing an oily residue, which was purified by flash chromatography. Purification conditions are described at the individual compounds.

9) Experimental data and characterization

***tert*-Butyl (2*R*,3*R*,4*R*)-3-amino-4-(aminomethyl)-2-methyl-1-((*R*)-1-phenylethyl)pyrrolidine-3-carboxylate (NH_2-R^{P}A)**

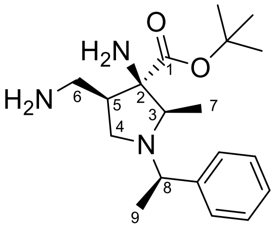

Pyrazoline **1R** (3.294 g, 10 mmol) was dissolved in MeOH (16 mL), Raney nickel suspension was added (50% suspension in H_2O (0.5 mL) washed three times with MeOH and suspended in MeOH (0.5 mL)) and the mixture was hydrogenated at 20 bar H_2 pressure at 50 °C for 24 h. The suspension was filtered through a plug of celite® (1 cm), which was washed with MeOH (25 mL). The filtrate was evaporated, and flash chromatography in $CHCl_3$/MeOH+1% Et_3N (50:1 to 10:1 gradient) afforded 2.423 g (73%) of free diamine **NH_2-R^{P}A** as an off-white solid.

Mp: 71-73 °C; $[\alpha]_D^{20}$: −31.2 (c 0.52, $CHCl_3$); **IR** ν[cm^{-1}]: 701, 755, 786, 850, 1154, 1231, 1255, 1284, 1372, 1396, 1457, 1481, 1498, 1567, 1589, 1606, 1725, 2877, 2939, 2981, 3396; **^{1}H NMR** (401 MHz, $CDCl_3$) δ 7.38-7.34 (m, 2H, Ar), 7.32-7.27 (m, 2H, Ar), 7.24-7.18 (m, 1H, Ar), 3.98 (q, *J* = 6.7 Hz, 1H, H-8), 3.10 (q, *J* = 6.2 Hz, 1H, H-3), 2.83-2.73 (m, 2H, H-4a, H-6a), 2.66-2.57 (m, 2H, H-5, H-6b), 2.15 (dd, *J* = 9.4, 7.0 Hz, 1H, H-4b), 1.51 (bs, 4H, 2NH_2), 1.48 (s, 9H, *t*Bu), 1.31 (d, *J* = 6.7 Hz, 3H, H-9), 0.93 (d, *J* = 6.2 Hz, 3H, H-7); **^{13}C NMR** (101 MHz, $CDCl_3$) δ 174.0 (C, C-1), 144.2 (C, Ar), 128.2 (CH, Ar), 127.5 (CH, Ar), 126.7 (CH, Ar), 81.5 (C, *t*Bu), 67.5 (C, C-2), 64.4 (CH, C-3), 54.8 (CH, C-8), 47.5 (CH_2, C-4), 46.9 (CH, C-5), 42.0 (CH_2, C-6), 28.1 (CH_3, *t*Bu), 12.7 (CH_3, C-7), 11.0 (CH_3, C-9); **MS** (ESI+) m/z, (%): 278 (8, $[M+H-C_4H_8]^+$), 334 (100, $[M+H]^+$), 356 (10, $[M+Na]^+$), 689 (22, $[2M+Na]^+$); **HRMS** (ESI+) m/z: $[M+H]^+$ Calcd for $C_{19}H_{32}N_3O_2$ 334.2489; Found 334.2491.

α/γ-Peptide Fmoc(Gly-(*R*,*R*,*R*,*R*)^{P}AAMP)O*t*Bu (Fmoc-2GR^{P}A)

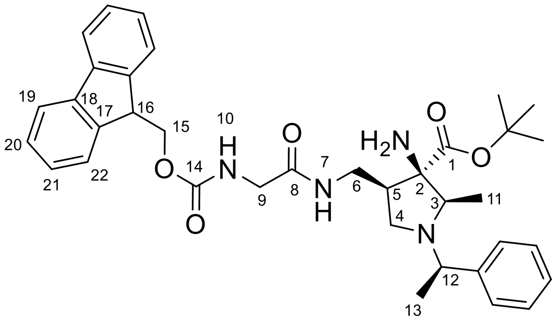

Prepared according to general **procedure A** from diamine **NH$_2$-R^{P}A** (400 mg, 1.20 mmol) and Fmoc-Gly-OH (297 mg, 1 mmol). The residue was purified by flash chromatography (cyclohexane/EtOAc, 1:1 to neat EtOAc gradient) to afford 590 mg (95%) of protected dipeptide **Fmoc-2GR^{P}A** as an off-white solid.

Mp: 130-132 °C; $[\alpha]_D^{20}$: –18.0 (c 0.2, $CHCl_3$); **IR** ν[cm^{-1}]: 646, 700, 732, 759, 783, 909, 1047, 1083, 1105, 1148, 1247, 1368, 1390, 1450, 1478, 1521, 1600, 1664, 1719, 2873, 2932, 2972, 3028, 3064, 3314; **^{1}H NMR** (401 MHz, $CDCl_3$) δ 7.77 (d, *J* = 7.5 Hz, 2H, H-19), 7.63 (d, *J* = 6.6 Hz, 2H, H-22), 7.40 (t, *J* = 7.5 Hz, 2H, H-20), 7.36-7.27 (m, 6H, Ar, H-21), 7.24-7.19 (m, 1H, Ar), 7.12 (bs, 1H, H-7), 5.44 (bs, 1H, H-10), 4.43 (dd, *J* = 10.5, 7.5 Hz, 1H, H-15a), 4.35 (dd, *J* = 10.6, 6.9 Hz, 1H, H-15b), 4.24 (t, *J* = 7.2 Hz, 1H, H-16), 3.95 (q, *J* = 6.7 Hz, 1H, H-12), 3.85 (dd, *J* = 16.9, 6.0 Hz, 1H, H-9a), 3.70 (dd, *J* = 16.8, 4.8 Hz, 1H, H-9b), 3.56 (ddd, *J* = 13.4, 7.2, 4.8 Hz, 1H, H-6a), 3.08-3.01 (m, 1H, H-6b), 3.01 (q, *J* = 6.2 Hz, 1H, H-3), 2.82-2.71 (m, 1H, H-5), 2.69 (dd, *J* = 10.0, 8.7 Hz, 1H, H-4a), 2.15 (dd, *J* = 8.6, 5.0 Hz, 1H, H-4b), 1.64 (bs, 2H, NH_2), 1.46 (s, 9H, *t*Bu), 1.30 (d, *J* = 6.7 Hz, 3H, H-13), 0.89 (d, *J* = 6.2 Hz, 3H, H-11); **^{13}C NMR** (101 MHz, $CDCl_3$) δ 174.0 (C, C-1), 168.2 (C, C-8), 156.3 (C, C-14), 144.04 (C, C-17), 143.99 (C, Ar), 141.4 (C, C-18), 128.3 (CH, Ar), 127.8 (CH, C-20), 127.5 (CH, Ar), 127.2 (CH, C-21), 126.9 (CH, Ar), 125.3 (CH, C-22), 120.1 (CH, C-19), 82.0 (C, *t*Bu), 68.0 (C, C-2), 67.3 (CH_2, C-15), 64.4 (CH, C-3), 54.9 (CH, C-12), 47.3 (CH, C-16), 47.0 (CH_2, C-4), 44.4 (CH_2, C-9), 43.0 (CH, C-5), 39.5 (CH_2, C-6), 28.1 (CH_3, *t*Bu), 12.6 (CH_3, C-11), 11.3 (CH_3, C-13); **MS** (ESI+) m/z, (%): 391 (42, $[M+H–dibenzofulvene–CO_2]^+$), 613 (100, $[M+H]^+$), 635 (14, $[M+Na]^+$); **HRMS** (ESI+) m/z: $[M+H]^+$ Calcd for $C_{36}H_{45}N_4O_5$ 613.3385; Found 613.3386.

α/γ-Peptide NH_2(Gly-(*R*,*R*,*R*,*R*)^{P}AAMP)O*t*Bu (NH_2-2GR^{P}A)

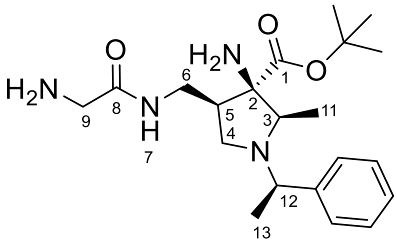

Prepared according to general **procedure C** from protected dipeptide **Fmoc-2GR^{P}A** (172 mg, 0.28 mmol). The residue was purified by flash chromatography ($CHCl_3$/MeOH + 1% TEA, neat to 25:1 gradient) to afford 103 mg (95%) of free amine **NH_2-2GR^{P}A** as a pale yellow oil.

$[\alpha]^{20}_{D}$: –20.4 (c 0.2, $CHCl_3$); **IR** ν[cm^{-1}]: 663, 700, 751, 784, 847, 1148, 1254, 1277, 1368, 1391, 1452, 1477, 1494, 1529, 1601, 1655, 1720, 2872, 2930, 2972, 3027, 3060, 3083, 3305, 3364; **^{1}H NMR** (401 MHz, $CDCl_3$) δ 7.62 (dd, *J* = 5.2, 6.5 Hz, 1H, H-7), 7.37-7.27 (m, 4H, Ar), 7.25-7.19 (m, 1H, Ar), 3.96 (q, *J* = 6.7 Hz, 1H, H-12), 3.49 (ddd, *J* = 13.1, 7.3, 4.9 Hz, 1H, H-6a), 3.23 (s, 2H, H-9), 3.14-3.04 (m, 1H, H-6b), 3.04 (q, *J* = 6.3 Hz, 1H, H-3), 2.85-2.74 (m, 2H, H-5, H-4a), 2.20 (dd, *J* = 8.5, 5.5 Hz, 1H, H-4b), 1.72 (bs, 4H, 2NH_2), 1.45 (s, 9H, *t*Bu), 1.31 (d, *J* = 6.8 Hz, 3H, H-13), 0.89 (d, *J* = 6.2 Hz, 3H, H-11); **^{13}C NMR** (101 MHz, $CDCl_3$) δ 173.6 (C, C-1), 172.4 (C, C-8), 143.6 (C, Ar), 128.3 (CH, Ar), 127.6 (CH, Ar), 127.0 (CH, Ar), 81.7 (C, *t*Bu), 67.8 (C, C-2), 64.8 (CH, C-3), 55.6 (CH, C-12), 47.5 (CH_2, C-4), 44.9 (CH_2, C-9), 43.5 (CH, C-5), 38.6 (CH_2, C-6), 28.0 (CH_3, *t*Bu), 12.6 (CH_3, C-13), 11.7 (CH_3, C-11); **MS** (ESI+) m/z, (%): 335 (22, $[M+H-C_4H_8]^+$), 391 (100, $[M+H]^+$), 413 (16, $[M+Na]^+$); **HRMS** (ESI+) m/z: $[M+H]^+$ Calcd for $C_{21}H_{35}N_4O_3$ 391.2704; Found 391.2700.

α/γ-Peptide Ac(Gly-(*R*,*R*,*R*,*R*)^{P}AAMP)O*t*Bu (Ac-2GR^{P}A)

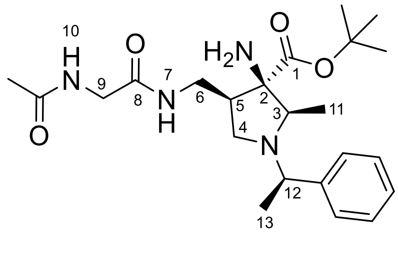

Prepared according to general **procedure A** from diamine **NH_2-R^{P}A** (400 mg, 1.20 mmol) and Ac-Gly-OH (117 mg, 1 mmol). The residue was purified by flash chromatography (cyclohexane/EtOAc, 1:1, gradient to neat EtOAc) to afford 398 mg (92%) of protected dipeptide **Ac-2GR^{P}A** as a pale yellow oil.

$[\alpha]_D^{20}$: −19.5 (c 0.2, $CHCl_3$); **IR** ν[cm^{-1}]: 664, 700, 752, 784, 847, 982, 1027, 1149, 1253, 1277, 1369, 1391, 1452, 1495, 1542, 1600, 1652, 1721, 2871, 2931, 2973, 3027, 3063, 3083, 3293; **^{1}H NMR** (401 MHz, $CDCl_3$) δ 7.38-7.27 (m, 4H, Ar), 7.25-7.19 (m, 1H, Ar), 7.06 (dd, *J* = 6.8, 5.2 Hz, 1H, H-7), 6.30 (bs, 1H, H-10), 3.96 (q, *J* = 6.7 Hz, 1H, H-12), 3.89 (dd, *J* = 16.8, 5.5 Hz, 1H, H-9a), 3.71 (dd, *J* = 16.8, 4.4 Hz, 1H, H-9b), 3.52 (ddd, *J* = 12.0, 7.0, 3.5 Hz, 1H, H-6a), 3.08-2.98 (m, 1H, H-6b), 3.01 (q, *J* = 6.2 Hz, 1H, H-3), 2.80-2.68 (m, 2H, H-4a, H-5), 2.18-2.14 (m, 1H, H-4b), 2.02 (s, 3H, Ac), 1.79 (s, 2H, NH_2), 1.45 (s, 9H, *t*Bu), 1.30 (d, *J* = 6.8 Hz, 3H, H-13), 0.89 (d, *J* = 6.2 Hz, 3H, H-11); **^{13}C NMR** (101 MHz, $CDCl_3$) δ 173.9 (C, C-1), 170.2 (C, Ac), 168.2 (C, C-8), 144.0 (C, Ar), 128.3 (CH, Ar), 127.5 (CH, Ar), 126.9 (CH, Ar), 82.0 (C, *t*Bu), 68.0 (C, C-2), 64.4 (CH, C-3), 54.9 (CH, C-12), 47.0 (CH_2, C-4), 43.1 (CH, C-5), 43.0 (CH_2, C-9), 39.4 (CH_2, C-6), 28.1 (CH_3, *t*Bu), 23.1 (CH_3, Ac), 12.6 (CH_3, C-11), 11.3 (CH_3, C-13); **MS** (ESI+) m/z, (%): 433 (100, $[M+H]^+$), 455 (7, $[M+Na]^+$); **HRMS** (ESI+) m/z: $[M+H]^+$ Calcd for $C_{23}H_{37}N_4O_4$ 433.2809; Found 433.2805.

α/γ-Peptide Ac(Gly-(*R*,*R*,*R*)^{H}AAMP)O*t*Bu (Ac-2GR^{H}A)

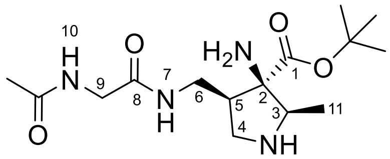

Prepared according to general **procedure D** from dipeptide **Ac-2GR^{P}A** (9 mg, 0.02 mmol) providing 6 mg (95%) of free base **Ac-2GR^{H}A** as a pale yellow oil.

$[\alpha]_D^{20}$: −13.5 (c 0.2, MeOH); **IR** ν[cm^{-1}]: 845, 1031, 1158, 1252, 1369, 1393, 1457, 1549, 1633, 1719, 2851, 2926, 2973, 3278; **^{1}H NMR** (400 MHz, MeOD) δ 3.79 (d, *J* = 16.6 Hz, 1H, H-9a), 3.70 (d, *J* = 16.6 Hz, 1H, H-9b), 3.44 (dd, *J* = 14.0, 8.8 Hz, 1H, H-6a), 3.41 (q, *J* = 6.6 Hz, 1H, H-3), 3.21-3.11 (m, 2H, H-4a, H-6b), 2.99-2.90 (m, 1H, H-5), 2.77 (dd, *J* = 11.2, 9.4 Hz, 1H, H-4b), 2.00 (s, 3H, Ac), 1.48 (s, 9H, *t*Bu), 1.08 (d, *J* = 6.7 Hz, 3H, H-11); **^{13}C NMR** (101 MHz, MeOD) δ 174.8 (C, C-1), 173.8 (C, Ac), 171.8 (C, C-8), 83.1 (C, *t*Bu), 67.9 (C, C-2), 64.2 (CH, C-3), 49.2 (CH, C-5), 48.5 (CH_2, C-4), 43.6 (CH_2, C-9), 38.6 (CH_2, C-6), 28.2 (CH_3, *t*Bu), 22.4 (CH_3, Ac), 12.3 (CH_3, C-13); **MS** (ESI+) m/z, (%): 273 (100, $[M+H-C_4H_8]^+$), 329 (73, $[M+H]^+$), 351 (60, $[M+Na]^+$); **HRMS** (ESI+) m/z: $[M+H]^+$ Calcd for $C_{15}H_{29}N_4O_4$ 329.2183; Found 329.2183.

α/γ-Peptide Fmoc(Gly-(*R*,*R*,*R*,*R*)[P]AAMP-Gly)O*t*Bu (Fmoc-3GR[P]AG)

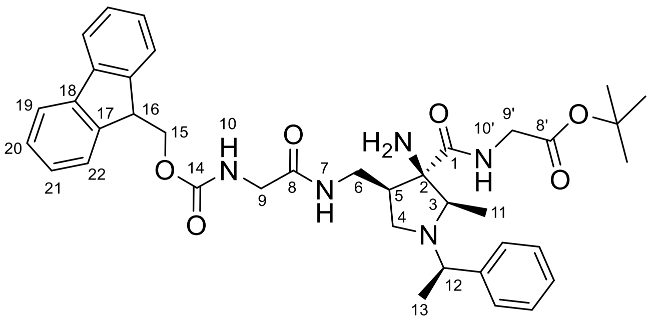

Prepared according to general **procedure B** from dipeptide **Fmoc-2GR[P]A** (74 mg, 0.122 mmol), DIPEA (0.1 mL, 0.610 mmol), glycine *tert*-butyl ester hydrochloride (31 mg, 0.183 mmol) and HATU (51 mg, 0.134 mmol). The residue was purified by flash chromatography (cyclohexane/EtOAc, 1:1 to neat EtOAc gradient) to afford 80 mg (98%) of protected tripeptide **Fmoc-3GR[P]AG** as an off-white solid.

Mp: 93-96 °C; $[\alpha]_D^{20}$: –15.5 (c 0.2, $CHCl_3$); **IR** ν[cm^{-1}]: 646, 700, 734, 759, 782, 845, 910, 1155, 1236, 1369, 1391, 1450, 1478, 1521, 1601, 1610, 1657, 1729, 2854, 2873, 2930, 3063, 3308, 3360; **^{1}H NMR** (401 MHz, $CDCl_3$) δ 8.05 (t, *J* = 6.0 Hz, 1H, H-10'), 7.76 (d, *J* = 7.5 Hz, 2H, H-19), 7.60 (d, *J* = 7.4 Hz, 2H, H-22), 7.42-7.28 (m, 8H, H-20, H-21, Ar), 7.25-7.20 (m, 1H, Ar), 6.47-6.40 (m, 1H, H-7), 5.91 (t, *J* = 5.9 Hz, 1H, H-10), 4.42 (dd, *J* = 10.7, 7.2 Hz, 1H, H-15a), 4.38 (dd, *J* = 10.5, 6.8 Hz, 1H, H-15b), 4.21 (t, *J* = 7.0 Hz, 1H, H-16), 4.06 (dd, *J* = 17.6, 6.4 Hz, 1H, H-9a'), 4.03 (q, *J* = 6.8 Hz, 1H, H-12), 3.91 (dd, *J* = 16.9, 6.3 Hz, 1H, H-9a), 3.72 (dd, *J* = 17.1, 5.5 Hz, 1H, H-9b), 3.70 (dd, *J* = 17.7, 5.6 Hz, 1H, H-9b'), 3.61-3.50 (m, 1H, H-6a), 3.20 (q, *J* = 6.3 Hz, 1H, H-3), 3.05-2.94 (m, 2H, H-5, H-6b), 2.84 (t, *J* = 9.8 Hz, 1H, H-4a), 2.09 (dd, *J* = 9.7, 7.6 Hz, 1H, H-4b), 1.96 (s, 2H, NH_2), 1.45 (s, 9H, *t*Bu), 1.31 (d, *J* = 6.7 Hz, 3H, H-13), 0.98 (d, *J* = 6.3 Hz, 3H, H-11); **^{13}C NMR** (101 MHz, $CDCl_3$) δ 175.6 (C, C-1), 169.9 (C, C-8), 169.7 (C, C-8'), 156.8 (C, C-14), 144.0 (C, C-17), 143.6 (C, Ar), 141.4 (C, C-18), 128.3 (CH, Ar), 127.8 (CH, C-20), 127.6 (CH, Ar), 127.20/127.19 (CH, C-21), 126.9 (CH, Ar), 125.2 (CH, C-22), 120.1 (CH, C-19), 82.5 (C, *t*Bu), 67.2 (CH_2, C-15), 66.5 (C, C-2), 63.7 (CH, C-3), 54.4 (CH, C-12), 47.3 (CH, C-16), 46.5 (CH_2, C-4), 44.5 (CH_2, C-9), 43.6 (CH, C-5), 42.0 (CH_2, C-9'), 38.2 (CH_2, C-6), 28.2 (CH_3, *t*Bu), 12.4 (CH_3, C-11), 10.4 (CH_3, C-13); **MS** (ESI+) m/z, (%): 392 (9, $[M+H-C_4H_8-\text{dibenzofulvene}-CO_2]^+$), 448 (9, $[M+H-\text{dibenzofulvene}-CO_2]^+$), 670 (100, $[M+H]^+$), 692 (38, $[M+Na]^+$); **HRMS** (ESI+) m/z: $[M+H]^+$ Calcd for $C_{38}H_{48}N_5O_6$ 670.3599; Found 670.3600.

α/γ-Peptide Fmoc(Gly-(*R*,*R*,*R*,*R*)^{P}AAMP)$_2$O*t*Bu (Fmoc-4GR^{P}A)

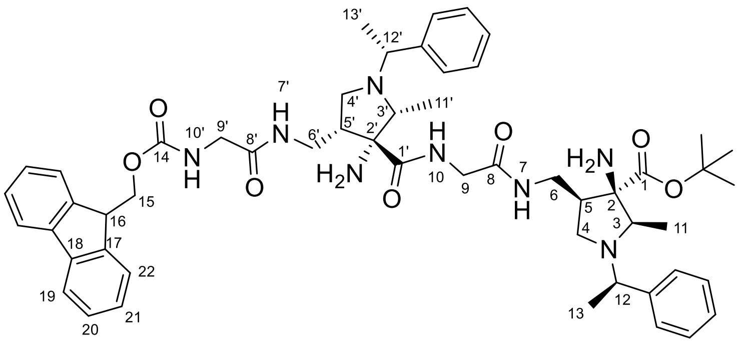

Prepared according to general **procedure B** from dipeptide **Fmoc-2GR^{P}A** (705 mg, 1.15 mmol)**,** DIPEA (0.8 mL, 4.60 mmol), free amine **NH_2-2GR^{P}A** (494 mg, 1.27 mmol) and HATU (524 mg, 1.38 mmol). The residue was purified by flash chromatography (EtOAc/MeOH, 10:1 to 2:1 gradient) to afford 992 mg (95%) of protected tetrapeptide **Fmoc-4GR^{P}A** as an off-white solid.

Mp: 99-101 °C; $[\alpha]_D^{20}$: –11.0 (c 0.2, $CHCl_3$); **IR** ν[cm^{-1}]: 645, 700, 736, 759, 784, 910, 1150, 1252, 1369, 1390, 1450, 1478, 1493, 1521, 1556, 1602, 1655, 1668, 1720, 2873, 2931, 2970, 3029, 3063, 3132, 3308, 3352; **^{1}H NMR** (600 MHz, $CDCl_3$) δ 8.00 (dd, *J* = 6.8, 5.1 Hz, 1H, H-10), 7.75 (d, *J* = 7.5 Hz, 2H, H-19), 7.61-7.57 (m, 2H, H-22), 7.40-7.34 (m, 4H, H-20, Ar), 7.34-7.20 (m, 9H, H-21, Ar), 7.19-7.17 (m, 1H, Ar), 7.01 (bs, 1H, H-7), 6.62 (bs, 1H, H-7'), 6.38 (dd, *J* = 6.3, 5.4 Hz, 1H, H-10'), 4.35 (dd, *J* = 10.6, 7.4 Hz, 1H, H-15a), 4.31 (dd, *J* = 10.6, 7.1 Hz, 1H, H-15b), 4.19 (t, *J* = 7.2 Hz, 1H, H-16), 4.04 (dd, *J* = 16.2, 7.0 Hz, 1H, H-9a), 4.02 (q, *J* = 6.5 Hz, 1H, H-12'), 3.92 (q, *J* = 6.7 Hz, 1H, H-12), 3.87 (dd, *J* = 17.0, 6.5 Hz, 1H, H-9a'), 3.67 (dd, *J* = 17.1, 5.2 Hz, 1H, H-9b'), 3.54 (dt, *J* = 12.8, 6.1 Hz, 1H, H-6a), 3.48-3.41 (m, 1H, H-6a'), 3.46 (dd, *J* = 16.3, 4.8 Hz, 1H, H-9b), 3.19 (q, *J* = 6.1 Hz, 1H, H-3'), 3.05-2.93 (m, 3H, H-5', H-6b, H-6b'), 2.98 (q, *J* = 6.2 Hz, 1H, H-3), 2.83 (t, *J* = 9.2 Hz, 1H, H-4a'), 2.75 (tt, *J* = 10.2, 5.7 Hz, 1H, H-5), 2.66 (t, *J* = 9.8 Hz, 1H, H-4a), 2.12-2.03 (m, 2H, H-4b, H-4b'), 1.75 (bs, 4H, $2NH_2$), 1.46 (s, 9H, *t*Bu), 1.30 (d, *J* = 6.5 Hz, 3H, H-13'), 1.24 (d, *J* = 6.7 Hz, 3H, H-13), 0.95 (d, *J* = 6.2 Hz, 3H, H-11'), 0.87 (d, *J* = 6.2 Hz, 3H, H-11); **^{13}C NMR** (151 MHz, $CDCl_3$) δ 175.6 (C, C-1'), 173.8 (C, C-1), 170.0 (C, C-8'), 168.9 (C, C-8), 156.9 (C, C-14), 144.1/144.0 (C, C-17), 143.9 (2C, Ar), 141.4 (C, C-18), 128.29 (CH, Ar), 128.27 (CH, Ar), 127.8 (CH, C-20), 127.6 (CH, Ar), 127.5 (CH, Ar), 127.20 (CH, C-21), 127.18 (CH, Ar), 126.9 (CH, Ar), 125.33/125.30 (CH, C-22), 120.1 (CH, C-19), 81.9 (C, *t*Bu), 67.8 (C, C-2), 67.1 (CH_2, C-15), 66.6 (C, C-2'), 64.5 (CH, C-3), 63.7 (CH, C-3'), 55.0 (CH, C-12), 54.2 (CH, C-12'), 47.2 (CH, C-16), 47.1 (CH_2, C-4), 46.6 (CH_2, C-4'), 44.5 (CH_2, C-9'), 43.5 (CH, C-5'), 42.9 (CH, C-5), 42.8 (CH_2, C-9), 39.6 (CH_2, C-6), 38.2 (CH_2, C-6'), 28.1 (CH_3, *t*Bu), 12.64 (CH_3, C-11), 12.56 (CH_3, C-11'), 11.4 (CH_3, C-13), 10.3 (CH_3, C-13'); **MS** (ESI+) m/z, (%): 326.0 (326.4) (12, $[M+2H-C_4H_8-\text{dibenzofulvene}-CO_2]^{2+}$), 437.0 (437.3) (69, $[M+2H-C_4H_8]^{2+}$), 465.0 (465.3) (100, $[M+2H]^{2+}$), 476.0 (476.3) (15, $[M+H+Na]^{2+}$), 929 (24,

[M+H]+), 951 (48, [M+Na]+); **HRMS** (ESI+) m/z: [M+H]+ Calcd for $C_{53}H_{69}N_8O_7$ 929.5284; Found 929.5279.

α/γ-Peptide NH2(Gly-(*R*,*R*,*R*,*R*)PAAMP)2O*t*Bu (NH2-4GRPA)

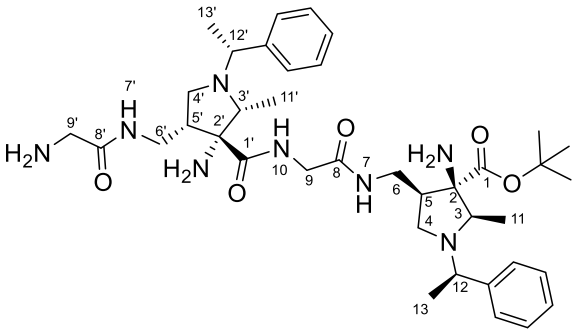

Prepared according to general **procedure C** from protected tetrapeptide **Fmoc-4GRPA** (585 mg, 0.63 mmol). The residue was purified by flash chromatography ($CHCl_3$/MeOH + 1% TEA, neat to 10:1 gradient) to afford 435 mg (98%) of free amine **NH2-2GRPA** as an off-white solid.

Mp: 103-105 °C; $[\alpha]_D^{20}$: –17.5 (c 0.2, $CHCl_3$); **IR** ν[cm^{-1}]: 700, 747, 784, 817, 846, 952, 1151, 1257, 1369, 1391, 1455, 1495, 1522, 1541, 1600, 1648, 1679, 1719, 2874, 2930, 2968, 3031, 3064, 3085, 3293, 3355; **^{1}H NMR** (500 MHz, $CDCl_3$) δ 8.08 (t, *J* = 5.9 Hz, 1H, H-10), 7.37-7.28 (m, 9H, Ar, H-7'), 7.24-7.20 (m, 2H, Ar), 7.09 (bs, 1H, H-7), 4.03 (q, *J* = 6.6 Hz, 1H, H-12'), 3.96 (q, *J* = 6.6 Hz, 1H, H-12), 3.86 (dd, *J* = 16.6, 6.4 Hz, 1H, H-9a), 3.71 (dd, *J* = 16.6, 5.3 Hz, 1H, H-9b), 3.49-3.40 (m, 2H, H-6a, H-6a'), 3.18 (q, *J* = 6.1 Hz, 1H, H-3'), 3.16 (s, 2H, H-9'), 3.11 (ddd, *J* = 14.0, 9.2, 4.3 Hz, 1H, H-6b), 3.03 (q, *J* = 6.2 Hz, 1H, H-3), 3.00-2.92 (m, 2H, H-6b', H-5'), 2.82-2.74 (m, 1H, H-5), 2.81 (t, *J* = 9.8, Hz, 1H, H-4a'), 2.73 (dd, *J* = 10.0, 8.9 Hz, 1H, H-4a), 2.16 (dd, *J* = 8.5, 5.5 Hz, 1H, H-4b), 2.07 (dd, *J* = 9.5, 7.9 Hz, 1H, H-4b'), 1.89 (s, 6H, 3NH_2), 1.47 (s, 9H, *t*Bu), 1.30 (d, *J* = 6.7 Hz, 6H, H-13, H-13'), 0.99 (d, *J* = 6.2 Hz, 3H, H-11'), 0.90 (d, *J* = 6.2 Hz, 3H, H-11); **^{13}C NMR** (126 MHz, CDCl3) δ 175.3 (C, C-1'), 173.8 (C, C-1), 173.4 (C, C-8'), 168.7 (C, C-8), 144.1 (C, Ar), 143.8 (C, Ar'), 128.26 (CH, Ar), 128.25 (CH, Ar), 127.6 (CH, Ar), 127.5 (CH, Ar), 126.83 (CH, Ar), 126.78 (CH, Ar), 81.9 (C, *t*Bu), 67.8 (C, C-2), 66.6 (C, C-2'), 64.4 (CH, C-3), 63.4 (CH, C-3'), 54.9 (CH, C-12), 54.1 (CH, C-12'), 47.1 (CH_2, C-4), 46.3 (CH_2, C-4'), 44.6 (CH_2, C-9'), 44.0 (CH, C-5'), 43.3 (CH, C-5), 43.0 (CH_2, C-9), 39.3 (CH_2, C-6), 37.7 (CH_2, C-6'), 28.1 (CH_3, *t*Bu), 12.72 (CH_3, C-11), 12.66 (CH_3, C-11'), 11.3 (CH_3, C-13), 10.2 (CH_3, C-13'); **MS** (ESI+) m/z, (%): 354.0 (354.2) (100, [M+2H]$^{2+}$), 729 (11, [M+Na]+); **HRMS** (ESI+) m/z: [M+Na]+ Calcd for $C_{38}H_{58}N_8O_5Na$ 729.4422; Found 729.4417.

α/γ-Peptide Ac(Gly-(*R*,*R*,*R*,*R*)[P]AAMP)$_2$O*t*Bu (Ac-4GR[P]A)

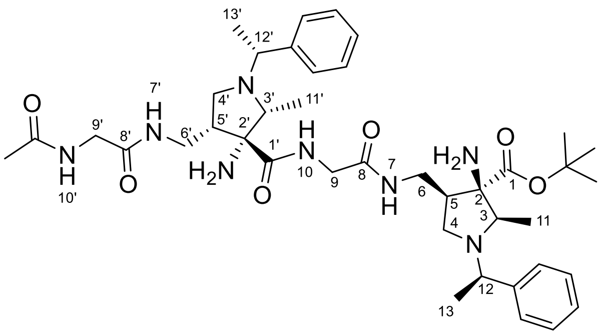

Prepared according to general **procedure B** from dipeptide **2R[P]AG-Ac** (50 mg, 0.12 mmol), DIPEA (0.08 mL, 0.46 mmol), free amine **NH$_2$-2GR[P]A** (49 mg, 0.13 mmol) and HATU (52 mg, 0.14 mmol). The residue was purified by flash chromatography (EtOAc/MeOH, 10:1 to 1:1 gradient) to afford 79 mg (92%) of protected hexapeptide **Ac-4GR[P]A** as an off-white solid.

$[\alpha]_D^{20}$: –4.5 (c 0.2, CHCl$_3$); **IR** ν[cm^{-1}]: 663, 701, 845, 960, 1026, 1147, 1265, 1371, 1409, 1453, 1495, 1518, 1602, 1665, 1719, 2855, 2932, 2976, 3008, 3064, 3088 3373; **^{1}H NMR** (400 MHz, CDCl$_3$) δ 8.05 (dd, *J* = 6.7, 4.9 Hz, 1H, H-10), 7.40-7.17 (m, 10H, Ar), 7.17-7.05 (m, 2H, H-7, H-10'), 6.75 (bs, 1H, H-7'), 4.03 (dd, *J* = 16.3, 6.9 Hz, 1H, H-9a), 4.01 (q, *J* = 6.6 Hz, 1H, H-12'), 3.96 (q, *J* = 6.8 Hz, 1H, H-12), 3.91 (dd, *J* = 16.8, 6.1 Hz, 1H, H-9a'), 3.62 (dd, *J* = 16.8, 5.0 Hz, 1H, H-9b'), 3.53-3.44 (m, 1H, H-6a), 3.51 (dd, *J* = 16.3, 4.8 Hz, 1H, H-9b), 3.46-3.37 (m, 1H, H-6a'), 3.17 (q, *J* = 6.0 Hz, 1H, H-3'), 3.10-2.99 (m, 1H, H-6b), 3.02 (q, *J* = 6.2 Hz, 1H, H-3), 2.99-2.87 (m, 2H, H-5', H-6b'), 2.85-2.71 (m, 3H, H-4a, H-4a', H-5), 2.18-2.13 (m, 1H, H-4b), 2.09-1.97 (m, 5H, H-4b', 2NH$_2$), 1.96 (s, 3H, Ac), 1.46 (s, 9H, *t*Bu), 1.30 (d, *J* = 6.7 Hz, 3H, H-13), 1.29 (d, *J* = 6.7 Hz, 3H, H-13'), 0.94 (d, *J* = 6.4 Hz, 3H, H-11'), 0.90 (d, *J* = 6.2 Hz, 3H, H-11); **^{13}C NMR** (101 MHz, CDCl$_3$) δ 175.5 (C, C-1'), 173.7 (C, C-1), 170.9 (C, Ac), 169.8 (C, C-8'), 168.9 (C, C-8), 144.0 (C, Ar), 143.7 (C, Ar'), 128.29 (CH, Ar), 128.26 (CH, Ar), 127.53 (CH, Ar), 127.46 (CH, Ar), 126.9 (CH, Ar), 126.8 (CH, Ar), 81.9 (C, *t*Bu), 67.9 (C, C-2), 66.5 (C, C-2'), 64.4 (CH, C-3), 63.5 (CH, C-3'), 54.9 (CH, C-12), 54.3 (CH, C-12'), 47.1 (CH$_2$, C-4), 46.4 (CH$_2$, C-4'), 43.6 (CH, C-5'), 43.03 (CH$_2$, C-9'), 42.98 (CH, C-5), 42.8 (CH$_2$, C-9), 39.6 (CH$_2$, C-6), 38.1 (CH$_2$, C-6'), 28.1 (CH$_3$, *t*Bu), 23.1 (CH$_3$, Ac), 12.7 (CH$_3$, C-11), 12.6 (CH$_3$, C-11'), 11.3 (CH$_3$, C-13), 10.2 (CH$_3$, C-13'); **MS** (ESI+) m/z, (%): 347.0 (347.2) (64, $[M+2H-C_4H_8]^{2+}$), 375.0 (375.2) (100, $[M+2H]^{2+}$), 589 (61, $[M+H-C_4H_8-PhCH=CH_2]^+$), 749 (18, $[M+H]^+$), 771 (77, $[M+Na]^+$); **HRMS** (ESI+) m/z: $[M+H]^+$ Calcd for $C_{40}H_{61}N_8O_6$ 749.4709; Found 749.4708.

α/γ-Peptide Ac(Gly-(*R*,*R*,*R*)^H^AAMP)$_2$O*t*Bu (Ac-4GR^H^A)

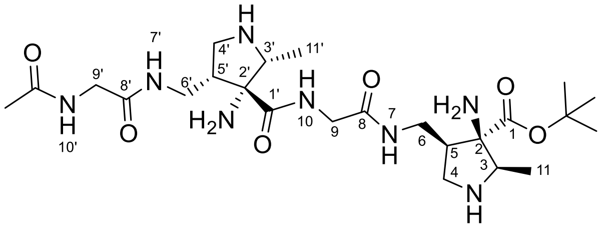

Prepared according to general **procedure D** from tetrapeptide **Ac-4GR^P^A** (15 mg, 0.02 mmol) providing 10 mg (95%) of free base **Ac-2GR^H^A** as a pale yellow oil.

$[\alpha]_D^{20}$: –13.2 (c 0.2, MeOH); **IR** ν[cm^{-1}]: 845, 1031, 1157, 1255, 1369, 1458, 1532, 1657, 1723, 2851, 2924, 2974, 2393; **^{1}H NMR** (400 MHz, MeOD) δ 3.86-3.72 (m, 4H, H-9, H-9'), 3.46 (dd, *J* = 14.0, 9.2 Hz, 1H, H-6a), 3.40 (dd, *J* = 13.9, 9.3 Hz, 1H, H-6a'), 3.31 (q, *J* = 6.5 Hz, 1H, H-3'), 3.29 (q, *J* = 6.6 Hz, 1H, H-3), 3.21-3.13 (m, 2H, H-6b, H-6b'), 3.11-3.02 (m, 2H, H-4a, H-4a'), 3.02-2.86 (m, 2H, H-5, H-5'), 2.72-2.62 (m, 2H, H-4b, H-4b'), 2.01 (s, 3H, Ac), 1.48 (s, 9H, *t*Bu), 1.026 (d, *J* = 6.6 Hz, 3H, H-11'), 1.023 (d, *J* = 6.6 Hz, 3H, H-11); **^{13}C NMR** (101 MHz, MeOD) δ 177.3 (C-1'), 175.5 (C-1), 173.7 (C-10'), 171.8 (C-7'), 171.4 (C-7), 82.8 (C, *t*Bu), 68.3 (C, C-2), 68.1 (C, C-2'), 64.3 (CH, C-3), 64.2 (CH, C-3'), 49.9 (CH, C-5), 49.5 (CH, C-5'), 49.0 (CH$_2$, C-4'), 48.6 (CH$_2$, C-4), 43.5 (CH$_2$, C-9), 43.4 (CH$_2$, C-9'), 38.9 (CH$_2$, C-6), 38.5 (CH$_2$, C-6'), 28.3 (CH$_3$, *t*Bu), 22.6 (CH$_3$, Ac), 12.9 (CH$_3$, C-11'), 12.7 (CH$_3$, C-1); **MS** (ESI+) m/z, (%): 243.0 (243.1) (100, $[M+2H-C_4H_8]^{2+}$), 271.0 (271.2) (9, $[M+2H]^{2+}$), 485 (5, $[M+H-C_4H_8]^+$), 541 (20, $[M+H]^+$), 563 (56, $[M+Na]^+$); **HRMS** (ESI+) m/z: $[M+H]^+$ Calcd for $C_{24}H_{45}N_8O_6$ 541.3457; Found 541.3455.

α/γ-Peptide Fmoc(Gly-(*R*,*R*,*R*,*R*)^P^AAMP)$_3$O*t*Bu (Fmoc-6GR^P^A)

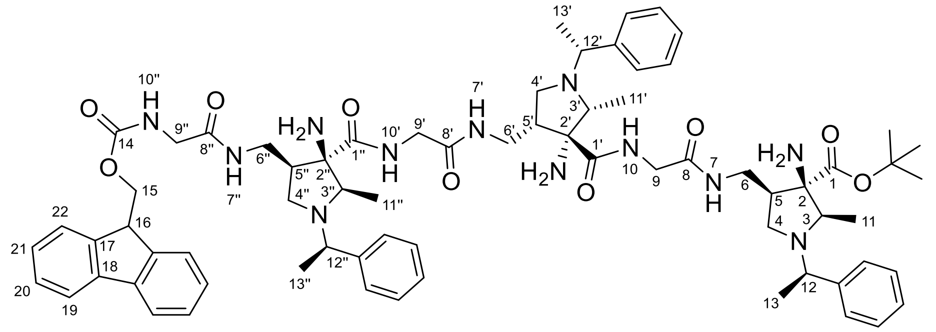

Prepared according to general **procedure B** from dipeptide **Fmoc-2GR^P^A** (14 mg, 0.023 mmol), DIPEA (18 µL, 0.102 mmol), free amine **NH$_2$-4GR^P^A** (18 mg, 0.025 mmol) and HATU (11 mg, 0.028 mmol). The residue was purified by flash chromatography (EtOAc/MeOH, 10:1 to 1:1 gradient) to afford 25 mg (89%) of protected hexapeptide **Fmoc-6GR^P^A** as an off-white solid.

Mp: 120-123 °C; $[\alpha]_D^{20}$: –9.0 (c 0.2, $CHCl_3$); **IR** ν[cm^{-1}]: 646, 699, 731, 758, 784, 909, 1046, 1084, 1103, 1150, 1259, 1369, 1390, 1449, 1475, 1491, 1516, 1601, 1652, 1721, 2853, 2927, 2966, 3029, 3064, 3084, 3305, 3363; **^{1}H NMR** (600 MHz, $CDCl_3$) δ 8.17 (t, *J* = 5.6 Hz, 1H, H-10), 8.01 (dd, *J* = 4.6, 7.4 Hz, 1H, H-10'), 7.75 (d, *J* = 7.5 Hz, 2H, H-19), 7.60 (d, *J* = 6.4 Hz, 2H, H-22), 7.40-7.13 (m, 19H, Ar, H-20, H-21), 7.04 (t, *J* = 5.7 Hz, 1H, H-7), 6.99 (bs, 1H, H-7'), 6.94 (bs, 1H, H-7''), 6.34 (t, *J* = 5.6 Hz, 1H, H-10''), 4.38 (dd, *J* = 10.4, 7.4 Hz, 1H, H-15a), 4.33 (dd, *J* = 10.5, 7.2 Hz, 1H, H-15b), 4.22 (t, *J* = 7.2 Hz, 1H, H-16), 4.08 (dd, *J* = 16.4, 7.6 Hz, 1H, H-9a'), 4.002 (q, *J* = 6.5 Hz, 1H, H-12''), 3.999 (dd, *J* = 16.4, 6.1 Hz, 1H, H-9a), 3.95 (q, *J* = 6.7 Hz, 2H, H-12, H-12'), 3.88 (dd, *J* = 17.1, 5.9 Hz, 1H, H-9a''), 3.72 (dd, *J* = 16.9, 5.7 Hz, 1H, H-9b''), 3.57 (dd, *J* = 16.3, 5.1 Hz, 1H, H-9b), 3.54-3.47 (m, 1H, H-6a), 3.46-3.41 (m, 1H, H-6a''), 3.41 (dd, *J* = 16.5, 4.5 Hz, 1H, H-9b'), 3.38-3.31 (m, 1H, H-6a'), 3.24 (q, *J* = 6.3 Hz, 1H, H-3'), 3.16 (q, *J* = 6.2 Hz, 1H, H-3''), 3.08-2.98 (m, 3H, H-6b, H-6b', H-6b''), 3.01 (q, *J* = 6.2 Hz, 1H, H-3), 2.96-2.88 (m, 1H, H-5''), 2.86-2.71 (m, 5H, H-4a, H-4a', H-4a'', H-5', H-5), 2.16 (dd, *J* = 8.4, 5.0 Hz, 1H, H-4b), 2.11-1.93 (m, 8H, H-4b', H-4b'', 3NH_2), 1.46 (s, 9H, *t*Bu), 1.291 (d, *J* = 6.7 Hz, 3H, H-13), 1.289 (d, *J* = 6.6 Hz, 3H, H-13''), 1.26 (d, *J* = 6.7 Hz, 3H, H-13'), 0.91 (d, *J* = 6.3 Hz, 3H, H-11'), 0.904 (d, *J* = 6.3 Hz, 3H, H-11''), 0.899 (d, *J* = 6.2 Hz, 3H, H-11); **^{13}C NMR** (151 MHz, $CDCl_3$) δ 175.5 (C, C-1'), 175.4 (C, C-1''), 173.8 (C, C-1), 170.1 (C, C-8''), 169.8 (C, C-8'), 169.1 (C, C-8), 156.9 (C, C-14), 144.1 (C, Ar), 144.04/144.00 (C, C-17), 143.8 (C, Ar), 143.7 (C, Ar), 141.42/141.41 (C, C-18), 128.32 (CH, Ar), 128.29 (CH, Ar), 128.2 (CH, Ar), 127.8 (CH, C-20), 127.6 (2CH, Ar), 127.5 (CH, Ar), 127.2 (CH, C-21), 126.91 (CH, Ar), 126.86 (CH, Ar), 126.8 (CH, Ar), 125.4/125.3 (CH, C-22), 120.1 (CH, C-19), 81.9 (C, *t*Bu), 67.9 (C, C-2), 67.2 (CH_2, C-15), 66.6 (2C, C-2', C-2''), 64.5 (CH, C-3), 63.6 (CH, C-3''), 63.4 (CH, C-3'), 55.0 (CH, C-12), 54.7 (CH, C-12'), 54.4 (CH, C-12''), 47.3 (CH, C-16), 47.2 (CH_2, C-4), 46.8 (CH_2, C-4'), 46.7 (CH_2, C-4''), 44.4 (CH_2, C-9''), 44.0 (CH, C-5'), 43.6 (CH, C-5''), 43.1 (CH, C-5), 43.0 (CH_2, C-9), 42.6 (CH_2, C-9'), 39.6 (CH_2, C-6), 38.3 (CH_2, C-6''), 38.1 (CH_2, C-6'), 28.1 (CH_3, *t*Bu), 12.8 (CH_3, C-11), 12.7 (2CH_3, C-11', C-11''), 11.4 (CH_3, C-13), 10.8 (CH_3, C-13'), 10.4 (CH_3, C-13''); **MS** (ESI+) m/z, (%): 415.6 (415.9) (14, $[M+3H]^{3+}$), 543.0 (543.3) (10, $[M+2H-C_4H_8-PhCH=CH_2]^{2+}$), 595.0 (595.3) (11, $[M+2H-C_4H_8]^{2+}$), 623.0 (623.4) (86, $[M+2H]^{2+}$), 1245 (100, $[M+H]^+$), 1267 (8, $[M+Na]^+$); **HRMS** (ESI+) m/z: $[M+H]^+$ Calcd for $C_{55}H_{83}N_{12}O_7$ 1245.7183; Found 1245.7186.

α/γ-Peptide Ac(Gly-(*R*,*R*,*R*,*R*)^{P}AAMP)$_3$O*t*Bu (Ac-6GR^{P}A)

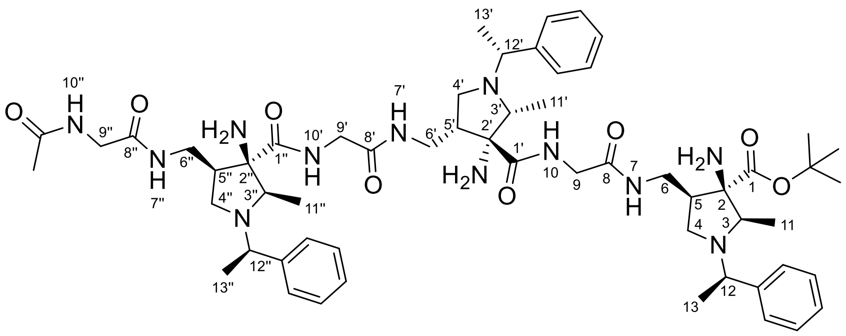

Prepared according to general **procedure B** from dipeptide **Ac-2GR^{P}A** (61 mg, 0.14 mmol), DIPEA (0.1 mL, 0.57 mmol), free amine **NH$_2$-4GR^{P}A** (120 mg, 0.17 mmol) and HATU (60 mg, 0.16 mmol). The residue was purified by flash chromatography (EtOAc/MeOH, 10:1 to 1:1 gradient) to afford 137 mg (90%) of protected hexapeptide **Ac-6GR^{P}A** as an off-white solid.

Mp: 125-128 °C; $[\alpha]_D^{20}$: –1.5 (c 0.2, CHCl$_3$); **IR** ν[cm^{-1}]: 645, 700, 730, 784, 851, 1151, 1256, 1370, 1390, 1450, 1493, 1522, 1601, 1654, 1722, 2853, 2871, 2931, 2970, 3030, 3062, 3083, 3303, 3360; **^{1}H NMR** (600 MHz, CDCl$_3$) δ 8.19 (dd, *J* = 6.5, 5.2 Hz, 1H, H-10), 8.08 (dd, *J* = 7.0, 4.4 Hz, 1H, H-10'), 7.40-7.34 (m, 6H, Ar), 7.33-7.28 (m, 6H, Ar), 7.25-7.20 (m, 4H, Ar, H-7'), 7.18 (bs, 1H, H-7''), 7.13 (bs, 1H, H-7), 7.03 (t, *J* = 5.3 Hz, 1H, H-10''), 4.08-4.04 (m, 1H, H-9a'), 4.03-3.95 (m, 3H, H-12, H-12', H-12''), 4.02 (dd, *J* = 16.4, 6.6 Hz, 1H, H-9a), 3.90 (dd, *J* = 16.7, 5.5 Hz, 1H, H-9a''), 3.71 (dd, *J* = 16.7, 5.2 Hz, 1H, H-9b''), 3.63 (dd, *J* = 16.5, 5.1 Hz, 1H, H-9b), 3.53-3.48 (m, 1H, H-6a), 3.49 (dd, *J* = 16.2, 4.4 Hz, 1H, H-9b'), 3.40-3.31 (m, 2H, H-6a', H-6a''), 3.17 (bs, 1H, H-3), 3.13-2.98 (m, 5H, H-3', H-3'', H-6b, H-6b', H-6b''), 2.95-2.75 (m, 6H, H-4a, H-4a', H-4a'', H-5, H-5', H-5''), 2.26-2.14 (m, 3H, H-4b, H-4b', H-4b''), 2.00 (s, 3H, Ac), 1.99 (bs, 6H, 3NH$_2$), 1.46 (s, 9H, *t*Bu), 1.34-1.28 (m, 9H, H-13, H-13', H-13''), 0.94 (d, *J* = 6.3 Hz, 3H, H-11'), 0.91 (d, *J* = 6.3 Hz, 3H, H-11), 0.89 (d, *J* = 6.3 Hz, 3H, H-11''); **^{13}C NMR** (151 MHz, CDCl$_3$) δ 175.2 (2C, C-1', C-1''), 173.7 (C, C-1), 170.9 (C, Ac), 169.9 (C, C-8'), 169.8 (C, C-8''), 169.1 (C, C-8), 143.7 (3C, Ar, Ar', Ar'')*, 128.4(2C)/128.3 (3CH, Ar, Ar', Ar''), 127.6/127.5(2C) (3CH, Ar, Ar', Ar''), 127.0 (3CH, Ar, Ar', Ar''), 82.0 (C, *t*Bu), 67.9 (C, C-2), 66.7 (2C, C-2', C-2''), 64.6 (2CH, C-3', C-3''), 63.7 (CH, C-3)*, 55.3/54.4/54.3 (3CH, C-12, C-12', C-12'')*, 47.4/47.1/46.8 (3CH$_2$, C-4, C-4', C-4'')*, 44.1/43.6/43.2 (3CH, C-5, C-5', C-5''), 43.0 (CH$_2$, C-9''), 42.9 (CH$_2$, C-9), 42.7 (CH$_2$, C-9'), 39.5 (CH$_2$, C-6), 38.2 (2CH$_2$, C-6', C-6''), 28.1 (CH$_3$, *t*Bu), 23.2 (CH$_3$, Ac), 12.8 (CH$_3$, C-11), 12.7 (CH$_3$, C-11'), 12.6 (CH$_3$, C-11''), 11.5 (3CH$_3$, C-13, C-13', C-13'')*; * HMBC determination; **MS** (ESI+) m/z, (%): 355.6 (355.9) (75, $[M+3H]^{3+}$), 453.0 (453.3) (59, $[M+2H-C_4H_8-PhCH=CH_2]^{2+}$), 533.0 (533.3) (100, $[M+2H]^{2+}$), 801 (12, $[M+H-C_4H_8-2PhCH=CH_2]^{+}$), 1065 (15, $[M+H]^{+}$), 1087 (40, $[M+Na]^{+}$); **HRMS** (ESI+) m/z: $[M+H]^{+}$ Calcd for $C_{57}H_{85}N_{12}O_8$ 1065.6608; Found 1065.6612.

The opposite enantiomer **Ac-6GS^{P}A** (Ac(Gly-(*S,S,S,S*) ^{P}AAMP)$_3$O*t*Bu was prepared the same way, starting from diamine **NH$_2$-S^{P}A** and its spectral and physical data match those for **Ac-6GR^{P}A**.

α/γ-Peptide Ac(Gly-(*R*,*R*,*R*,*R*)AAMP)$_3$O*t*Bu (Ac-6GR^{H}A)

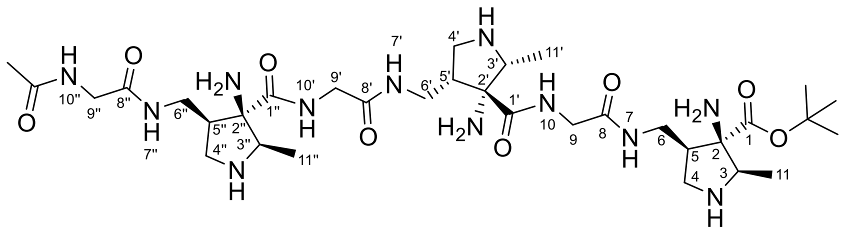

Prepared according to general **procedure D** from hexapeptide **Ac-6GR^{P}A** (21 mg, 0.02 mmol) providing 14 mg (95%) of free base **Ac-6GR^{H}A** as an off-white solid.

Mp: 157-159 °C; $[\alpha]_D^{20}$: −11.0 (c 0.2, MeOH); **IR** (MeOH, 0.025 mm CaF_2 cell) ν[cm^{-1}]: 1089, 1156, 1256, 1371, 1445, 1548, 1657, 1723; **^{1}H NMR** (600 MHz, H_2O:D_2O 9:1, CD_3COOD, pH 4.5) δ 8.71 (t, *J* = 5.7 Hz, 1H, H-10), 8.70 (t, *J* = 5.7 Hz, 1H, H-10'), 8.29 (dd, *J* = 7.6, 5.3 Hz, 1H, H-7), 8.25 (t, *J* = 6.1 Hz, 1H, H-10''), 8.17 (t, *J* = 6.4 Hz, 1H, H-7'), 8.10 (t, *J* = 6.4 Hz, 1H, H-7''), 4.05 (q, *J* = 6.9 Hz, 1H, H-3), 3.99 (q, *J* = 6.9 Hz, 1H, H-3'), 3.98 (q, *J* = 6.9 Hz, 1H, H-3''), 3.96 (dd, *J* = 16.7, 5.7 Hz, 1H, H-9a), 3.93 (dd, *J* = 16.7, 5.6 Hz, 1H, H-9a'), 3.85 (dd, *J* = 16.7, 5.7 Hz, 1H, H-9b'), 3.84 (dd, *J* = 16.6, 5.8 Hz, 1H, H-9b), 3.81 (d, *J* = 6.1 Hz, 2H, H-9''), 3.67-3.61 (m, 1H, H-4a, H-4a', H-4a''), 3.53 (ddd, *J* = 14.3, 8.8, 7.5 Hz, 1H, H-6a), 3.44 (ddd, *J* = 14.1, 7.9, 6.4 Hz, 1H, H-6a''), 3.42 (ddd, *J* = 14.1, 7.9, 6.4 Hz, 1H, H-6a'), 3.34 (ddd, *J* = 14.2, 7.0, 6.4 Hz, 1H, H-6b'), 3.30 (dt, *J* = 14.2, 6.6 Hz, 1H, H-6b''), 3.24 (dt, *J* = 14.2, 5.4 Hz, 1H, H-6b), 3.20 (dd, *J* = 12.1, 11.1 Hz, 1H, H-4b'), 3.19 (dd, *J* = 12.1, 11.1 Hz, 1H, H-4b''), 3.17-3.03 (m, 3H, H-5, H-5', H-5''), 3.13 (dd, *J* = 12.8, 11.0 Hz, 1H, H-4b), 2.05 (s, 3H, Ac), 1.49 (s, 9H, *t*Bu), 1.29 (d, *J* = 6.7 Hz, 3H, H-11'), 1.284 (d, *J* = 6.9 Hz, 3H, H-11''), 1.279 (d, *J* = 6.8 Hz, 3H, H-11); the amine NH and NH_2 resonances not detectable likely due to water presaturation; **^{13}C NMR** (151 MHz, D_2O, CD_3COOD, pH 4.5) δ 175.4 (C, Ac), 174.72 (C, C-1''), 174.68 (C, C-1'), 173.0 (C, C-1), 172.5 (C, C-8''), 171.52 (C, C-8'), 171.51 (C, C-8), 85.3 (C, *t*Bu), 66.7 (2C, C-2', C-2''), 66.3 (C, C-2), 63.59/63.57 (2CH, C-3', C-3''), 63.3 (CH, C-3), 46.6/46.53/46.50 (3CH, C-5, C-5', C-5''), 46.44/46.42/46.3 (3CH_2, C-4, C-4', C-4''), 43.3 (CH_2, C-9), 43.23 (CH_2, C-9'), 43.16 (CH_2, C-9''), 36.91/36.88/36.86 (3CH_2, C-6, C-6', C-6''), 27.7 (CH_3, *t*Bu), 22.4 (CH_3, Ac), 10.6/10.54/10.52 (CH_3, C-11, C-11', C-11''); **MS** (ESI+) m/z, (%): 349.0 (349.2) (48, $[M+2H-C_4H_8]^{2+}$), 753 (21, $[M+H]^+$), 775 (100, $[M+Na]^+$), 791 (8, $[M+K]^+$); **HRMS** (ESI+) m/z: $[M+H]^+$ Calcd for $C_{33}H_{61}N_{12}O_8$ 753.4730; Found 753.4728.

α/γ-Peptide Fmoc(Gly-(*R*,*R*,*R*,*R*)^{P}AAMP)$_4$O*t*Bu (Fmoc-8GR^{P}A)

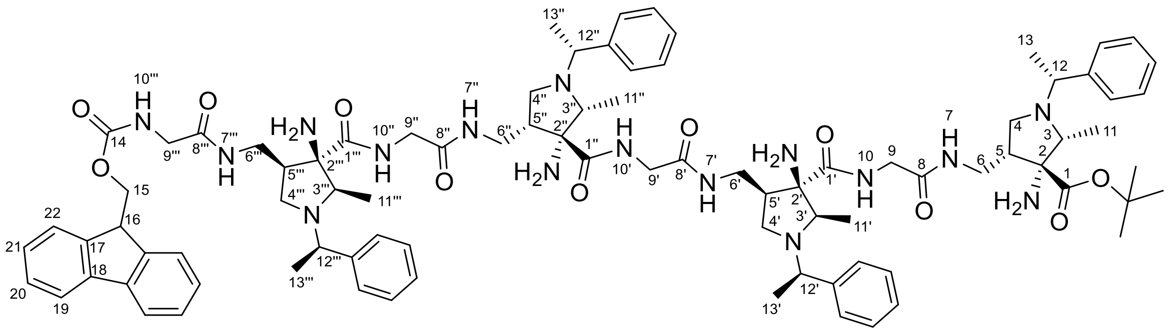

Prepared according to general **procedure B** from tetrapeptide **Fmoc-4GR^{P}A** (158 mg, 0.17 mmol), DIPEA (0.18 mL, 1.04 mmol), free amine **NH$_2$-4GR^{P}A** (140 mg, 0.20 mmol) and HATU (79 mg, 0.21 mmol). The residue was purified by flash chromatography (EtOAc/MeOH, 10:1 to 1:1 gradient) to afford 222 mg (82%) of protected octapeptide **Fmoc-8GR^{P}A** as an off-white solid.

Mp: 124-126 °C; $[\alpha]_D^{20}$: –17.5 (c 0.2, $CHCl_3$); **IR** ν[cm^{-1}]: 699, 730, 759, 784, 848, 909, 1025, 1151, 1251, 1371, 1449, 1515, 1651, 1720, 2853, 2873, 2896, 2931, 2970, 3029, 3063, 3085, 3300, 3358; **^{1}H NMR** (401 MHz, $CDCl_3$) δ 8.27 (t, *J* = 5.5 Hz, 1H, H-10), 8.20 (t, *J* = 5.2 Hz, 1H, H-10'), 8.08 (t, *J* = 5.4 Hz, 1H, H-10''), 7.74 (d, *J* = 7.5 Hz, 2H, H-19), 7.60 (d, *J* = 7.2 Hz, 2H, H-22), 7.42-7.17 (m, 26H, Ar, H-20, H-21, H-7', H-7''), 7.16 (bs, 1H, H-7'''), 7.11 (dd, *J* = 6.6, 4.4 Hz, 1H, H-7), 6.51 (bs, 1H, H-10'''), 4.38 (dd, *J* = 10.4, 7.4 Hz, 1H, H-15a), 4.32 (dd, *J* = 10.5, 7.4 Hz, 1H, H-15b), 4.21 (t, *J* = 7.2 Hz, 1H, H-16), 4.05-3.92 (m, 7H, H-12, H-12', H-12'', H-12''', H-9a, H-9a', H-9a''), 3.81 (dd, *J* = 17.0, 5.9 Hz, 1H, H-9a'''), 3.74 (dd, *J* = 17.1, 6.0 Hz, 1H, H-9b'''), 3.69 (dd, *J* = 16.4, 5.2 Hz, 1H, H-9b), 3.62-3.33 (m, 6H, H-6a, H-6a', H-6a'', H-6a''', H-9b', H-9b''), 3.24 (q, *J* = 6.1 Hz, 1H, H-3'), 3.17 (q, *J* = 6.1 Hz, 1H, H-3''), 3.15 (q, *J* = 6.0 Hz, 1H, H-3'''), 3.12-2.99 (m, 2H, H-6b, H-6b'''), 3.02 (q, *J* = 6.2 Hz, 1H, H-3), 2.99-2.71 (m, 10H, H-4a, H-4a', H-4a'', H-4a''', H-5, H-5', H-5'', H-5''', H-6b', H-6b''), 2.19-2.14 (m, 1H, H-4b), 2.10-2.00 (m, 3H, H-4b', H-4b'', H-4b'''), 1.94 (s, 8H, 4NH_2), 1.46 (s, 9H, *t*Bu), 1.32-1.21 (m, 12H, H-13, H-13', H-13'', H-13'''), 0.98 (d, *J* = 6.2 Hz, 3H, H-11'), 0.93 (d, *J* = 6.2 Hz, 3H, H-11''), 0.90 (d, *J* = 6.2 Hz, 3H, H-11), 0.88 (d, *J* = 6.3 Hz, 3H, H-11'''); **^{13}C NMR** (101 MHz, $CDCl_3$) δ 175.5 (C, C-1'), 175.3 (2C, C-1'', C-1'''), 173.7 (C, C-1), 169.93/169.88/169.86 (3C, C-8', C-8'', C-8'''), 169.1 (C, C-8), 156.9 (C, C-14), 144.01/143.96 (C, C-17), 143.8 (4C, Ar, Ar', Ar'', Ar'''), 141.3 (C, C-18), 128.23/128.21/128.19/128.15 (4CH, Ar, Ar', Ar'', Ar'''), 127.8 (CH, C-20), 127.51/127.50/127.47/127.4 (4CH, Ar, Ar', Ar'', Ar'''), 127.17/127.16 (CH, C-21), 126.81/126.76/126.74/126.70 (4CH, Ar, Ar', Ar'', Ar'''), 125.3/125.2 (CH, C-22), 120.0 (CH, C-19), 81.7 (C, *t*Bu), 67.7 (C, C-2), 67.2 (CH_2, C-15), 66.6 (2C, C-2'', C-2'''), 66.5 (C, C-2'), 64.4 (CH, C-3), 63.6/63.5 (2CH, C-3'', C-3'''), 63.4 (CH, C-3'), 55.0 (CH, C-12), 54.6/54.3/54.2 (3CH, C-12', C-12'', C-12'''), 47.22 (CH_2, C-4), 47.17 (CH, C-16), 46.8/46.6/46.5 (3CH_2, C-4', C-4'', C-4'''), 44.3

(CH_2, C-9'''), 44.0/43.8/43.7 (3CH, C-5', C-5'', C-5'''), 43.2 (CH, C-5), 43.0 (CH_2, C-9), 42.7 ($2CH_2$, C-9', C-9''), 39.5 (CH_2, C-6), 38.2 ($3CH_2$, C-6', C-6'', C-6'''), 28.1 (CH_3, *t*Bu), 12.8/12.71/12.68/12.66 ($4CH_3$, C-11, C-11', C-11'', C-11'''), 11.4 (CH_3, C-13), 10.6/10.3/10.2 ($3CH_3$, C-13', C-13'', C-13'''); **MS** (ESI+) m/z, (%): 391.0 (391.2) (5, $[M+4H]^{4+}$), 521.0 (521.3) (87, $[M+3H]^{3+}$), 781.0 (781.5) (100, $[M+2H]^{2+}$), 792.0 (792.5) (23, $[M+H+Na]^{2+}$), 1561 (18, $[M+H]^{+}$), 1583 (10, $[M+Na]^{+}$); **HRMS** (ESI+) m/z: $[M+2H]^{2+}$ Calcd for $C_{87}H_{118}N_{16}O_{11}$ 781.4578; Found 781.4570.

α/γ-Peptide NH_2(Gly-(*R*,*R*,*R*,*R*)^{P}AAMP)$_4$O*t*Bu (NH_2-8GR^{P}A)

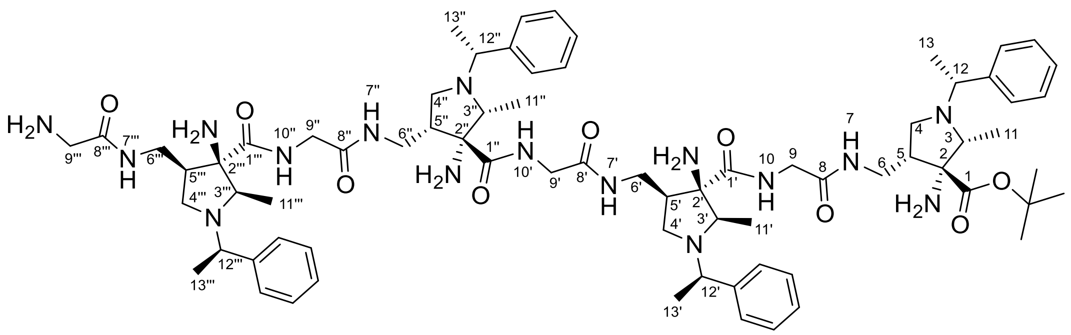

Prepared according to general **procedure C** from protected dipeptide **Fmoc-8GR^{P}A** (234 mg, 0.15 mmol). The residue was purified by flash chromatography ($CHCl_3$/MeOH + 1% TEA, neat to 5:1 gradient) to afford 169 mg (85%) of free amine **NH_2-8GR^{P}A** as an off-white solid.

Mp: 125-127 °C; $[\alpha]_D^{20}$: –9.0 (c 0.2, $CHCl_3$); **IR** ν[cm^{-1}]: 666, 700, 753, 784, 846, 974, 1025, 1153, 1216, 1259, 1372, 1452, 1521, 1602, 1651, 1720, 2854, 2871, 2929, 2969, 3029, 3062, 3083, 3293, 3363; **^{1}H NMR** (401 MHz, $CDCl_3$) δ 8.18 (dd, *J* = 5.6, 4.8 Hz, 1H, H-10), 8.16-8.11 (m, 2H, H-10', H-10''), 7.58-7.47 (m, 2H, H-7', H-7''), 7.38-7.27 (m, 17H, Ar, H-7'''), 7.25-7.17 (m, 4H, Ar), 7.09 (t, *J* = 5.4 Hz, 1H, H-7), 4.19-3.97 (m, 6H, H-12', H-12'', H-12''', H-9a, H-9a', H-9a''), 3.96 (q, *J* = 6.7 Hz, 1H, H-12), 3.69 (dd, *J* = 16.6, 4.6 Hz, 1H, H-9b), 3.55-3.41 (m, 6H, H-6a, H-6a', H-6a'', H-6a''', H-9b', H-9b''), 3.22 (q, *J* = 6.3 Hz, 1H, H-3'), 3.17-3.07 (m, 5H, H-3'', H-3''', H-6b, H-9'''), 3.03 (q, *J* = 6.2 Hz, 1H, H-3), 2.99-2.71 (m, 11H, H-4a, H-4a', H-4a'', H-4a''', H-5, H-5', H-5'', H-5''', H-6b', H-6b'', H-6b'''), 2.21-2.15 (m, 1H, H-4b), 2.09-2.00 (m, 3H, H-4b', H-4b'', H-4b'''), 1.86 (s, 10H, $5NH_2$), 1.47 (s, 9H, *t*Bu), 1.31-1.26 (m, 12H, H-13, H-13', H-13'', H-13'''), 0.98 (d, *J* = 6.2 Hz, 3H, H-11'), 0.96 (d, *J* = 6.3 Hz, 3H, H-11''), 0.94 (d, *J* = 6.3 Hz, 3H, H-11'''), 0.91 (d, *J* = 6.2 Hz, 3H, H-11); **^{13}C NMR** (101 MHz, $CDCl_3$) δ 175.4 (C, C-1'), 175.2/175.0 (2C, C-1'', C-1'''), 173.8 (C, C-1), 170.4/170.14/170.08 (3C, C-8', C-8'', C-8'''), 169.1 (C, C-8), 144.1/143.93/143.90/143.8 (4C, Ar, Ar', Ar'', Ar'''), 128.3/128.24/128.23/128.21 (4CH, Ar, Ar', Ar'', Ar'''), 127.60/127.58/127.57/127.5 (4CH, Ar, Ar', Ar'', Ar'''), 126.84/126.76/126.74/126.71 (4CH, Ar, Ar', Ar'', Ar'''), 81.8 (C, *t*Bu), 67.8 (C, C-2), 66.74/66.67/66.6 (3C, C-2', C-2'', C-2'''), 64.4 (CH, C-3), 63.6

(2CH, C-3'', C-3'''), 63.5 (CH, C-3'), 55.0 (CH, C-12), 54.3/54.0/53.9 (3CH, C-12', C-12'', C-12'''), 47.3 (CH_2, C-4), 46.5/46.4/46.1 (3CH_2, C-4', C-4'', C-4'''), 44.4 (CH_2, C-9'''), 44.2/44.03/44.00 (3CH, C-5', C-5'', C-5'''), 43.3 (CH, C-5), 43.0 (CH_2, C-9), 42.7/42.6 (2CH_2, C-9', C-9''), 39.5 (CH_2, C-6), 38.1/38.0/37.6 (3CH_2, C-6', C-6'', C-6'''), 28.1 (CH_3, *t*Bu), 12.87/12.86/12.8/12.7 (4CH_3, C-11, C-11', C-11'', C-11'''), 11.4 (CH_3, C-13), 10.4/10.0/9.8 (3CH_3, C-13', C-13'', C-13'''); **MS** (ESI+) m/z, (%): 428.3 (428.6) (11, $[M+3H-C_4H_8]^{3+}$), 447.0 (447.3) (100, $[M+3H]^{3+}$), 590 (590.4) (13, $[M+2H-C_4H_8-PhCH=CH_2]^{2+}$), 670.0 (670.4) (24, $[M+2H]^{2+}$), 1361 (11, $[M+Na]^{+}$); **HRMS** (ESI+) m/z: $[M+H+Na]^+$ Calcd for $C_{72}H_{106}N_{16}O_9Na$ 1361.8221; Found 1361.8215.

α/γ-Peptide Boc(Gly-(*R*,*R*,*R*,*R*)^{P}AAMP)$_4$O*t*Bu (Boc-8GR^{P}A)

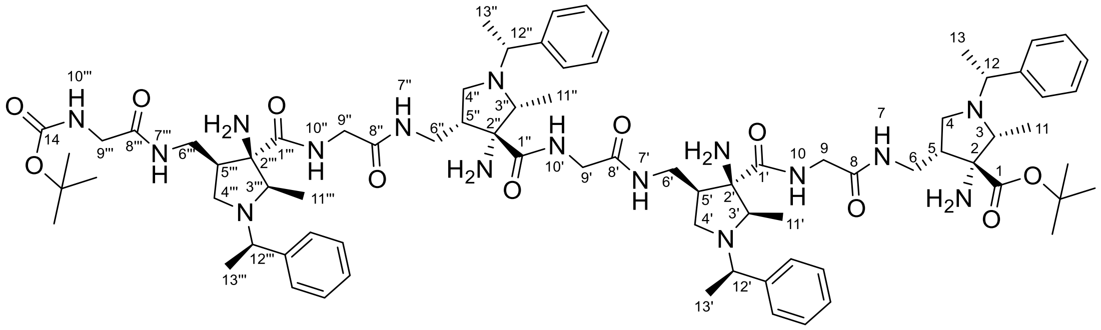

Prepared according to general **procedure E** from free amine **NH_2-8GR^{P}A** (100 mg, 0.075 mmol). The residue was purified by flash chromatography (EtOAc/MeOH, 10:1 to 1:1 gradient) to afford 89 mg (83%) of protected peptide **Boc-8GR^{P}A** as an off-white solid.

Mp: 93-95 °C; $[\alpha]_D^{20}$**:** –18.5 (c 0.2, $CHCl_3$); **IR** ν[cm^{-1}]: 700, 752, 784, 859, 979, 1026, 1156, 1252, 1369, 1452, 1518, 1653, 1719, 2854, 2873, 2929, 2970, 3029, 3063, 3302, 3350; **^{1}H NMR** (600 MHz, $CDCl_3$) δ 8.25 (s, 1H, H-10), 8.19 (s, 1H, H-10'), 8.06 (t, *J* = 5.5 Hz, 1H, H-10''), 7.39 (bs, 2H, H-7', H-7''), 7.37-7.27 (m, 16H, Ar), 7.24-7.19 (m, 4H, Ar), 7.08 (dd, 6.1, 4.9 Hz, 1H, H-7), 7.01 (bs, 1H, H-7'''), 5.76 (bs, 1H, H-10'''), 4.06 (dd, *J* = 16.7, 7.1 Hz, 1H, H-9a'), 4.05-3.97 (m, 5H, H-12', H-12'', H-12''', H-9a, H-9a''), 3.96 (q, *J* = 6.7 Hz, 1H, H-12), 3.75 (dd, *J* = 16.5, 5.6 Hz, 1H, H-9a'''), 3.67 (dd, *J* = 16.4, 5.2 Hz, 1H, H-9b), 3.59 (dd, *J* = 16.9, 5.5 Hz, 1H, H-9b'''), 3.55-3.42 (m, 4H, H-6a, H-6a', H-6a'', H-9b''), 3.52 (dd, *J* = 16.5, 4.7 Hz, 1H, H-9b'), 3.39-3.31 (m, 1H, H-6a'''), 3.25 (q, *J* = 6.3 Hz, 1H, H-3'), 3.19 (q, *J* = 6.3 Hz, 1H, H-3''), 3.16 (q, *J* = 6.3 Hz, 1H, H-3'''), 3.08 (ddd, *J* = 13.8, 9.3, 4.8 Hz, 1H, H-6b), 3.03 (q, *J* = 6.2 Hz, 1H, H-3), 3.03-2.96 (m, 1H, H-6b'''), 2.96-2.73 (m, 10H, H-4a, H-4a', H-4a'', H-4a''', H-5, H-5', H-5'', H-5''', H-6b', H-6b''), 2.18 (dd, *J* = 8.2, 5.0 Hz, 1H, H-4b), 2.05 (bs, 3H, H-4b', H-4b'', H-4b'''), 1.86 (s, 8H, 4NH_2), 1.47 (s, 9H, *t*Bu), 1.40 (s, 9H, Boc), 1.30 (d, *J* = 6.7 Hz, 3H, H-13), 1.30-1.27 (m, 9H, H-13', H-13'', H-13'''), 0.98 (d, *J* = 6.2 Hz, 3H, H-11'), 0.91 (d, *J* = 6.2 Hz, 6H, H-11'', H-11'''), 0.90 (d, *J* = 6.2 Hz,

3H, H-11); **^{13}C NMR** (151 MHz, $CDCl_3$) δ 175.5 (C, C-1'), 175.4 (2C, C-1'', C-1'''), 173.7 (C, C-1), 170.4 (C, C-8'''), 170.04 (C, C-8''), 169.96 (C, C-8'), 169.2 (C, C-8), 156.3 (C, C-14), 144.1 (C, Ar), 143.9/143.8/143.7 (3C, Ar', Ar'', Ar'''), 128.29 (CH, Ar), 128.26 (2CH, Ar), 128.2 (CH, Ar), 127.58 (2CH, Ar), 127.56 (CH, Ar), 127.5 (CH, Ar), 126.9 (2CH, Ar), 126.80 (CH, Ar), 126.77 (CH, Ar), 81.8 (C, *t*Bu), 79.9 (C, Boc), 67.8 (C, C-2), 66.71 (C, C-2''), 66.68 (C, C-2'), 66.6 (C, C-2'''), 64.5 (CH, C-3), 63.6 (CH, C-3'''), 63.4 (2CH, C-3', C-3''), 55.1 (CH, C-12), 54.3 (3CH, C-12', C-12'', C-12'''), 47.3 (CH_2, C-4), 46.8/46.5/46.0 ($3CH_2$, C-4', C-4'', C-4'''), 44.0/43.8/43.7 (3CH, C-5', C-5'', C-5'''), 43.9 (CH_2, C-9'''), 43.2 (CH, C-5), 43.1 (CH_2, C-9), 42.8 (CH_2, C-9'), 42.7 (CH_2, C-9''), 39.6 (CH_2, C-6), 38.3 ($3CH_2$, C-6', C-6'', C-6'''), 28.5 (CH_3, Boc), 28.1 (CH_3, *t*Bu), 12.83/12.76/12.71 ($3CH_3$, C-11, C-11'', C-11'''), 12.68 (CH_3, C-11'), 11.5 (CH_3, C-13), 10.4/10.3/10.1 ($3CH_3$, C-13', C-13'', C-13'''); **MS** (ESI+) m/z, (%): 461.6 (461.9) (11, $[M+3H-C_4H_8]^{3+}$), 480.3 (480.6) (90, $[M+3H]^{3+}$), 720.0 (720.4) (100, $[M+2H]^{2+}$), 731.0 (731.4) (25, $[M+H+Na]^{2+}$), 1439 (22, $[M+H]^{+}$), 1461 (19, $[M+Na]^{+}$); **HRMS** (ESI+) m/z: $[M+2H]^{2+}$ Calcd for $C_{77}H_{116}N_{16}O_{11}$ 720.4499; Found 720.4496.

α/γ-Peptide Boc(Gly-(*R*,*R*,*R*,*R*)AAMP)$_4$O*t*Bu (Boc-8GR^{H}A)

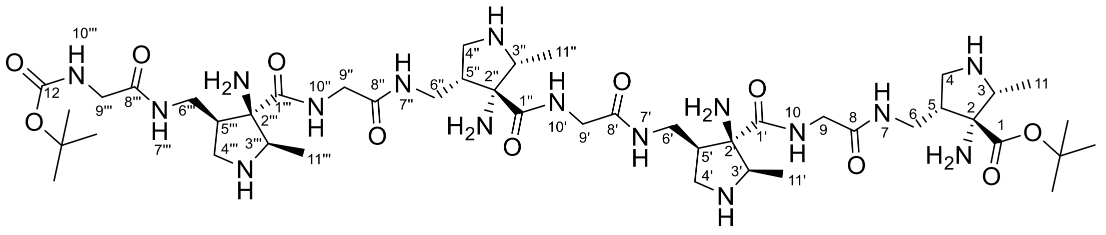

Prepared according to general **procedure D** from octapeptide **Boc-8GR^{P}A** (29 mg, 0.02 mmol) providing 19 mg (95%) of free base **Boc-8GR^{H}A** as an off-white amorphous solid.

Mp: 150-152 °C; **$[\alpha]_D^{20}$:** –5.5 (c 0.2, $CHCl_3$); **IR** ν[cm^{-1}]: 848, 968, 1031, 1162, 1253, 1368, 1392, 1409, 1453, 1521, 1659, 1721, 2876, 2932, 2972, 3069, 3296; ^{1}H NMR (600 MHz, H_2O:D_2O 9:1, CD_3COOD, pH 4.5) δ 8.79-8.68 (m, 3H, H-10, H-10', H-10''), 8.32 (dd, *J* = 7.4, 5.3 Hz, 1H, H-7), 8.20-8.09 (m, 3H, H-7', H-7'', H-7'''), 6.84 (s, 1H, H-10'''), 4.10 (q, *J* = 7.0 Hz, 1H, H-3), 4.03-3.83 (m, 9H, H-3', H-3'', H-3''', H-9, H-9', H-9''), 3.72-3.62 (m, 6H, H-4a, H-4a', H-4a'', H-4a''', H-9'''), 3.59-3.50 (m, 1H, H-6a), 3.48-3.31 (m, 6H, H-6', H-6'', H-6'''), 3.30-3.24 (m, 1H, H-6b), 3.24-3.01 (m, 8H, H-4b, H-4b', H-4b'', H-4b''', H-5, H-5', H-5'', H-5'''), 1.50 (s, 9H, *t*Bu), 1.44 (s, 9H, Boc), 1.32-1.29 (m, 12H, H-11, H-11', H-11'', H-11'''); the amine NH and NH_2 resonances not detectable likely due to water presaturation; **^{13}C NMR** (151 MHz, D_2O, CD_3COOD, pH 4.5) δ 174.52/174.48/174.4 (3C, C-1', C-1'', C-1'''), 173.32 (C, C-1), 172.26 (C, C-8'''), 171.5/171.42/171.38 (3C, C-8, C-8'', C-8'''), 158.4 (C, C-12), 85.5 (C, *t*Bu), 82.1 (C, Boc), 66.7/66.64/66.61 (3C, C-2', C-2'', C-2'''), 66.3 (C, C-2), 63.50/63.49/63.46 (3CH, C-3', C-3'', C-3'''),

63.1 (CH, C-3), 46.54/46.50/46.46 ($3CH_2$, C-4', C-4'', C-4'''), 46.45/46.43(2C)/46.40 (4CH, C-5, C-5', C-5'', C-5'''), 46.3 (CH_2, C-4), 43.9 (CH_2, C-9'''), 43.19/43.17/43.1 ($3CH_2$, C-9, C-9', C-9''), 37.03/37.01/36.94/36.87 ($4CH_2$, C-6, C-6', C-6'', C-6'''), 28.2 (CH_3, Boc), 27.6 (CH_3, *t*Bu), 10.6/10.48/10.47/10.46 ($4CH_3$, C-11, C-11', C-11'', C-11'''); **MS** (ESI+) m/z, (%): 289.7 (289.8) (26, $[M+3H-2C_4H_8-CO_2]^{3+}$), 434.0 (434.3) (27, $[M+2H-2C_4H_8-CO_2]^{2+}$), 512.0 (512.3) (6, $[M+2H]^{2+}$), 1023 (6, $[M+H]^+$), 1045 (100, $[M+Na]^+$); **HRMS** (ESI+) m/z: $[M+Na]^+$ Calcd for $C_{45}H_{82}N_{16}O_{11}Na$ 1045.6241; Found 1045.6238.

α/γ-Peptide Ac(Gly-L-Dab)$_3$NH$_2$ (Ac-6GSDab)

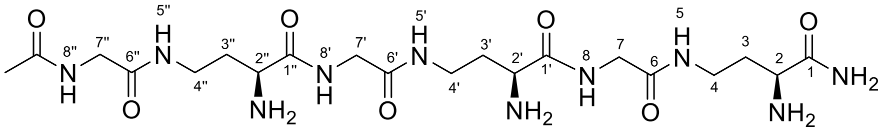

Hexapeptide **Ac-6GSDab** was prepared on Rink Amide AM resin (200-400 mesh, 0.61 mmol/g) (150 mg, 0.092 mmol) using the Fmoc solid phase strategy. Sequential steps couplings of Fmoc-Gly-OH (109 mg, 0.366 mmol) and Boc-L-Dab(Fmoc)-OH (161 mg, 0.366 mmol) were performed in three cycles using an automatic synthesizer and each peptide coupling step was carried out in two 60 min stages, for the first using DIC (92 mg, 0.366 mmol), for the second using HBTU (138 mg, 0.366 mmol) and DIPEA (127 µL, 0.732 mmol). After the coupling was complete, the resin was capped by two treatments with 5% acetic anhydride (v/v, 250 µL) and 2.5% DIPEA (v/v 125 µL) in NMP/DCM (1:1, 5 mL) each lasting for 15 min. The resin was washed with NMP/DCM (1:1, 3x5mL) and dried. The peptide was cleaved from the resin with a mixture of TFA/H_2O/triisopropylsilane (95:2.5:2.5, 5 mL) for 3 h followed by filtration of the residual resin that was washed with another portion of TFA (5 mL). The combined filtrates were concentrated *in vacuo*, redissolved in water, and neutralized through a plug of DOWEX® 1X8 chloride form, which was made basic by 0.1 M solution of NaOH before, to yield 23 mg (37%) of the unprotected basic peptide **Ac-6GSDab** as an off-white amorphous solid.

$[\alpha]_D^{20}$**:** +14.3 (c 0.1, $CHCl_3$); **IR** ν[cm^{-1}]: 1028, 1126, 1253, 1283, 1373, 1409, 1437, 1549, 1658, 2852, 2926, 3076, 3284; **^{1}H NMR** (600 MHz, H_2O:D_2O 9:1, CD_3COOD, pH 4.5) δ 8.88-8.83 (m, 2H, H-8, H-8'), 8.35 (t, *J* = 6.2 Hz, 1H, H-8''), 8.30-8.20 (m, 3H, H-5, H-5', H-5''), 7.95 (s, 1H, $CONH_2$), 7.37 (s, 1H, $CONH_2$), 4.87 (bs, 6H, $3NH_2$), 4.08-3.96 (m, 7H, H-2, H-2', H-2'', H-7, H-7'), 3.88 (d, *J* = 5.8 Hz, 2H, H-7''), 3.46-3.34 (m, 6H, H-4, H-4', H-4''), 2.17-2.04 (m, 6H, H-3, H-3', H-3''), 2.06 (s, 3H, Ac); **^{13}C NMR** (151 MHz, H_2O:D_2O 9:1, CD_3COOD, pH 4.5) δ 175.8 (C, C-1), 173.1 (C, Ac), 172.4 (C, C-6''), 172.1/172.0 (2C, C-1', C-1''), 170.81/170.76 (2C, C-6, C-6'), 51.9/51.8/51.7 (3CH, C-2, C-2', C-2''), 43.5/43.20/43.16

(CH_2, C-7, C-7', C-7''), 35.8/35.6(2C) (3CH_2, C-4, C-4', C-4''), 31.5/31.3/31.2 (CH_2, C-3, C-3', C-3''), 22.4 (CH_3, Ac); **MS** (ESI+) m/z, (%): 266.0 (266.2) (20, $[M+2H]^{2+}$), 531 (37, $[M+H]^+$), 553 (100, $[M+Na]^+$); **HRMS** (ESI+) m/z: $[M+H]^+$ Calcd for $C_{20}H_{39}N_{10}O_7$ 531.2998; Found 531.2994.

α/γ-Peptide Fmoc(Ala-(*S*,*S*,*S*,*S*)^{P}AAMP)O*t*Bu (Fmoc-2AS^{P}A)

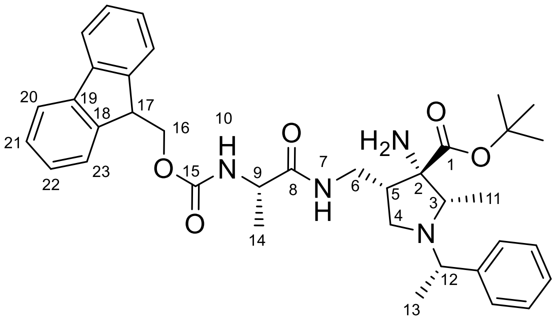

Prepared according to general procedure A from diamine **NH_2-S^{P}A** (400 mg, 1.20 mmol) and Fmoc-Ala-OH (311 mg, 1 mmol). The residue was purified by flash chromatography (cyclohexane/EtOAc, 1:1 to neat EtOAc gradient) to afford 584 mg (93%) of protected dipeptide **Fmoc-2AS^{P}A** as an off-white solid.

Mp: 128-130 °C; $[\alpha]_D^{20}$: +23.9 (c 0.2, $CHCl_3$); **IR** ν[cm^{-1}]: 646, 700, 737, 759, 784, 846, 910, 1078, 1108, 1147, 1246, 1287, 1319, 1369, 1390, 1450, 1478, 1492, 1519, 1601, 1661, 1720, 2873, 2931, 2973, 3028, 3064, 3294, 3386; **^{1}H NMR** (401 MHz, $CDCl_3$) δ 7.76 (dt, *J* = 7.6, 1.0 Hz, 2H, H-20), 7.60 (d, *J* = 7.5 Hz, 2H, H-23), 7.42-7.37 (m, 2H, H-21), 7.34-7.27 (m, 6H, Ar, H-22), 7.24 (t, *J* = 5.6 Hz, 1H, H-7), 7.22-7.17 (m, 1H, Ar), 5.50 (d, *J* = 7.1 Hz, 1H, H-10), 4.38 (d, *J* = 7.1 Hz, 2H, H-16), 4.22 (t, *J* = 7.0 Hz, 1H, H-17), 4.13 (quint, *J* = 6.7 Hz, 1H, H-9), 3.94 (q, *J* = 6.7 Hz, 1H, H-12), 3.47-3.39 (m, 1H, H-6a), 3.23-3.14 (m, 1H, H-6b), 2.99 (q, *J* = 6.2 Hz, 1H, H-3), 2.77-2.68 (m, 2H, H-4a, H-5), 2.28-2.20 (m, 1H, H-4b), 2.03 (s, 2H, NH_2), 1.47 (s, 9H, *t*Bu), 1.31 (d, *J* = 6.5 Hz, 3H, H-14), 1.29 (d, *J* = 6.8 Hz, 3H, H-13), 0.90 (d, *J* = 6.2 Hz, 3H, H-11); **^{13}C NMR** (101 MHz, $CDCl_3$) δ 173.4 (C, C-1), 171.9 (C, C-8), 155.8 (C, C-15), 144.1/144.0 (C, C-18), 143.8 (C, Ar), 141.4 (C, C-19), 128.3 (CH, Ar), 127.8 (CH, C-21), 127.5 (CH, Ar), 127.2 (CH, C-22), 126.9 (CH, Ar), 125.2 (CH, C-23), 120.1 (CH, C-20), 82.0 (C, *t*Bu), 68.2 (C, C-2), 67.0 (CH_2, C-16), 64.8 (CH, C-3), 55.3 (CH, C-12), 50.6 (CH, C-9), 47.3 (CH, C-17), 47.2 (CH_2, C-4), 42.8 (CH, C-5), 39.3 (CH_2, C-6), 28.1 (CH_3, *t*Bu), 19.3 (CH_3, C-14), 12.7 (CH_3, C-11), 11.6 (CH_3, C-13); **MS** (ESI+) m/z, (%): 627 (100, $[M+H]^+$), 649 (5, $[M+Na]^+$); **HRMS** (ESI+) m/z: $[M+H]^+$ Calcd for $C_{37}H_{47}N_4O_5$ 627.3541; Found 627.3542.

α/γ-Peptide NH_2(Ala-(*S*,*S*,*S*,*S*)^{P}AAMP)O*t*Bu (NH_2-2AS^{P}A)

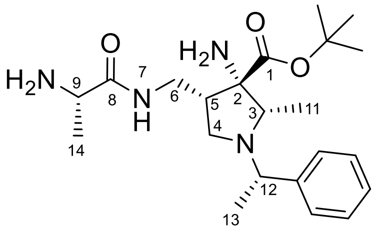

Prepared according to general **procedure C** from protected dipeptide **Fmoc-2AS^{P}A** (451 mg, 0.72 mmol). The residue was purified by flash chromatography ($CHCl_3$/MeOH + 1% TEA, neat to 25:1 gradient) to afford 285 mg (98%) of free amine **NH_2-2AS^{P}A** as a pale yellow oil.

$[\alpha]_D^{20}$: +26.0 (c 0.2, $CHCl_3$); **IR** ν[cm^{-1}]: 701, 757, 785, 801, 848, 1148, 1252, 1368, 1388, 1453, 1478, 1491, 1520, 1602, 1657, 1721, 2873, 2930, 2972, 3028, 3060, 3304, 3371; **^{1}H NMR** (401 MHz, $CDCl_3$) δ 7.71 (dd, *J* = 7.1, 5.4 Hz, 1H, H-7), 7.37-7.26 (m, 4H, Ar), 7.23-7.18 (m, 1H, Ar), 3.95 (q, *J* = 6.7 Hz, 1H, H-12), 3.45 (ddd, *J* = 13.5, 7.3, 5.0 Hz, 1H, H-6a), 3.36 (q, *J* = 6.9 Hz, 1H, H-9), 3.06 (ddd, *J* = 13.5, 9.5, 5.2 Hz, 1H, H-6b), 3.01 (q, *J* = 6.2 Hz, 1H, H-3), 2.80 (dddd, *J* = 10.0, 9.6, 5.7, 5.0 Hz, 1H, H-5), 2.73 (dd, *J* = 10.1, 8.9 Hz, 1H, H-4a), 2.15 (dd, *J* = 8.9, 5.9 Hz, 1H, H-4b), 1.65 (s, 4H, $2NH_2$), 1.45 (s, 9H, *t*Bu), 1.29 (d, *J* = 6.7 Hz, 3H, H-13), 1.23 (d, *J* = 6.9 Hz, 3H, H-14), 0.88 (d, *J* = 6.2 Hz, 3H, H-11); **^{13}C NMR** (101 MHz, $CDCl_3$) δ 175.5 (C, C-8), 173.8 (C, C-1), 144.1 (C, Ar), 128.2 (CH, Ar), 127.5 (CH, Ar), 126.8 (CH, Ar), 81.6 (C, *t*Bu), 67.9 (C, C-2), 64.6 (CH, C-3), 55.0 (CH, C-12), 51.0 (CH, C-9), 47.1 (CH_2, C-4), 43.3 (CH, C-5), 38.8 (CH_2, C-6), 28.1 (CH_3, *t*Bu), 21.7 (CH_3, C-14), 12.6 (CH_3, C-11), 11.4 (CH_3, C-13); **MS** (ESI+) m/z, (%): 405 (100, $[M+H]^+$), 427 (4, $[M+Na]^+$); **HRMS** (ESI+) m/z: $[M+H]^+$ Calcd for $C_{22}H_{37}N_4O_3$ 405.2860; Found 405.2861.

α/γ-Peptide Ac(Ala-(*S*,*S*,*S*,*S*)^{P}AAMP)O*t*Bu (Ac-2AS^{P}A)

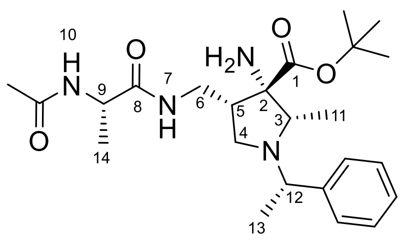

Prepared according to general **procedure A** from diamine **NH_2-S^{P}A** (400 mg, 1.20 mmol) and Ac-Ala-OH (131 mg, 1 mmol). The residue was purified by flash chromatography (cyclohexane/EtOAc, 1:1, gradient to neat EtOAc) to afford 425 mg (95%) of protected dipeptide **Ac-2AS^{P}A** as a pale yellow oil.

$[\alpha]_D^{20}$: +43.7 (c 0.2, $CHCl_3$); **IR** ν[cm^{-1}]: 700, 732, 784, 846, 912, 975, 1025, 1149, 1251, 1369, 1390, 1452, 1495, 1536, 1600, 1648, 1722, 2873, 2932, 2974, 3030, 3063, 3084, 3289; **^{1}H NMR** (401 MHz, $CDCl_3$) δ 7.36-7.27 (m, 4H, Ar), 7.23-7.18 (m, 2H, Ar, H-7), 6.38 (d, *J* = 7.4 Hz, 1H, H-10), 4.32 (quint, *J* = 7.0 Hz, 1H, H-9), 3.94 (q, *J* = 6.8 Hz, 1H, H-12), 3.43-3.36 (m, 1H, H-6a), 3.18-3.10 (m, 1H, H-6b), 2.99 (q, *J* = 6.2 Hz, 1H, H-3), 2.73-2.66 (m, 2H, H-4a, H-5), 2.23-2.15 (m, 1H, H-4b), 1.94 (s, 3H, Ac), 1.86 (s, 2H, NH_2), 1.45 (s, 9H, *t*Bu), 1.29 (d, *J* = 6.6 Hz, 3H, H-13), 1.27 (d, *J* = 6.8 Hz, 3H, H-14), 0.90 (d, *J* = 6.3 Hz, 3H, H-11); **^{13}C NMR** (101 MHz, $CDCl_3$) δ 173.4 (C, C-1), 172.1 (C, C-8), 169.8 (C, Ac), 144.0 (C, Ar), 128.3 (CH, Ar), 127.4 (CH, Ar), 126.9 (CH, Ar), 82.1 (C, *t*Bu), 68.1 (C, C-2), 64.7 (CH, C-3), 55.0 (CH, C-12), 49.0 (CH, C-9), 47.2 (CH_2, C-4), 42.9 (CH, C-5), 39.2 (CH_2, C-6), 28.1 (CH_3, *t*Bu), 23.3 (CH_3, Ac), 19.0 (CH_3, C-14), 12.7 (CH_3, C-11), 11.4 (CH_3, C-13); **MS** (ESI+) m/z, (%): 287 (11, $[M+H-C_4H_8-PhCH=CH_2]^+$), 391 (37, $[M+H-C_4H_8]^+$), 447 (100, $[M+H]^+$), 469 (14, $[M+Na]^+$); **HRMS** (ESI+) m/z: $[M+H]^+$ Calcd for $C_{24}H_{39}N_4O_4$ 447.2966; Found 447.2962.

α/γ-Peptide Fmoc(Ala-(*S*,*S*,*S*,*S*)^{P}AAMP)$_2$O*t*Bu (Fmoc-4AS^{P}A)

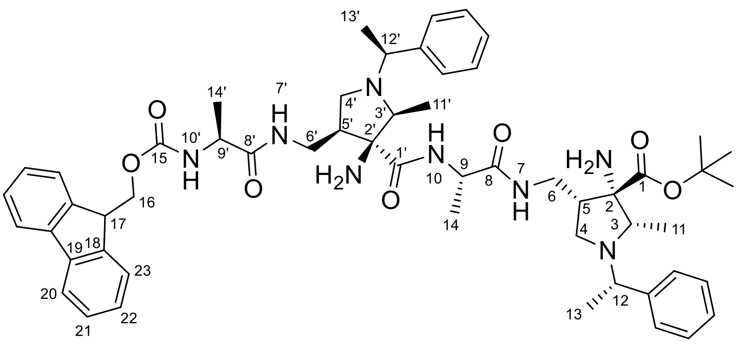

Prepared according to general **procedure B** from dipeptide **Fmoc-2AS^{P}A** (376 mg, 0.60 mmol)**,** DIPEA (0.42 mL, 2.40 mmol), free amine **NH_2-2AS^{P}A** (405 mg, 0.72 mmol) and HATU (273 mg, 0.72 mmol). The residue was purified by flash chromatography (EtOAc/MeOH, 10:1 to 2:1 gradient) to afford 532 mg (93%) of protected tetrapeptide **Fmoc-4AS^{P}A** as an off-white solid.

Mp: 99-101 °C; $[\alpha]_D^{20}$: +22.7 (c 0.2, $CHCl_3$); **IR** ν[cm^{-1}]: 664, 700, 742, 757, 784, 846, 875, 978, 1033, 1057, 1079, 1149, 1248, 1322, 1370, 1390, 1450, 1495, 1509, 1601, 1655, 1722, 2854, 2873, 2931, 2973, 3028, 3062, 3301; **^{1}H NMR** (400 MHz, $CDCl_3$) δ 7.86 (d, *J* = 6.7 Hz, 1H, H-10), 7.77 (d, *J* = 7.7 Hz, 2H, H-20), 7.62-7.55 (m, 2H, H-23), 7.42-7.27 (m, 9H, Ar, H-21, H-22, H-7), 7.25-7.16 (m, 4H, Ar), 7.13-7.02 (m, 2H, Ar), 6.77 (s, 1H, H-7'), 6.61 (d, *J* = 6.9 Hz, 1H, H-10'), 4.32-4.24 (m, 2H, H-9', H-16a), 4.22-4.14 (m, 3H, H-9, H-16b, H-17), 4.00 (q, *J* = 6.6 Hz, 1H, H-12'), 3.84 (q, *J* = 6.6 Hz, 1H, H-12), 3.66-3.58 (m, 1H, H-6a), 3.47-3.37 (m, 1H, H-6a'), 3.17 (q, *J* = 6.4 Hz, 1H, H-3'), 3.03-2.87 (m, 4H, H-3, H-5', H-6b, H-6b'), 2.81 (t, *J* = 9.6 Hz, 1H, H-4a'), 2.65 (tt, *J* = 9.6, 5.3 Hz, 1H, H-5), 2.48 (t, *J* = 9.5 Hz, 1H, H-4a),

2.10-1.96 (m, 2H, H-4b, H-4b'), 1.67 (s, 4H, $2NH_2$), 1.46 (s, 9H, *t*Bu), 1.37 (d, *J* = 6.9 Hz, 3H, H-14'), 1.31 (d, *J* = 7.1 Hz, 3H, H-14), 1.29 (d, *J* = 6.5 Hz, 3H, H-13'), 1.17 (d, *J* = 6.3 Hz, 3H, H-13), 0.94 (d, *J* = 6.3 Hz, 3H, H-11'), 0.85 (d, *J* = 6.2 Hz, 3H, H-11); **^{13}C NMR** (126 MHz, $CDCl_3$) δ 174.9 (C, C-1'), 173.7 (C, C-1), 173.3 (C, C-8'), 172.5 (C, C-8), 156.4 (C, C-15), 144.13/144.08 (C, C-18), 143.8 (C, Ar), 143.7 (C, Ar), 141.39/141.36 (C, C-19), 128.3 (CH, Ar), 128.2 (CH, Ar), 127.8 (CH, C-21), 127.6 (CH, Ar), 127.3 (CH, Ar), 127.2 (CH, C-22), 126.9 (CH, Ar), 126.8 (CH, Ar), 125.4 (CH, C-23), 120.0 (CH, C-20), 81.9 (C, *t*Bu), 67.9 (C, C-2), 67.1 (CH_2, C-16), 66.4 (C, C-2'), 65.1 (CH, C-3), 63.7 (CH, C-3'), 55.1 (CH, C-12), 54.3 (CH, C-12'), 50.6 (CH, C-9'), 49.7 (CH, C-9), 47.6 (CH_2, C-4), 47.2 (CH, C-17), 46.9 (CH_2, C-4'), 43.1 (CH, C-5'), 42.7 (CH, C-5), 39.9 (CH_2, C-6), 38.6 (CH_2, C-6'), 28.2 (CH_3, *t*Bu), 18.3 (CH_3, C-14'), 18.1 (CH_3, C-14), 12.7 (CH_3, C-11), 12.4 (CH_3, C-11'), 11.6 (CH_3, C-13), 10.4 (CH_3, C-13'); **MS** (ESI+) m/z, (%): 451.0 (451.2) (12, $[M+2H-C_4H_8]^{2+}$), 479.0 (479.3) (100, $[M+2H]^{2+}$), 957 (13, $[M+H]^+$), 979 (5, $[M+Na]^+$); **HRMS** (ESI+) m/z: $[M+H]^+$ Calcd for $C_{55}H_{73}N_8O_7$ 957.5597; Found 957.5590.

α/γ-Peptide NH_2(Ala-(*S*,*S*,*S*,*S*)^{P}AAMP)$_2$O*t*Bu (NH_2-4AS^{P}A)

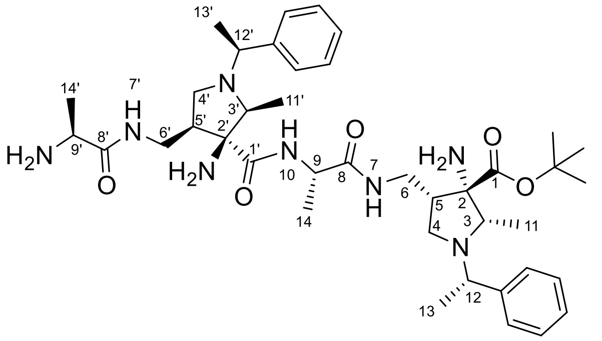

Prepared according to general **procedure C** from protected tetrapeptide **Fmoc-4AS^{P}A** (220 mg, 0.23 mmol). The residue was purified by flash chromatography ($CHCl_3$/MeOH + 1% TEA, neat to 10:1 gradient) to afford 165 mg (98%) of free amine **NH_2-4AS^{P}A** as a pale yellow amorphous solid.

$[\alpha]^{20}_{D}$: +21.5 (c 0.2, $CHCl_3$); **IR** ν[cm^{-1}]: 665, 700, 753, 784, 846, 1026, 1150, 1255, 1370, 1390, 1452, 1495, 1510, 1601, 1651, 1723, 2854, 2928, 2970, 3028, 3062, 3083, 3285; **^{1}H NMR** (401 MHz, $CDCl_3$) δ 8.10 (d, *J* = 8.0 Hz, 1H, H-10), 7.39-7.25 (m, 9H, Ar, H-7), 7.26-7.16 (m, 2H, Ar), 7.15 (t, *J* = 5.8 Hz, 1H, H-7'), 4.28 (dq, *J* = 7.8, 6.9 Hz, 1H, H-9), 3.99 (q, *J* = 6.8 Hz, 1H, H-12'), 3.95 (q, *J* = 6.8 Hz, 1H, H-12), 3.44-3.37 (m, 1H, H-6a), 3.32-3.23 (m, 1H, H-6a'), 3.25 (q, *J* = 7.0 Hz, 1H, H-9'), 3.17 (q, *J* = 6.3 Hz, 1H, H-3'), 3.16-3.09 (m, 1H, H-6b), 3.05-2.97 (m, 1H, H-6b'), 3.02 (q, *J* = 6.2 Hz, 1H, H-3), 2.95-2.86 (m, 1H, H-5'), 2.81 (t, *J* = 9.9 Hz, 1H, H-4a'), 2.77-2.70 (m, 2H, H-4a, H-5), 2.28-2.19 (m, 1H, H-4b), 2.09 (dd, *J* = 9.4, 7.8 Hz, 1H, H-4b'), 2.03 (bs, 6H, $3NH_2$), 1.47 (s, 9H, *t*Bu), 1.30 (d, *J* = 6.7 Hz, 6H, H-13, H-13'), 1.29 (d, *J* = 7.0 Hz, 3H, H-14), 1.14 (d, *J* = 7.0 Hz, 3H, H-14'), 0.94 (d, *J* = 6.3 Hz, 3H, H-11'), 0.90 (d, *J* = 6.2

Hz, 3H, H-11); **^{13}C NMR** (101 MHz, $CDCl_3$) δ 176.1 (C, C-8'), 174.4 (C, C-1'), 173.5 (C, C-1), 172.1 (C, C-8), 143.8 (C, Ar), 143.6 (C, Ar'), 128.3 (2CH, Ar), 127.6 (2CH, Ar), 127.0 (2CH, Ar), 82.2 (C, *t*Bu), 68.0 (C, C-2), 66.5 (C, C-2'), 64.9 (CH, C-3), 63.7 (CH, C-3'), 55.4 (2CH, C-12, C-12'), 50.6 (CH, C-9'), 49.0 (CH, C-9), 47.6 (CH_2, C-4), 47.0 (CH_2, C-4'), 43.4 (CH, C-5'), 43.0 (CH, C-5), 39.3 (CH_2, C-6), 38.1 (CH_2, C-6'), 28.2 (CH_3, *t*Bu), 21.3 (CH_3, C-14'), 18.7 (CH_3, C-14), 12.7 (CH_3, C-11), 12.3 (CH_3, C-11'), 11.7 ($2CH_3$, C-13, C-13'); **MS** (ESI+) m/z, (%): 340.0 (340.2) (71, $[M+2H-C_4H_8]^{2+}$), 368.0 (368.2) (100, $[M+2H]^{2+}$), 575 (11, $[M+H-C_4H_8-PhCH=CH_2]^+$), 735 (63, $[M+H]^+$), 757 (36, $[M+Na]^+$); **HRMS** (ESI+) m/z: $[M+H]^+$ Calcd for $C_{40}H_{63}N_8O_5$ 735.4916; Found 735.4908.

α/γ-Peptide Fmoc(Ala-(*S*,*S*,*S*,*S*)^{P}AAMP)$_3$O*t*Bu (Fmoc-6AS^{P}A)

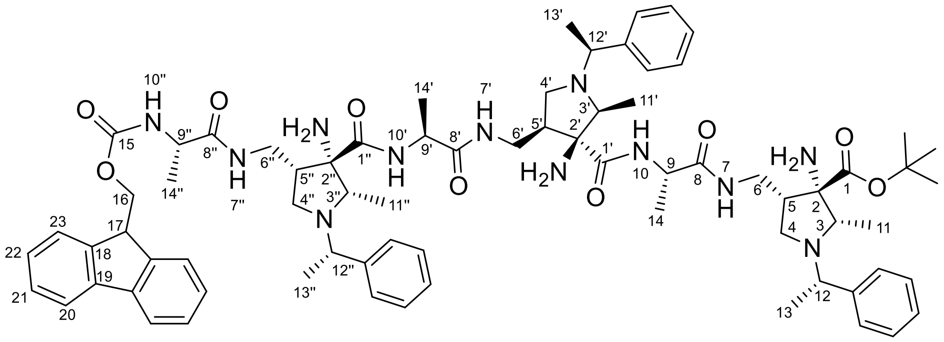

Prepared according to general **procedure B** from dipeptide **Fmoc-2AS^{P}A** (69 mg, 0.112 mmol), DIPEA (78 µL, 0.448 mmol), free amine **NH_2-4AS^{P}A** (93 mg, 0.127 mmol) and HATU (45 mg, 0.118 mmol). The residue was purified by flash chromatography (EtOAc/MeOH, 10:1 to 1:1 gradient) to afford 123 mg (85%) of protected hexapeptide **Fmoc-6AS^{P}A** as an off-white solid.

Mp: 118-120 °C; $[\alpha]_D^{20}$: +3.0 (c 0.2, $CHCl_3$); **IR** ν[cm^{-1}]: 699, 735, 758, 784, 846, 876, 911, 979, 1027, 1057, 1148, 1246, 1320, 1370, 1388, 1449, 1495, 1506, 1601, 1648, 1720, 2854, 2873, 2903, 2931, 2972, 3028, 3062, 3085, 3305, 3360; **^{1}H NMR** (600 MHz, $CDCl_3$) δ 8.08 (d, *J* = 6.8 Hz, 1H, H-10), 7.79 (d, *J* = 5.6 Hz, 1H, H-10'), 7.75 (dd, *J* = 7.6, 2.8 Hz, 2H, H-20), 7.60-7.57 (m, 2H, H-23), 7.40-7.16 (m, 18H, Ar, H-21, H-22, H-7, H-7'), 7.11 (t, *J* = 7.5 Hz, 2H, Ar), 7.04 (t, *J* = 7.3 Hz, 1H, Ar), 6.94 (dd, *J* = 7.0, 3.2 Hz, 1H, H-7''), 6.54 (d, *J* = 8.0 Hz, 1H, H-10''), 4.37-4.32 (m, 2H, H-16a, H-9''), 4.30-4.25 (m, 1H, H-9'), 4.23-4.17 (m, 3H, H-17, H-16b, H-9), 3.98 (q, *J* = 6.6 Hz, 1H, H-12''), 3.93 (q, *J* = 6.5 Hz, 1H, H-12), 3.79 (q, *J* = 6.4 Hz, 1H, H-12'), 3.52-3.47 (m, 1H, H-6a), 3.47-3.42 (m, 1H, H-6a''), 3.39-3.33 (m, 1H, H-6a'), 3.27 (q, *J* = 6.2 Hz, 1H, H-3'), 3.14 (q, *J* = 6.3 Hz, 1H, H-3''), 3.10-3.03 (m, 2H, H-6b, H-6b'), 3.01-2.89 (m, 3H, H-6b'', H-3, H-5''), 2.80 (t, *J* = 10.0 Hz, 1H, H-4a''), 2.75-2.66 (m, 3H, H-5, H-4a', H-4a), 2.66-2.60 (m, 1H, H-5'), 2.24-2.21 (m, 1H, H-4b), 2.04 (dd, *J* = 9.1, 7.3 Hz, 1H, H-4b''), 1.93-1.90 (m, 1H, H-4b'), 1.73 (s, 6H, $3NH_2$), 1.47 (s, 9H, *t*Bu), 1.38 (d, *J* = 7.1 Hz, 3H, H-14''), 1.29 (d, *J* = 6.9 Hz, 3H,

H-13), 1.27 (d, *J* = 6.8 Hz, 3H, H-13''), 1.25 (d, *J* = 7.1 Hz, 6H, H-14, H-14'), 1.15 (d, *J* = 6.4 Hz, 3H, H-13'), 0.90 (d, *J* = 6.3 Hz, 3H, H-11), 0.87 (d, *J* = 6.3 Hz, 6H, H-11', H-11''); **^{13}C NMR** (151 MHz, $CDCl_3$) δ 175.2 (C, C-1'), 175.0 (C, C-1''), 173.6 (C, C-1), 173.3 (C, C-8''), 173.2 (C, C-8'), 173.0 (C, C-8), 156.2 (C, C-15), 144.1 (C, Ar), 144.0/143.9 (C, C-18), 143.8 (C, Ar'), 143.7 (C, Ar''), 141.29/141.26 (C, C-19), 128.15 (CH, Ar), 128.13 (CH, Ar), 128.0 (CH, Ar), 127.7/127.6 (CH, C-21), 127.4 (CH, Ar), 127.32 (CH, Ar), 127.27 (CH, Ar), 127.1/127.0 (CH, C-22), 126.72 (CH, Ar), 126.69 (CH, Ar), 126.5 (CH, Ar), 125.4/125.3 (CH, C-23), 120.0 (CH, C-20), 81.8 (C, *t*Bu), 67.8 (C, C-2), 67.0 (CH_2, C-16), 66.6 (C, C-2'), 66.3 (C, C-2''), 64.9 (CH, C-3), 63.5 (CH, C-3''), 63.4 (CH, C-3'), 55.3 (CH, C-12'), 55.1 (CH, C-12), 54.2 (CH, C-12''), 50.5 (CH, C-9''), 49.8 (CH, C-9'), 49.2 (CH, C-9), 47.8 (CH_2, C-4'), 47.7 (CH_2, C-4), 47.1 (CH, C-17), 46.9 (CH_2, C-4''), 43.7 (CH, C-5'), 42.9 (CH, C-5''), 42.7 (CH, C-5), 39.7 (CH_2, C-6), 38.7 (2CH_2, C-6', C-6''), 28.1 (CH_3, *t*Bu), 18.5 (CH_3, C-14''), 17.7 (CH_3, C-14'), 17.6 (CH_3, C-14), 12.70 (CH_3, C-11), 12.66 (CH_3, C-11''), 12.4 (CH_3, C-11'), 11.9 (CH_3, C-13'), 11.7 (CH_3, C-13), 10.4 (CH_3, C-13''); **MS** (ESI+) m/z, (%): 429.7 (429.9) (28, $[M+3H]^{3+}$), 564.0 (564.3) (14, $[M+2H-C_4H_8-PhCH=CH_2]^{2+}$), 616.0 (644.4) (12, $[M+2H-C_4H_8]^{2+}$), 644.0 (644.4) (100, $[M+2H]^{2+}$), 655 (9, $[M+H+Na]^{2+}$), 1287 (72, $[M+H]^+$), 1309 (48, $[M+Na]^+$); **HRMS** (ESI+) m/z: $[M+H]^+$ Calcd for $C_{70}H_{93}N_{12}O_9$ 1287.7653; Found 1287.7655.

α/γ-Peptide NH$_2$(Ala-(*S*,*S*,*S*,*S*)^{P}AAMP)$_3$O*t*Bu (NH$_2$-6AS^{P}A)

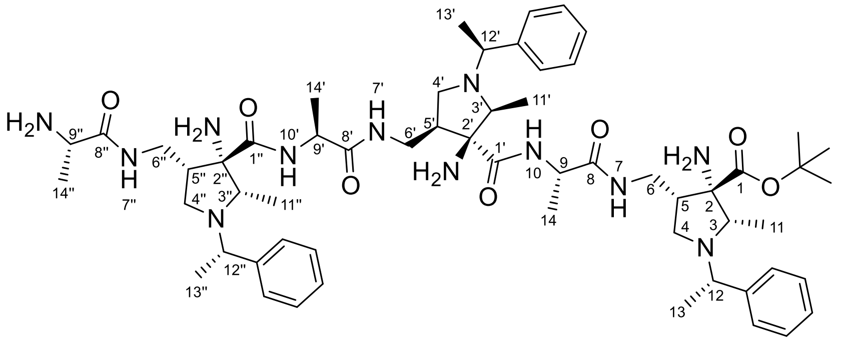

Prepared according to general **procedure C** from protected hexapeptide **Fmoc-6AS^{P}A** (103 mg, 0.08 mmol). The residue was purified by flash chromatography ($CHCl_3$/MeOH + 1% TEA, neat to 10:1 gradient) to afford 81 mg (98%) of free amine **NH$_2$-6AS^{P}A** as an off-white amorphous solid.

$[\alpha]_D^{20}$: +27.5 (c 0.2, $CHCl_3$); **IR** ν[cm^{-1}]: 665, 699, 751, 784, 847, 880, 950, 1025, 1064, 1150, 1217, 1252, 1370, 1390, 1450, 1495, 1508, 1601, 1647, 1722, 2855, 2873, 2931, 2971, 3028, 3059, 3086, 3304, 3352; **^{1}H NMR** (600 MHz, $CDCl_3$) δ 8.15 (d, *J* = 7.6 Hz, 1H, H-10), 7.99 (d, *J* = 7.7 Hz, 1H, H-10'), 7.38-7.25 (m, 13H, Ar, H-7), 7.25-7.17 (m, 3H, Ar), 7.24 (bs, 1H, H-7''), 7.17 (bs, 1H, H-7'), 4.33 (quint, *J* = 7.2 Hz, 1H, H-9), 4.32 (quint, *J* = 7.2 Hz, 1H, H-9'), 4.00 (q, *J* = 6.5 Hz, 1H, H-12'), 3.99 (q, *J* = 6.4 Hz, 1H, H-12''), 3.94 (q, *J* = 6.7 Hz, 1H, H-12), 3.49 (ddd, *J* = 13.7, 7.0, 5.0 Hz, 1H, H-6a), 3.37 (ddd, *J* = 13.5,

10.4, 7.5 Hz, 1H, H-6a''), 3.35-3.29 (m, 1H, H-6a'), 3.31 (q, *J* = 6.9 Hz, 1H, H-9''), 3.26 (q, *J* = 6.3 Hz, 1H, H-3'), 3.16 (ddd, *J* = 13.6, 8.6, 4.7 Hz, 1H, H-6b), 3.11 (q, *J* = 6.3 Hz, 1H, H-3''), 3.00 (q, *J* = 6.2 Hz, 1H, H-3), 2.99-2.95 (m, 1H, H-6b'), 2.95-2.90 (m, 1H, H-6b''), 2.90-2.86 (m, 1H, H-5''), 2.85-2.80 (m, 2H, H-5', H-4a'), 2.78 (t, *J* = 9.9 Hz, 1H, H-4a''), 2.75-2.70 (m, 2H, H-4a, H-5), 2.27 (dd, *J* = 7.4, 3.9 Hz, 1H, H-4b), 2.10-2.03 (m, 2H, H-4b', H-4b''), 1.78 (bs, 8H, $4NH_2$), 1.48 (s, 9H, *t*Bu), 1.31 (d, *J* = 6.7 Hz, 3H, H-13), 1.301 (d, *J* = 6.7 Hz, 3H, H-13'), 1.299 (d, *J* = 7.2 Hz, 3H, H-14), 1.28 (d, *J* = 6.7 Hz, 3H, H-13''), 1.20 (d, *J* = 7.0 Hz, 3H, H-14'), 1.18 (d, *J* = 6.9 Hz, 3H, H-14''), 0.97 (d, *J* = 6.3 Hz, 3H, H-11'), 0.911 (d, *J* = 6.3 Hz, 3H, H-11''), 0.909 (d, *J* = 6.3 Hz, 3H, H-11); **^{13}C NMR** (151 MHz, $CDCl_3$) δ 176.8 (C, C-8''), 174.8 (C, C-1'), 174.2 (C, C-1''), 173.7 (C, C-1), 172.8 (C, C-8'), 172.5 (C, C-8), 144.3/144.00/143.96 (3C, Ar, Ar', Ar''), 128.27/128.26/128.25 (3CH, Ar, Ar', Ar''), 127.6/127.5/127.4 (3CH, Ar, Ar', Ar''), 126.81/126.80/126.78 (3CH, Ar, Ar', Ar''), 81.9 (C, *t*Bu), 68.0 (C, C-2), 66.50 (C, C-2'), 66.48 (C, C-2''), 65.0 (CH, C-3), 63.7 (CH, C-3''), 63.2 (CH, C-3'), 55.2 (CH, C-12), 54.5 (CH, C-12'), 54.3 (CH, C-12''), 50.7 (CH, C-9''), 49.3 (CH, C-9), 48.6 (CH, C-9'), 47.8 (CH_2, C-4), 46.9 (CH_2, C-4''), 46.8 (CH_2, C-4'), 43.62 (CH, C-5'), 43.59 (CH, C-5''), 43.0 (CH, C-5), 39.8 (CH_2, C-6), 38.3 (CH_2, C-6'), 38.1 (CH_2, C-6''), 28.2 (CH_3, *t*Bu), 21.5 (CH_3, C-14''), 18.5 (CH_3, C-14'), 18.4 (CH_3, C-14), 12.8 (CH_3, C-11), 12.5 ($2CH_3$, C-11', C-11''), 11.9 (CH_3, C-13), 10.8 (CH_3, C-13'), 10.5 (CH_3, C-13''); **MS** (ESI+) m/z, (%): 355.7 (355.9) (11, $[M+3H]^{3+}$), 453.0 (453.3) (53, $[M+2H-C_4H_8-PhCH=CH_2]^{2+}$), 481.0 (481.3) (20, $[M+2H-PhCH=CH_2]^{2+}$), 505.0 (505.3) (7, $[M+2H-C_4H_8]^{2+}$), 533.0 (533.3) (100, $[M+2H]^{2+}$), 544.0 (544.3) (32, $[M+H+Na]^{2+}$), 1065 (23, $[M+H]^+$), 1087 (24, $[M+Na]^+$); **HRMS** (ESI+) m/z: $[M+H]^+$ Calcd for $C_{58}H_{89}N_{12}O_7$ 1065.6971; Found 1065.6976.

α/γ-Peptide Ac(Ala-(*S*,*S*,*S*,*S*)^{P}AAMP)$_3$O*t*Bu (Ac-6AS^{P}A)

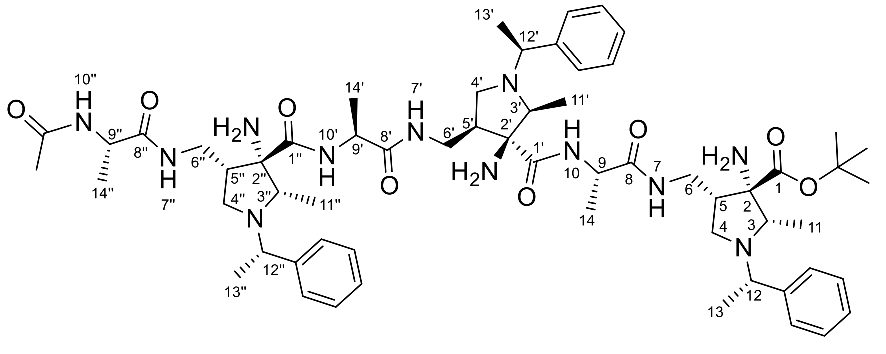

Prepared according to general **procedure B** from dipeptide **Ac-2AS^{P}A** (45 mg, 0.096 mmol)**,** DIPEA (84 µL, 0.482 mmol), free amine **NH_2-4AS^{P}A** (85 mg, 0.116 mmol) and HATU (44 mg, 0.116 mmol). The residue was purified by flash chromatography (EtOAc/MeOH, 10:1 to 1:1 gradient) to afford 96 mg (90%) of protected hexapeptide **Ac-6AS^{P}A** as an off-white solid.

Mp: 112-115 °C; $[\alpha]_D^{20}$: +24.1 (c 0.2, $CHCl_3$); **IR** ν[cm^{-1}]: 700, 732, 784, 847, 881, 911, 977, 1026, 1063, 1151, 1251, 1371, 1390, 1451, 1495, 1510, 1601, 1647, 1723, 2873, 2932, 2972, 3029, 3061, 3086, 3304; **^{1}H NMR** (600 MHz, $CDCl_3$) δ 8.09 (d, *J* = 6.8 Hz, 1H, H-10), 7.88 (d, *J* = 5.4 Hz, 1H, H-10'), 7.50 (bs, 1H, H-7'), 7.38-7.28 (m, 14H, Ar, H-7, H-10''), 7.24-7.19 (m, 3H, Ar), 6.83 (bs, 1H, H-7''), 4.49 (quint, *J* = 7.1 Hz, 1H, H-9''), 4.25 (dq, *J* = 7.1, 5.5 Hz, 1H, H-9'), 4.21 (quint, *J* = 7.1 Hz, 1H, H-9), 4.01 (q, *J* = 6.3 Hz, 1H, H-12'), 3.98 (q, *J* = 6.8 Hz, 1H, H-12''), 3.94 (q, *J* = 6.7 Hz, 1H, H-12), 3.52-3.47 (m, 2H, H-6a, H-6a''), 3.41-3.35 (m, 2H, H-3', H-6a'), 3.13-3.08 (m, 2H, H-6b, H-3''), 3.08-3.02 (m, 1H, H-6b'), 2.97 (q, *J* = 6.2 Hz, 1H, H-3), 2.93-2.87 (m, 2H, H-6b'', H-5''), 2.84-2.76 (m, 2H, H-4a', H-4a''), 2.74-2.67 (m, 3H, H-4a, H-5, H-5'), 2.28-2.22 (m, 1H, H-4b), 2.06-2.02 (m, 1H, H-4b', H-4b''), 1.97 (s, 3H, Ac), 1.81 (s, 6H, $3NH_2$), 1.47 (s, 9H, *t*Bu), 1.32-1.29 (m, 9H, H-13, H-13', H-14''), 1.263 (d, *J* = 6.6 Hz, 3H, H-13''), 1.259 (d, *J* = 7.2 Hz, 3H, H-14), 1.24 (d, *J* = 7.1 Hz, 3H, H-14'), 0.98 (d, *J* = 6.3 Hz, 3H, H-11'), 0.90 (d, *J* = 6.3 Hz, 3H, H-11), 0.86 (d, *J* = 6.3 Hz, 3H, H-11''); **^{13}C NMR** (151 MHz, $CDCl_3$) δ 175.4 (C, C-1'), 175.2 (C, C-1''), 173.7 (C, C-1), 173.33 (C, C-8'), 173.30 (C, C-8''), 173.2 (C, C-8), 170.3 (C, Ac), 144.2 (C, Ar), 144.0 (C, Ar'), 143.8 (C, Ar''), 128.31/128.28/128.25 (3CH, Ar, Ar', Ar''), 127.52/127.51/127.4 (3CH, Ar, Ar', Ar''), 126.89/126.87/126.8 (3CH, Ar, Ar', Ar''), 82.0 (C, *t*Bu), 68.0 (C, C-2), 66.7 (C, C-2'), 66.5 (C, C-2''), 65.1 (CH, C-3), 63.7 (CH, C-3''), 63.3 (CH, C-3'), 55.3 (CH, C-12), 54.4 (CH, C-12'), 54.3 (CH, C-12''), 50.0 (CH, C-9), 49.4 (CH, C-9'), 48.8 (CH, C-9''), 47.8 (CH_2, C-4), 47.4 (CH_2, C-4'), 47.0 (CH_2, C-4''), 43.9 (CH, C-5'), 43.0 (CH, C-5''), 42.8 (CH, C-5), 39.9 (CH_2, C-6), 38.9 (CH_2, C-6'), 38.8 (CH_2, C-6''), 28.2 (CH_3, *t*Bu), 23.3 (CH_3, Ac), 18.1 (CH_3, C-14''), 17.9 (CH_3, C-14'), 17.8 (CH_3, C-14), 12.8 (CH_3, C-11), 12.6 (CH_3, C-11'), 12.5 (CH_3, C-11''), 11.9 (CH_3, C-13), 10.5 (CH_3, C-13'), 10.4 (CH_3, C-13''); **MS** (ESI+) m/z, (%): 351.0 (351.2) (24, $[M+3H-C_4H_8]^{3+}$), 474.0 (474.3) (100, $[M+2H-C_4H_8-PhCH=CH_2]^{2+}$), 502.0 (502.3) (50, $[M+2H-PhCH=CH_2]^{2+}$), 554.0 (554.4) (86, $[M+2H]^{2+}$), 565.0 (565.4) (15, $[M+H+Na]^{2+}$), 1107 (2, $[M+H]^+$); **HRMS** (ESI+) m/z: $[M+2H]^{2+}$ Calcd for $C_{60}H_{92}N_{12}O_8$ 554.3580; Found 554.3575.

α/γ-Peptide Ac(Ala-(*S*,*S*,*S*,*S*)AAMP)$_3$O*t*Bu (Ac-6AS^{H}A)

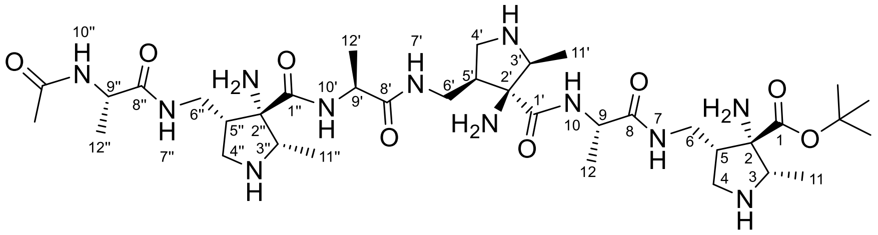

Prepared according to general **procedure D** from hexapeptide **Ac-6AS^{P}A** (22 mg, 0.02 mmol) providing 15 mg (95%) of free base **Ac-6AS^{H}A** as an off-white solid.

Mp: 135-137 °C; $[\alpha]_D^{20}$: +7.0 (c 0.2, MeOH); **IR** (MeOH, 0.025 mm CaF_2 cell) ν[cm^{-1}]: 1089, 1157, 1252, 1371, 1387, 1450, 1522, 1550, 1653, 1724; **^{1}H NMR** (600 MHz, H_2O:D_2O 9:1, CD_3COOD, pH 4.5) δ 8.51 (d, *J* = 7.0 Hz, 1H, H-10), 8.48 (d, *J* = 6.2 Hz, 1H, H-10'), 8.39 (t, *J* = 6.5 Hz, 1H, H-7), 8.33 (dd, *J* = 6.8, 5.7 Hz, 1H, H-7'), 8.27 (d, *J* = 5.9 Hz, 1H, H-10''), 8.18 (t, *J* = 6.2 Hz, 1H, H-7''), 4.36-4.27 (m, 2H, H-9, H-9'), 4.19 (dq, *J* = 7.3, 5.8 Hz, 1H, H-9''), 4.06 (q, *J* = 6.9 Hz, 1H, H-3), 4.02 (q, *J* = 7.0 Hz, 1H, H-3'), 4.00 (q, *J* = 7.0 Hz, 1H, H-3''), 3.65 (dd, *J* = 12.3, 9.2 Hz, 1H, H-4a'), 3.64 (dd, *J* = 12.1, 9.4 Hz, 1H, H-4a), 3.62 (dd, *J* = 12.6, 9.6 Hz, 1H, H-4a''), 3.53 (ddd, *J* = 14.2, 7.8, 6.5 Hz, 1H, H-6a), 3.49 (ddd, *J* = 14.1, 8.6, 6.9 Hz, 1H, H-6a'), 3.39 (ddd, *J* = 14.2, 8.0, 6.2 Hz, 1H, H-6a''), 3.34 (dt, *J* = 14.1, 6.5 Hz, 1H, H-6b''), 3.33 (dt, *J* = 14.2, 6.0 Hz, 1H, H-6b'), 3.29 (dt, *J* = 14.2, 6.7 Hz, 1H, H-6b), 3.26 (dd, *J* = 12.1, 11.4 Hz, 1H, H-4b'), 3.24 (dd, *J* = 12.1, 11.3 Hz, 1H, H-4b''), 3.18 (dd, *J* = 12.0, 11.1 Hz, 1H, H-4b), 3.13-3.07 (m, 1H, H-5), 3.10-3.04 (m, 1H, H-5'), 3.05-2.99 (m, 1H, H-5''), 2.03 (s, 3H, Ac), 1.49 (s, 9H, *t*Bu), 1.39 (d, *J* = 7.2 Hz, 3H, H-12), 1.37 (d, *J* = 7.3 Hz, 3H, H-12'), 1.31 (d, *J* = 7.4 Hz, 3H, H-12''), 1.30 (d, *J* = 7.0 Hz, 3H, H-11), 1.28 (d, *J* = 7.0 Hz, 3H, H-11'), 1.27 (d, *J* = 7.1 Hz, 3H, H-11''); the amine NH and NH_2 resonances not detectable likely due to water presaturation; **^{13}C NMR** (151 MHz, D_2O, CD_3COOD, pH 4.5) δ 176.2 (C, C-8''), 175.3 (C, C-8'), 175.0 (C, C-8), 174.7 (C, C-Ac), 173.7 (C, C-1''), 173.2 (C, C-1'), 172.9 (C, C-1), 85.4 (C, *t*Bu), 66.8 (C, C-2'), 66.7 (C, C-2''), 66.3 (C, C-2), 63.4 (CH, C-3'), 63.3 (CH, C-3''), 63.1 (CH, C-3), 50.8 (CH, C-9'), 50.6 (CH, C-9''), 50.5 (CH, C-9), 46.74/46.65/46.63 (3CH_2, C-4, C-4', C-4''), 46.57/46.49/46.47 (3CH, C-5, C-5', C-5''), 37.3 (CH_2, C-6), 37.2 (CH_2, C-6'), 37.1 (CH_2, C-6''), 27.7 (CH_3, *t*Bu), 22.3 (CH_3, Ac), 18.1 (CH_3, C-12), 17.6 (CH_3, C-12'), 17.2 (CH_3, C-12''), 10.7 (CH_3, C-11), 10.5 (2CH_3, C-11', C-11''); **MS** (ESI+) m/z, (%): 247.0 (247.2) (57, $[M+3H-C_4H_8]^{3+}$), 370.0 (370.2) (100, $[M+2H-C_4H_8]^{2+}$), 398.0 (398.3) (61, $[M+2H]^{2+}$), 795 (5, $[M+H]^+$), 817 (39, $[M+Na]^+$); **HRMS** (ESI+) m/z: $[M+2H]^{2+}$ Calcd for $C_{36}H_{48}N_{12}O_8$ 398.2636; Found 398.2639.

α/γ-Peptide Fmoc(Ala-(*S,S,S,S*)^{P}AAMP)$_4$O*t*Bu (Fmoc-8AS^{P}A)

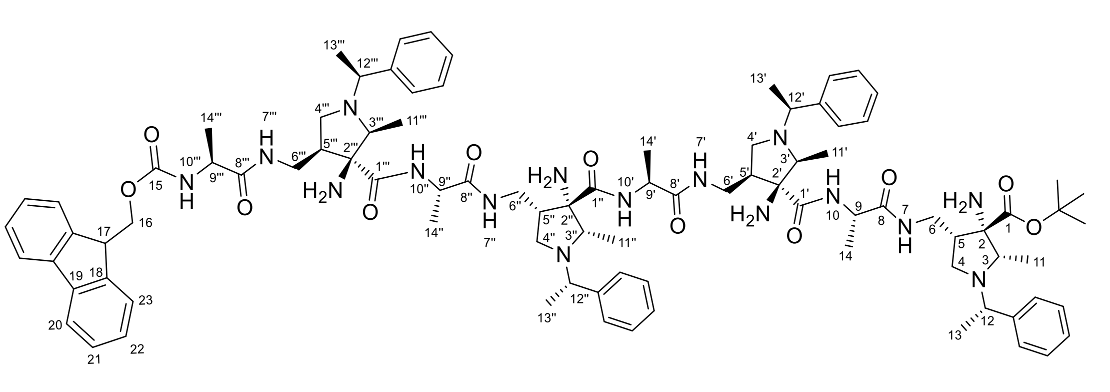

Prepared according to general **procedure B** from tetrapeptide **Fmoc-4AS^{P}A** (182 mg, 0.19 mmol)**,** DIPEA (0.20 mL, 1.13 mmol), free amine **NH_2-4AS^{P}A** (175 mg, 0.24 mmol) and HATU (86 mg, 0.23

mmol). The residue was purified by flash chromatography (EtOAc/MeOH, 10:1 to 1:1 gradient) to afford 253 mg (83%) of protected octapeptide **Fmoc-8AS^{P}A** as an off-white solid.

Mp: 128-131 °C; $[\alpha]_D^{20}$: +57.0 (c 0.2, $CHCl_3$); **IR** ν[cm^{-1}]: 700, 733, 759, 784, 874, 911, 1028, 1061, 1152, 1247, 1283, 1321, 1372, 1390, 1451, 1494, 1510, 1601, 1651, 1722, 2874, 2932, 2972, 3030, 3064, 3085, 3308; **^{1}H NMR** (401 MHz, $CDCl_3$) δ 8.13 (d, *J* = 6.2 Hz, 1H, H-10), 8.00 (d, *J* = 5.0 Hz, 1H, H-10'), 7.83 (d, *J* = 4.5 Hz, 1H, H-10''), 7.75 (dd, *J* = 7.6, 3.2 Hz, 2H, H-20), 7.70 (bs, 1H, H-7''), 7.62-7.56 (m, 3H, H-7', H-23), 7.44-7.18 (m, 20H, Ar, H-21, H-22), 7.16-7.05 (m, 4H, H-7, Ar), 7.03-6.94 (m, 2H, H-7''', Ar), 6.74 (d, *J* = 7.1 Hz, 1H, H-10'''), 4.42-4.29 (m, 3H, H-9', H-9'', H-9'''), 4.41 (dd, *J* = 10.3, 7.1 Hz, 1H, H-16a), 4.22 (t, *J* = 7.3 Hz, 1H, H-17), 4.17-4.09 (m, 2H, H-9, H-16b), 4.00 (q, *J* = 6.9 Hz, 1H, H-12'), 3.98 (q, *J* = 6.9 Hz, 1H, H-12''), 3.93 (q, *J* = 6.8 Hz, 1H, H-12), 3.72 (q, *J* = 6.8 Hz, 1H, H-12'''), 3.59-3.44 (m, 4H, H-6a, H-6a', H-6a'', H-6a'''), 3.39 (q, *J* = 6.1 Hz, 1H, H-3'), 3.24 (q, *J* = 6.3 Hz, 1H, H-3'''), 3.14-3.03 (m, 1H, H-6b), 3.13 (q, *J* = 6.3 Hz, 1H, H-3''), 3.00-2.90 (m, 4H, H-5''', H-6b', H-6b'', H-6b'''), 2.96 (q, *J* = 6.3 Hz, 1H, H-3), 2.84-2.62 (m, 7H, H-4a, H-4a', H-4a'', H-4a''', H-5, H-5', H-5''), 2.25 (dd, *J* = 10.8, 4.0 Hz, 1H, H-4b), 2.07-2.00 (m, 2H, H-4b', H-4b''), 1.93-1.88 (m, 1H, H-4b'''), 1.77 (bs, 8H, $4NH_2$), 1.47 (s, 9H, *t*Bu), 1.42 (d, *J* = 7.1 Hz, 3H, H-14'''), 1.33-1.24 (m, 9H, H-13, H-13', H-13''), 1.30 (d, *J* = 6.7 Hz, 3H, H-14''), 1.22 (d, *J* = 7.2 Hz, 3H, H-14), 1.18 (d, *J* = 7.2 Hz, 3H, H-14'), 1.08 (d, *J* = 6.7 Hz, 3H, H-13'''), 0.98 (d, *J* = 6.3 Hz, 3H, H-11'), 0.90 (d, *J* = 6.3 Hz, 3H, H-11), 0.87 (d, *J* = 6.3 Hz, 3H, H-11''), 0.74 (d, *J* = 6.2 Hz, 3H, H-11'''); **^{13}C NMR** (101 MHz, $CDCl_3$) δ 175.51 (C, C-1'), 175.46 (C, C-1''), 175.1 (C, C-1'''), 174.1 (C, C-8''), 173.6 (C, C-1), 173.5 (C, C-8'''), 173.4 (C, C-8'), 173.3 (C, C-8), 156.2 (C, C-15), 144.3/144.19/144.1/143.7 (4C, Ar, Ar', Ar'', Ar'''), 144.22/143.9 (C, C-18), 141.3 (C, C-19), 128.29/128.27/128.2/128.1 (4CH, Ar, Ar', Ar'', Ar'''), 127.81/127.78 (CH, C-21), 127.6/127.5/127.39/127.36 (4CH, Ar, Ar', Ar'', Ar'''), 127.21/127.16 (CH, C-22), 126.9/126.84/126.76/126.6 (4CH, Ar, Ar', Ar'', Ar'''), 125.4/125.2 (CH, C-23), 119.9 (CH, C-20), 81.9 (C, *t*Bu), 67.8 (C, C-2), 67.0 (CH_2, C-16), 66.8 (C, C-2'''), 66.7 (C, C-2'), 66.3 (C-2''), 65.0 (CH, C-3), 64.0 (CH, C-3'''), 63.5 (CH, C-3''), 63.2 (CH, C-3'), 56.0 (CH, C-12'''), 55.2 (CH, C-12), 54.4 (CH, C-12''), 54.1 (CH, C-12'), 50.5 (CH, C-9'''), 50.2 (CH, C-9), 49.7 (CH, C-9'), 49.3 (CH, C-9''), 48.6 (CH_2, C-4'''), 47.8 (CH_2, C-4), 47.4 (CH_2, C-4''), 47.1 (CH, C-17), 47.0 (CH_2, C-4'), 43.9/43.4/42.83/42.80 (4CH, C-5, C-5', C-5'', C-5'''), 39.8 (CH_2, C-6), 39.3/39.1/39.0 ($3CH_2$, C-6', C-6'', C-6'''), 28.1 (CH_3, *t*Bu), 18.5 (CH_3, C-14'''), 17.7 (CH_3, C-14''), 17.2 (CH_3, C-14), 16.9 (CH_3, C-14'), 13.0 ($2CH_3$, C-11''', C-13'''), 12.7 (CH_3, C-11'), 12.6 (CH_3, C-11), 12.4 (CH_3, C-11''), 11.9 (CH_3, C-13), 10.4 ($2CH_3$, C-13', C-13''); **MS** (ESI+) m/z, (%): 405.0 (405.2) (47, $[M+4H]^{4+}$), 539.7 (540.0) (100, $[M+3H]^{3+}$), 809.0 (809.4) (85, $[M+2H]^{2+}$), 820.0 (820.4) (41, $[M+H+Na]^{2+}$), 1617 (11, $[M+H]^{+}$), 1639 (8, $[M+Na]^{+}$); **HRMS** (ESI+) m/z: $[M+Na]^{+}$ Calcd for $C_{91}H_{124}N_{16}O_{11}$ 1639.9528; Found 1639.9532.

α/γ-Peptide NH$_2$(Ala-(*S*,*S*,*S*,*S*)^{P}AAMP)$_4$O*t*Bu (NH$_2$-8AS^{P}A)

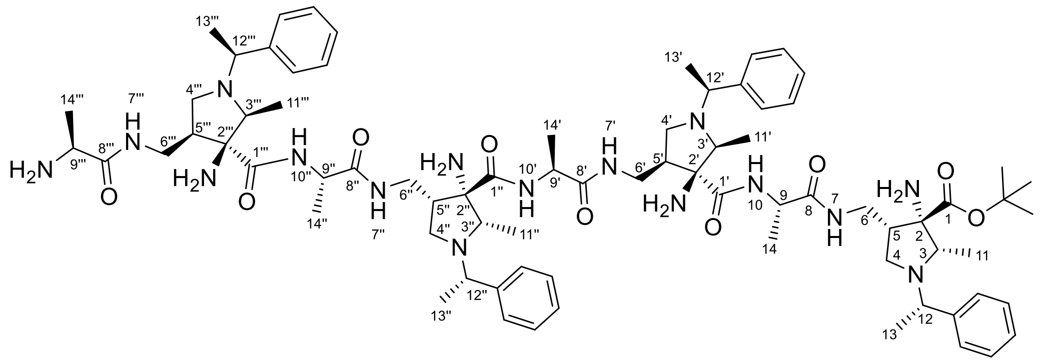

Prepared according to general **procedure C** from protected octapeptide **Fmoc-8AS^{P}A** (259 mg, 0.16 mmol). The residue was purified by flash chromatography (CHCl$_3$/MeOH + 1% TEA, neat to 5:1 gradient) to afford 216 mg (98%) of free amine **NH$_2$-8AS^{P}A** as an off-white solid.

Mp: 126-128 °C; $[\alpha]_D^{20}$: +41.0 (c 0.2, CHCl$_3$); **IR** ν[cm^{-1}]: 700, 732, 784, 847, 879, 911, 1026, 1063, 1152, 1211, 1253, 1371, 1389, 1451, 1495, 1509, 1601, 1650, 1724, 2856, 2872, 2931, 2972, 3028, 3062, 3087, 3313, 3360; **^{1}H NMR** (401 MHz, CDCl$_3$) δ 8.17 (d, *J* = 7.1 Hz, 1H, H-10), 8.03 (d, *J* = 7.1 Hz, 1H, H-10''), 8.02 (d, *J* = 7.2 Hz, 1H, H-10'), 7.51 (dd, *J* = 6.3, 3.4 Hz, 1H, H-7'), 7.44 (dd, *J* = 5.9, 3.5 Hz, 1H, H-7''), 7.38-7.26 (m, 18H, H-7, H-7''', Ar), 7.24-7.18 (m, 4H, Ar), 4.41-4.33 (m, 2H, H-9', H-9''), 4.26 (quint, *J* = 6.9 Hz, 1H, H-9), 4.03-3.91 (m, 4H, H-12, H-12', H-12'', H-12'''), 3.52-3.42 (m, 1H, H-6a), 3.42-3.30 (m, 5H, H-3', H-6a', H-6a'', H-6a''', H-9'''), 3.24 (q, *J* = 6.2 Hz, 1H, H-3'''), 3.17-3.07 (m, 1H, H-6b), 3.12 (q, *J* = 6.3 Hz, 1H, H-3''), 3.06-2.87 (m, 4H, H-5''', H-6b', H-6b'', H-6b'''), 2.99 (d, *J* = 6.2 Hz, 1H, H-3), 2.86-2.69 (m, 7H, H-4a, H-4a', H-4a'', H-4a''', H-5, H-5', H-5''), 2.26 (dd, *J* = 10.6, 3.9 Hz, 1H, H-4b), 2.16-2.04 (m, 3H, H-4b', H-4b'', H-4b'''), 1.88 (s, 10H, 5NH$_2$), 1.48 (s, 9H, *t*Bu), 1.33-1.20 (m, 24H, H-13, H-13', H-13'', H-13''', H-14, H-14', H-14'', H-14'''), 0.96 (d, *J* = 6.3 Hz, 3H, H-11'), 0.91 (d, *J* = 6.3 Hz, 6H, H-11, H-11''), 0.90 (d, *J* = 6.4 Hz, 3H, H-11'''); **^{13}C NMR** (101 MHz, CDCl$_3$) δ 176.6 (C, C-8'''), 175.1 (C, C-1'), 174.9 (C, C-1'''), 174.3 (C, C-1''), 173.6 (C, C-1), 173.5 (C, C-8''), 173.0 (C, C-8'), 172.8 (C, C-8), 144.1/143.91/143.89/143.8 (4C, Ar, Ar', Ar'', Ar'''), 128.2/128.14/128.12/128.10 (4CH, Ar, Ar', Ar'', Ar'''), 127.5/127.44/127.42/127.3 (4CH, Ar, Ar', Ar'', Ar'''), 126.8/126.70/126.68/126.67 (4CH, Ar, Ar', Ar'', Ar'''), 81.9 (C, *t*Bu), 67.8 (C, C-2), 66.6 (C, C-2'''), 66.5 (C, C-2'), 66.3 (C, C-2''), 64.9 (CH, C-3), 63.5/63.44/63.40 (3CH, C-3', C-3'', C-3'''), 55.2 (CH, C-12), 54.54/54.51/54.3 (3CH, C-12', C-12'', C-12'''), 50.5 (CH, C-9'''), 49.7 (CH, C-9), 49.1 (CH, C-9'), 48.6 (CH, C-9''), 47.8 (CH$_2$, C-4), 47.4/47.2/46.9 (3CH$_2$, C-4', C-4'', C-4'''), 43.8/43.34/43.31/42.8 (4CH, C-5, C-5', C-5'', C-5'''), 39.7 (CH$_2$, C-6), 38.6/38.5/38.1 (3CH$_2$, C-6', C-6'', C-6'''), 28.1 (CH$_3$, *t*Bu), 21.4 (CH$_3$, C-14'''), 18.4 (CH$_3$, C-14''), 17.8 (CH$_3$, C-14), 17.6 (CH$_3$, C-14'), 12.7/12.61/12.56/12.4 (4CH$_3$, C-11, C-11', C-11'', C-11'''), 11.8 (CH$_3$, C-

13), 11.0/10.8/10.6 ($3CH_3$, C-13', C-13'', C-13'''); **MS** (ESI+) m/z, (%): 412.3 (412.6) (53, $[M+3H-C_4H_8-PhCH=CH_2]^{3+}$), 465.7 (465.9) (100, $[M+3H]^{3+}$), 473.0 (473.3) (9, $[M+2H+Na]^{3+}$), 566.0 (566.3) (22, $[M+2H-C_4H_8-2PhCH=CH_2]^{2+}$), 618.0 (618.4) (5, $[M+2H-C_4H_8-PhCH=CH_2]^{2+}$), 698.0 (698.4) (31, $[M+2H]^{2+}$), 709.0 (709.4) (18, $[M+H+Na]^{2+}$); **HRMS** (ESI+) m/z: $[M+3H]^{3+}$ Calcd for $C_{76}H_{117}N_{16}O_9$ 465.9724; Found 465.9728.

α/γ-Peptide Boc(Ala-(*S*,*S*,*S*,*S*)^{P}AAMP)$_4$O*t*Bu (Boc-8AS^{P}A)

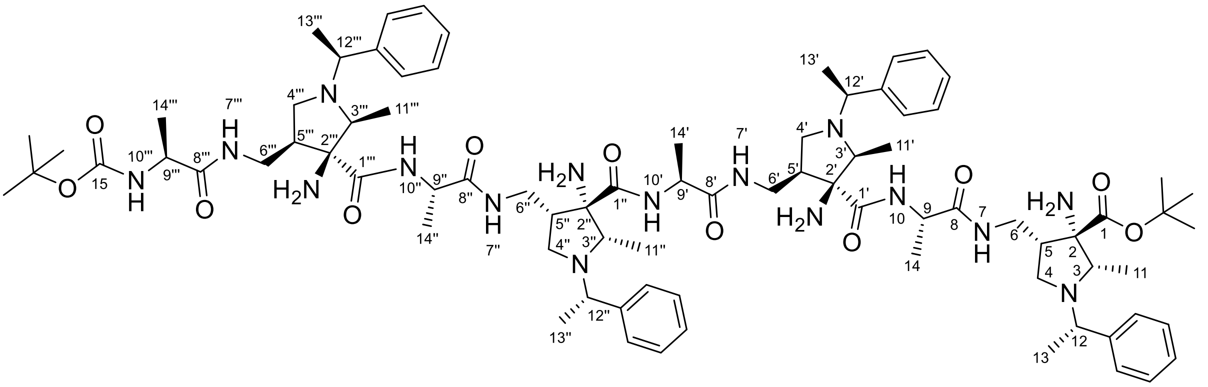

Prepared according to general **procedure E** from free amine **NH_2-8AS^{P}A** (114 mg, 0.082 mmol). The residue was purified by flash chromatography (EtOAc/MeOH, 10:1 to 1:1 gradient) to afford 91 mg (74%) of protected peptide **Boc-8AS^{P}A** as an off-white solid.

Mp: 122-125 °C; $[\alpha]_D^{20}$: +57.4 (c 0.2, $CHCl_3$); **IR** ν[cm^{-1}]: 700, 733, 784, 848, 882, 910, 978, 1026, 1059, 1157, 1249, 1369, 1388, 1452, 1495, 1509, 1601, 1651, 1717, 2874, 2932, 2973, 3029, 3063, 3311; **^{1}H NMR** (600 MHz, $CDCl_3$) δ 8.14 (d, *J* = 6.1 Hz, 1H, H-10), 8.01 (d, *J* = 4.7 Hz, 1H, H-10'), 7.84 (d, *J* = 3.7 Hz, 1H, H-10''), 7.67 (bs, 1H, H-7''), 7.59 (bs, 1H, H-7'), 7.38-7.27 (m, 16H, Ar), 7.24-7.18 (m, 4H, Ar), 7.14 (dd, *J* = 7.2, 4.3 Hz, 1H, H-7), 6.95 (bs, 1H, H-7'''), 6.22 (d, *J* = 8.5 Hz, 1H, H-10'''), 4.36-4.30 (m, 2H, H-9', H-9''), 4.26 (dq, *J* = 8.1, 7.6 Hz, 1H, H-9'''), 4.16 (dq, *J* = 7.2, 6.2 Hz, 1H, H-9), 4.00 (q, *J* = 6.7 Hz, 1H, H-12'), 3.96 (q, *J* = 6.7 Hz, 1H, H-12''), 3.95-3.92 (m, 1H, H-12'''), 3.93 (q, *J* = 6.7 Hz, 1H, H-12), 3.56-3.47 (m, 4H, H-6a, H-6a', H-6a'', H-6a'''), 3.40 (q, *J* = 6.2 Hz, 1H, H-3'), 3.34 (q, *J* = 6.3 Hz, 1H, H-3'''), 3.13-3.05 (m, 2H, H-3'', H-6b), 2.99-2.88 (m, 4H, H-5, H-6b', H-6b'', H-6b'''), 2.97 (q, *J* = 6.3 Hz, 1H, H-3), 2.87-2.65 (m, 6H, H-4a, H-4a'', H-4a''', H-5', H-5'', H-5'''), 2.81 (t, *J* = 9.8 Hz, 1H, H-4a'), 2.28-2.23 (m, 1H, H-4b), 2.12-2.08 (m, 1H, H-4b''), 2.08-1.99 (m, 2H, H-4b', H-4b'''), 1.76 (s, 8H, $4NH_2$), 1.48 (s, 9H, *t*Bu), 1.36 (s, 9H, Boc), 1.33 (d, *J* = 7.5 Hz, 3H, H-14'''), 1.31 (d, *J* = 6.8 Hz, 3H, H-13), 1.30-1.28 (m, 9H, H-13', H-13''', H-14''), 1.25 (d, *J* = 7.0 Hz, 1H, H-13''), 1.24 (d, *J* = 7.3 Hz, 3H, H-14), 1.18 (d, *J* = 7.2 Hz, 3H, H-14'), 0.99 (d, *J* = 6.3 Hz, 3H, H-11'), 0.90 (d, *J* = 6.3 Hz, 3H, H-11), 0.86 (d, *J* = 6.4 Hz, 3H, H-11'''), 0.85 (d, *J* = 6.3 Hz, 3H, H-11''); **^{13}C NMR** (151 MHz, $CDCl_3$) δ 175.54 (C, C-1'), 175.46 (C, C-1''),

175.3 (C, C-1'''), 174.1 (C, C-8'), 173.9 (C, C-8'''), 173.6 (C, C-1), 173.3 (2C, C-8, C-8''), 155.7 (C, C-15), 144.4 (C, Ar'''), 144.1 (C, Ar), 143.9 (C, Ar'), 143.8 (C, Ar''), 128.31/128.27/128.22/128.21 (4CH, Ar, Ar', Ar'', Ar'''), 127.62/127.59/127.5/127.4 (4CH, Ar, Ar', Ar'', Ar'''), 126.9/126.83/126.77/126.7 (4CH, Ar, Ar', Ar'', Ar'''), 81.9 (C, *t*Bu), 79.3 (C, Boc), 67.8 (C, C-2), 66.73 (C, C-2'''), 66.69 (C, C-2'), 66.3 (C, C-2''), 65.0 (CH, C-3), 63.8 (CH, C-3'''), 63.5 (CH, C-3''), 63.2 (CH, C-3'), 55.4 (CH, C-12'''), 55.2 (CH, C-12), 54.4 (CH, C-12'), 54.2 (CH, C-12''), 50.3 (CH, C-9), 50.0 (CH, C-9'''), 49.7 (CH, C-9'), 49.3 (CH, C-9''), 48.1 (CH_2, C-4''), 47.8 (CH_2, C-4), 47.4 (CH_2, C-4'), 47.1 (CH_2, C-4'''), 43.7 (CH, C-5'), 43.4 (CH, C-5''), 42.9 (CH, C-5'''), 42.7 (CH, C-5), 39.8 (CH_2, C-6), 39.1 (CH_2, C-6''), 38.9 (CH_2, C-6'), 38.8 (CH_2, C-6'''), 28.4 (CH_3, Boc), 28.1 (CH_3, *t*Bu), 18.4 (CH_3, C-14'''), 17.6 (CH_3, C-14''), 17.2 (CH_3, C-14), 17.0 (CH_3, C-14'), 12.8 (CH_3, C-11'''), 12.7 (CH_3, C-11), 12.6 (CH_3, C-11'), 12.4 (CH_3, C-11''), 12.0 (CH_3, C-13'''), 11.9 (CH_3, C-13), 10.4 ($2CH_3$, C-13'', C-13'); **MS** (ESI+) m/z, (%): 480.3 (480.7) (7, $[M+3H-C_4H_8]^{3+}$), 499.0 (499.3) (80, $[M+3H]^{3+}$), 566.0 (566.3) (75, $[M+2H-2C_4H_8-CO_2-2PhCH=CH_2]^{2+}$), 618.0 (618.3) (63, $[M+2H-2C_4H_8-CO_2-PhCH=CH_2]^{2+}$), 668.0 (668.4) (29, $[M+2H-C_4H_8-PhCH=CH_2]^{2+}$), 696.0 (696.4) (18, $[M+2H-PhCH=CH_2]^{2+}$), 748.0 (748.5) (100, $[M+2H]^{2+}$), 759.0 (759.5) (48, $[M+H+Na]^{2+}$), 1495 (2, $[M+H]^{+}$), 1517 (6, $[M+Na]^{+}$); **HRMS** (ESI+) m/z: $[M+2H]^{2+}$ Calcd for $C_{81}H_{124}N_{16}O_{11}$ 748.4812; Found 748.4817.

α/γ-Peptide Boc(Ala-(*S*,*S*,*S*,*S*)AAMP)$_4$O*t*Bu (Boc-8AS^{P}A)

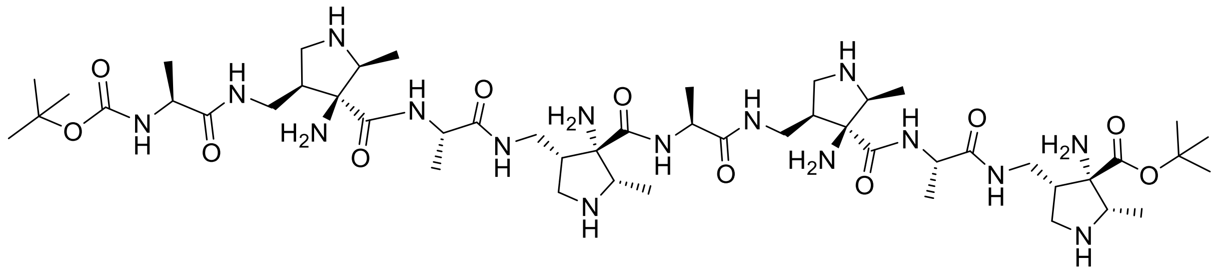

Prepared according to general **procedure D** from octapeptide **Boc-8AS^{P}A** (30 mg, 0.02 mmol) affording 21 mg (95%) of free base **Boc-8AS^{H}A** as an off-white amorphous solid.

$[\alpha]_D^{20}$: +4.3 (c 0.3, $CHCl_3$); **IR** ν[cm^{-1}]: 1161, 1250, 1368, 1392, 1454, 1506, 1553, 1715, 2873, 2929, 2973, 3069, 3303; ^{1}H NMR (600 MHz, H_2O:D_2O 9:1, CD_3COOD, pH 4.5) δ 8.52-8.46 (m, 3H, H-10, H-10', H-10''), 8.41-8.16 (m, 4H, H-7, H-7', H-7'', H-7'''), 6.91 (s, 1H, H-10'''), 4.37-4.28 (m, 3H, H-9, H-9', H-9''), 4.07 (q, *J* = 7.2 Hz, 1H, H-3), 4.04-3.94 (m, 4H, H-3', H-3'', H-3''', H-9'''), 3.69-3.59 (m, 4H, H-4a, H-4a', H-4a'', H-4a'''), 3.57-3.37 (m, 4H, H-6a, H-6a', H-6a'', H-6a'''), 3.37-2.93 (m, 12H, H-4b, H-4b', H-4b'', H-4b''', H-5, H-5', H-5'', H-5''', H-6b, H-6b', H-6b'', H-6b'''), 1.49 (s, 9H, *t*Bu), 1.43 (s, 9H, Boc), 1.40-1.35 (m, 12H, H-12, H-12', H-12'', H-12'''), 1.32-1.26 (m, 12H, H-11, H-11', H-11'', H-11'''); the

amine NH and NH_2 resonances not detectable likely due to water presaturation; **^{13}C NMR** (151 MHz, D_2O, CD_3COOD, pH 4.5) δ 175.19/175.16/174.9 (3C, C-1', C-1'', C-1''), 173.5/173.4/173.3/173.1 (4C, C-8, C-8', C-8'', C-8'''), 172.4 (C, C-1), 157.8 (C, C-13), 85.4 (C, *t*Bu), 81.9 (C, Boc), 66.7/66.64/66.61 (3C, C-2', C-2'', C-2'''), 66.4 (C, C-2), 63.4/63.31/63.30 (3CH, C-3', C-3'', C-3'''), 63.0 (CH, C-3), 51.3/50.69/50.66/50.4 (4CH, C-9, C-9', C-9'', C-9'''), 46.63 (3CH_2, C-4', C-4'', C-4'''), 46.58 (CH_2, C-4), 46.5/46.41/46.39/46.38 (4CH, C-5, C-5', C-5'', C-5'''), 37.2/37.13/37.08/37.06 (4CH_2, C-6, C-6', C-6'', C-6'''), 28.2 (CH_3, *t*Bu), 27.5 (CH_3, Boc), 18.0/17.6/17.5/17.4 (4CH_3, C-12, C-12', C-12'', C-12'''), 10.6/10.47/10.46/10.4 (4CH_3, C-11, C-11', C-11'', C-11'''); **MS** (ESI+) m/z, (%): 308.3 (308.6) (79, $[M+3H-2C_4H_8-CO_2]^{3+}$), 360.3 (360.6) (44, $[M+3H]^{3+}$), 512.0 (512.3) (34, $[M+2H-C_4H_8]^{2+}$), 540.0 (540.4) (100, $[M+2H]^{2+}$), 551.0 (551.4) (33, $[M+Na+H]^{2+}$), 1079 (22, $[M+H]^+$), 1101 (27, $[M+Na]^+$); **HRMS** (ESI+) m/z: $[M+H]^+$ Calcd for $C_{49}H_{91}N_{16}O_{11}$ 1079.7048; Found 1079.7047.

α/γ-Peptide Fmoc(Ala-(*R*,*R*,*R*,*R*)^{P}AAMP)O*t*Bu (Fmoc-2AR^{P}A)

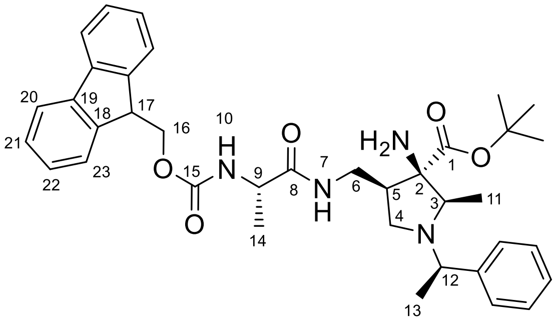

Prepared according to general **procedure A** from diamine **NH_2-R^{P}A** (400 mg, 1.20 mmol) and Fmoc-Ala-OH (311 mg, 1 mmol). The residue was purified by flash chromatography (cyclohexane/EtOAc, 1:1 to neat EtOAc gradient) to afford 583 mg (93%) of protected dipeptide **Fmoc-2AR^{P}A** as an off-white solid.

Mp: 129-131 °C; $[\alpha]_D^{20}$: −13.5 (c 0.2, $CHCl_3$); **IR** ν[cm^{-1}]: 701, 739, 759, 845, 911, 1078, 1107, 1148, 1247, 1287, 1319, 1339, 1369, 1388, 1450, 1495, 1523, 1601, 1662, 1721, 2874, 2932, 2974, 3030, 3039, 3063, 3315, 3394; **^{1}H NMR** (401 MHz, $CDCl_3$) δ 7.77 (td, *J* = 7.6, 1.0 Hz, 2H, H-20), 7.65-7.60 (m, 2H, H-23), 7.40 (tt, *J* = 7.5, 1.0 Hz, 2H, H-21), 7.36-7.19 (m, 8H, Ar, H-22, H-7), 5.57 (d, *J* = 7.5 Hz, 1H, H-10), 4.36 (dd, *J* = 10.7, 7.5 Hz, 1H, H-16a), 4.34 (dd, *J* = 10.5, 6.9 Hz, 1H, H-16b), 4.22 (t, *J* = 7.2 Hz, 1H, H-17), 4.14 (quint, *J* = 7.1 Hz, 1H, H-9), 3.96 (q, *J* = 6.7 Hz, 1H, H-12), 3.49 (ddd, *J* = 13.5, 6.7, 4.4 Hz, 1H, H-6a), 3.11 (ddd, *J* = 13.8, 8.4, 5.0 Hz, 1H, H-6b), 3.02 (q, *J* = 6.2 Hz, 1H, H-3), 2.78-2.68 (m, 2H, H-5, H-4a), 2.19-2.13 (m, 1H, H-4b), 1.70 (s, 2H, NH_2), 1.45 (s, 9H, *t*Bu), 1.31 (d, *J* = 7.0 Hz, 3H, H-14), 1.30 (d, *J* = 6.7 Hz, 3H, H-13), 0.89 (d, *J* = 6.2 Hz, 3H, H-11); **^{13}C NMR** (101 MHz, $CDCl_3$) δ 173.6 (C, C-1),

171.8 (C, C-8), 155.7 (C, C-15), 144.0 (C, C-18), 143.7 (C, Ar), 141.4 (C, C-19), 128.3 (CH, Ar), 127.8 (CH, C-21), 127.5 (CH, Ar), 127.2 (CH, C-22), 127.0 (CH, Ar), 125.4/125.3 (CH, C-23), 120.1 (CH, C-20), 82.2 (C, *t*Bu), 68.2 (C, C-2), 67.1 (CH_2, C-16), 64.5 (CH, C-3), 55.2 (CH, C-12), 50.7 (CH, C-9), 47.3 (CH, C-17), 46.9 (CH_2, C-4), 43.0 (CH, C-5), 39.3 (CH_2, C-6), 28.1 (CH_3, *t*Bu), 19.6 (CH_3, C-14), 12.6 (CH_3, C-11), 11.5 (CH_3, C-13); **MS** (ESI+) m/z, (%): 627 (100, $[M+H]^+$), 649 (5, $[M+Na]^+$); **HRMS** (ESI+) m/z: $[M+H]^+$ Calcd for $C_{37}H_{47}N_4O_5$ 627.3541; Found 627.3542.

α/γ-Peptide NH_2(Ala-(*R*,*R*,*R*,*R*)^{P}AAMP)O*t*Bu (NH_2-2AR^{P}A)

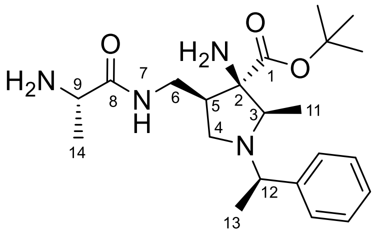

Prepared according to general **procedure C** from protected dipeptide **Fmoc-2AR^{P}A** (326 mg, 0.52 mmol). The residue was purified by flash chromatography ($CHCl_3$/MeOH + 1% TEA, neat to 25:1 gradient) to afford 200 mg (95%) of free amine **NH_2-2AR^{P}A** as a pale yellow oil.

$[\alpha]_D^{20}$: –35.5 (c 0.2, $CHCl_3$); **IR** v[cm^{-1}]: 701, 753, 784, 848, 1148, 1253, 1368, 1390, 1453, 1479, 1495, 1519, 1602, 1664, 1722, 2872, 2930, 2972, 3030, 3061, 3212, 3313, 3368; **^{1}H NMR** (400 MHz, $CDCl_3$) δ 7.55 (t, *J* = 5.6 Hz, 1H, H-7), 7.37-7.27 (m, 4H, Ar), 7.24-7.18 (m, 1H, Ar), 3.95 (q, *J* = 6.7 Hz, 1H, H-12), 3.44-3.37 (m, 1H, H-6a), 3.33 (q, *J* = 7.0 Hz, 1H, H-9), 3.20-3.12 (m, 1H, H-6b), 3.02 (q, *J* = 6.2 Hz, 1H, H-3), 2.78-2.72 (m, 2H, H-5, H-4a), 2.20-2.17 (m, 1H, H-4b), 1.50 (s, 4H, $2NH_2$), 1.47 (s, 9H, *t*Bu), 1.30 (d, *J* = 6.7 Hz, 3H, H-13), 1.24 (d, *J* = 6.9 Hz, 3H, H-14), 0.91 (d, *J* = 6.2 Hz, 3H, H-11); **^{13}C NMR** (101 MHz, $CDCl_3$) δ 175.6 (C, C-8), 173.7 (C, C-1), 144.2 (C, Ar), 128.2 (CH, Ar), 127.5 (CH, Ar), 126.8 (CH, Ar), 81.7 (C, *t*Bu), 67.9 (C, C-2), 64.6 (CH, C-3), 55.0 (CH, C-12), 51.0 (CH, C-9), 47.1 (CH_2, C-4), 43.6 (CH, C-5), 38.7 (CH_2, C-6), 28.2 (CH_3, *t*Bu), 21.9 (CH_3, C-14), 12.8 (CH_3, C-11), 11.4 (CH_3, C-13); **MS** (ESI+) m/z, (%): 405 (100, $[M+H]^+$), 427 (6, $[M+Na]^+$); **HRMS** (ESI+) m/z: $[M+H]^+$ Calcd for $C_{22}H_{37}N_4O_3$ 405.2860; Found 405.2865.

α/γ-Peptide Ac(Ala-(*R*,*R*,*R*,*R*)^{P}AAMP)$_4$O*t*Bu (Ac-2AR^{P}A)

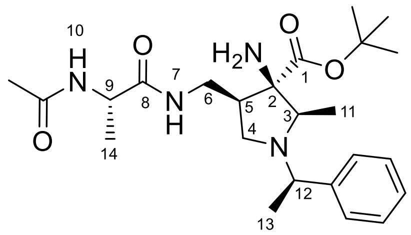

Prepared according to general **procedure A** from diamine **NH$_2$-R^{P}A** (400 mg, 1.20 mmol) and Ac-Ala-OH (131 mg, 1 mmol). The residue was purified by flash chromatography (cyclohexane/EtOAc, 1:1, gradient to neat EtOAc) to afford 403 mg (90%) of protected dipeptide **Ac-2AR^{P}A** as a pale yellow oil.

$[\alpha]_D^{20}$: –29.5 (c 0.2, CHCl$_3$); **IR** ν[cm^{-1}]: 701, 729, 784, 847, 975, 1025, 1150, 1252, 1282, 1370, 1391, 1451, 1494, 1545, 1601, 1647, 1723, 2873, 2932, 2974, 3026, 3067, 3083, 3294; **^{1}H NMR** (401 MHz, CDCl$_3$) δ 7.36-7.27 (m, 4H, Ar), 7.24-7.17 (m, 1H, Ar), 7.17 (dd, *J* = 6.2, 5.6 Hz, 1H, H-7), 6.29 (d, *J* = 7.3 Hz, 1H, H-10), 4.33 (quint, *J* = 7.0 Hz, 1H, H-9), 3.97 (q, *J* = 6.5 Hz, 1H, H-12), 3.45 (ddd, *J* = 13.5, 6.5, 4.2 Hz, 1H, H-6a), 3.10 (ddd, *J* = 13.7, 8.3, 5.2 Hz, 1H, H-6b), 3.01 (q, *J* = 6.1 Hz, 1H, H-3), 2.78-2.67 (m, 2H, H-4a, H-5), 2.17-2.13 (m, 1H, H-4b), 1.98 (s, 3H, Ac), 1.78 (s, 2H, NH$_2$), 1.45 (s, 9H, *t*Bu), 1.30 (d, *J* = 6.8 Hz, 3H, H-13), 1.25 (d, *J* = 7.0 Hz, 3H, H-14), 0.90 (d, *J* = 6.2 Hz, 3H, H-11); **^{13}C NMR** (101 MHz, CDCl$_3$) δ 173.7 (C, C-1), 171.9 (C, C-8), 169.6 (C, Ac), 143.9 (C, Ar), 128.3 (CH, Ar), 127.5 (CH, Ar), 126.9 (CH, Ar), 82.1 (C, *t*Bu), 68.2 (C, C-2), 64.4 (CH, C-3), 54.8 (CH, C-12), 48.9 (CH, C-9), 46.7 (CH$_2$, C-4), 43.0 (CH, C-5), 39.3 (CH$_2$, C-6), 28.1 (CH$_3$, *t*Bu), 23.4 (CH$_3$, Ac), 19.3 (CH$_3$, C-14), 12.6 (CH$_3$, C-11), 11.2 (CH$_3$, C-13); **MS** (ESI+) m/z, (%): 447 (100, [M+H]$^+$), 469 (14, [M+Na]$^+$); **HRMS** (ESI+) m/z: [M+H]$^+$ Calcd for $C_{24}H_{39}N_4O_4$ 447.2966; Found 447.2959.

α/γ-Peptide Fmoc(Ala-(*R*,*R*,*R*,*R*)^{P}AAMP)$_2$O*t*Bu (Fmoc-4AR^{P}A)

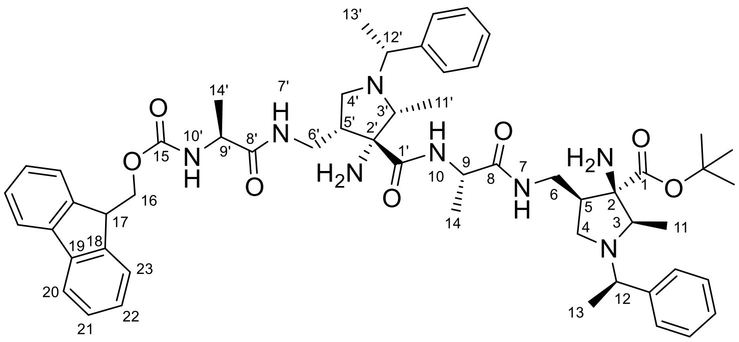

Prepared according to general **procedure B** from dipeptide **Fmoc-2AR^{P}A** (288 mg, 0.46 mmol), DIPEA (0.24 mL, 1.39 mmol), free amine **NH$_2$-2AR^{P}A** (193 mg, 0.48 mmol) and HATU (211 mg, 0.56 mmol).

The residue was purified by flash chromatography (EtOAc/MeOH, 10:1 to 2:1 gradient) to afford 419 mg (95%) of protected tetrapeptide **Fmoc-4AR^{P}A** as an off-white solid.

Mp: 123-125 °C; $[\alpha]_D^{20}$: –45.5 (c 0.2, $CHCl_3$); **IR** ν[cm^{-1}]: 700, 738, 759, 784, 845, 877, 910, 1047, 1079, 1108, 1149, 1250, 1319, 1337, 1370, 1390, 1450, 1494, 1509, 1601, 1643, 1721, 2873, 2932, 2972, 3038, 3063, 3086, 3293, 3363; **^{1}H NMR** (401 MHz, $CDCl_3$) δ 7.99 (d, *J* = 7.6 Hz, 1H, H-10), 7.76 (dd, *J* = 7.6, 1.0 Hz, 2H, H-20), 7.59 (d, *J* = 7.5 Hz, 2H, H-23), 7.44-7.24 (m, 12H, H-21, H-22, Ar), 7.26-7.16 (m, 2H, Ar), 7.12 (t, *J* = 5.8 Hz, 1H, H-7), 6.65 (bs, 1H, H-7'), 5.61 (d, *J* = 7.7 Hz, 1H, H-10'), 4.38 (dd, *J* = 10.3, 7.3 Hz, 1H, H-16a), 4.33 (dd, *J* = 10.4, 6.9 Hz, 1H, H-16b), 4.24 (quint, *J* = 7.2 Hz, 1H, H-9), 4.20 (t, *J* = 7.1 Hz, 1H, H-17), 4.13 (quint, *J* = 7.3 Hz, 1H, H-9'), 3.98 (q, *J* = 6.5 Hz, 1H, H-12'), 3.96 (q, *J* = 6.7 Hz, 1H, H-12), 3.42-3.28 (m, 2H, H-6a, H-6a'), 3.26-3.17 (m, 1H, H-6b), 3.19 (q, *J* = 6.3 Hz, 1H, H-3'), 3.08 (dt, *J* = 13.8, 4.6 Hz, 1H, H-6b'), 3.03 (q, *J* = 6.2 Hz, 1H, H-3), 2.87-2.75 (m, 2H, H-4a', H-5'), 2.75-2.67 (m, 2H, H-4a, H-5), 2.24-2.15 (m, 2H, H-4b, H-4b'), 2.21 (bs, 4H, $2NH_2$), 1.45 (s, 9H, *t*Bu), 1.30 (d, *J* = 7.0 Hz, 3H, H-14), 1.29 (d, *J* = 6.7 Hz, 6H, H-13, H-13'), 1.28 (d, *J* = 7.3 Hz, 3H, H-14'), 0.98 (d, *J* = 6.3 Hz, 3H, H-11'), 0.90 (d, *J* = 6.2 Hz, 3H, H-11); **^{13}C NMR** (101 MHz, $CDCl_3$) δ 174.7 (C, C-1'), 173.5 (C, C-1), 172.3 (C, C-8'), 171.8 (C, C-8), 155.9 (C, C-15), 143.94 (C, Ar), 143.92 (C, C-18), 143.89 (C, Ar), 141.4 (C, C-19), 128.33 (CH, Ar), 128.26 (CH, Ar), 127.8 (CH, C-21), 127.53 (CH, Ar), 127.47 (CH, Ar), 127.2 (CH, C-22), 126.92 (CH, Ar), 126.86 (CH, Ar), 125.2 (CH, C-23), 120.1 (CH, C-20), 81.9 (C, *t*Bu), 68.2 (C, C-2), 67.1 (CH_2, C-16), 66.7 (C, C-2'), 64.4 (CH, C-3), 63.5 (CH, C-3'), 55.0 (CH, C-12), 54.8 (CH, C-12'), 50.5 (CH, C-9'), 49.2 (CH, C-9), 47.2 (CH, C-17), 47.0 (CH_2, C-4'), 46.8 (CH_2, C-4), 43.23 (CH, C-5'), 43.16 (CH, C-5), 39.2 (CH_2, C-6), 38.8 (CH_2, C-6'), 28.1 (CH_3, *t*Bu), 18.9 (CH_3, C-14'), 18.6 (CH_3, C-14), 12.7 (CH_3, C-11), 12.1 (CH_3, C-11'), 11.31 (CH_3, C-13), 11.26 (CH_3, C-13'); **MS** (ESI+) m/z, (%): 451.0 (451.3) (18, $[M+2H-C_4H_8]^{2+}$), 479.0 (479.3) (100, $[M+2H]^{2+}$), 957 (13, $[M+H]^+$), 979 (5, $[M+Na]^+$); **HRMS** (ESI+) m/z: $[M+H]^+$ Calcd for $C_{55}H_{73}N_8O_7$ 957.5597; Found 957.5598.

α/γ-Peptide NH$_2$(Ala-(*R*,*R*,*R*,*R*)^{P}AAMP)$_2$O*t*Bu (NH$_2$-4AR^{P}A)

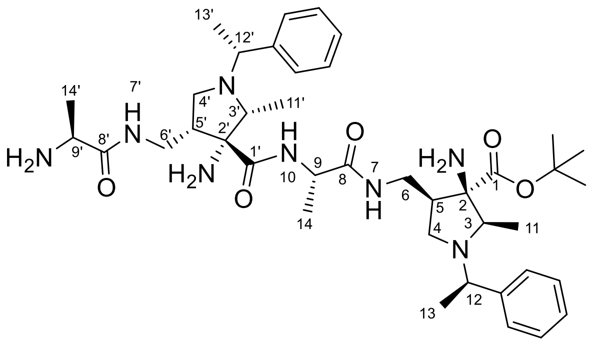

Prepared according to general **procedure C** from protected tetrapeptide **Fmoc-4AR^{P}A** (124 mg, 0.131 mmol). The residue was purified by flash chromatography ($CHCl_3$/MeOH + 1% TEA, neat to 10:1 gradient) to afford 92 mg (96%) of free amine **NH$_2$-4AR^{P}A** as an off-white amorphous solid.

$[\alpha]_D^{20}$: –59.0 (c 0.2, $CHCl_3$); **IR** ν[cm^{-1}]: 666, 700, 752, 784, 847, 878, 1025, 1149, 1254, 1320, 1369, 1390, 1451, 1495, 1513, 1601, 1652, 1722, 2873, 2931, 2972, 3027, 3060, 3085, 3312, 3354; **^{1}H NMR** (401 MHz, $CDCl_3$) δ 8.03 (d, *J* = 7.6 Hz, 1H, H-10), 7.38-7.27 (m, 9H, Ar, H-7'), 7.24-7.18 (m, 2H, Ar), 7.02 (t, *J* = 5.9 Hz, 1H, H-7), 4.20 (quint, *J* = 7.2 Hz, 1H, H-9), 4.01 (q, *J* = 6.7 Hz, 1H, H-12'), 3.96 (q, *J* = 6.8 Hz, 1H, H-12), 3.45 (ddd, *J* = 13.6, 9.9, 8.7 Hz, 1H, H-6a'), 3.40-3.33 (m, 1H, H-6a), 3.33 (q, *J* = 7.0 Hz, 1H, H-9'), 3.20-3.17 (m, 1H, H-6b), 3.14 (q, *J* = 6.3 Hz, 1H, H-3'), 3.02 (q, *J* = 6.2 Hz, 1H, H-3), 2.99-2.85 (m, 2H, H-5, H-6b'), 2.79 (t, *J* = 9.7 Hz, 1H, H-4a'), 2.76-2.67 (m, 2H, H-4a, H-5'), 2.24-2.16 (m, 1H, H-4b), 2.08 (dd, *J* = 9.5, 8.2 Hz, 1H, H-4b'), 1.72 (bs, 6H, 3NH_2), 1.46 (9H, *t*Bu), 1.30 (d, *J* = 7.0 Hz, 3H, H-13'), 1.29 (d, *J* = 6.6 Hz, 3H, H-13), 1.28 (d, *J* = 7.1 Hz, 3H, H-14), 1.24 (d, *J* = 6.9 Hz, 3H, H-14'), 0.98 (d, *J* = 6.3 Hz, 3H, H-11'), 0.90 (d, *J* = 6.2 Hz, 3H, H-11); **^{13}C NMR** (101 MHz, $CDCl_3$) δ 175.6 (C, C-8'), 174.8 (C, C-1'), 173.5 (C, C-1), 171.8 (C, C-8), 144.1 (C, Ar), 143.8 (C, Ar), 128.2 (2CH, Ar), 127.6 (CH, Ar), 127.5 (CH, Ar), 126.81 (CH, Ar), 126.77 (CH, Ar), 81.9 (C, *t*Bu), 68.2 (C, C-2), 66.4 (C, C-2'), 64.4 (CH, C-3), 63.5 (CH, C-3'), 54.9 (CH, C-12), 54.2 (CH, C-12'), 50.9 (CH, C-9'), 49.1 (CH, C-9), 46.9 (CH_2, C-4), 46.3 (CH_2, C-4'), 43.9 (CH, C-5), 43.2 (CH, C-5'), 39.2 (CH_2, C-6), 37.7 (CH_2, C-6'), 28.2 (CH_3, *t*Bu), 21.6 (CH_3, C-14'), 18.5 (CH_3, C-14), 12.7 (CH_3, C-11), 12.5 (CH_3, C-11'), 11.4 (CH_3, C-13), 10.4 (CH_3, C-13'); **MS** (ESI+) m/z, (%): 340.0 (340.2) (9, $[M+2H-C_4H_8]^{2+}$), 368.0 (368.2) (14, $[M+2H]^{2+}$), 735 (100, $[M+H]^+$), 757 (20, $[M+Na]^+$), 1469 (7, $[2M+H]^+$), 1491 (22, $[2M+Na]^+$); **HRMS** (ESI+) m/z: $[M+H]^+$ Calcd for $C_{40}H_{63}N_8O_5$ 735.4916; Found 735.4917.

α/γ-Peptide Fmoc(Ala-(*R*,*R*,*R*,*R*)^{P}AAMP)$_3$O*t*Bu (Fmoc-6AR^{P}A)

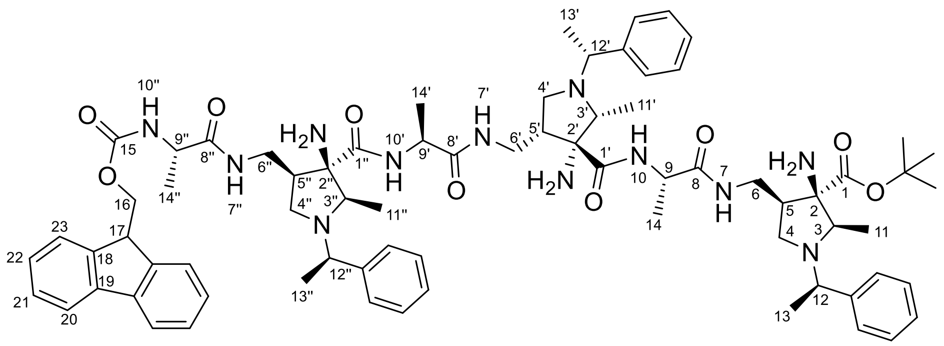

Prepared according to general **procedure B** from dipeptide **Fmoc-2AR^{P}A** (38 mg, 0.059 mmol), DIPEA (41 µL, 0.234 mmol), free amine **NH_2-4AR^{P}A** (43 mg, 0.070 mmol) and HATU (27 mg, 0.070 mmol). The residue was purified by flash chromatography (EtOAc/MeOH, 10:1 to 1:1 gradient) to afford 64 mg (85%) of protected hexapeptide **Fmoc-6AR^{P}A** as an off-white solid.

Mp: 140-142 °C; $[\alpha]_D^{20}$: –55.5 (c 0.2, $CHCl_3$); **IR** ν[cm^{-1}]: 666, 698, 753, 784, 847, 884, 944, 972, 1032, 1047, 1070, 1127, 1152, 1238, 1338, 1369, 1388, 1449, 1495, 1514, 1562, 1601, 1637, 1681, 1720, 2872, 2929, 2972, 3064, 3087, 3107, 3295, 3365; **^{1}H NMR** (600 MHz, $CDCl_3$) δ 8.22 (d, *J* = 9.0 Hz, 1H, H-10'), 8.11 (d, *J* = 7.8 Hz, 1H, H-10), 7.75 (dd, *J* = 7.6, 1.0 Hz, 2H, H-20), 7.61-7.55 (m, 2H, H-23), 7.40-7.19 (m, 19H, Ar, H-21, H-22, H-7'), 7.15-7.10 (m, 1H, Ar), 7.14 (t, *J* = 7.3 Hz, 1H, H-7), 5.74 (d, *J* = 7.5 Hz, 1H, H-10''), 4.40 (quint, *J* = 7.2 Hz, 1H, H-9''), 4.34 (d, *J* = 7.5 Hz, 2H, H-16), 4.22 (dq, *J* = 8.9, 7.1 Hz, 1H, H-9'), 4.18 (t, *J* = 7.2 Hz, 1H, H-17), 4.13 (quint, *J* = 7.3 Hz, 1H, H-9), 4.02 (q, *J* = 6.6 Hz, 1H, H-12''), 4.00 (q, *J* = 6.7 Hz, 1H, H-12'), 3.85 (q, *J* = 6.7 Hz, 1H, H-12), 3.61-3.51 (m, 2H, H-6a', H-6a''), 3.30 (dt, *J* = 14.1, 6.9 Hz, 1H, H-6a), 3.14-3.08 (m, 1H, H-6b), 3.00-2.90 (m, 4H, H-3'', H-5'', H-3', H-5'), 2.98 (q, *J* = 6.3 Hz, 1H, H-3), 2.86-2.81 (m, 1H, H-6b'), 2.76-2.66 (m, 4H, H-6b'', H-5, H-4a'', H-4a'), 2.60 (t, *J* = 9.7 Hz, 1H, H-4a), 2.22-2.18 (m, 1H, H-4b), 2.09-2.03 (m, 2H, H-4b'', H-4b'), 1.85 (bs, 6H, 3NH_2), 1.39 (s, 9H, *t*Bu), 1.29 (d, *J* = 7.1 Hz, 3H, H-14), 1.25 (d, *J* = 7.0 Hz, 3H, H-14'), 1.22 (d, *J* = 6.7 Hz, 3H, H-13'), 1.20 (d, *J* = 7.1 Hz, 3H, H-13''), 1.19 (d, *J* = 6.6 Hz, 3H, H-13), 1.18 (d, *J* = 6.9 Hz, 3H, H-14''), 1.05 (d, *J* = 6.2 Hz, 3H, H-11'), 0.96 (d, *J* = 6.3 Hz, 3H, H-11''), 0.83 (d, *J* = 6.2 Hz, 3H, H-11); **^{13}C NMR** (151 MHz, $CDCl_3$) δ 175.1 (C, C-1''), 174.9 (C, C-1'), 173.3 (C, C-1), 172.3 (C, C-8''), 172.2 (C, C-8), 172.1 (C, C-8'), 155.9 (C, C-15), 144.3 (C, Ar), 143.89/143.86 (C, C-18), 143.61 (C, Ar), 143.57 (C, Ar), 141.4 (C, C-19), 128.33 (CH, Ar), 128.27 (CH, Ar), 128.2 (CH, Ar), 127.9 (CH, C-21), 127.45 (2CH, Ar), 127.42 (CH, Ar), 127.2 (CH, C-22), 126.9 (CH, Ar), 126.8 (CH, Ar), 126.7 (CH, Ar), 125.3/125.2 (CH, C-23), 120.11/120.10 (CH, C-20), 81.5 (C, *t*Bu), 67.9 (C, C-2), 67.2 (CH_2, C-16), 66.14 (C, C-2'), 66.10 (C, C-2''), 64.4 (3CH, C-3, C-3', C-3''), 55.2 (CH, C-12), 53.8 (CH, C-12'), 53.6 (CH, C-12''), 49.9 (CH, C-9''), 49.5 (CH, C-9), 48.7 (CH, C-9'), 47.3 (CH_2, C-4), 47.2 (CH, C-17), 46.4 (CH_2, C-4'), 46.3 (CH_2, C-4''), 43.8 (CH, C-5''), 43.6 (CH, C-5),

43.4 (CH, C-5'), 39.2 (CH_2, C-6), 38.3 (CH_2, C-6'), 38.0 (CH_2, C-6''), 28.1 (CH_3, *t*Bu), 19.6 (CH_3, C-14''), 18.5 (CH_3, C-14), 18.1 (CH_3, C-14'), 12.9 (CH_3, C-11), 12.3 (CH_3, C-11'), 12.1 (CH_3, C-11''), 11.9 (CH_3, C-13), 9.9 (CH_3, C-13'), 9.6 (CH_3, C-13''); **MS** (ESI+) m/z, (%): 411.0 (411.3) (38, $[M+3H-C_4H_8]^{3+}$), 429.7 (429.9) (95, $[M+3H]^{3+}$), 564.0 (564.4) (48, $[M+2H-C_4H_8-PhCH=CH_2]^{2+}$), 592.0 (592.4) (35, $[M+2H-PhCH=CH_2]^{2+}$), 644.0 (644.4) (100, $[M+2H]^{2+}$), 655.0 (655.4) (21, $[M+H+Na]^{2+}$), 1287 (13, $[M+H]^+$), 1309 (8, $[M+Na]^+$); **HRMS** (ESI+) m/z: $[M+H]^+$ Calcd for $C_{73}H_{99}N_{12}O_9$ 1287.7653; Found 1287.7649.

α/γ-Peptide Ac(Ala-(*R*,*R*,*R*,*R*)^{P}AAMP)$_3$O*t*Bu (Ac-6AR^{P}A)

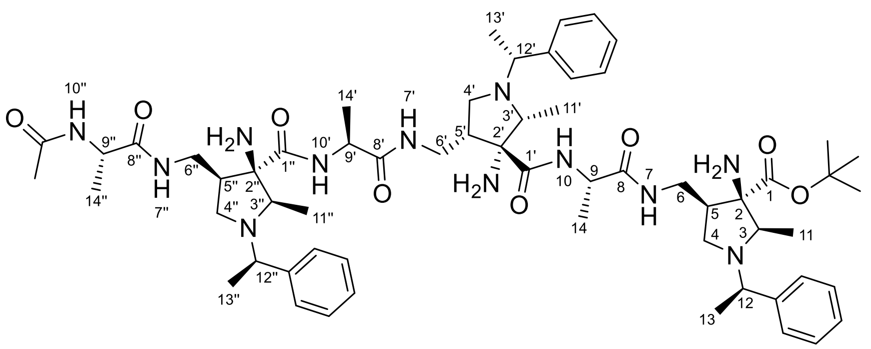

Prepared according to general **procedure B** from dipeptide **Ac-2AR^{P}A** (80 mg, 0.175 mmol) using DIPEA (122 µL, 0.700 mmol), free amine **NH$_2$-4AR^{P}A** (153 mg, 0.208 mmol) and HATU (73 mg, 0.193 mmol). The residue was purified by flash chromatography (EtOAc/MeOH, 10:1 to 1:1 gradient) to afford 180 mg (93%) of protected hexapeptide **Ac-6AR^{P}A** as an off-white solid.

Mp: 197-200 °C; $[\alpha]_D^{20}$: –71.6 (c 0.2, $CHCl_3$); **IR** ν[cm^{-1}]: 646, 699, 732, 784, 847, 911, 1026, 1153, 1238, 1370, 1390, 1449, 1495, 1510, 1601, 1649, 1723, 2853, 2872, 2929, 2970, 3029, 3061, 3086, 3299; **^{1}H NMR** (600 MHz, $CDCl_3$) δ 8.22 (d, *J* = 9.2 Hz, 1H, H-10'), 8.07 (d, *J* = 7.9 Hz, 1H, H-10), 7.71 (bs, 1H, H-7'), 7.36-7.28 (m, 13H, Ar, H-7, H-7''), 7.27-7.20 (m, 3H, Ar), 7.20-7.14 (m, 1H, Ar), 6.50 (d, *J* = 7.6 Hz, 1H, H-10''), 4.66 (quint, *J* = 7.2 Hz, 1H, H-9''), 4.17 (dq, *J* = 9.2, 7.0 Hz, 1H, H-9'), 4.13 (quint, *J* = 7.5 Hz, 1H, H-9), 4.01 (q, *J* = 6.6 Hz, 1H, H-12'), 3.99 (q, *J* = 6.6 Hz, 1H, H-12''), 3.90 (q, *J* = 6.7 Hz, 1H, H-12), 3.62-3.51 (m, 2H, H-6a', H-6a''), 3.36 (dt, *J* = 12.8, 6.2 Hz, 1H, H-6a), 3.11-3.05 (m, 1H, H-6b), 3.05 (d, *J* = 6.3 Hz, 1H, H-3), 2.97-2.87 (m, 4H, H-3', H-3'', H-5', H-5''), 2.80 (dt, *J* = 14.5, 4.0 Hz, 1H, H-6b'), 2.73-2.66 (m, 4H, H-4a, H-4a', H-4a'', H-6b''), 2.65-2.59 (m, 1H, H-5), 2.33 (t, *J* = 7.9 Hz, 1H, H-4b), 2.06-2.01 (m, 2H, H-4b', H-4b''), 1.92 (s, 3H, Ac), 1.87 (s, 6H, $3NH_2$), 1.45 (s, 9H, *t*Bu), 1.28 (d, *J* = 7.1 Hz, 3H, H-14), 1.27 (d, *J* = 6.6 Hz, 3H, H-13), 1.21 (d, *J* = 7.1 Hz, 3H, H-14'), 1.20 (d, *J* = 6.7 Hz, 3H, H-13''), 1.18 (d, *J* = 6.6 Hz, 3H, H-13'), 1.10 (d, *J* = 6.8 Hz, 3H, H-14''), 1.06 (d, *J* = 6.2 Hz, 3H, H-11'), 0.96 (d, *J* = 6.3 Hz, 3H, H-11''), 0.87 (d, *J* = 6.2 Hz, 3H, H-11); **^{13}C NMR** (151 MHz, $CDCl_3$) δ 175.3 (C, C-

1'), 174.8 (C, C-1''), 173.2 (C, C-1), 172.30 (C, C-8''), 172.29 (C, C-8), 172.2 (C, C-8'), 170.1 (C, Ac), 144.5 (C, Ar), 143.64/143.56 (2C, Ar', Ar''), 128.4/128.3/128.2 (3CH, Ar, Ar', Ar''), 127.5/127.45/127.44 (3CH, Ar, Ar', Ar''), 126.9/126.8/126.7 (3CH, Ar, Ar', Ar''), 81.6 (C, *t*Bu), 68.1 (C, C-2), 66.0 (2C, C-2', C-2''), 64.6 (CH, C-3'), 64.4 (CH, C-3), 64.3 (CH, C-3''), 55.5 (CH, C-12), 53.8 (CH, C-12''), 53.4 (CH, C-12'), 49.6 (CH, C-9), 48.6 (CH, C-9'), 48.4 (CH, C-9''), 47.7 (CH_2, C-4), 46.3/46.1 ($2CH_2$, C-4', C-4''), 44.1 (CH, C-5), 44.0 (CH, C-5'), 43.4 (CH, C-5''), 39.1 (CH_2, C-6), 38.2 (CH_2, C-6'), 37.8 (CH_2, C-6''), 28.1 (CH_3, *t*Bu), 23.4 (CH_3, Ac), 19.2 (CH_3, C-14''), 18.5 (CH_3, C-14), 18.1 (CH_3, C-14'), 13.1 (CH_3, C-11), 12.2 ($2CH_3$, C-11', C-13), 12.1 (CH_3, C-11''), 9.8 (CH_3, C-13'), 9.4 (CH_3, C-13''); **MS** (ESI+) m/z, (%): 422.0 (422.3) (14, $[M+2H-C_4H_8-2PhCH=CH_2]^{2+}$), 474.0 (474.3) (57, $[M+2H-C_4H_8-PhCH=CH_2]^{2+}$), 526.0 (526.3) (34, $[M+2H-C_4H_8]^{2+}$), 554.0 (554.3) (100, $[M+2H]^{2+}$), 565.0 (565.3) (19, $[M+H+Na]^{2+}$), 843 (11, $[M+H-C_4H_8-2PhCH=CH_2]^{+}$), 1107 (7, $[M+H]^+$), 1129 (12, $[M+Na]^+$); **HRMS** (ESI+) m/z: $[M+H]^+$ Calcd for $C_{60}H_{90}N_{12}O_8Na$ 1129.6897; Found 1129.6895.

α/γ-Peptide Ac(Ala-(*R*,*R*,*R*,*R*)AAMP)$_3$O*t*Bu (Ac-6AR^{H}A)

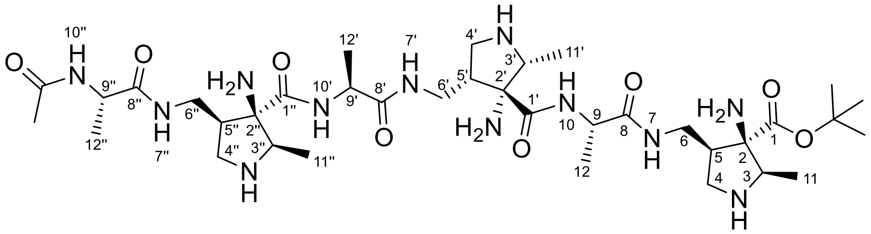

Prepared according to general **procedure D** from hexapeptide **Ac-6AR^{P}A** (22 mg, 0.02 mmol) providing 14 mg (95%) of free base **Ac-6AR^{H}A** as an off-white solid.

Mp: 228-230 °C; $[\alpha]_D^{20}$: −71.4 (c 0.2, MeOH); **IR** (MeOH, 0.025 mm CaF_2 cell) ν[cm^{-1}]: 1089, 1252, 1313, 1371, 1389, 1447, 1524, 1540, 1653, 1723; **^{1}H NMR** (600 MHz, H_2O:D_2O 9:1, CD_3COOD, pH 4.5) δ 8.42 (d, *J* = 6.4 Hz, 1H, H-10), 8.39 (d, *J* = 6.0 Hz, 1H, H-10'), 8.34 (t, *J* = 6.4 Hz, 1H, H-7), 8.28 (t, *J* = 6.3 Hz, 1H, H-7'), 8.25 (d, *J* = 6.3 Hz, 1H, H-10''), 8.17 (t, *J* = 6.4 Hz, 1H, H-7''), 4.28-4.22 (m, 2H, H-9, H-9'), 4.18 (dq, *J* = 7.3, 6.2 Hz, 1H, H-9''), 4.05 (q, *J* = 6.9 Hz, 1H, H-3), 3.920 (q, *J* = 7.0 Hz, 1H, H-3''), 3.918 (q, *J* = 7.0 Hz, 1H, H-3'), 3.623 (dd, *J* = 12.2, 10.7 Hz, 1H, H-4a'), 3.621 (dd, *J* = 11.6, 8.9 Hz, 1H, H-4a), 3.60 (dd, *J* = 12.2, 10.5 Hz, 1H, H-4a''), 3.50 (ddd, *J* = 14.1, 7.3, 6.2 Hz, 1H, H-6a), 3.42 (ddd, *J* = 14.2, 7.3, 6.3 Hz, 1H, H-6a'), 3.39 (ddd, *J* = 14.2, 7.6, 6.4 Hz, 1H, H-6a''), 3.29-3.22 (m, 3H, H-6b, H-6b', H-6b''), 3.184 (dd, *J* = 12.0, 11.1 Hz, 1H, H-4b'), 3.178 (dd, *J* = 12.1, 11.2 Hz, 1H, H-4b''), 3.16 (dd, *J* = 11.6, 10.8 Hz, 1H, H-4b), 3.13-3.08 (m, 1H, H-5), 3.08-3.03 (m, 2H, H-5', H-5''), 2.02 (s, 3H, Ac), 1.49 (s, 9H, *t*Bu), 1.40 (d, *J* = 7.3 Hz, 3H, H-12), 1.37 (d, *J* = 7.4 Hz, 3H, H-12'), 1.31 (d, *J* = 7.4 Hz, 3H, H-12''), 1.29 (d, *J* = 6.9 Hz, 3H, H-11), 1.284 (d, *J* = 7.0 Hz, 3H, H-11''), 1.277 (d, *J* = 7.0 Hz, 3H, H-11'); the

amine NH and NH_2 resonances not detectable likely due to water presaturation; **^{13}C NMR** (151 MHz, D_2O, CD_3COOD, pH 4.5) δ 176.1 (C, C-8''), 175.41 (C, C-8'), 175.38 (C, C-8), 174.7 (C, Ac), 174.0 (C, C-1''), 173.9 (C, C-1'), 172.8 (C, C-1), 85.5 (C, *t*Bu), 66.53 (C, C-2'), 66.47 (C, C-2''), 66.4 (C, C-2), 63.7 (2CH, C-3', C-3''), 63.2 (CH, C-3), 50.9 (2CH, C-9, C-9'), 50.4 (CH, C-9''), 46.8 (CH_2, C-4), 46.6/46.5 ($2CH_2$, C-4', C-4''), 46.4 (CH, C-5), 46.3 (CH, C-5'), 46.1 (CH, C-5''), 37.5 (CH_2, C-6), 37.12 (CH_2, C-6''), 37.06 (CH_2, C-6'), 27.7 (CH_3, *t*Bu), 22.4 (CH_3, Ac), 17.3 ($2CH_3$, C-12', C-12''), 17.2 (CH_3, C-12), 10.7/10.57/10.56 ($3CH_3$, C-11, C-11', C-11''); **MS** (ESI+) m/z, (%): 370.0 (370.2) (100, $[M+2H-C_4H_8]^{2+}$), 381.0 (381.2) (23, $[M+H+Na-C_4H_8]^{2+}$), 392.0 (392.2) (12, $[M+2Na-C_4H_8]^{2+}$), 398.0 (398.3) (29, $[M+2H]^{2+}$), 795 (13, $[M+H]^+$), 817 (100, $[M+Na]^+$); **HRMS** (ESI+) m/z: $[M+H]^+$ Calcd for $C_{36}H_{68}N_{12}O_8$ 817.5012; Found 817.5019.

10) Supplementary References

1. Kapras, V.; Pohl, R.; Císařová, I.; Jahn, U., Asymmetric Domino Aza-Michael Addition/[3 + 2] Cycloaddition Reactions as a Versatile Approach to α,β,γ-Triamino Acid Derivatives. *Org. Lett.* **16**, 1088–1091 (2014).

2. Ma, S., Cao, X., Mak, M., Sadik, A., Walkner, C., Freedman, T. B., Lednev, I. K., Dukor, R. K., Nafie, L. A. Vibrational Circular Dichroism Shows Unusual Sensitivity to Protein Fibril Formation and Development in Solution. *J. Am. Chem. Soc.* **129**, 12364–12365 (2007).

3. Abraham, M. J., Murtola, T., Schulz, R., Páll, S., Smith, J. C., Hess, B., Lindahl, E. GROMACS: High performance molecular simulations through multi-level parallelism from laptops to supercomputers. *SoftwareX* ***1-2***, 19–25 (2015).

4. Frisch, M. J., Trucks, G. W., Schlegel, H. B., Scuseria, G. E., Robb, M. A., Cheeseman, J. R., Scalmani, G., Barone, V., Petersson, G. A., Nakatsuji, H., Li, X., Caricato, M., Marenich, A. V., Bloino, J., Janesko, B. G., Gomperts, R., Mennucci, B., Hratchian, H. P., Ortiz, J. V., Izmaylov, A. F., Sonnenberg, J. L., Williams, Ding, F., Lipparini, F., Egidi, F., Goings, J., Peng, B., Petrone, A., Henderson, T., Ranasinghe, D., Zakrzewski, V. G., Gao, J., Rega, N., Zheng, G., Liang, W., Hada, M., Ehara, M., Toyota, K., Fukuda, R., Hasegawa, J., Ishida, M., Nakajima, T., Honda, Y., Kitao, O., Nakai, H., Vreven, T., Throssell, K., Montgomery Jr., J. A., Peralta, J. E., Ogliaro, F., Bearpark, M. J., Heyd, J. J., Brothers, E. N., Kudin, K. N., Staroverov, V. N., Keith, T. A., Kobayashi, R., Normand, J., Raghavachari, K., Rendell, A. P., Burant, J. C., Iyengar, S. S., Tomasi, J., Cossi, M., Millam, J. M., Klene, M., Adamo, C., Cammi, R., Ochterski, J. W., Martin, R. L., Morokuma, K., Farkas, O., Foresman, J. B., Fox, D. J. *Gaussian 16 Rev. C.01*, Wallingford, CT, (2016).

5. Jorgensen, W. L., Chandrasekhar, J., Madura, J. D., Impey, R. W., Klein, M. L. Comparison of simple potential functions for simulating liquid water. *J. Chem. Phys.* **79**, 926–935 (1983).

6. Hess, B., Bekker, H., Berendsen, H. J. C., Fraaije, J. G. E. M. LINCS: A linear constraint solver for molecular simulations. *J. Comput. Chem.* **18**, 1463–1472 (1997).

7. Bussi, G., Donadio, D., Parrinello, M. Canonical sampling through velocity rescaling. *J. Chem. Phys.* **126**, 014101 (2007).

8. Berendsen, H. J. C., Postma, J. P. M., Gunsteren, W. F. v. DiNola, A., Haak, J. R. Molecular dynamics with coupling to an external bath. *J. Chem. Phys.* **81**, 3684–3690 (1984).

9. Bonomi, M., Bussi, G., Camilloni, C., Tribello, G. A., Banáš, P., Barducci, A., Bernetti, M., Bolhuis, P. G., Bottaro, S., Branduardi, D., Capelli, R., Carloni, P., Ceriotti, M., Cesari, A., Chen, H., Chen, W., Colizzi, F., De, S., De La Pierre, M., Donadio, D., Drobot, V., Ensing, B., Ferguson, A. L., Filizola, M., Fraser, J. S., Fu, H., Gasparotto, P., Gervasio, F. L., Giberti, F., Gil-Ley, A., Giorgino, T., Heller, G. T., Hocky, G. M., Iannuzzi, M., Invernizzi, M., Jelfs, K. E., Jussupow, A., Kirilin, E., Laio, A., Limongelli, V.,

Lindorff-Larsen, K., Löhr, T., Marinelli, F., Martin-Samos, L., Masetti, M., Meyer, R., Michaelides, A., Molteni, C., Morishita, T., Nava, M., Paissoni, C., Papaleo, E., Parrinello, M., Pfaendtner, J., Piaggi, P., Piccini, G., Pietropaolo, A., Pietrucci, F., Pipolo, S., Provasi, D., Quigley, D., Raiteri, P., Raniolo, S., Rydzewski, J., Salvalaglio, M., Sosso, G. C., Spiwok, V., Šponer, J., Swenson, D. W. H., Tiwary, P., Valsson, O., Vendruscolo, M., Voth, G. A., White, A. The PLUMED consortium, Promoting transparency and reproducibility in enhanced molecular simulations. *Nat. Methods* **16**, 670–673 (2019).

10. Cossi, M., Rega, N., Scalmani, G., Barone, V. Energies, structures, and electronic properties of molecules in solution with the C-PCM solvation model. *J. Comput. Chem.* **24**, 669–681 (2003).

11. Jensen, F. Basis Set Convergence of Nuclear Magnetic Shielding Constants Calculated by Density Functional Methods. *J. Chem. Theory Comput.* **4**, 719–727 (2008).

12. Jensen, F. The optimum contraction of basis sets for calculating spin–spin coupling constants. *Theor. Chem. Acc.* **126**, 371–382 (2010).

13. (a) Sheldrick, G. SHELXT - Integrated space-group and crystal-structure determination. *Acta Cryst. A* **A71**, 3–8 (2015). (b) Sheldrick, G. Crystal structure refinement with SHELXL. *Acta Cryst*. **C71**, 3–8 (2015). (c) Parsons, S., Flack, H. D., Wagner, T. Use of intensity quotients and differences in absolute structure refinement. *Acta Cryst. B* **B69**, 249–259 (2013).
