# Supplementary material for: Foldamers controlled by functional triamino acids: structural investigation of α/γ-hybrid oligopeptides
Source: Commun Chem. 2024 May 25;7:114. doi: 10.1038/s42004-024-01201-7 (PMC11128005; doi:10.1038/s42004-024-01201-7)
Supplement: Supplementary file 3 — Description of Additional Supplementary Files [file 42004_2024_1201_MOESM3_ESM.pdf]

Description of Additional Supplementary Files

File name: Supplementary Data 1 **MFmoc-6GR^{P}A**(RH).gif

Description: .gif file of **MFmoc-6GR^{P}A**(RH)

File name: Supplementary Data 2 **MFmoc-6GR^{P}A**(S).gif

Description: .gif file of **MFmoc-6GR^{P}A**(S)

File name: Supplementary Data 3 **MBoc-8GR^{P}A**(RH).gif

Description: .gif file of **MBoc-8GR^{P}A**(RH)

File name: Supplementary Data 4 **MBoc-8GR^{P}A**(S).gif

Description: .gif file of **MBoc-8GR^{P}A**(S)

File name: Supplementary Data 5 **MAc-6GR^{H}A**(RH).gif

Description: .gif file of **MAc-6GR^{H}A**(RH)

File name: Supplementary Data 6 NMR Spectra.pdf

Description: pdfs of NMR spectra of all compounds

File name: Supplementary Data 7 **Fmoc-2GR^{P}A**.cif

Description: .cif file of **Fmoc-2GR^{P}A**

File name: Supplementary Data 8 **NH_2-4GR^{P}A**.cif

Description: .cif file of **NH_2-4GR^{P}A**
