# Supplementary figures and images for: Foldamers controlled by functional triamino acids: structural investigation of α/γ-hybrid oligopeptides
Source: Commun Chem. 2024 May 25;7:114. doi: 10.1038/s42004-024-01201-7 (PMC11128005; doi:10.1038/s42004-024-01201-7)

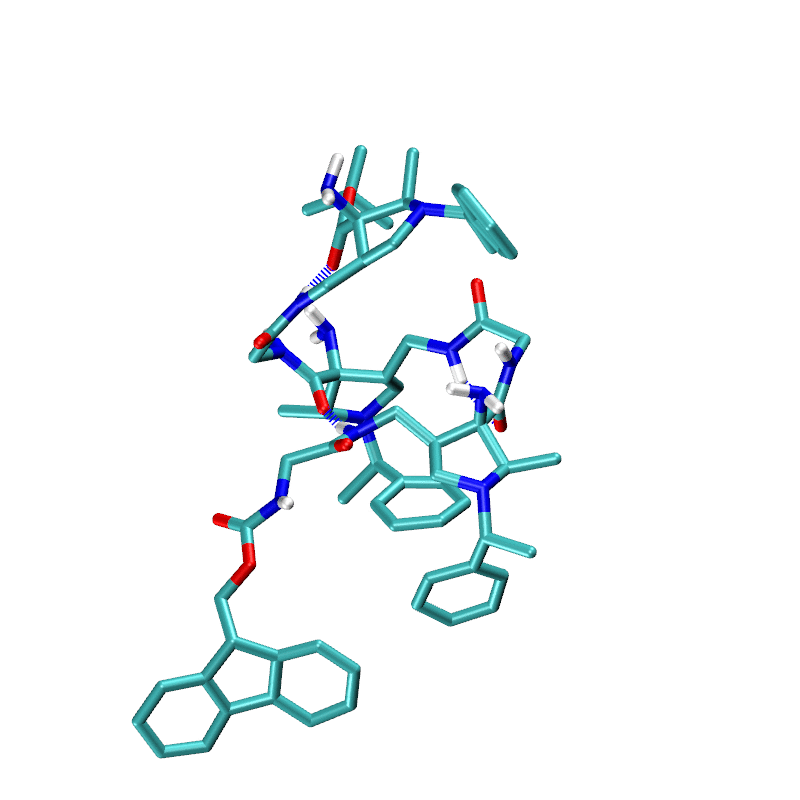

Supplement: Supplementary file 4 — Supplementary data 1 MFmoc-6GRPA(RH) [file 42004_2024_1201_MOESM4_ESM.gif]

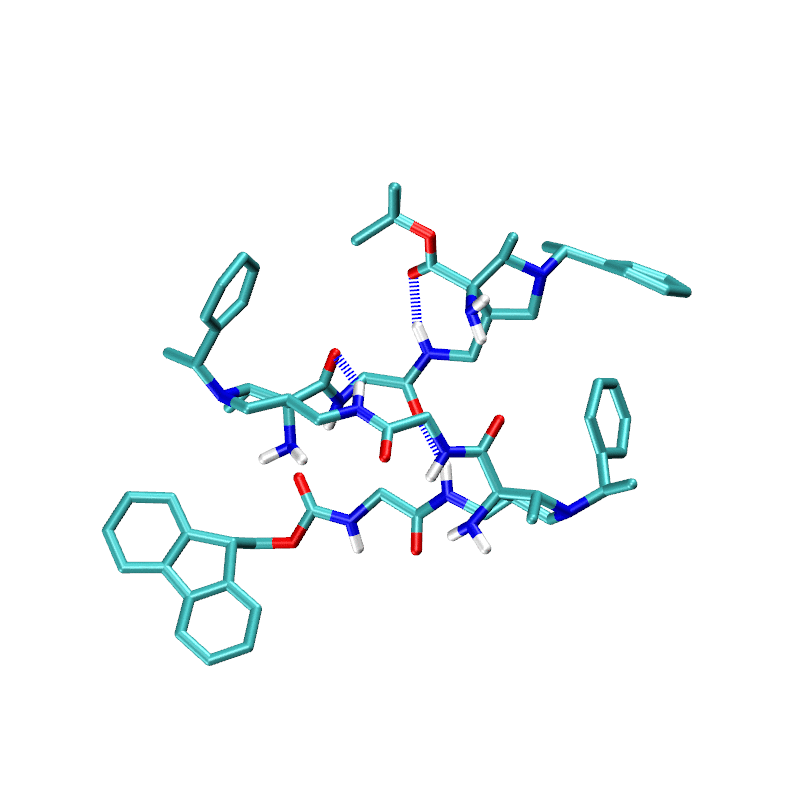

Supplement: Supplementary file 5 — Supplementary data 2 MFmoc-6GRPA(S) [file 42004_2024_1201_MOESM5_ESM.gif]

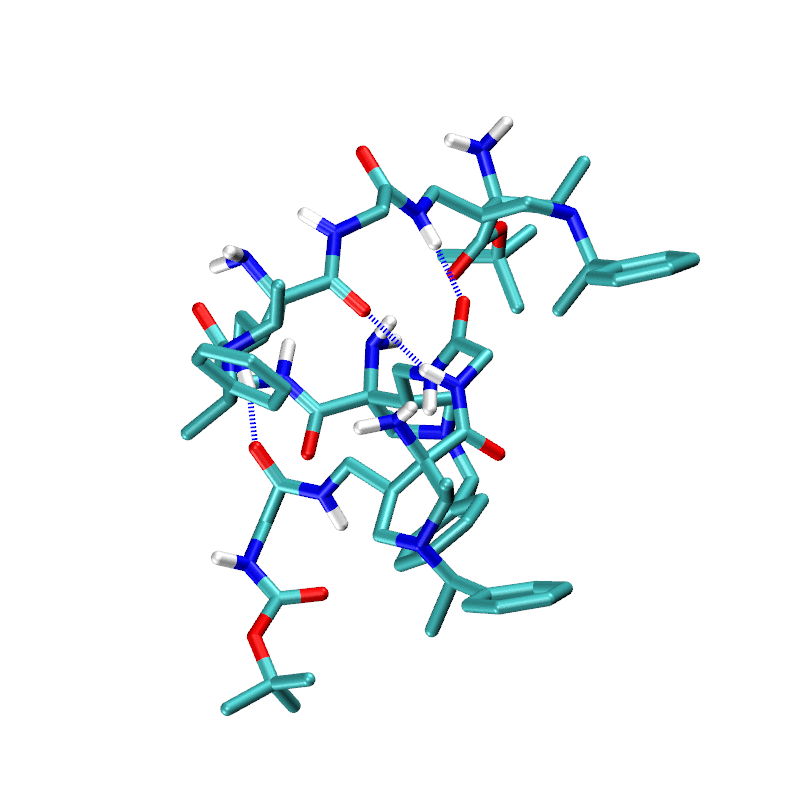

Supplement: Supplementary file 6 — Supplementary data 3 MBoc-8GRPA(RH) [file 42004_2024_1201_MOESM6_ESM.gif]

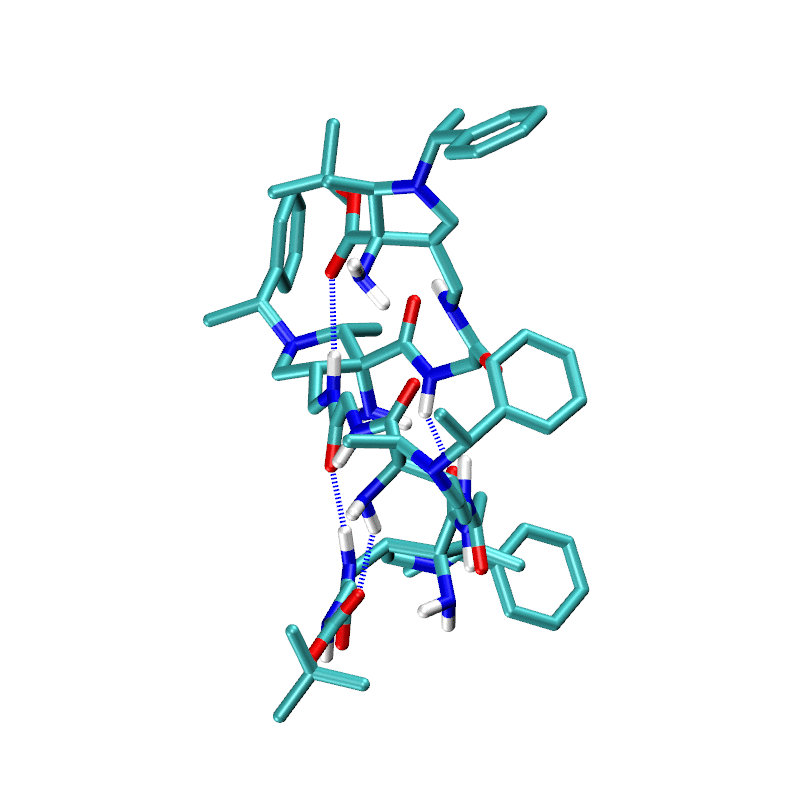

Supplement: Supplementary file 7 — Supplementary data 4 MBoc-8GRPA(S) [file 42004_2024_1201_MOESM7_ESM.gif]

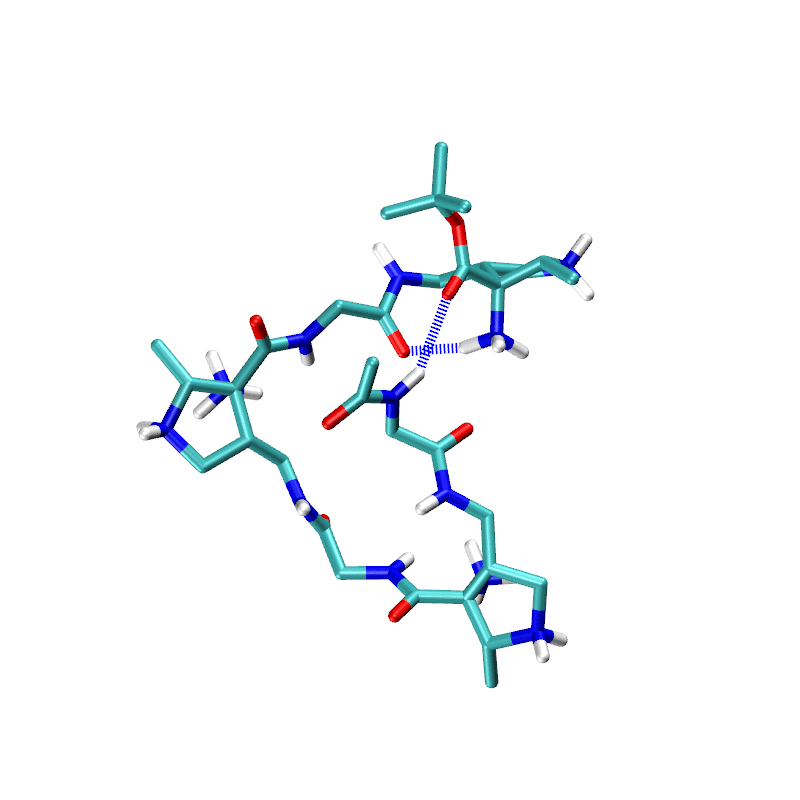

Supplement: Supplementary file 8 — Supplementary data 5 MAc-6GRHA(RH) [file 42004_2024_1201_MOESM8_ESM.gif]
