# Supplementary material for: Foldamers controlled by functional triamino acids: structural investigation of α/γ-hybrid oligopeptides
Source: Commun Chem. 2024 May 25;7:114. doi: 10.1038/s42004-024-01201-7 (PMC11128005; doi:10.1038/s42004-024-01201-7)
Supplement: Supplementary file 9 — Supplementary data 6 NMR Spectra [file 42004_2024_1201_MOESM9_ESM.pdf]

Foldamers Controlled by Functional Triamino Acids: Structural Investigation of α/γ-Hybrid Oligopeptides

David Just[1], Vladimír Palivec[1], Kateřina Bártová[1], Lucie Bednárová[1], Markéta Pazderková[1], Ivana Císařová[2], Hector Martinez-Seara[1]*, Ullrich Jahn[1]*

[1] Institute of Organic Chemistry and Biochemistry, Czech Academy of Sciences, Flemingovo náměstí 2, 16610 Prague 6, Czech Republic

[2] Department of Inorganic Chemistry, Faculty of Science, Charles University in Prague, Hlavova 2030/8, 12843 Prague 2, Czech Republic

NMR Spectra

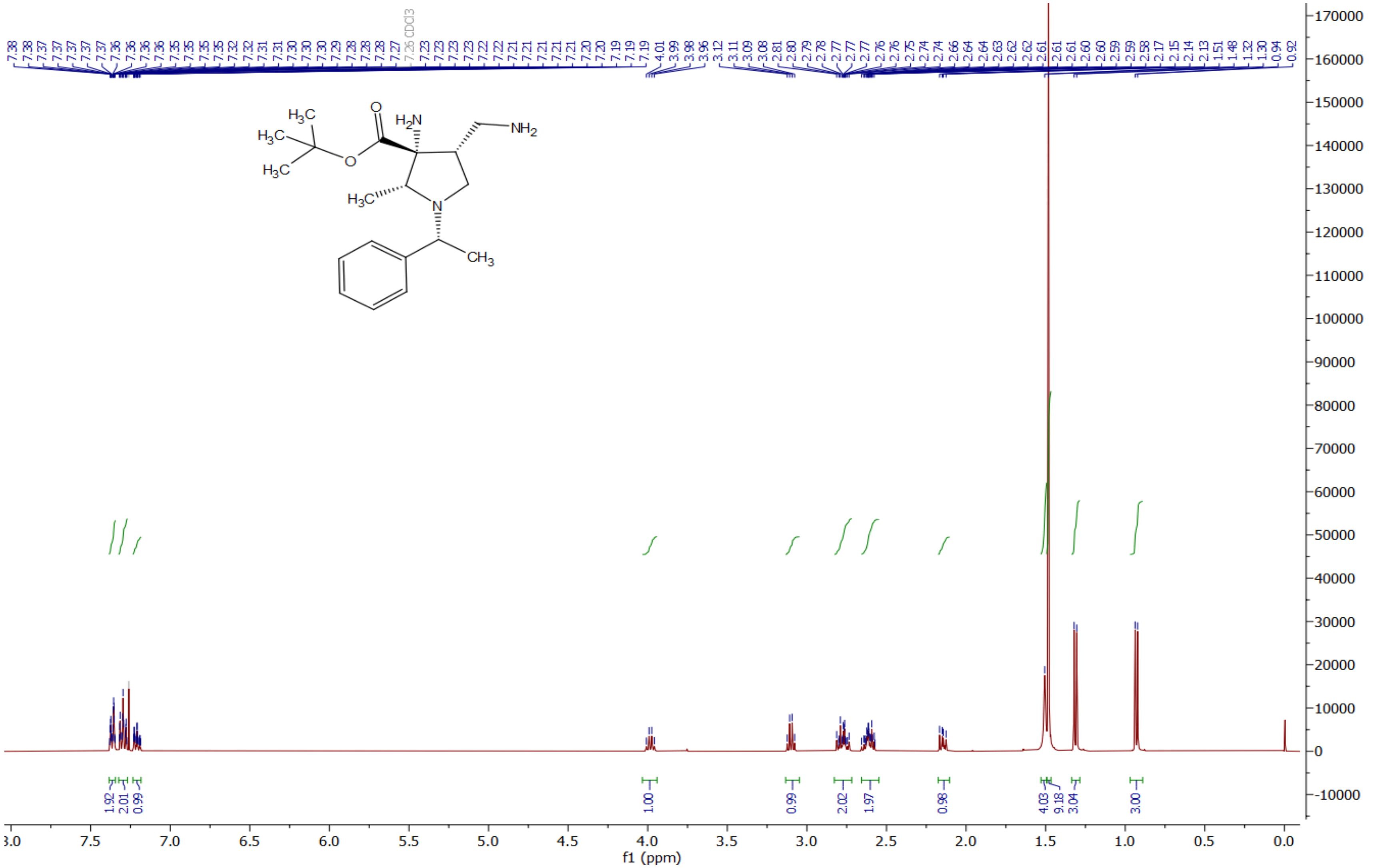

^{1}H-NMR spectrum of *tert*-butyl (2*R*,3*R*,4*R*)-3-amino-4-(aminomethyl)-2-methyl-1-((*R*)-1-phenylethyl)pyrrolidine-3-carboxylate **NH_2-R^{P}A** measured in $CDCl_3$ at 401 MHz.

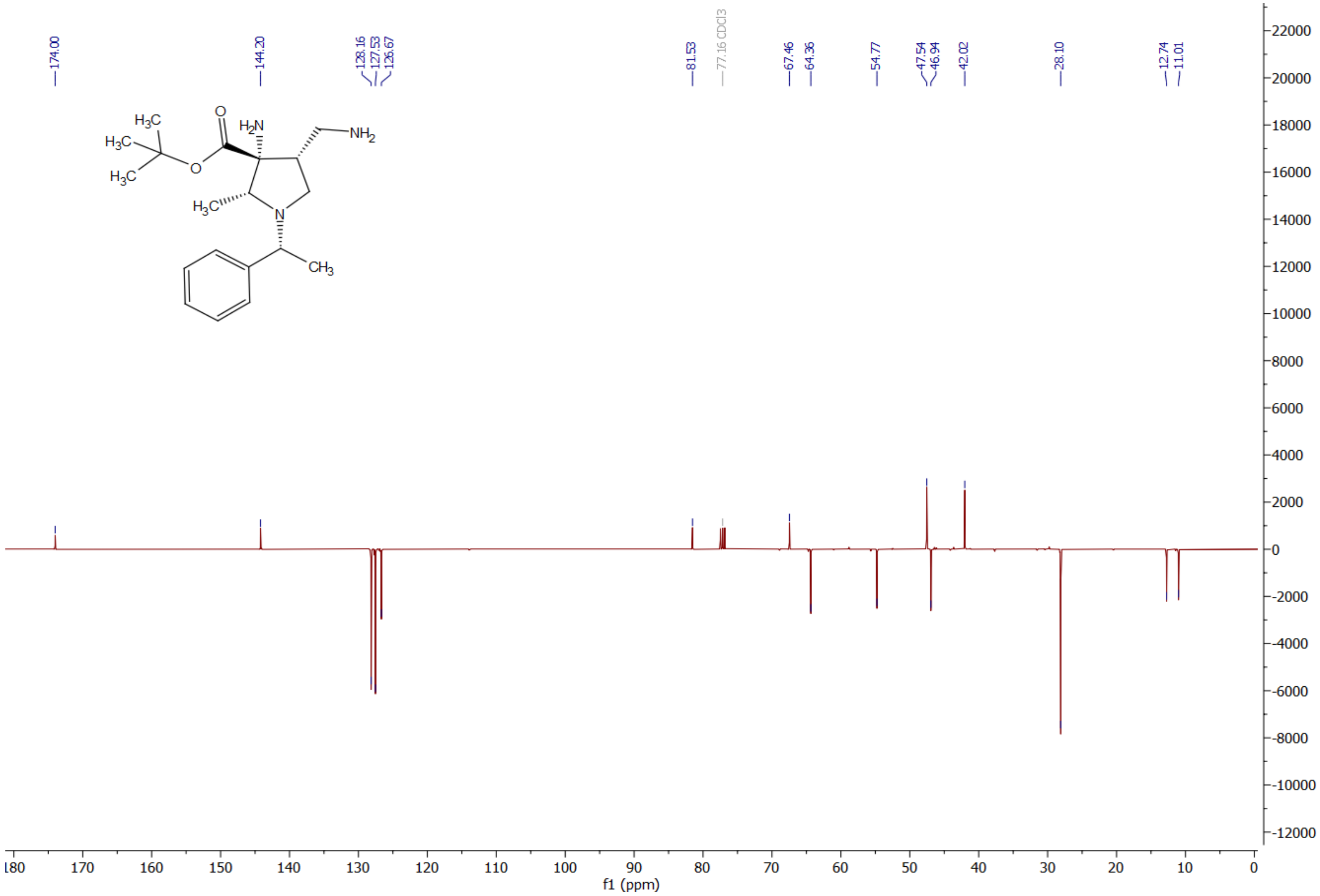

^{13}C-NMR spectrum of *tert*-butyl (2*R*,3*R*,4*R*)-3-amino-4-(aminomethyl)-2-methyl-1-((*R*)-1-phenylethyl)pyrrolidine-3-carboxylate **NH_2-R^{P}A** measured in $CDCl_3$ at 101 MHz.

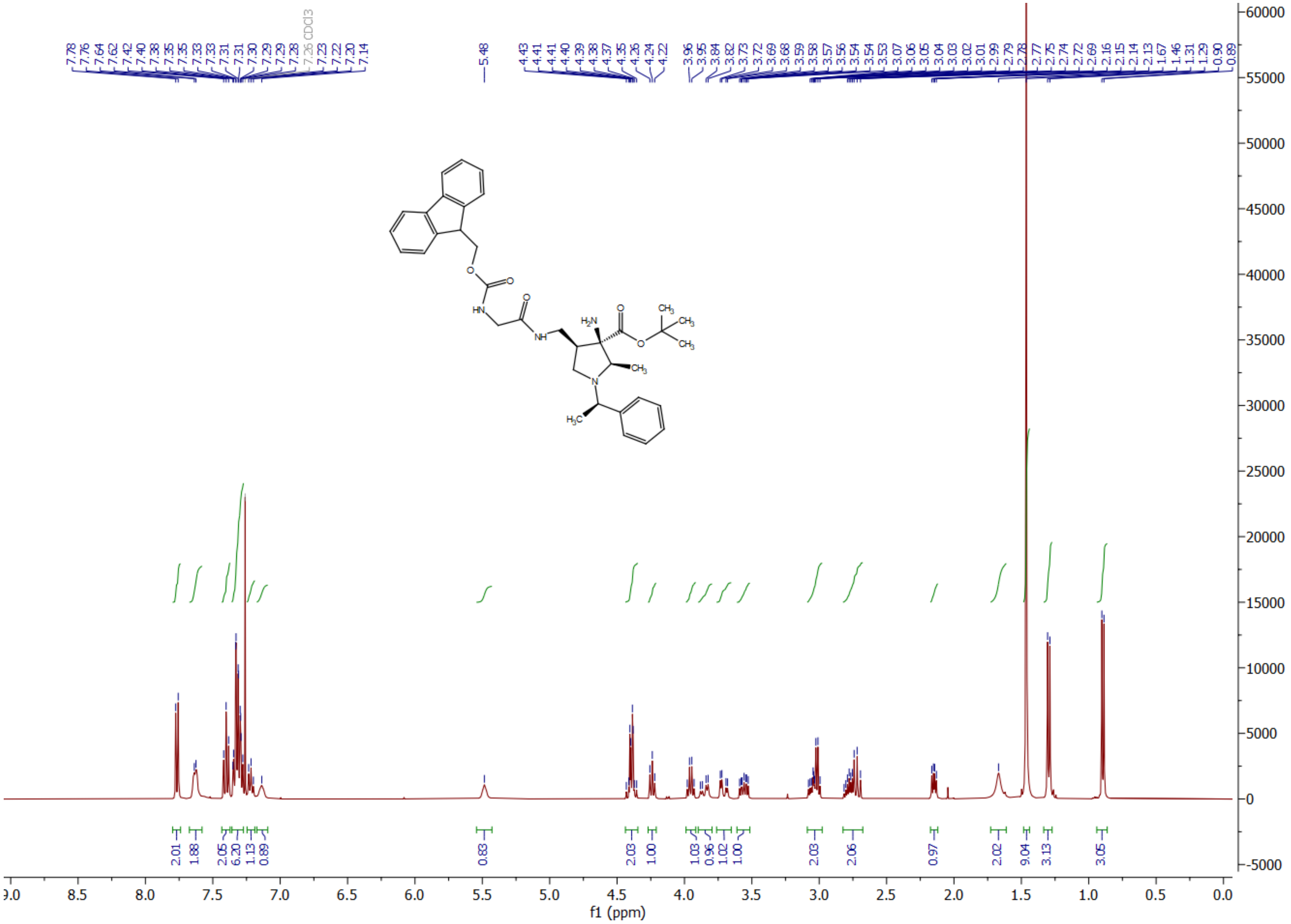

^{1}H-NMR spectrum of α/γ-peptide Fmoc(Gly-(*R*,*R*,*R*,*R*)[P]AAMP)O*t*Bu **Fmoc-2GR[P]A** measured in $CDCl_3$ at 401 MHz.

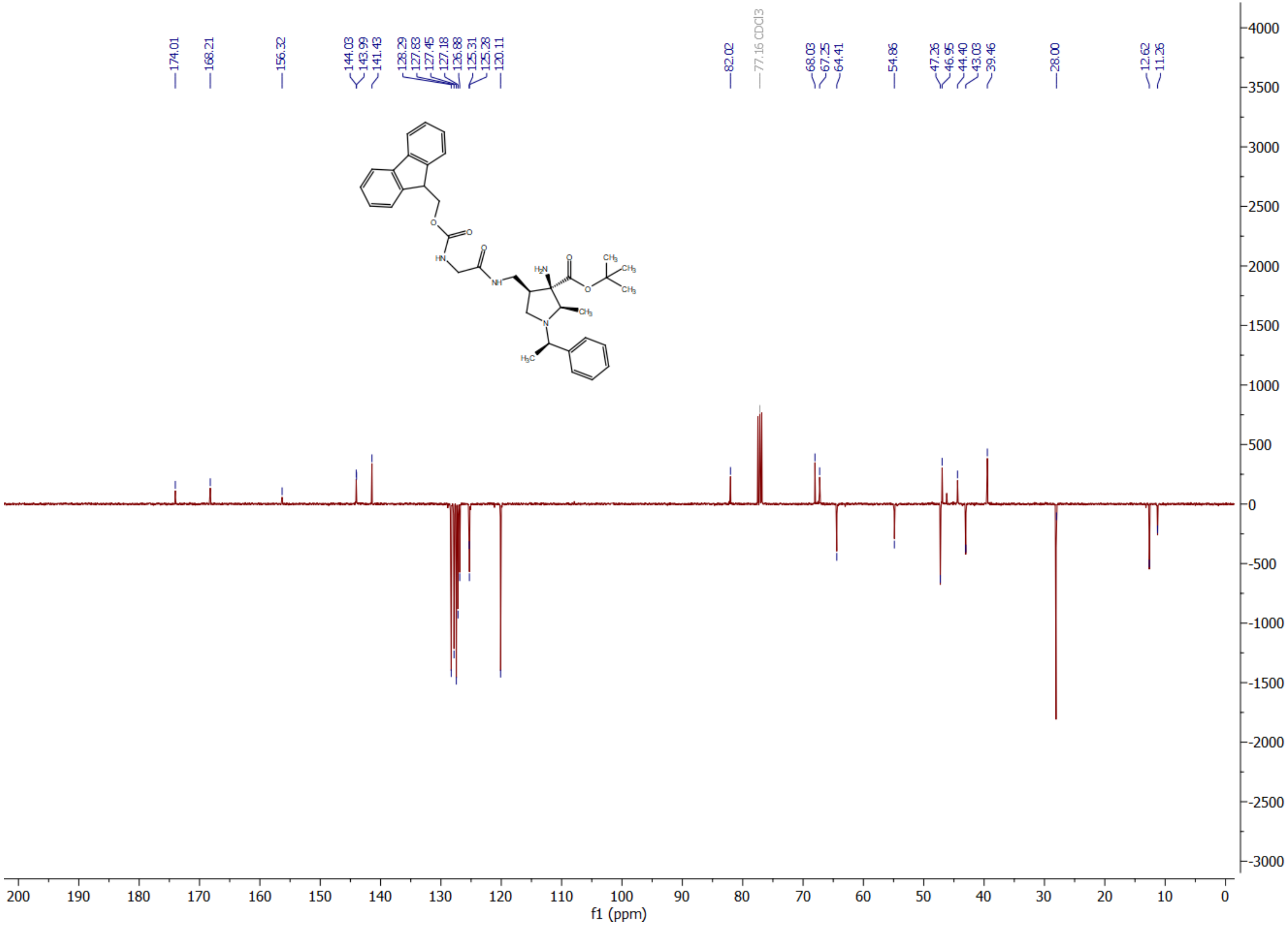

^{13}C-NMR spectrum of α/γ-peptide Fmoc(Gly-(*R*,*R*,*R*,*R*)^{P}AAMP)O*t*Bu **Fmoc-2GR^{P}A** measured in $CDCl_3$ at 101 MHz.

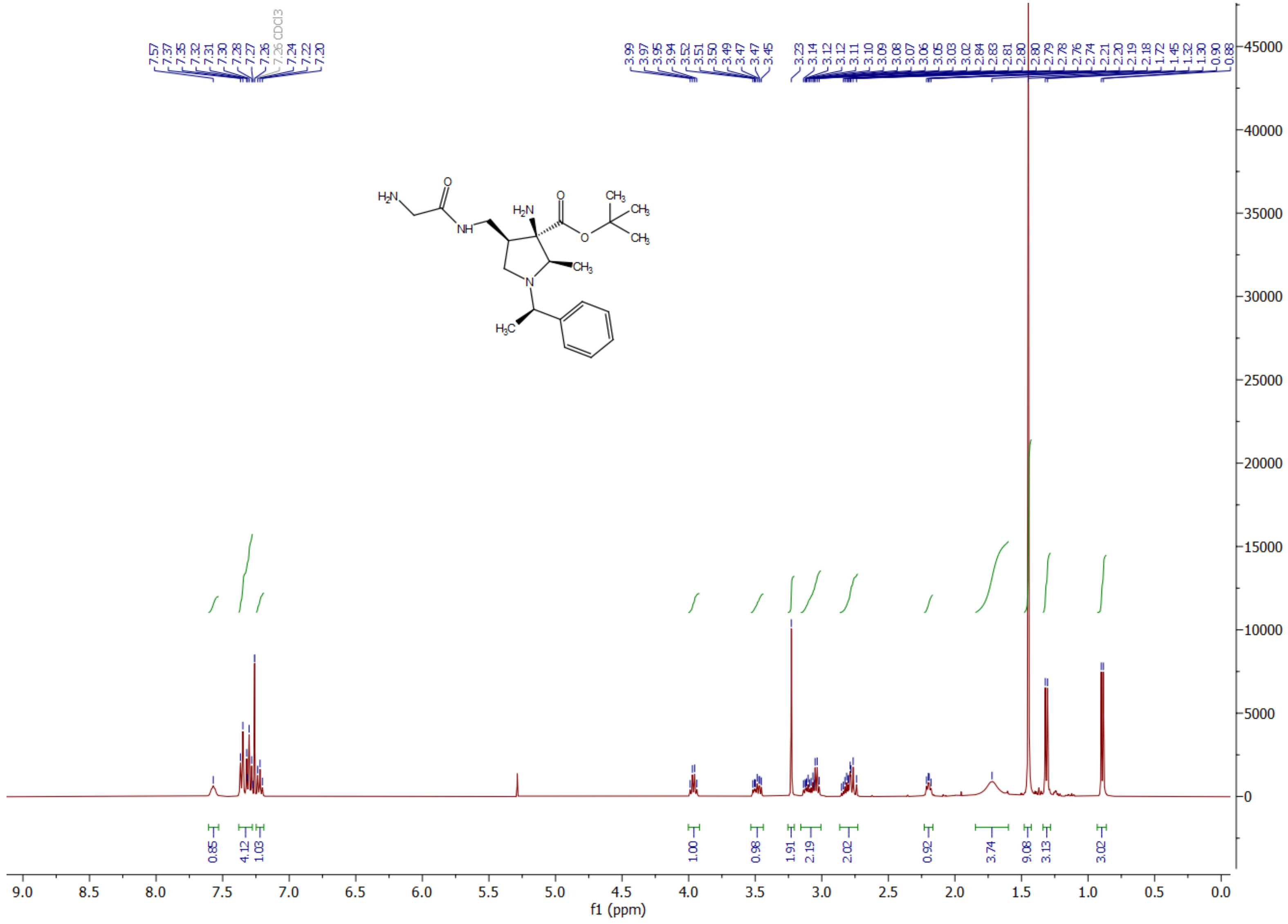

^{1}H-NMR spectrum of α/γ-peptide NH_2(Gly-(*R*,*R*,*R*,*R*)[P]AAMP)O*t*Bu **NH_2-2GR[P]A** measured in $CDCl_3$ at 401 MHz**.**

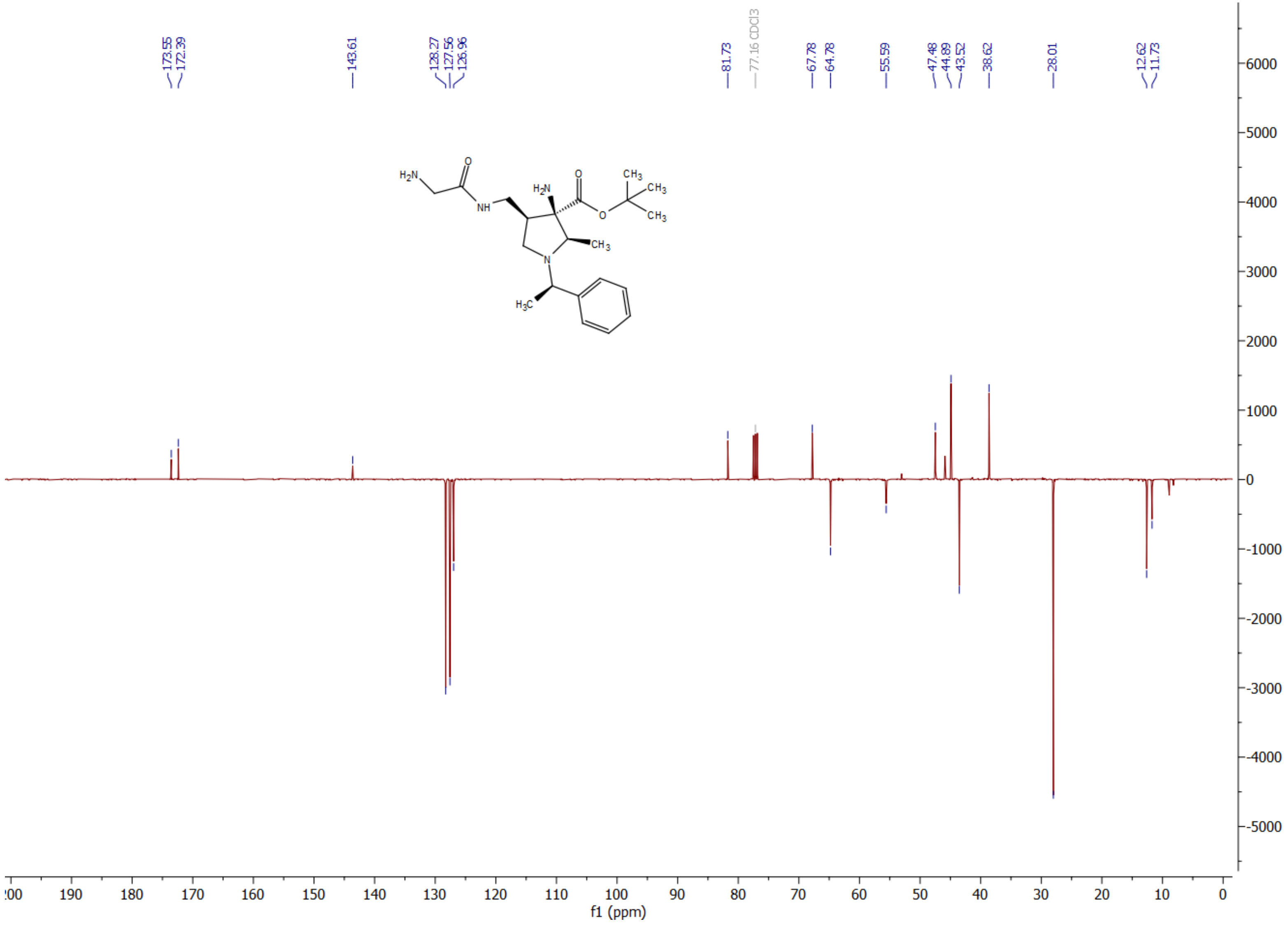

^{13}C-NMR spectrum of α/γ-peptide NH_2(Gly-(*R*,*R*,*R*,*R*)^{P}AAMP)O*t*Bu **NH_2-2GR^{P}A** measured in $CDCl_3$ at 101 MHz.

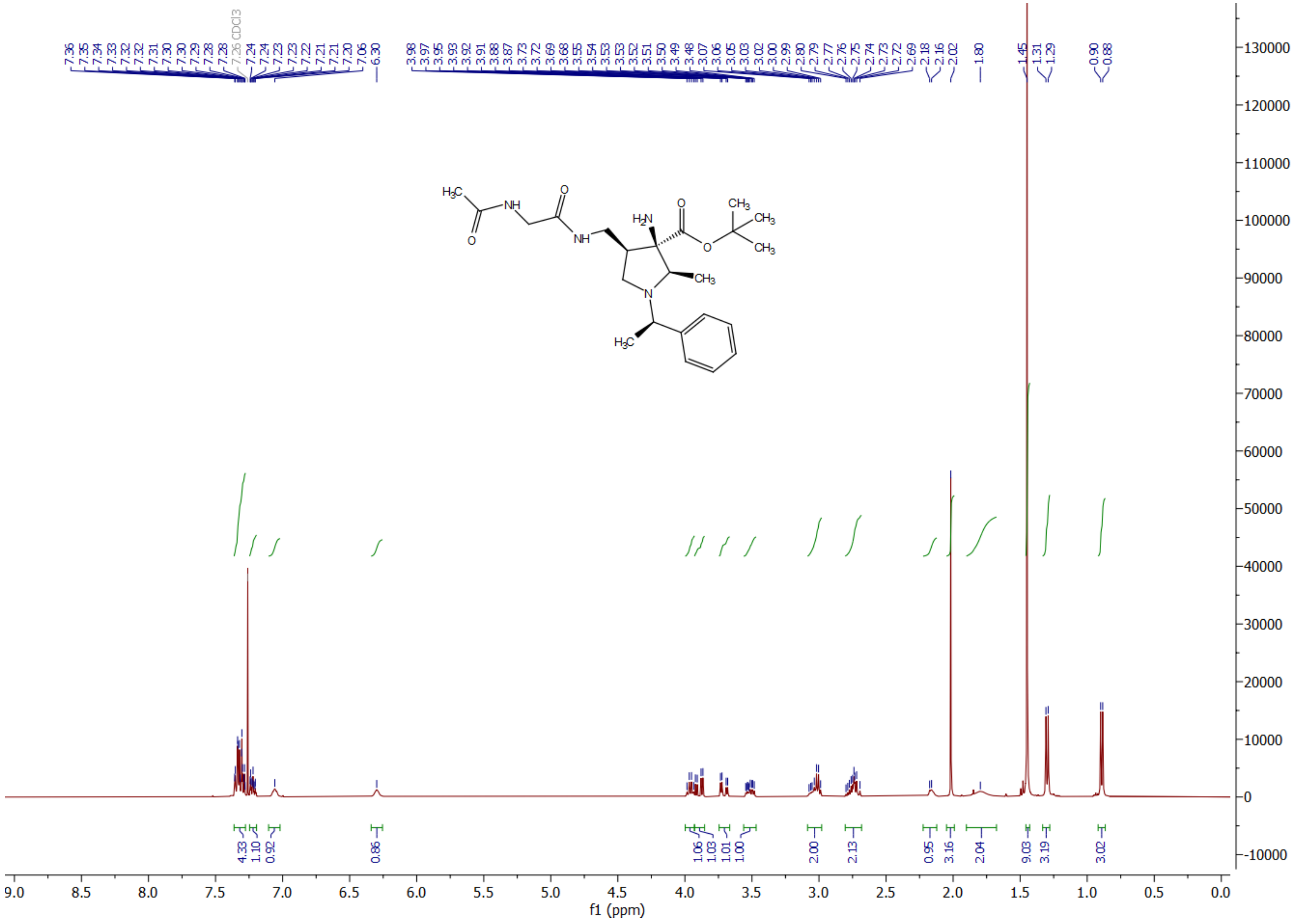

^{1}H-NMR spectrum of α/γ-peptide Ac(Gly-(*R*,*R*,*R*,*R*)[P]AAMP)O*t*Bu **Ac-2GR[P]A** measured in $CDCl_3$ at 401 MHz.

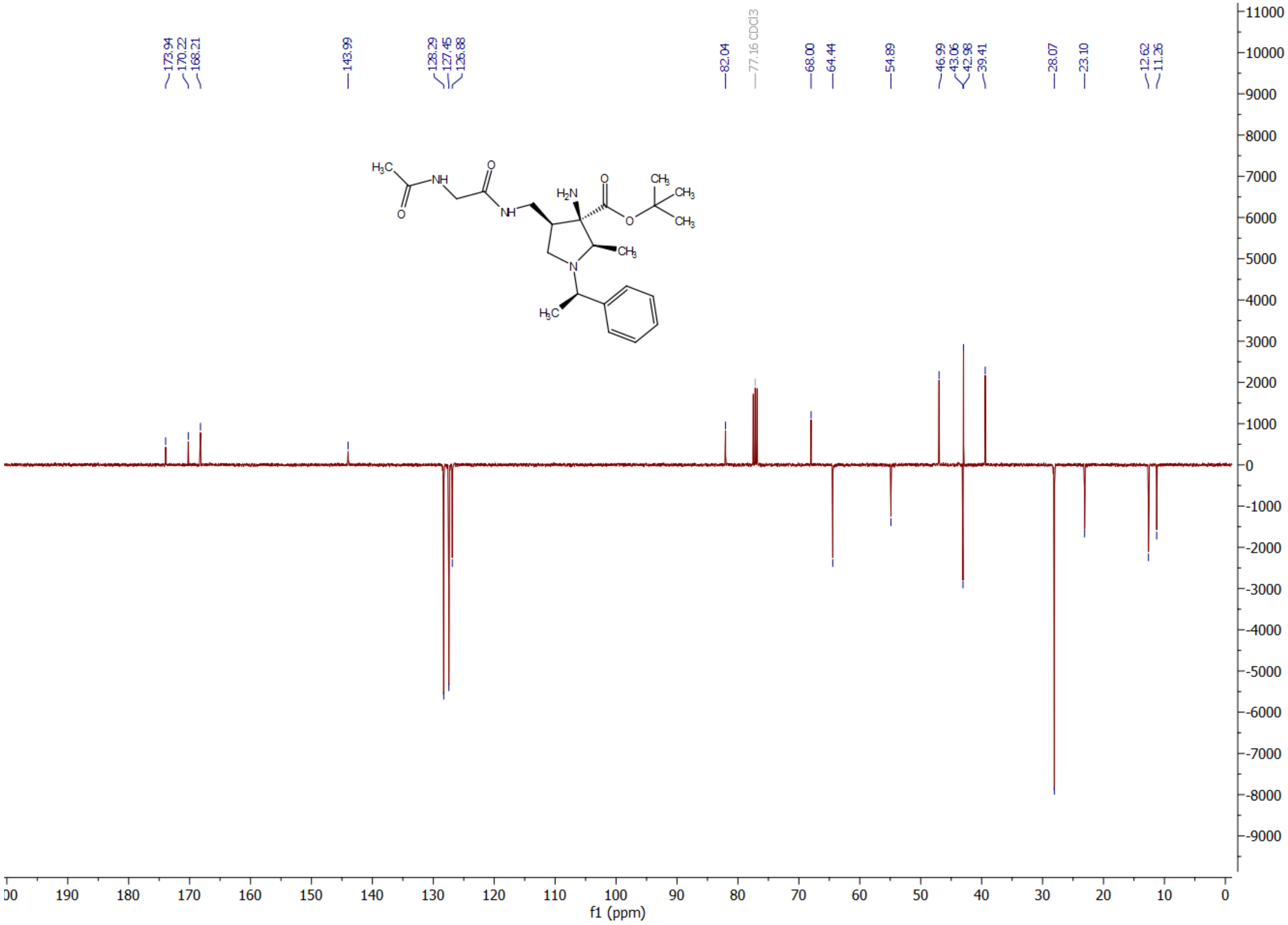

^{13}C-NMR spectrum of α/γ-peptide Ac(Gly-(*R*,*R*,*R*,*R*)[P]AAMP)O*t*Bu **Ac-2GR[P]A** measured in $CDCl_3$ at 101 MHz.

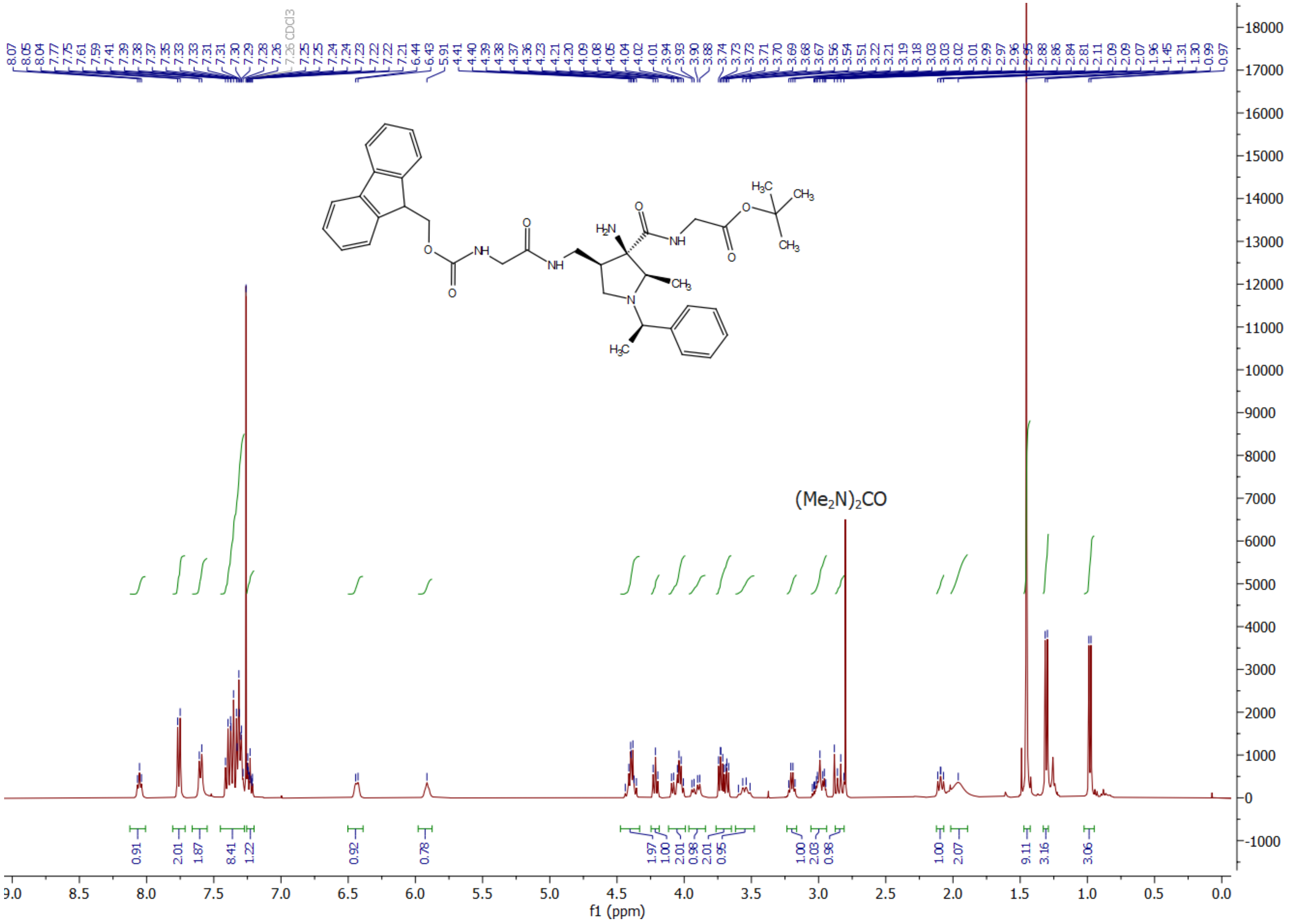

^{1}H-NMR spectrum of α/γ-peptide Fmoc(Gly-(*R*,*R*,*R*,*R*)[P]AAMP-Gly)O*t*Bu **Fmoc-3GR[P]AG** measured in $CDCl_3$ at 401 MHz.

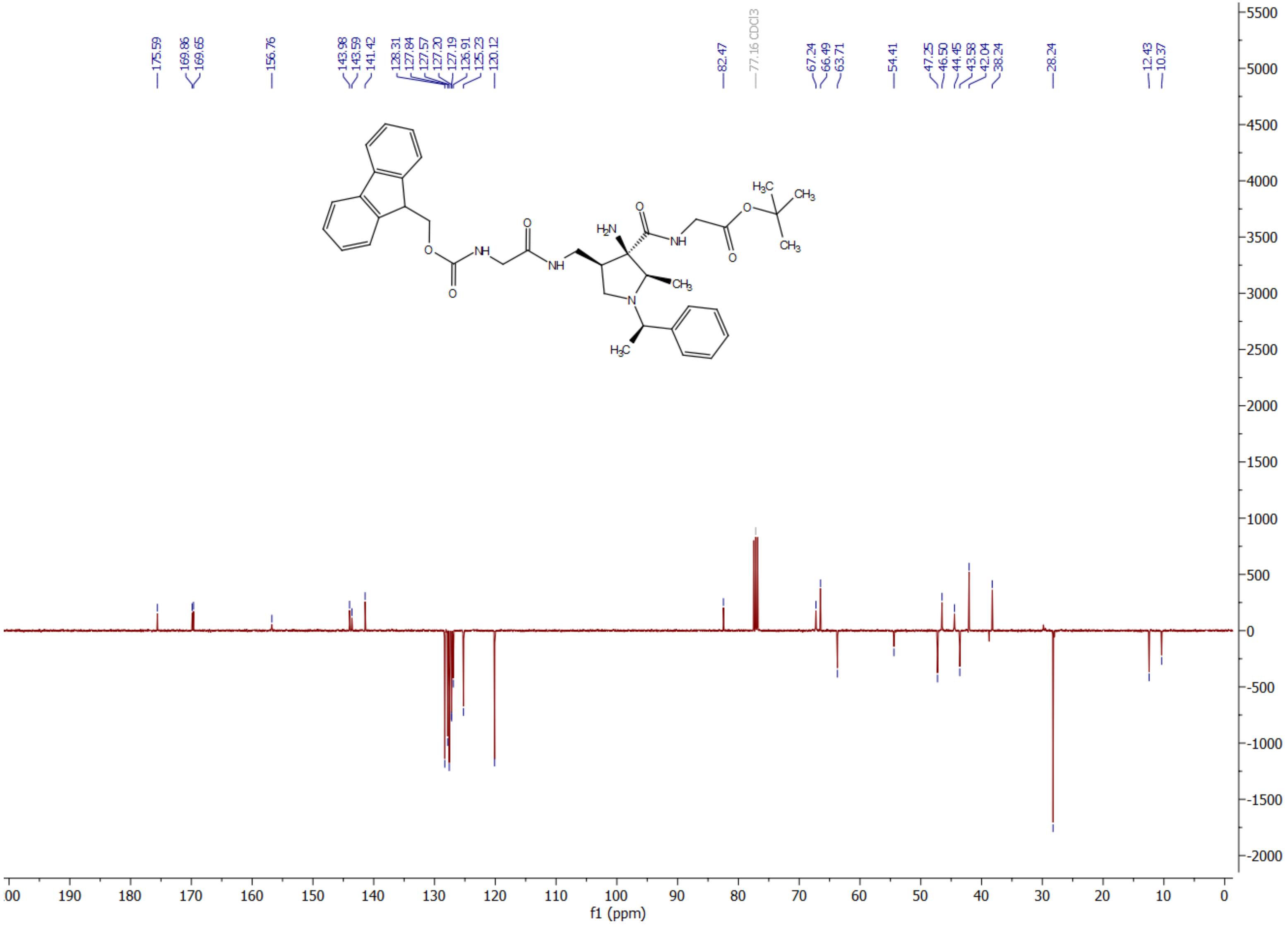

^{13}C-NMR spectrum of α/γ-peptide Fmoc(Gly-(*R*,*R*,*R*,*R*)^{P}AAMP-Gly)O*t*Bu **Fmoc-3GR^{P}AG** measured in $CDCl_3$ at 101 MHz.

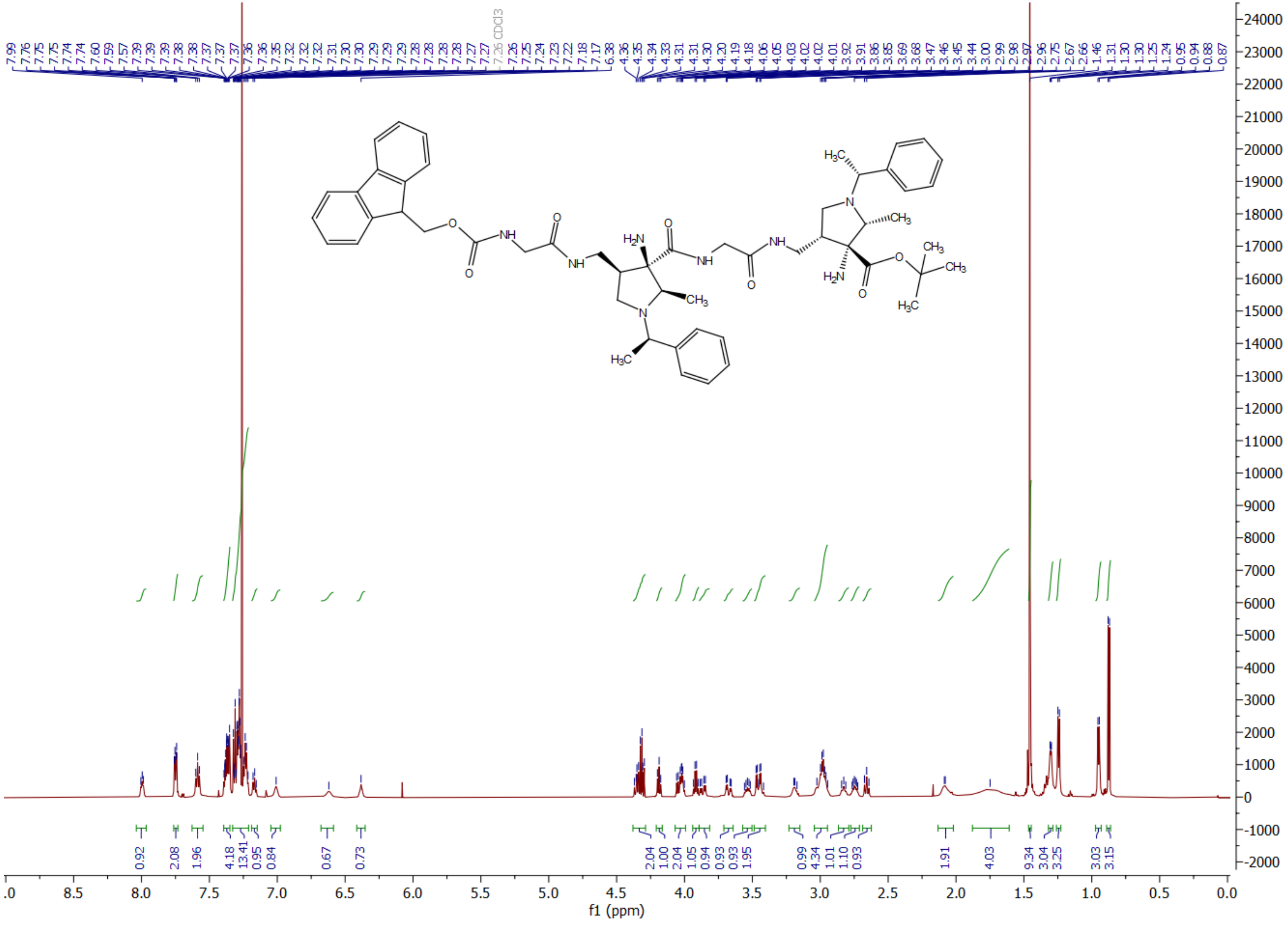

^{1}H-NMR spectrum of α/γ-peptide Fmoc(Gly-(*R*,*R*,*R*,*R*)[P]AAMP)$_2$O*t*Bu **Fmoc-4GR[P]A** measured in $CDCl_3$ at 600 MHz.

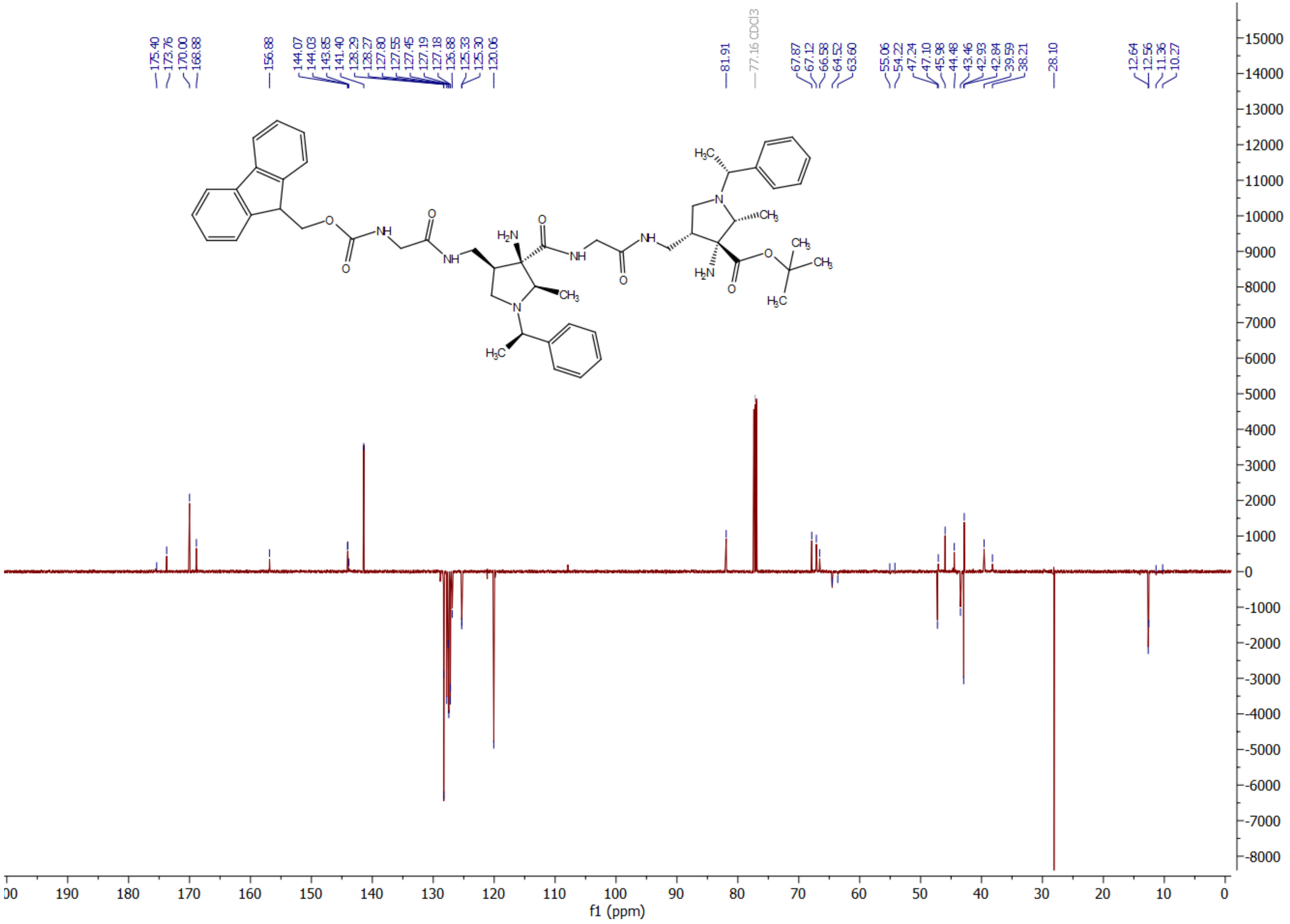

^{13}C-NMR spectrum of α/γ-peptide Fmoc(Gly-(*R*,*R*,*R*,*R*)^{P}AAMP)$_2$O*t*Bu **Fmoc-4GR^{P}A** measured in $CDCl_3$ at 151 MHz.

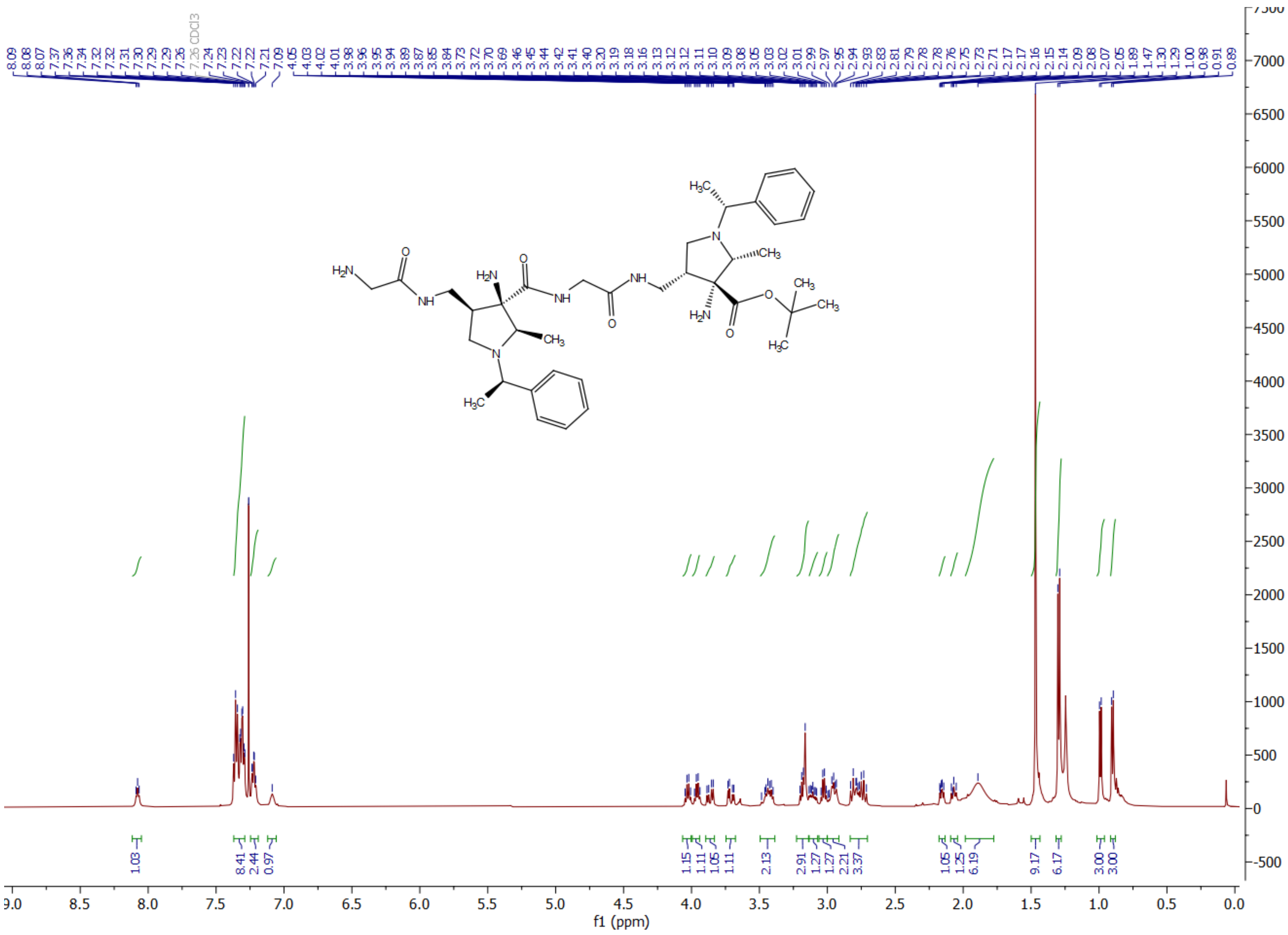

^{1}H-NMR spectrum of α/γ-peptide NH_2(Gly-(*R*,*R*,*R*,*R*)[P]AAMP)$_2$O*t*Bu **NH_2-4GR[P]A** measured in $CDCl_3$ at 500 MHz.

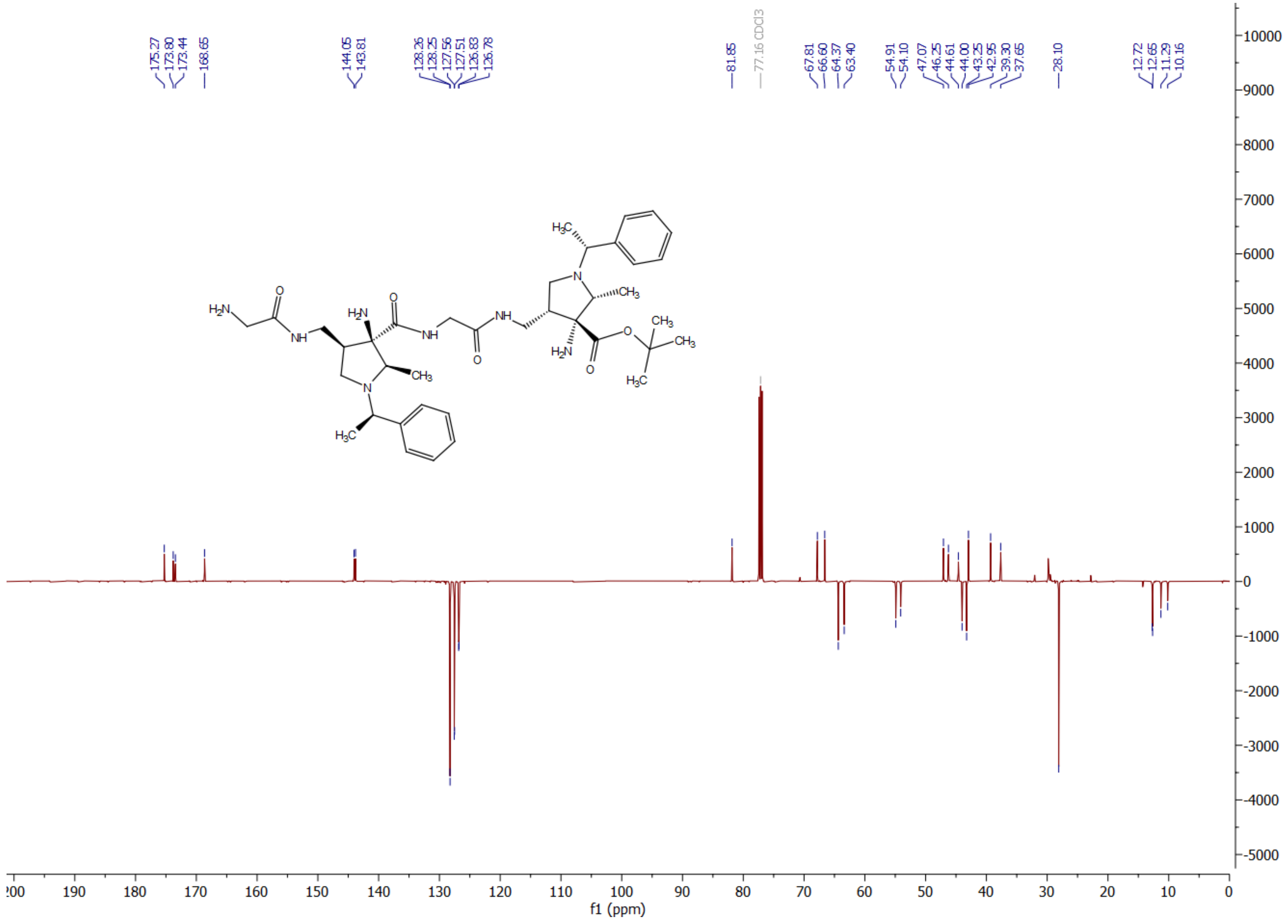

^{13}C-NMR spectrum of α/γ-peptide NH_2(Gly-(*R*,*R*,*R*,*R*)^{P}AAMP)$_2$O*t*Bu **NH_2-4GR^{P}A** measured in $CDCl_3$ at 126 MHz.

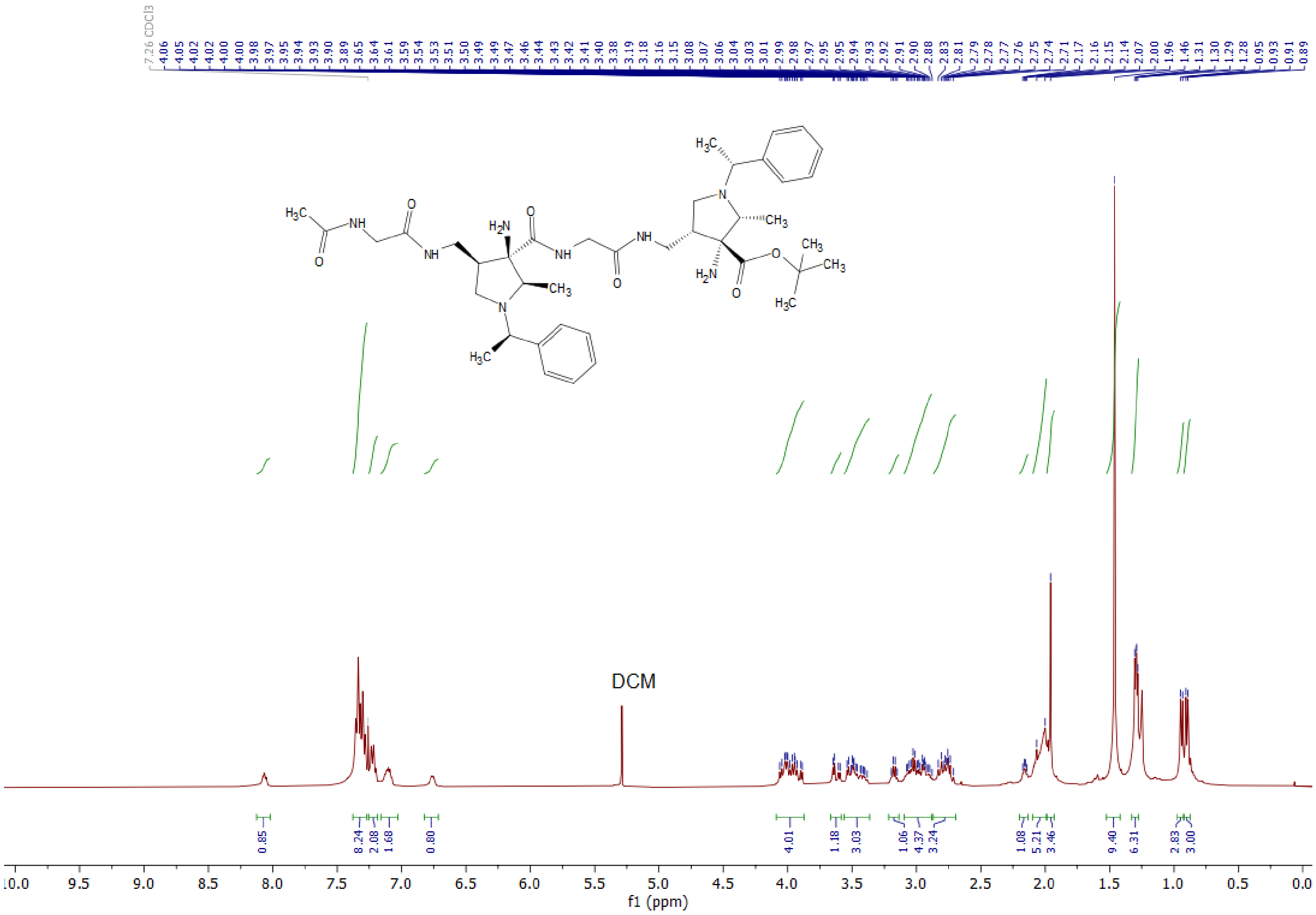

^{1}H-NMR spectrum of α/γ-peptide Ac(Gly-(*R*,*R*,*R*,*R*)[P]AAMP)$_2$O*t*Bu **Ac-4GR[P]A** measured in $CDCl_3$ at 401 MHz.

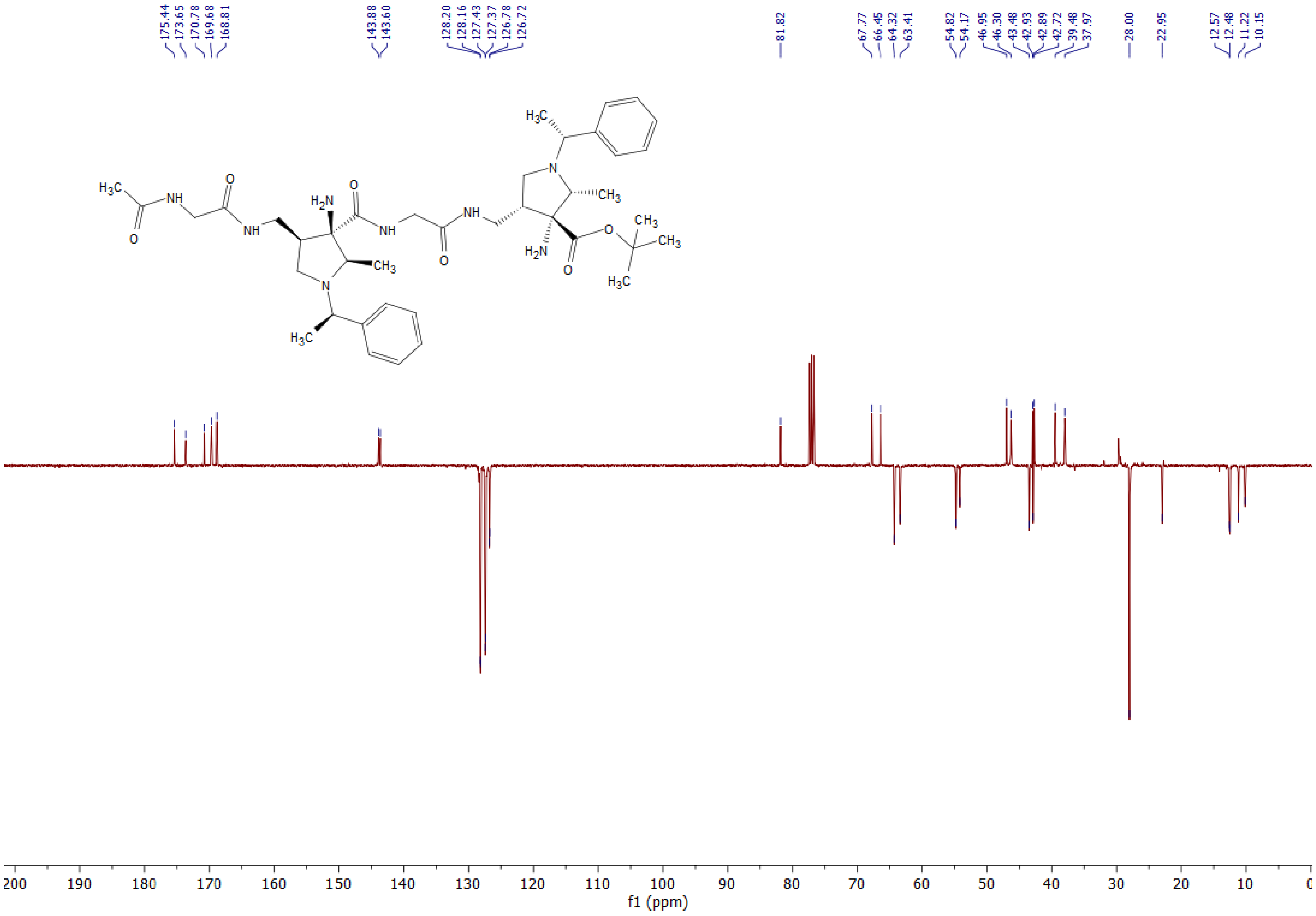

^{13}C-NMR spectrum of α/γ-peptide Ac(Gly-(*R*,*R*,*R*,*R*)^{P}AAMP)$_2$O*t*Bu **Ac-4GR^{P}A** measured in $CDCl_3$ at 101 MHz.

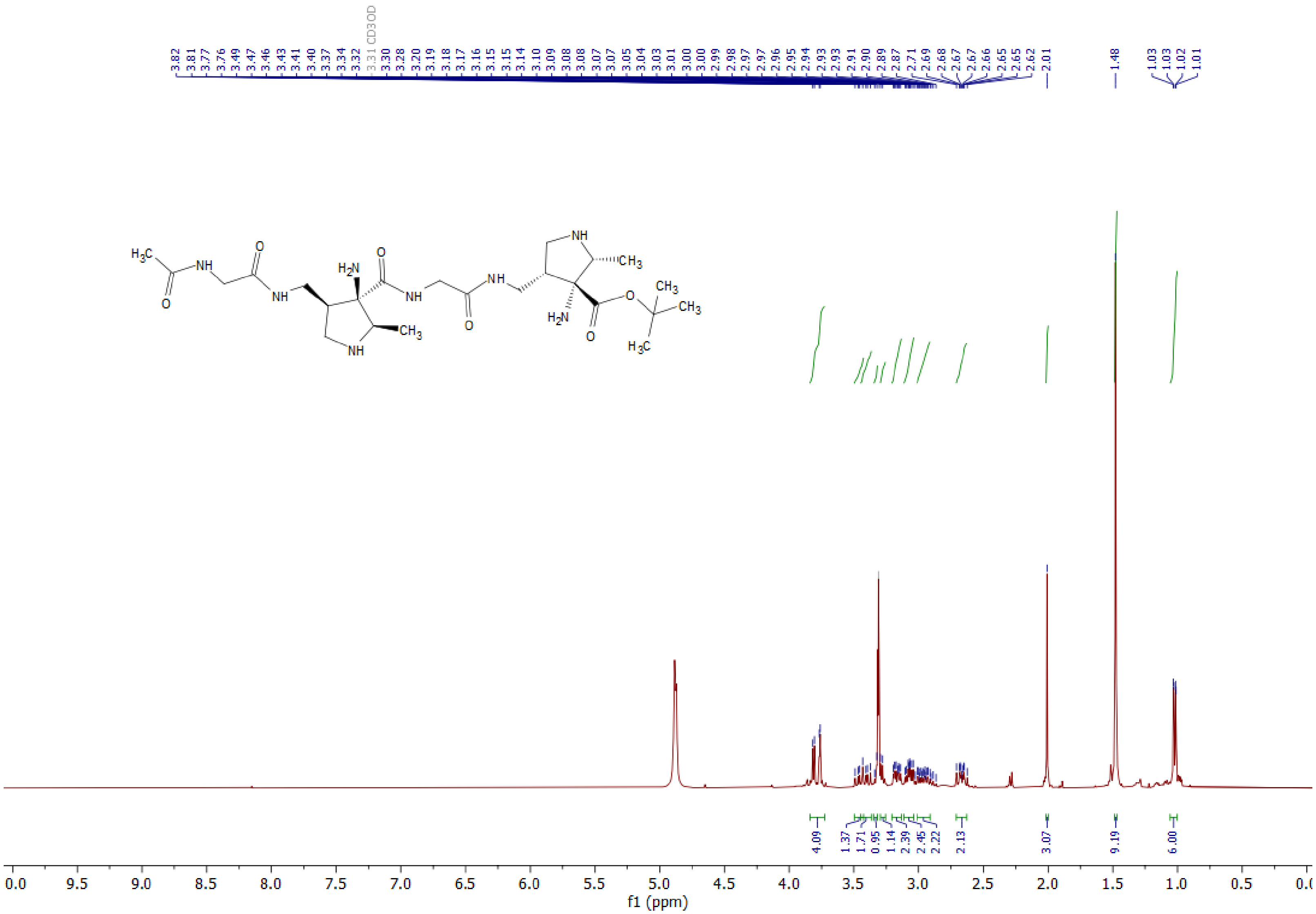

^{1}H-NMR spectrum of α/γ-peptide Ac(Gly-(*R*,*R*,*R*)AAMP)$_2$O*t*Bu **Ac-4GR^{H}A** measured in MeOD at 400 MHz.

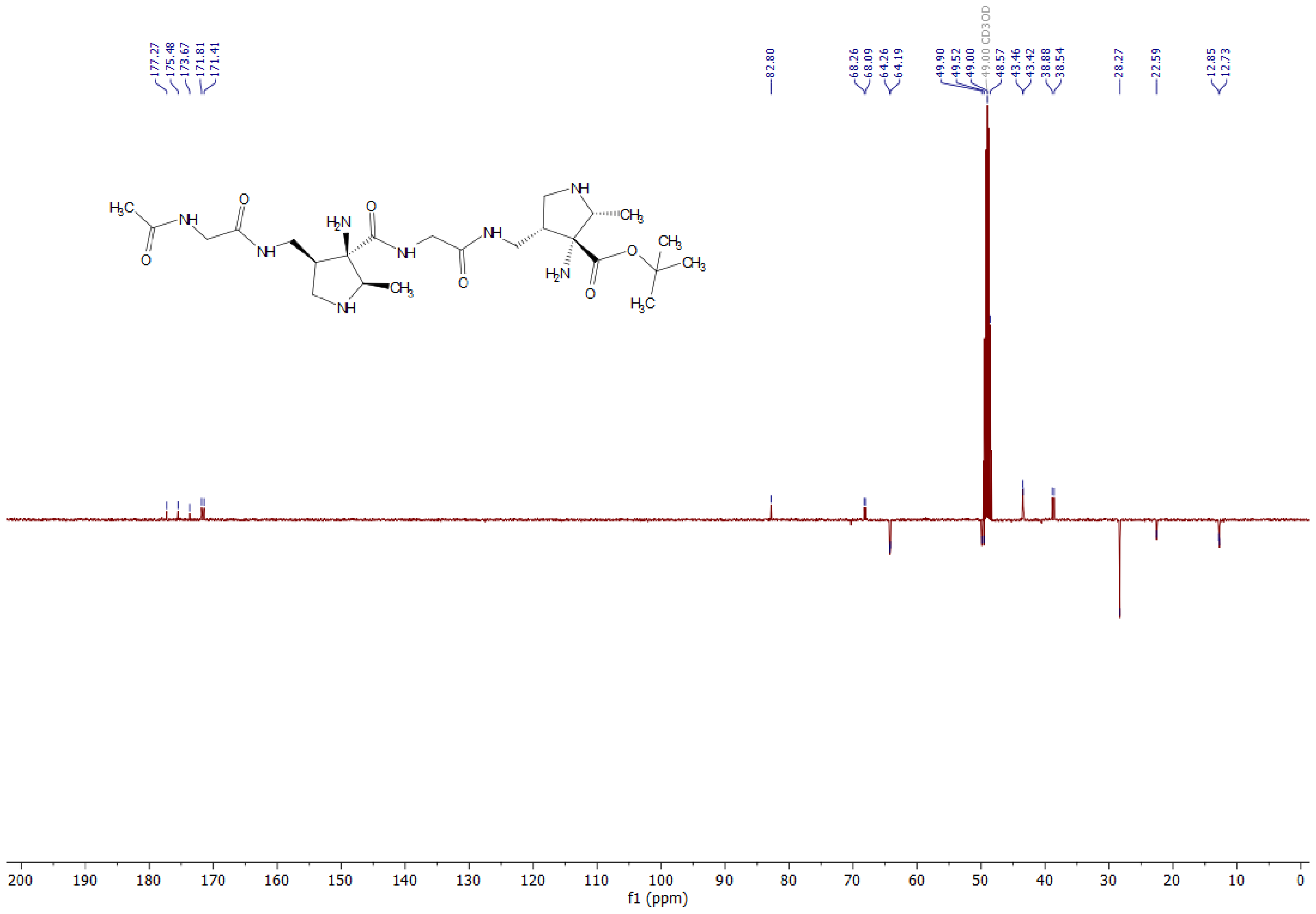

^{13}C-NMR spectrum of α/γ-peptide Ac(Gly-(*R*,*R*,*R*)AAMP)$_2$O*t*Bu **Ac-4GR^{H}A** measured in MeOD at 101 MHz.

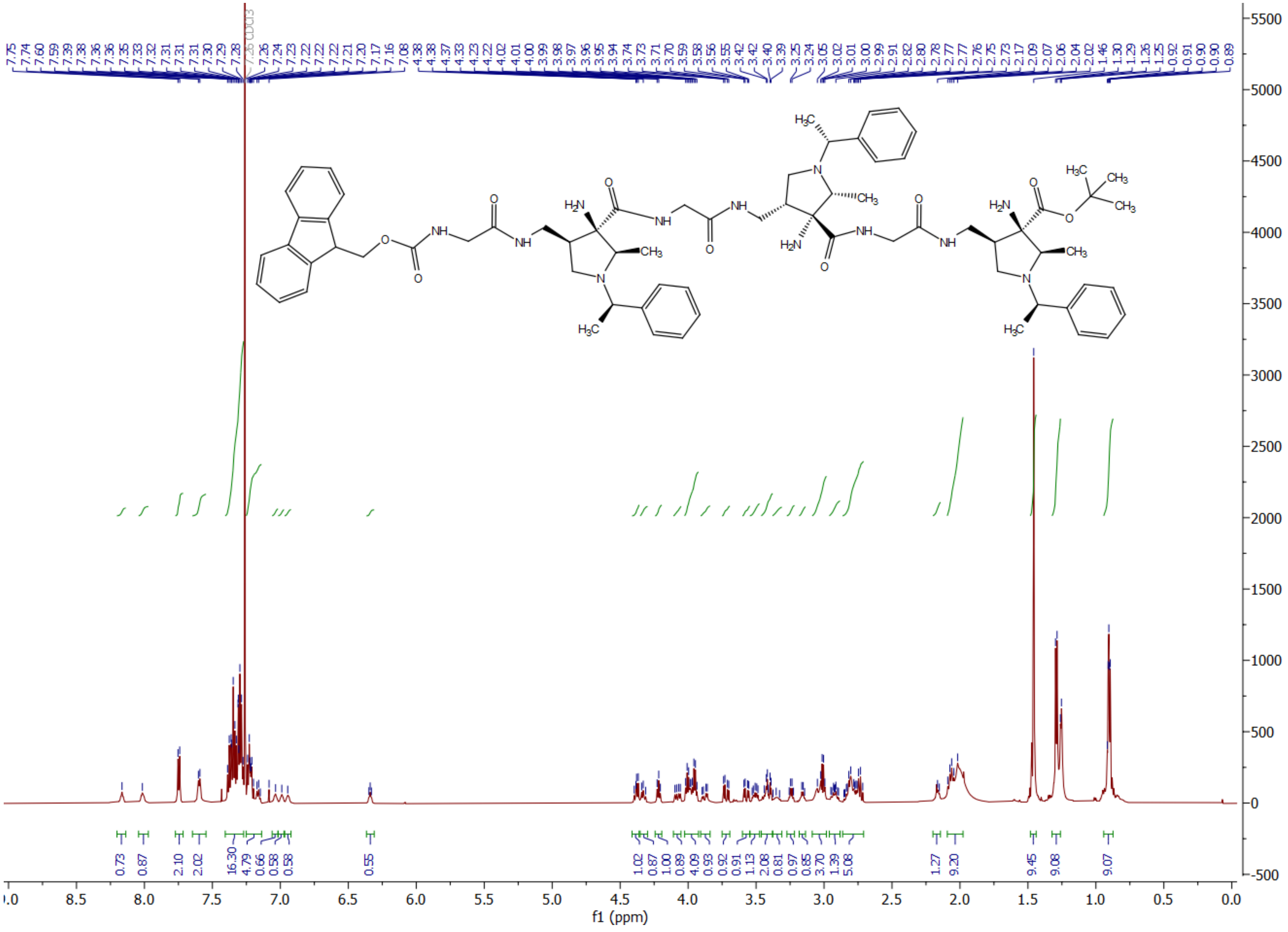

^{1}H-NMR spectrum of α/γ-peptide Fmoc(Gly-(*R*,*R*,*R*,*R*)[P]AAMP)$_3$O*t*Bu **Fmoc-6GR[P]A** measured in $CDCl_3$ at 600 MHz.

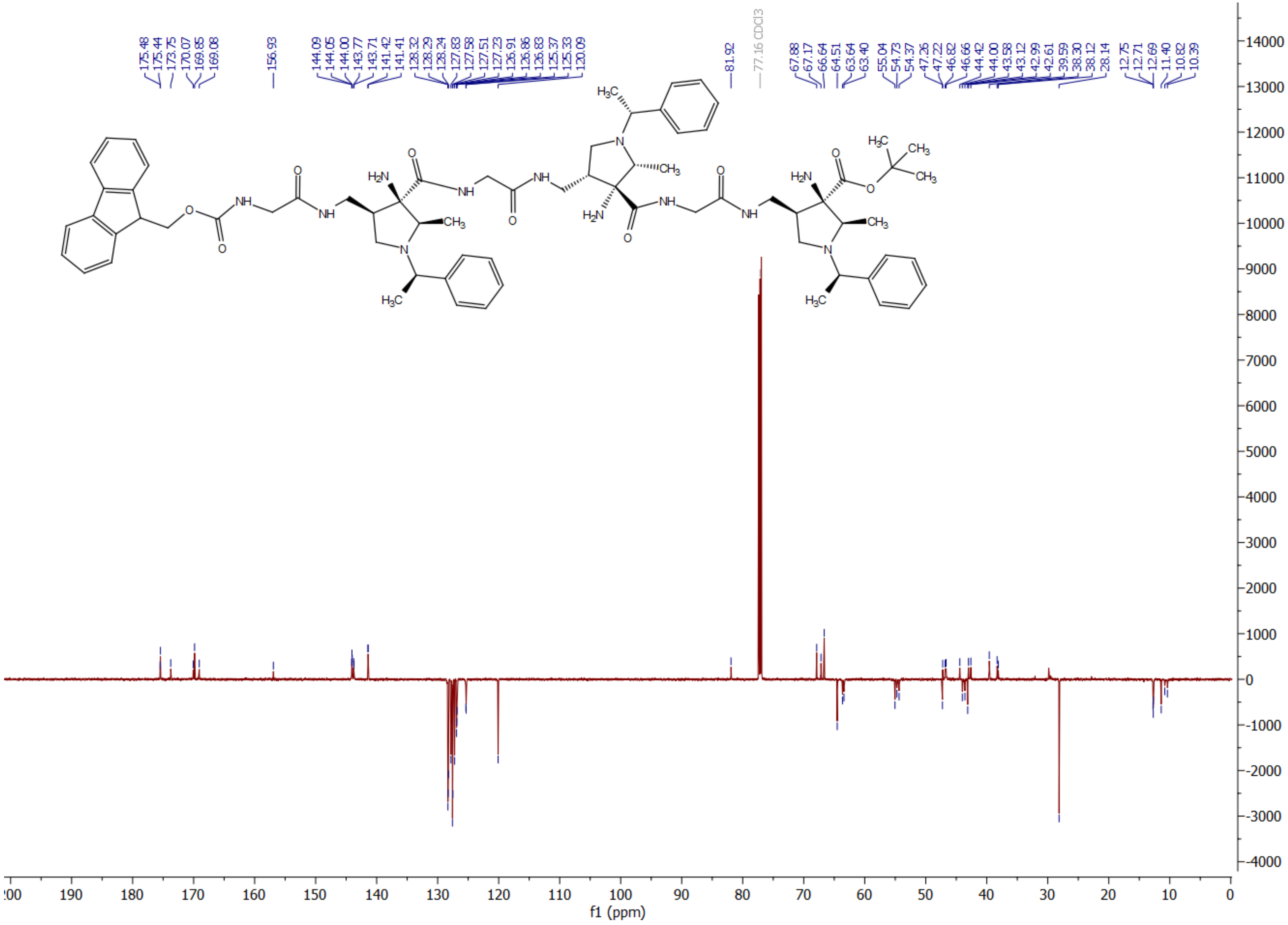

^{13}C-NMR spectrum of α/γ-peptide Fmoc(Gly-(*R*,*R*,*R*,*R*)^{P}AAMP)$_3$O*t*Bu **Fmoc-6GR^{P}A** measured in $CDCl_3$ at 151 MHz.

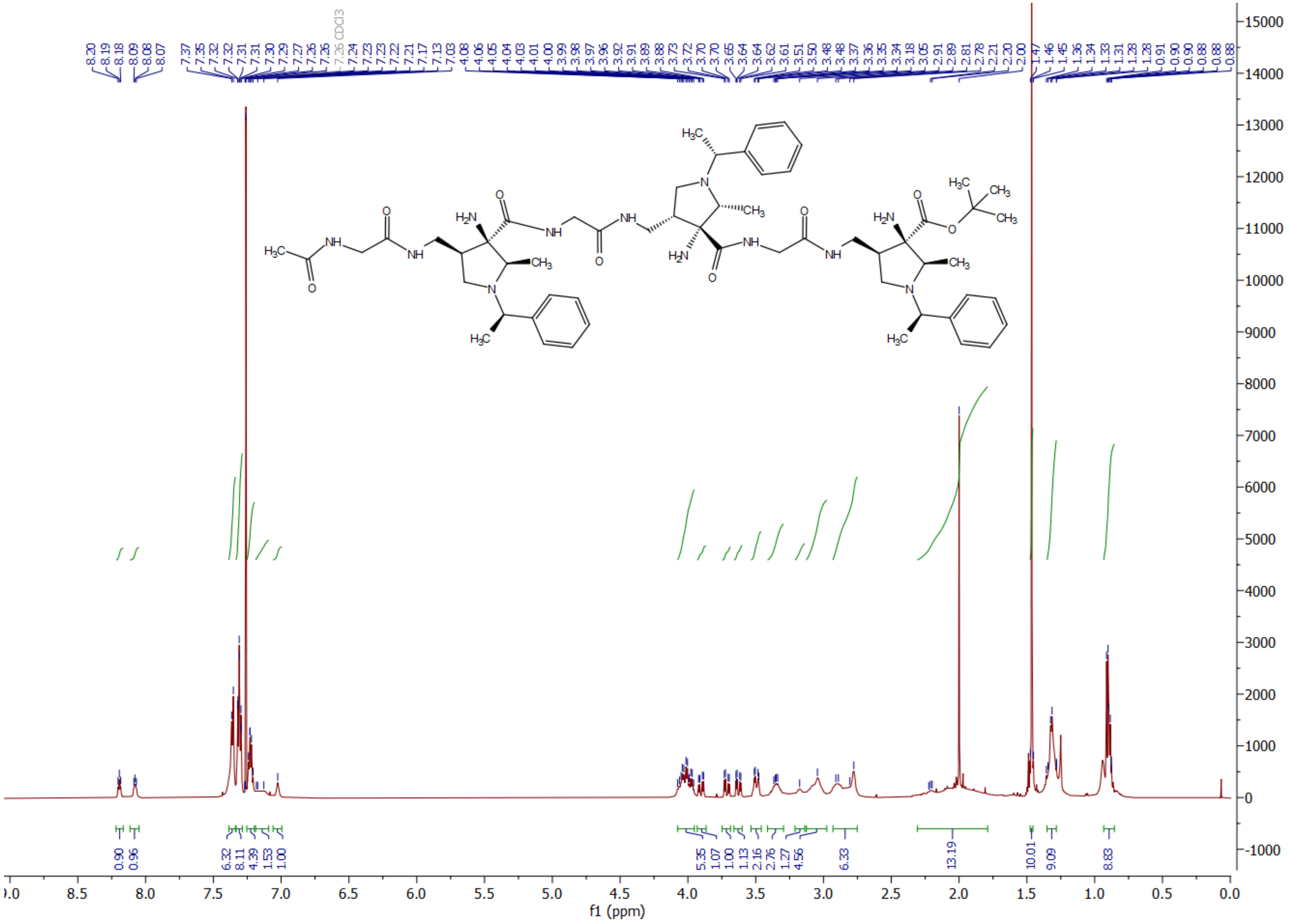

^{1}H-NMR spectrum of α/γ-peptide Ac(Gly-(*R*,*R*,*R*,*R*)[P]AAMP)$_3$O*t*Bu **Ac-6GR[P]A** measured in $CDCl_3$ at 600 MHz.

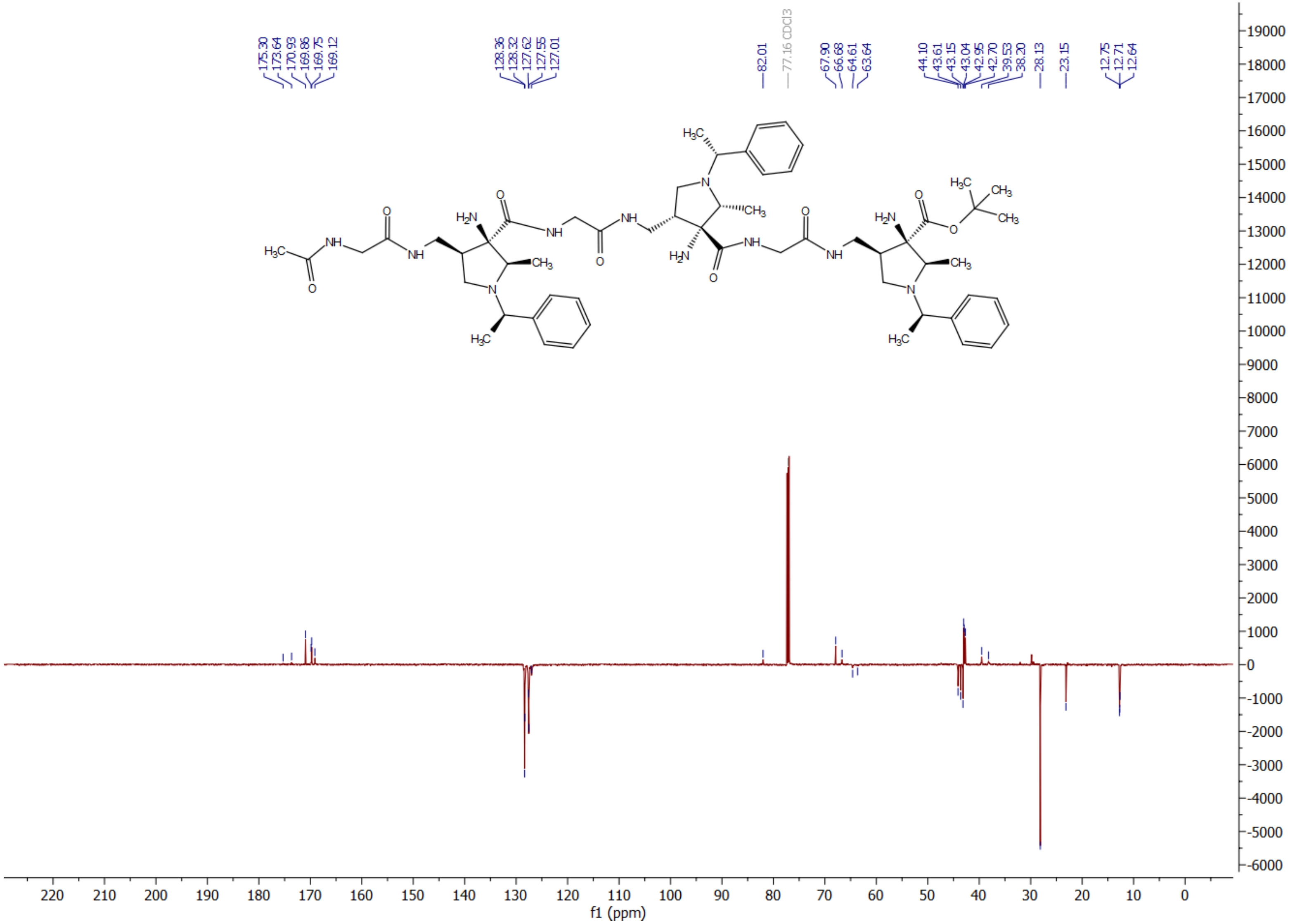

^{13}C-NMR spectrum of α/γ-peptide Ac(Gly-(*R*,*R*,*R*,*R*)^{P}AAMP)$_3$O*t*Bu **Ac-6GR^{P}A** measured in $CDCl_3$ at 151 MHz.

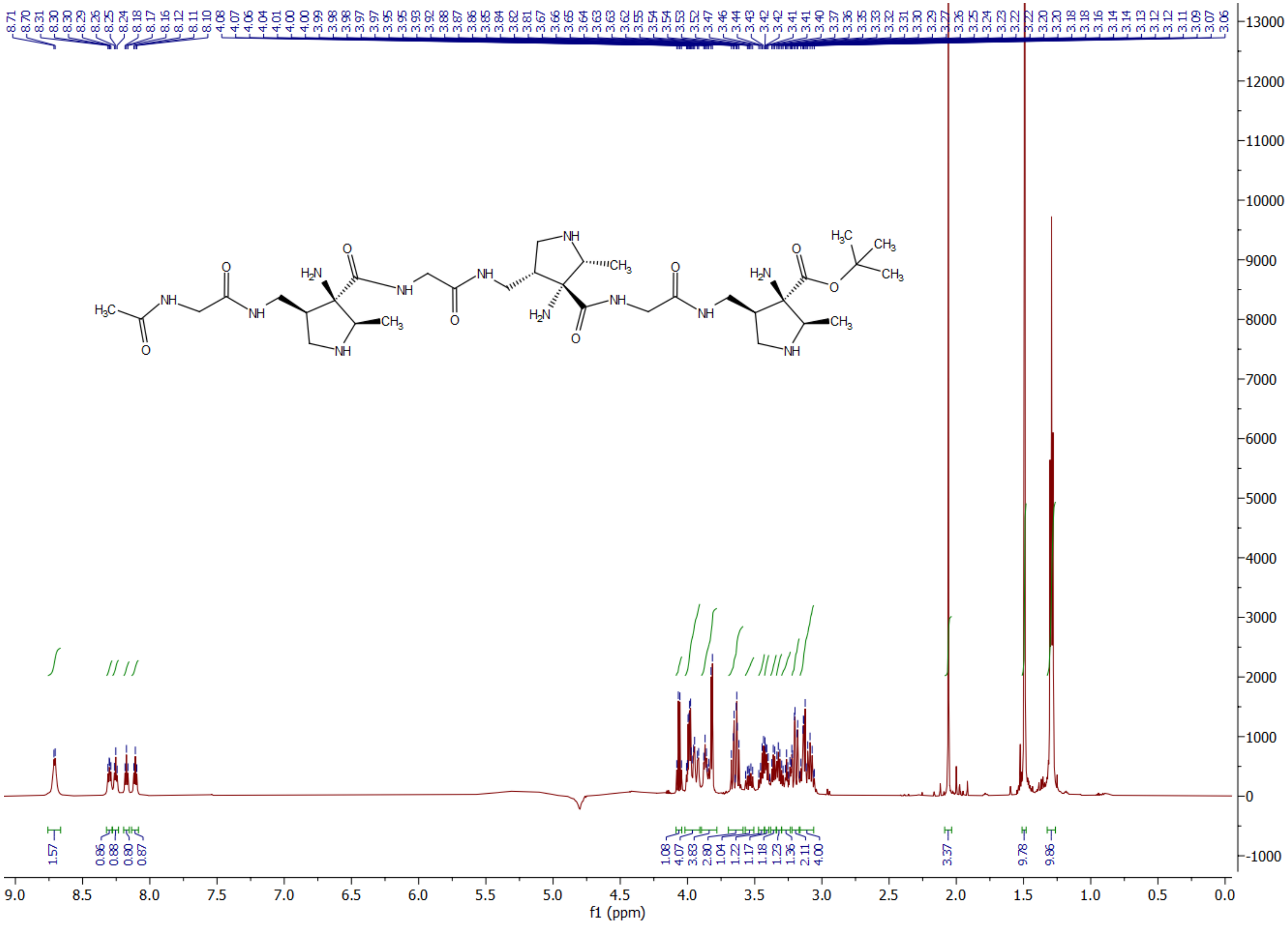

^{1}H-NMR spectrum of α/γ-peptide Ac(Gly-(*R*,*R*,*R*)AAMP)$_3$O*t*Bu **Ac-6GR$^{\mathbf{H}}$A** measured in H_2O:D_2O 9:1, acidified with CD_3COOD to pH of 4 at 600 MHz.

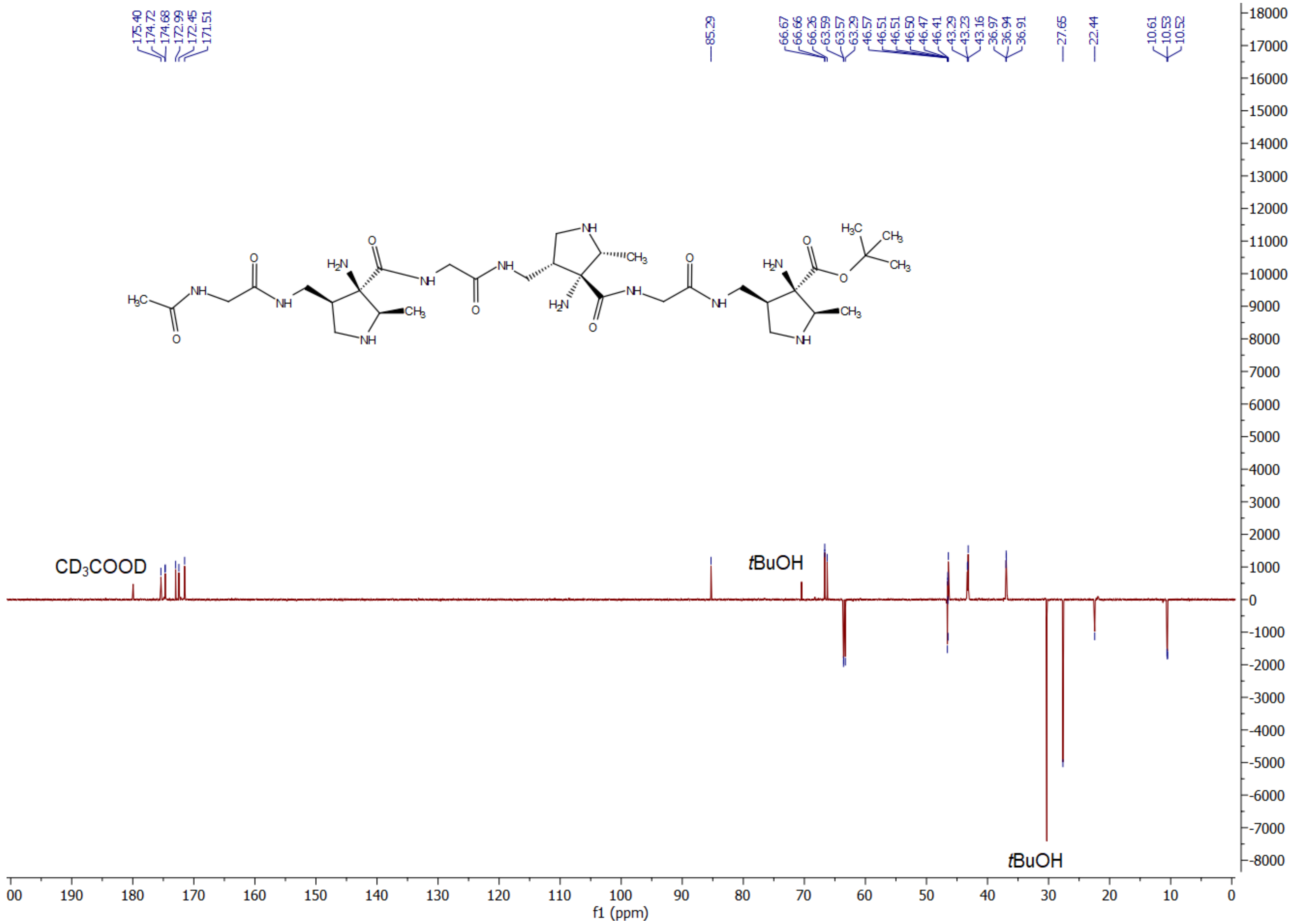

^{13}C-NMR spectrum of α/γ-peptide Ac(Gly-(*R*,*R*,*R*)AAMP)$_3$O*t*Bu **Ac-6GR^{H}A** measured in H_2O:D_2O 9:1, acidified with CD_3COOD to pH of 4 at 151 MHz.

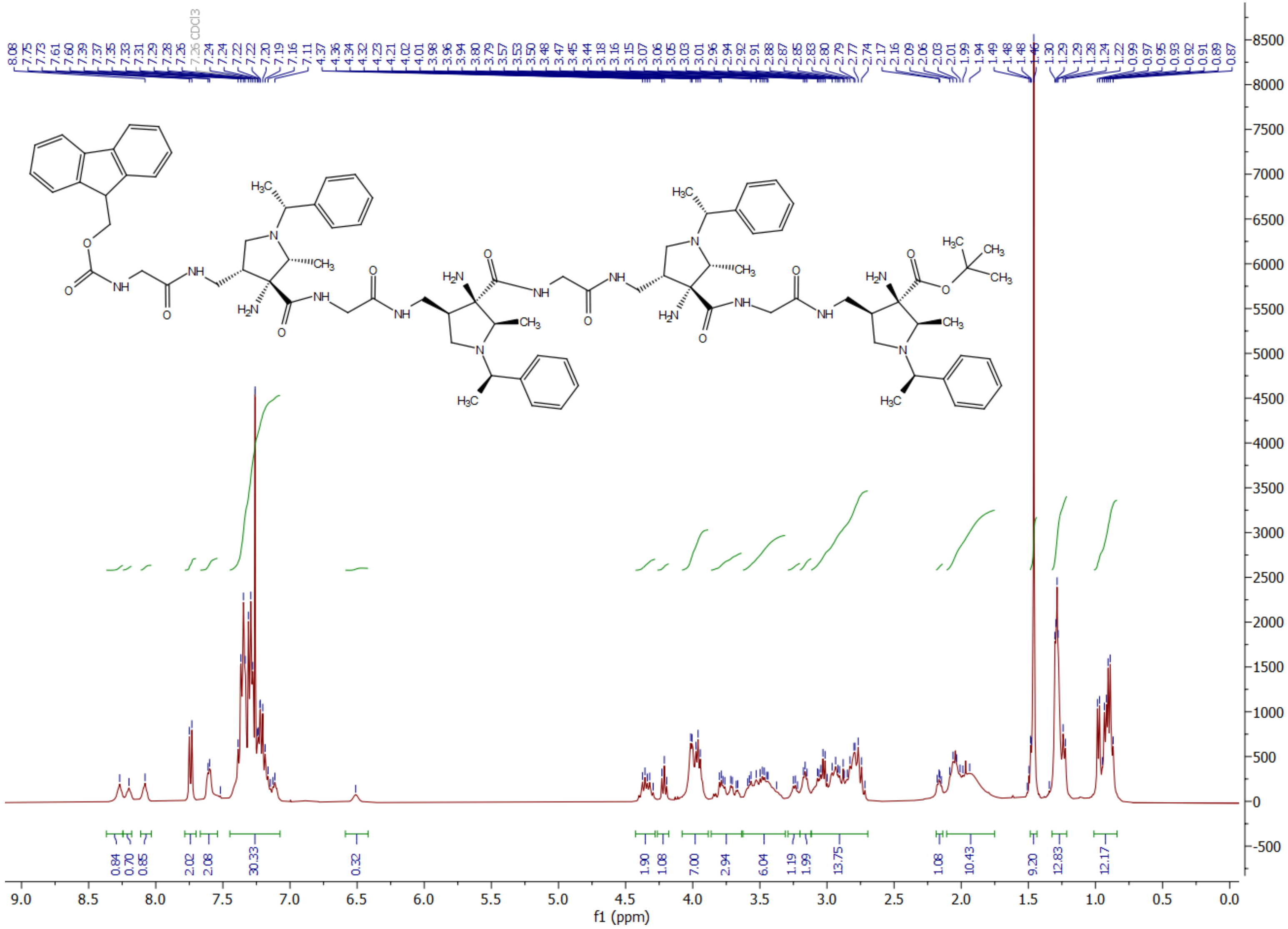

^{1}H-NMR spectrum of α/γ-peptide Fmoc(Gly-(*R*,*R*,*R*,*R*)^{P}AAMP)$_4$O*t*Bu **Fmoc-8GR^{P}A** measured in $CDCl_3$ at 401 MHz.

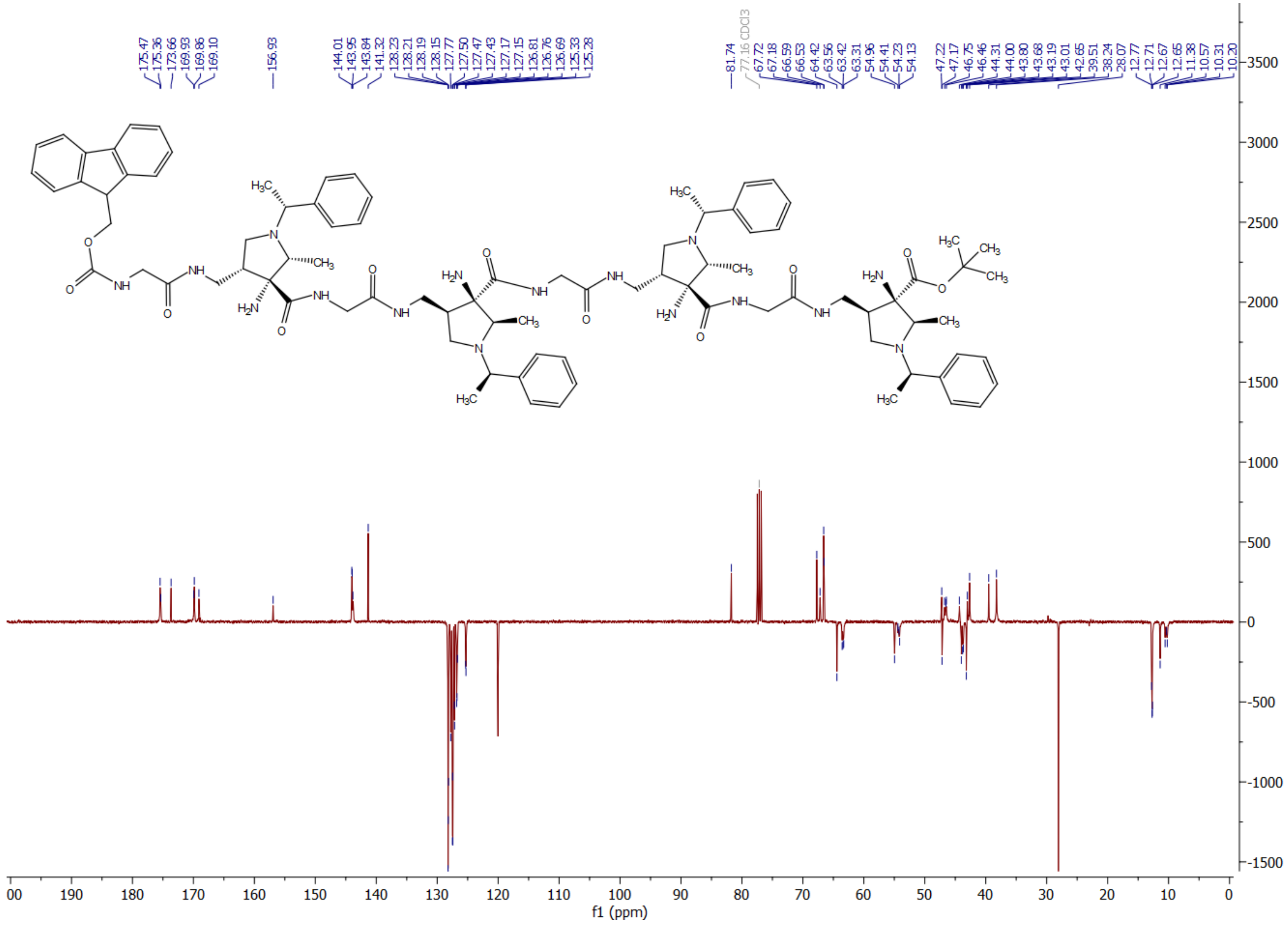

^{13}C-NMR spectrum of α/γ-peptide Fmoc(Gly-(*R*,*R*,*R*,*R*)^{P}AAMP)$_4$O*t*Bu **Fmoc-8GR^{P}A** measured in $CDCl_3$ at 101 MHz.

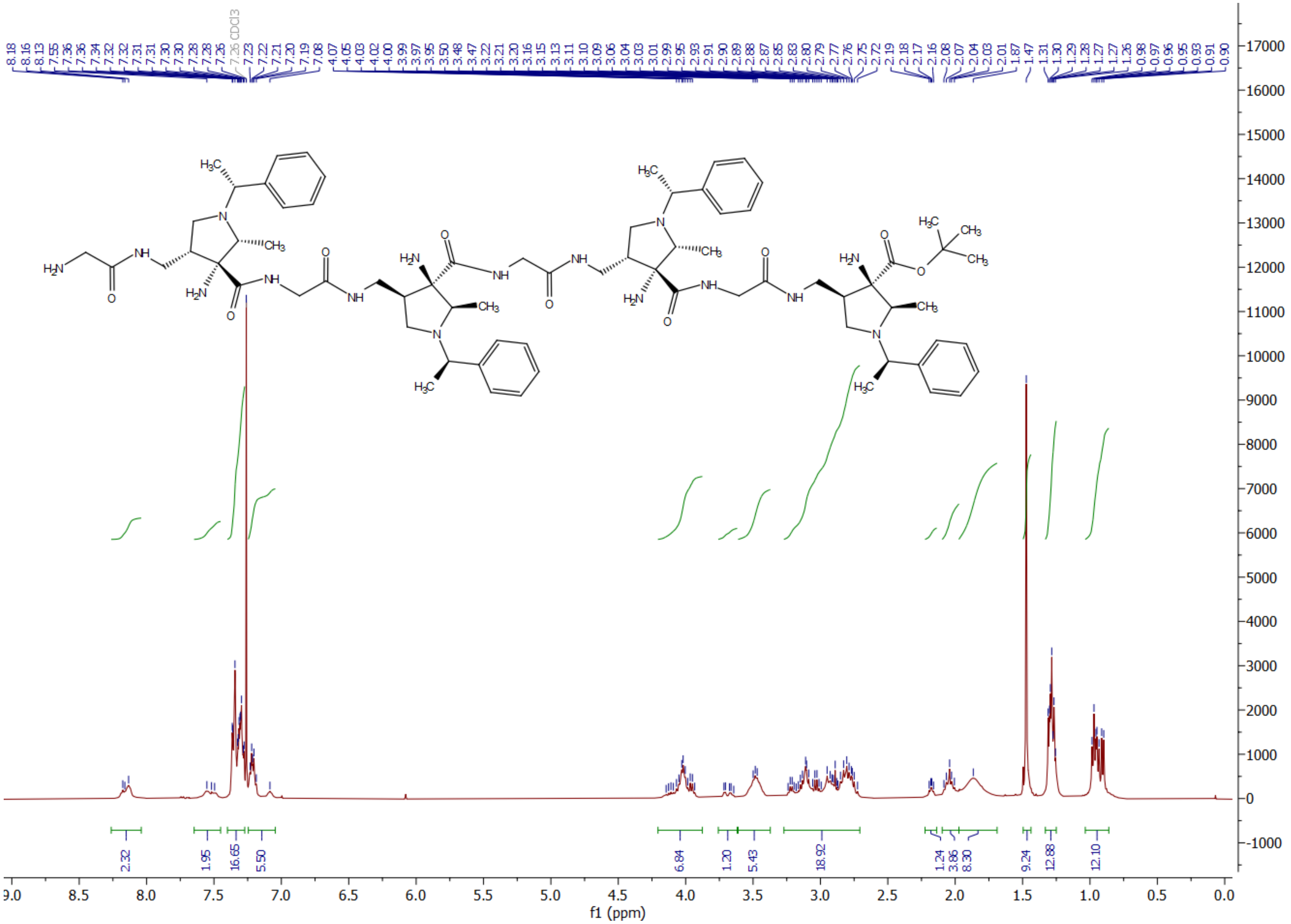

^{1}H-NMR spectrum of α/γ-peptide NH_2(Gly-(*R*,*R*,*R*,*R*)[P]AAMP)$_4$O*t*Bu **NH_2-8GR[P]A** measured in $CDCl_3$ at 401 MHz.

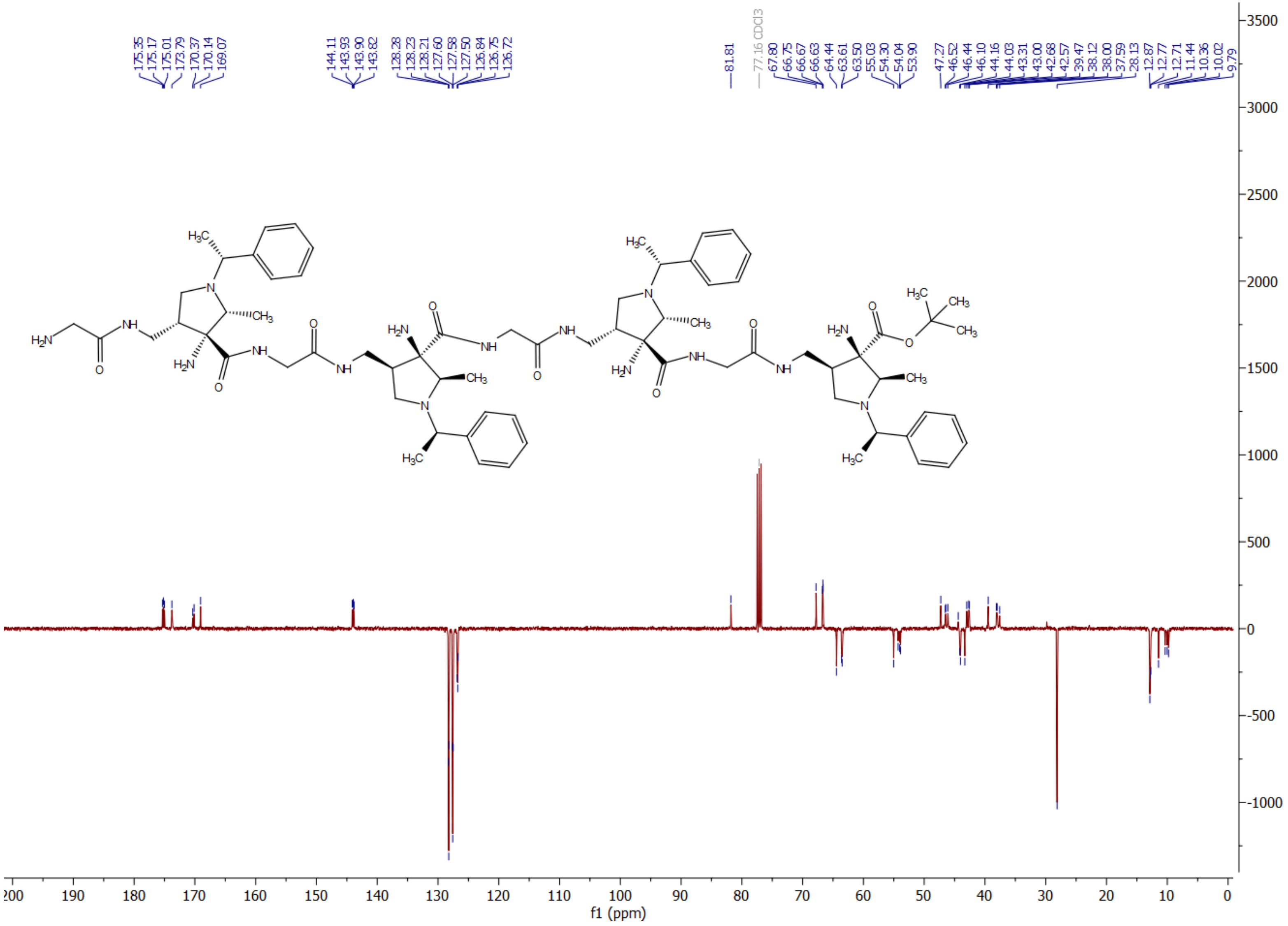

^{13}C-NMR spectrum of α/γ-peptide NH_2(Gly-(*R*,*R*,*R*,*R*)^{P}AAMP)$_4$O*t*Bu **NH_2-8GR^{P}A** measured in $CDCl_3$ at 101 MHz.

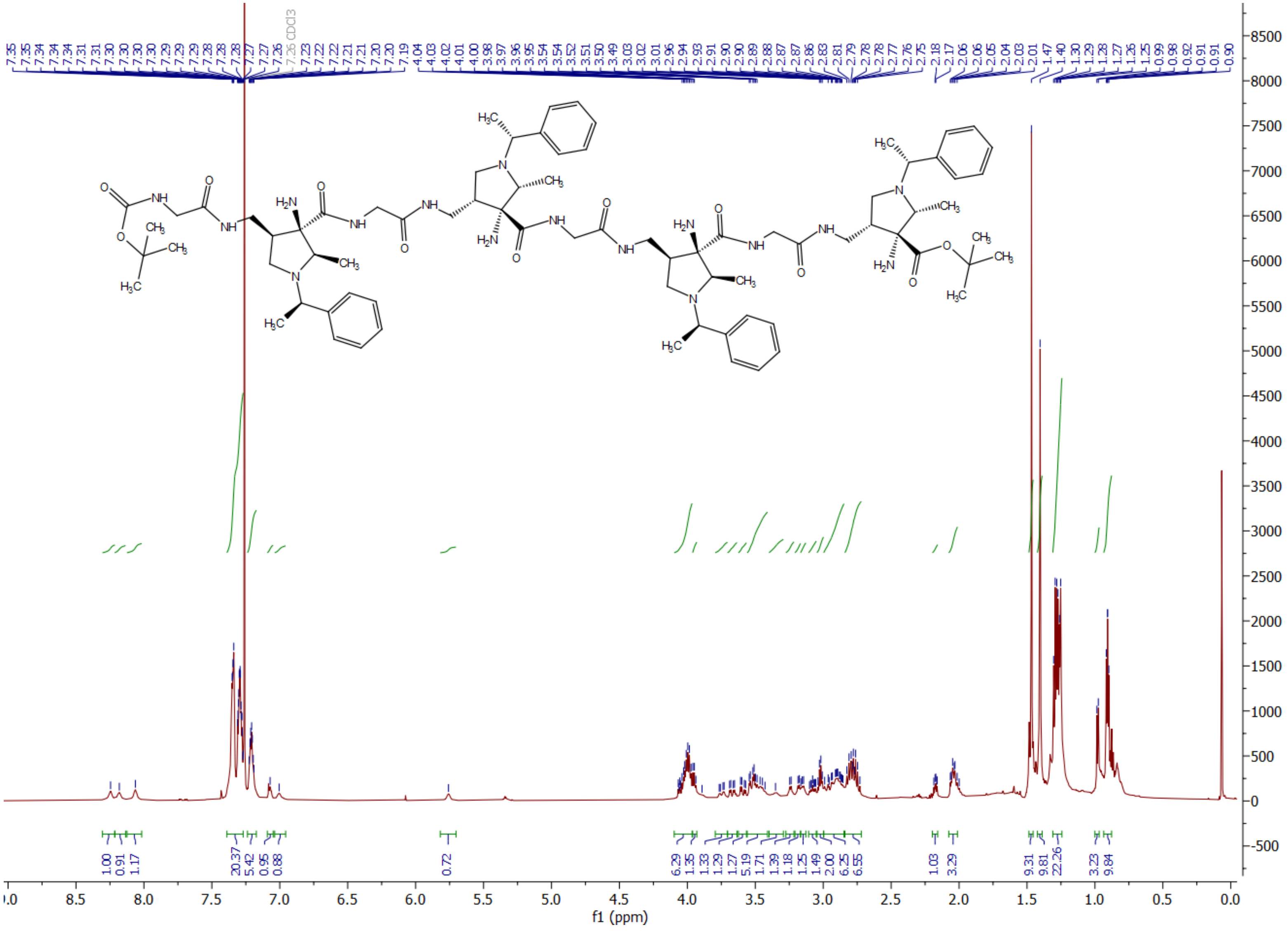

^{1}H-NMR spectrum of α/γ-peptide Boc(Gly-(*R*,*R*,*R*,*R*)^{P}AAMP)$_4$O*t*Bu **Boc-8GR^{P}A** measured in $CDCl_3$ at 600 MHz.

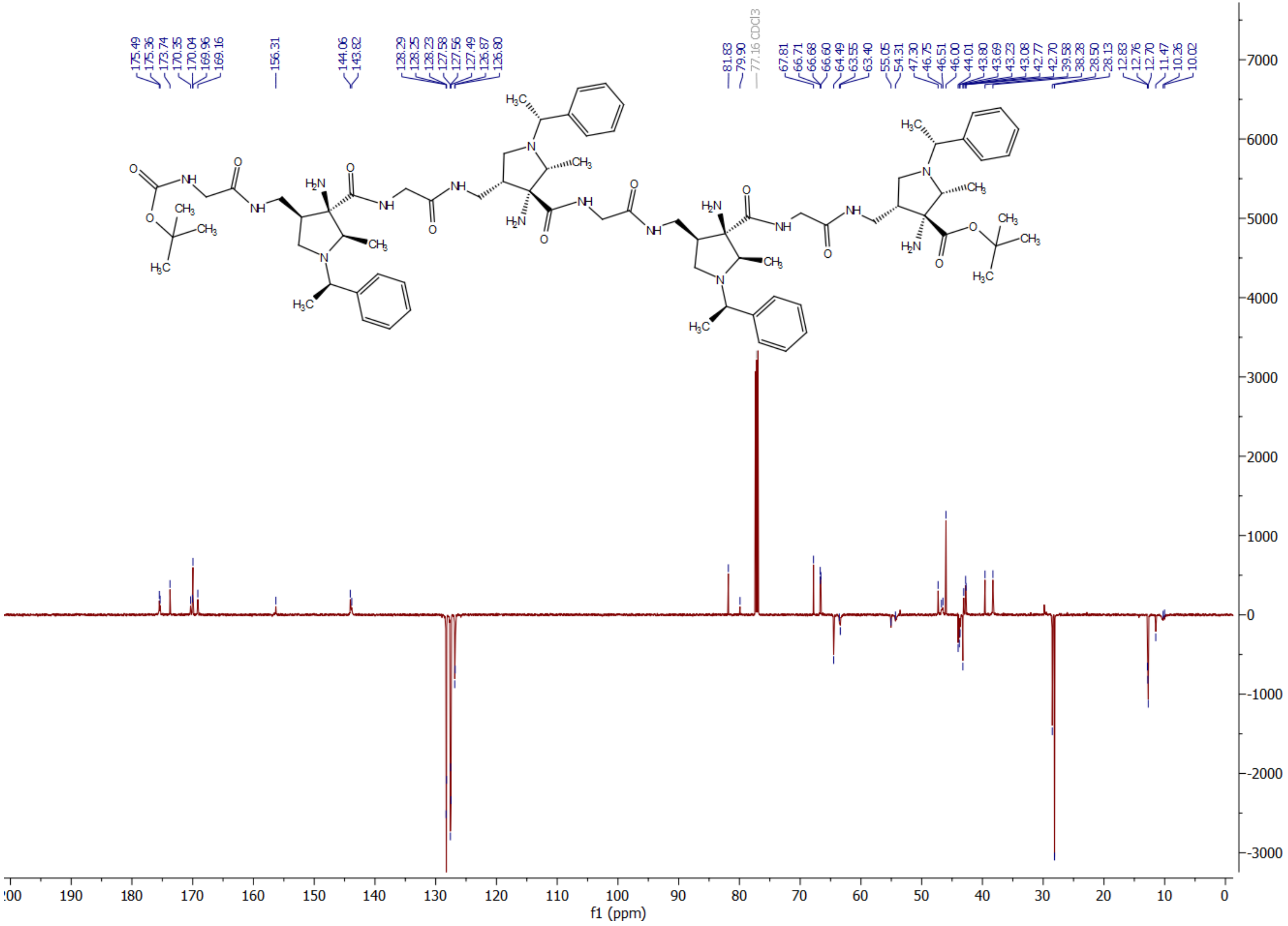

^{13}C-NMR spectrum of α/γ-peptide Boc(Gly-(*R*,*R*,*R*,*R*)^{P}AAMP)$_4$O*t*Bu **Boc-8GR^{P}A** measured in $CDCl_3$ at 151 MHz.

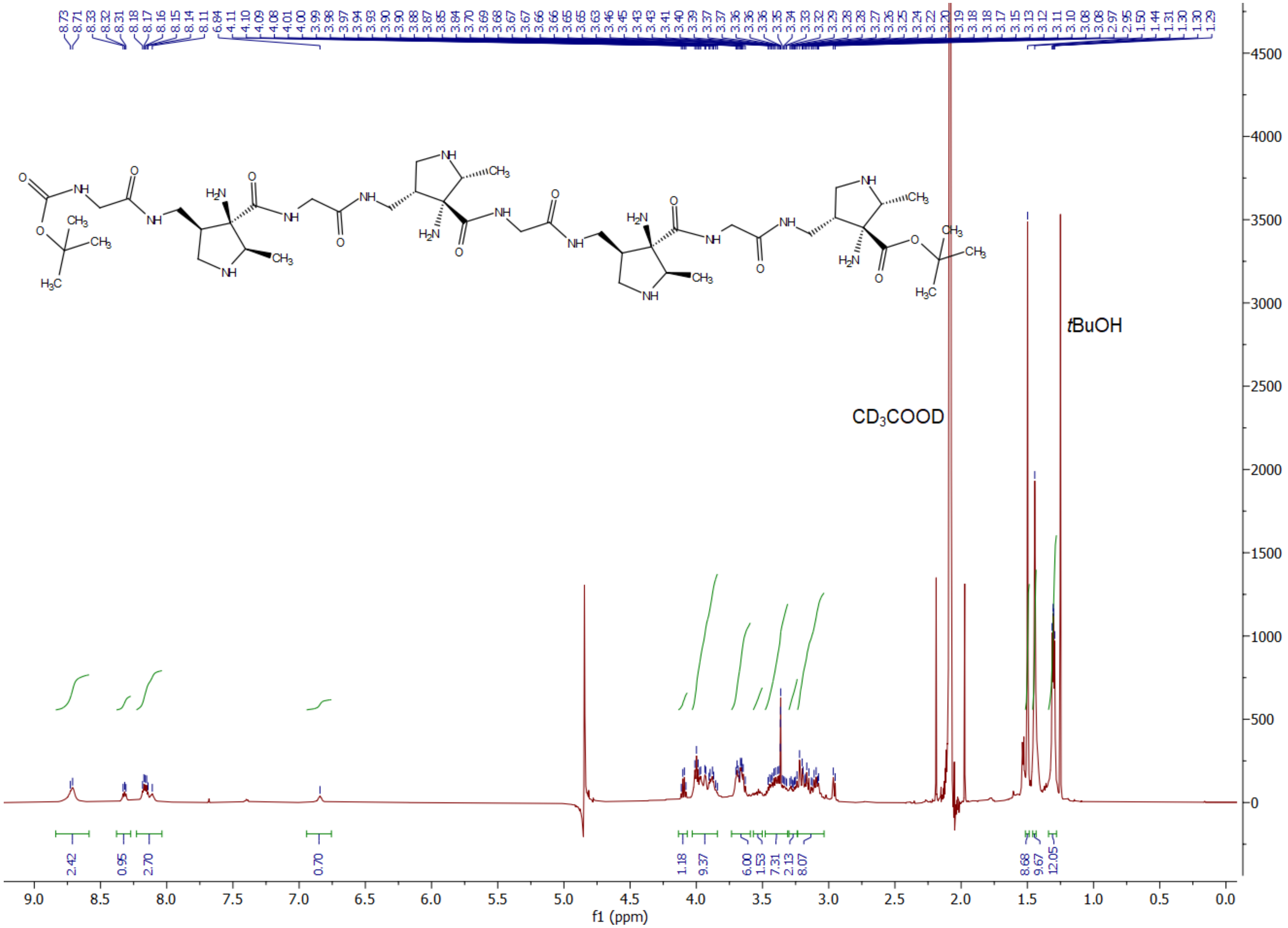

^{1}H-NMR spectrum of α/γ-peptide Boc(Gly-(*R*,*R*,*R*)AAMP)$_4$O*t*Bu **Boc-8GR^{H}A** measured in H_2O:D_2O 9:1, acidified with CD_3COOD to pH of 4 at 600 MHz.

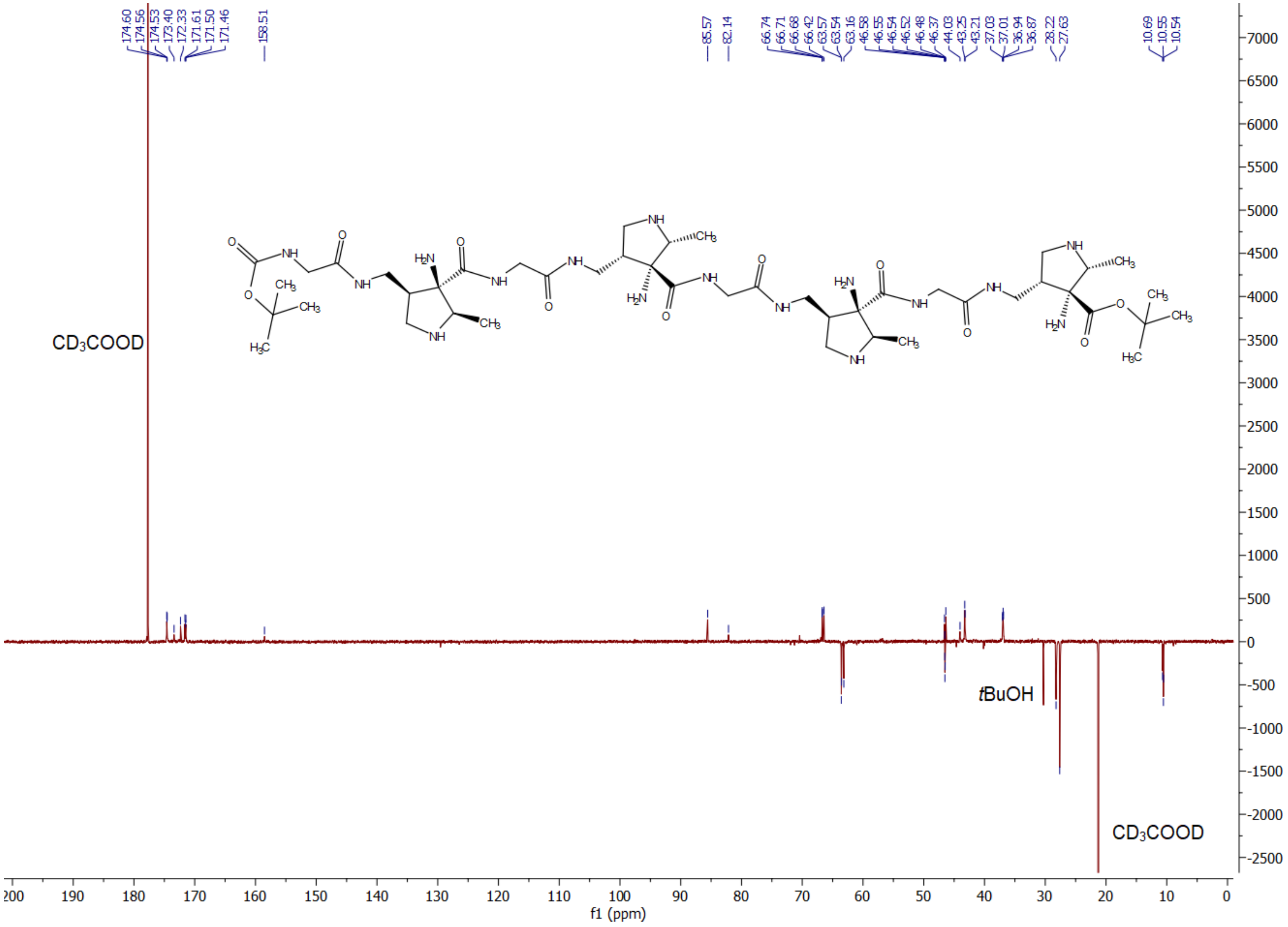

^{13}C-NMR spectrum of α/γ-peptide Boc(Gly-(*R*,*R*,*R*)AAMP)$_4$O*t*Bu **Boc-8GR^{H}A** measured in H_2O:D_2O 9:1, acidified with CD_3COOD to pH of 4 at 151 MHz.

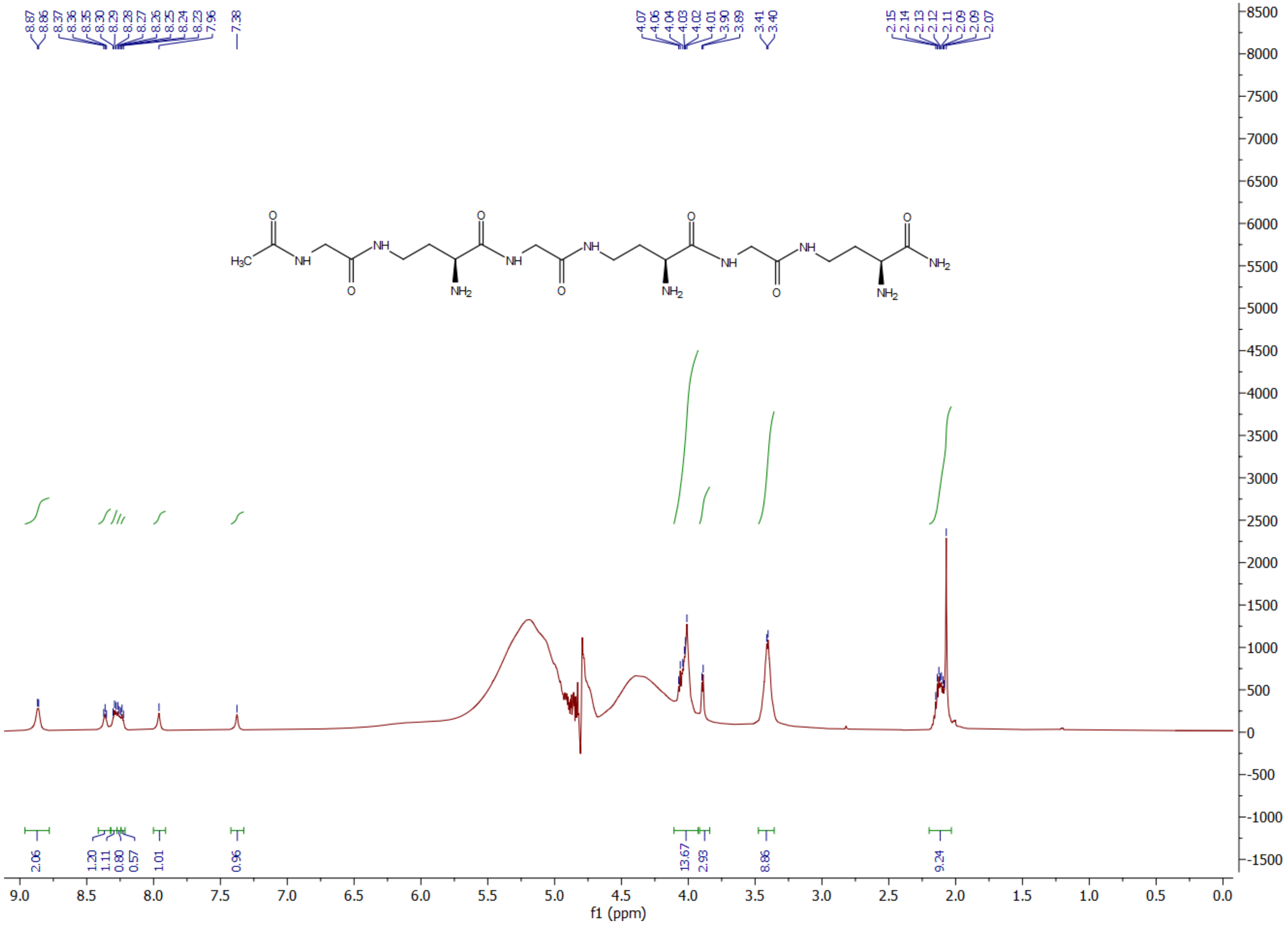

^{1}H-NMR spectrum of α/γ-peptide Ac(Gly-Dab)$_3$NH$_2$ **Ac-6GSDab** measured in H_2O:D_2O 9:1, acidified with CD_3COOD to pH of 4 at 600 MHz.

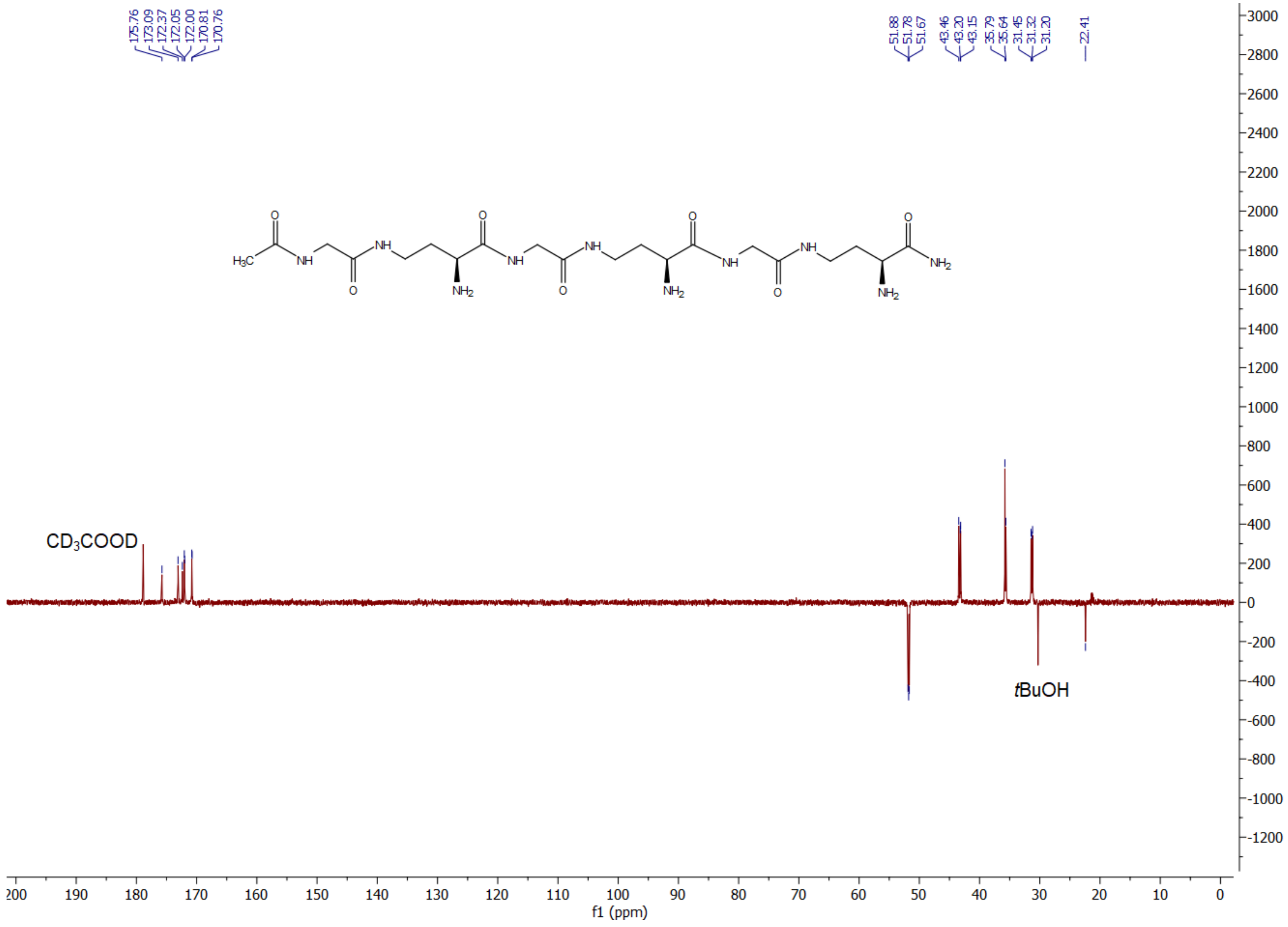

^{13}C-NMR spectrum of α/γ-peptide Ac(Gly-Dab)$_3$NH$_2$ **Ac-6GSDab** measured in H_2O:D_2O 9:1, acidified with CD_3COOD to pH of 4 at 151 MHz.

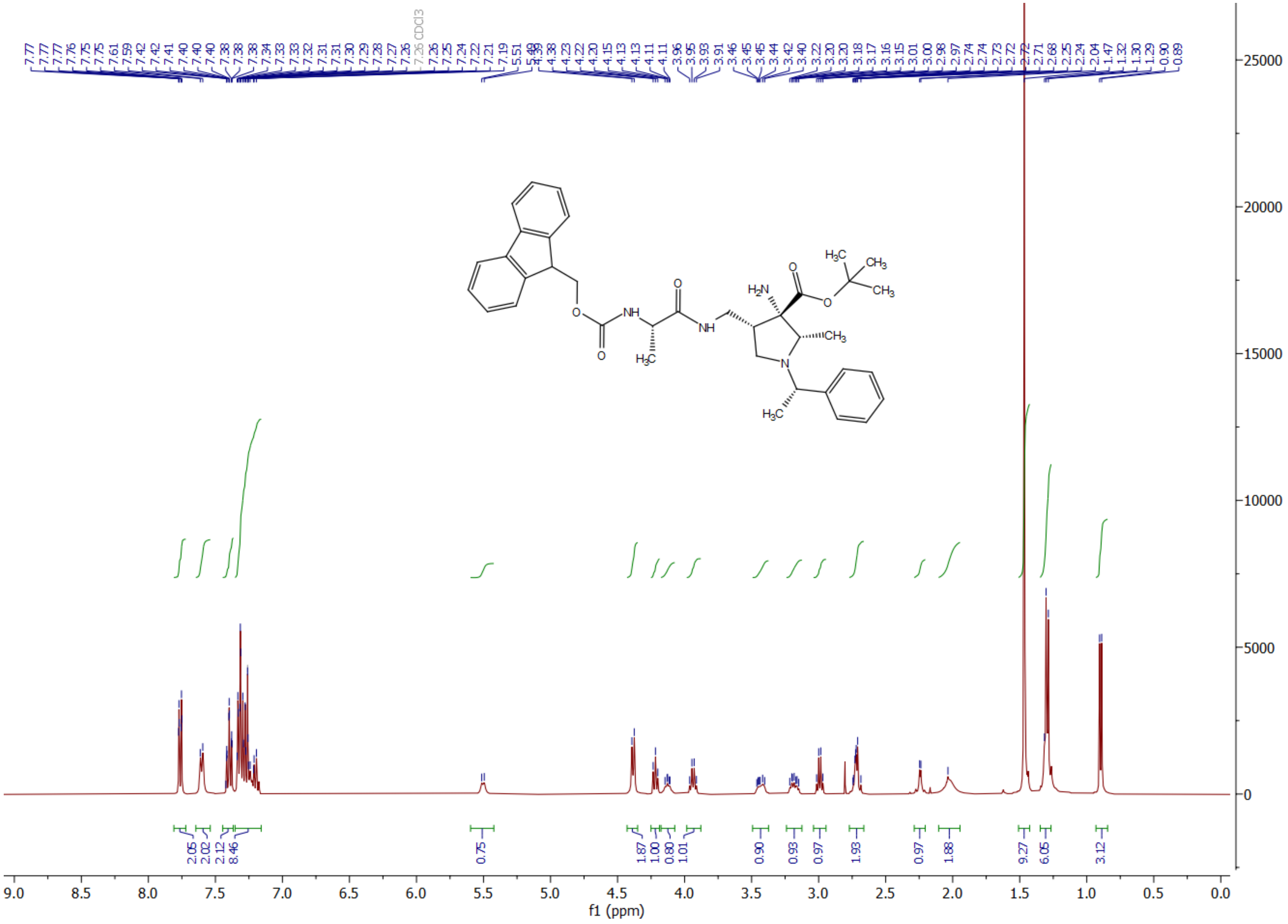

^{1}H-NMR spectrum of α/γ-peptide Fmoc(Ala-(*S*,*S*,*S*,*S*)[P]AAMP)O*t*Bu **Fmoc-2AS[P]A** measured in $CDCl_3$ at 401 MHz.

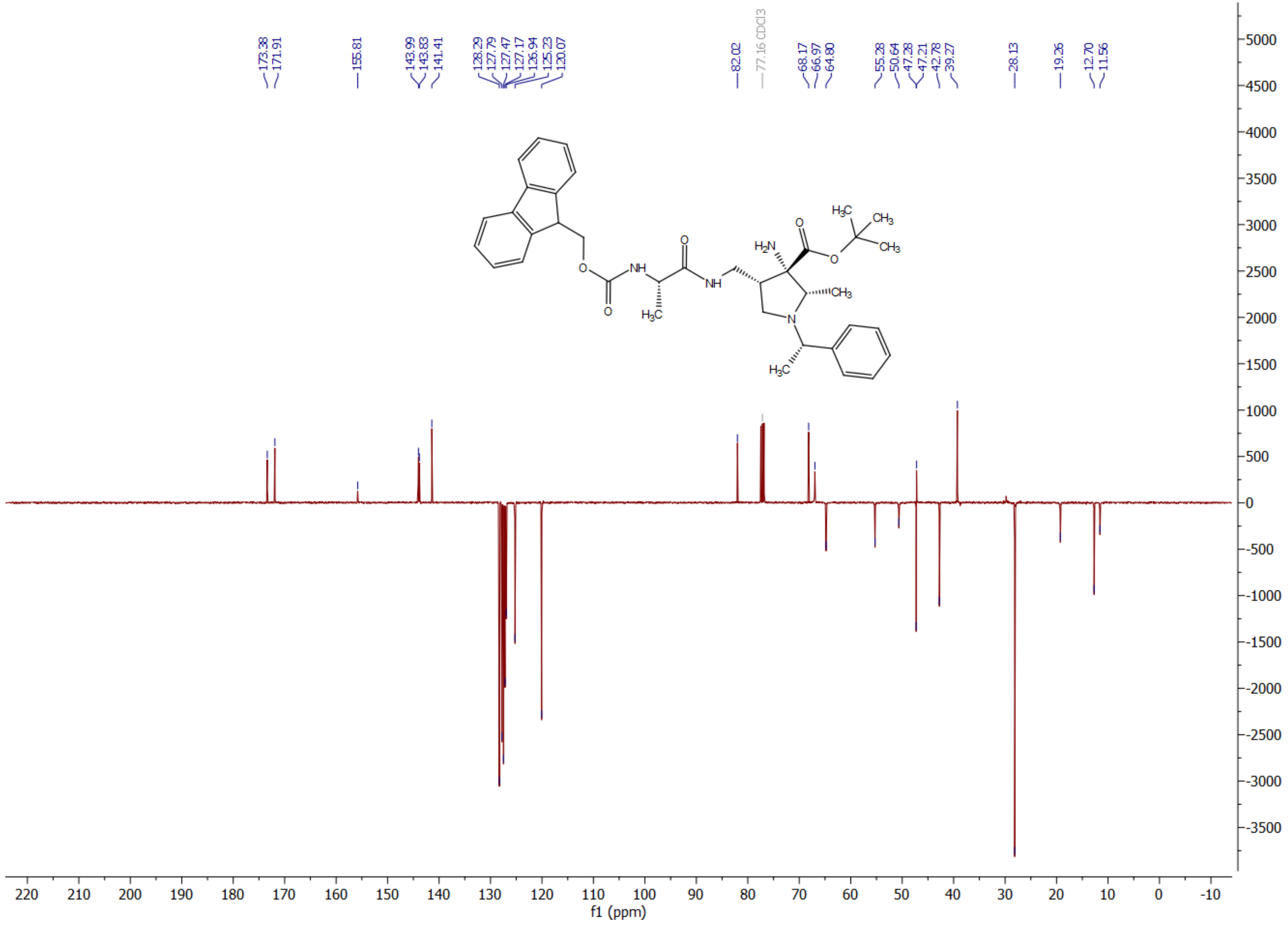

^{13}C-NMR spectrum of α/γ-peptide Fmoc(Ala-(*S*,*S*,*S*,*S*)^{P}AAMP)O*t*Bu **Fmoc-2AS^{P}A** measured in $CDCl_3$ at 101 MHz.

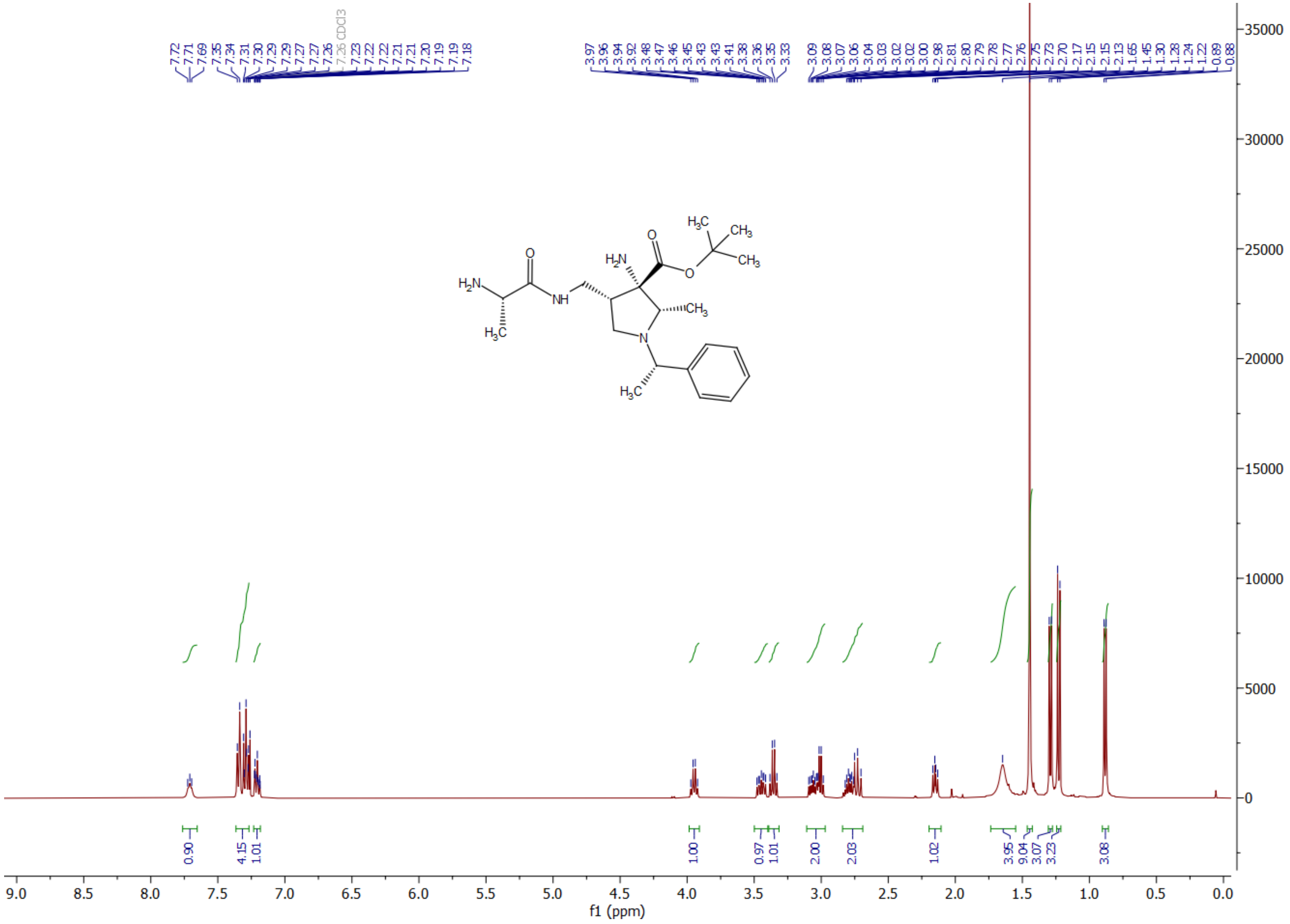

^{1}H-NMR spectrum of α/γ-peptide NH_2(Ala-(*S*,*S*,*S*,*S*)[P]AAMP)O*t*Bu **NH_2-2AS[P]A** measured in $CDCl_3$ at 401 MHz.

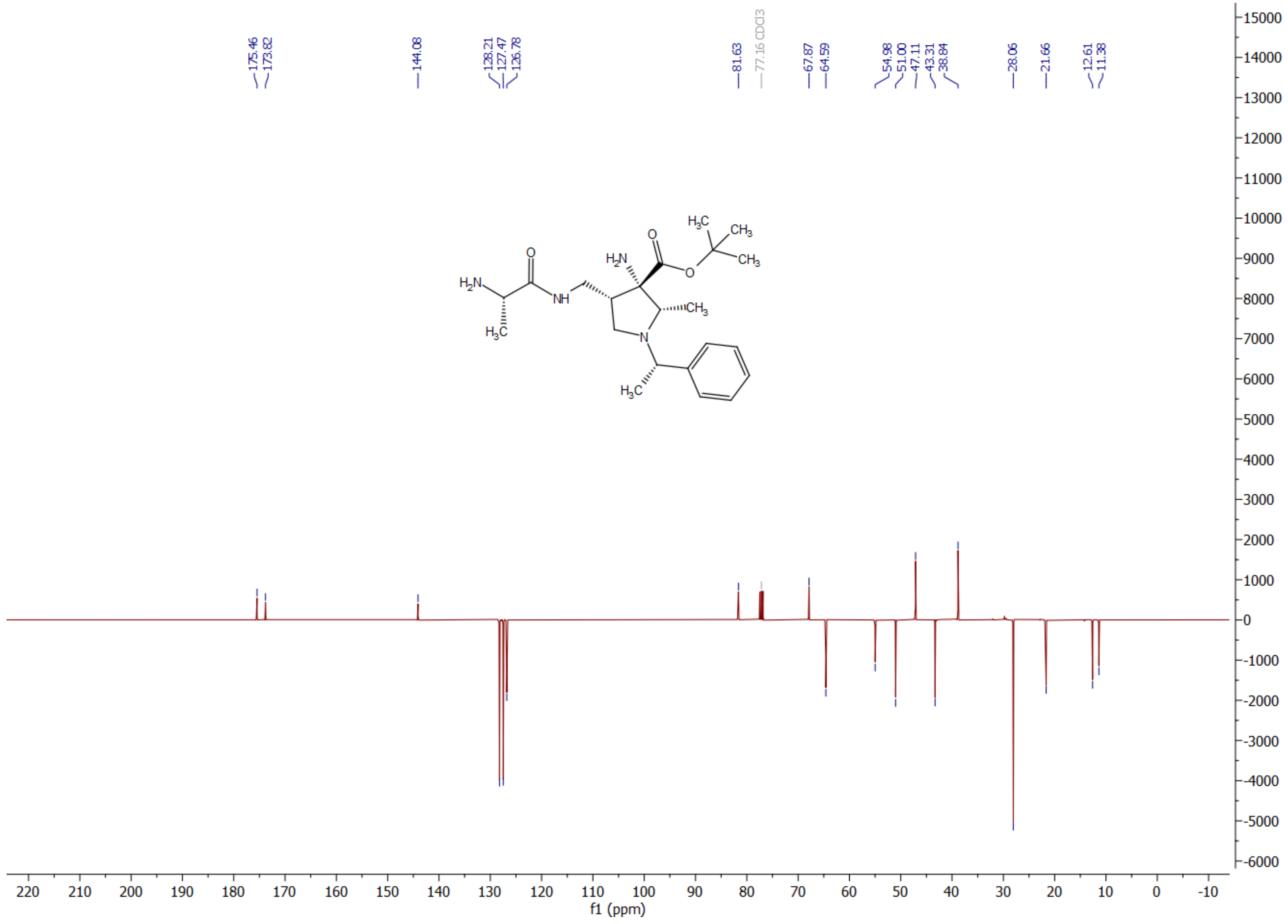

^{13}C-NMR spectrum of α/γ-peptide NH_2(Ala-(*S*,*S*,*S*,*S*)^{P}AAMP)O*t*Bu **NH_2-2AS^{P}A** measured in $CDCl_3$ at 101 MHz.

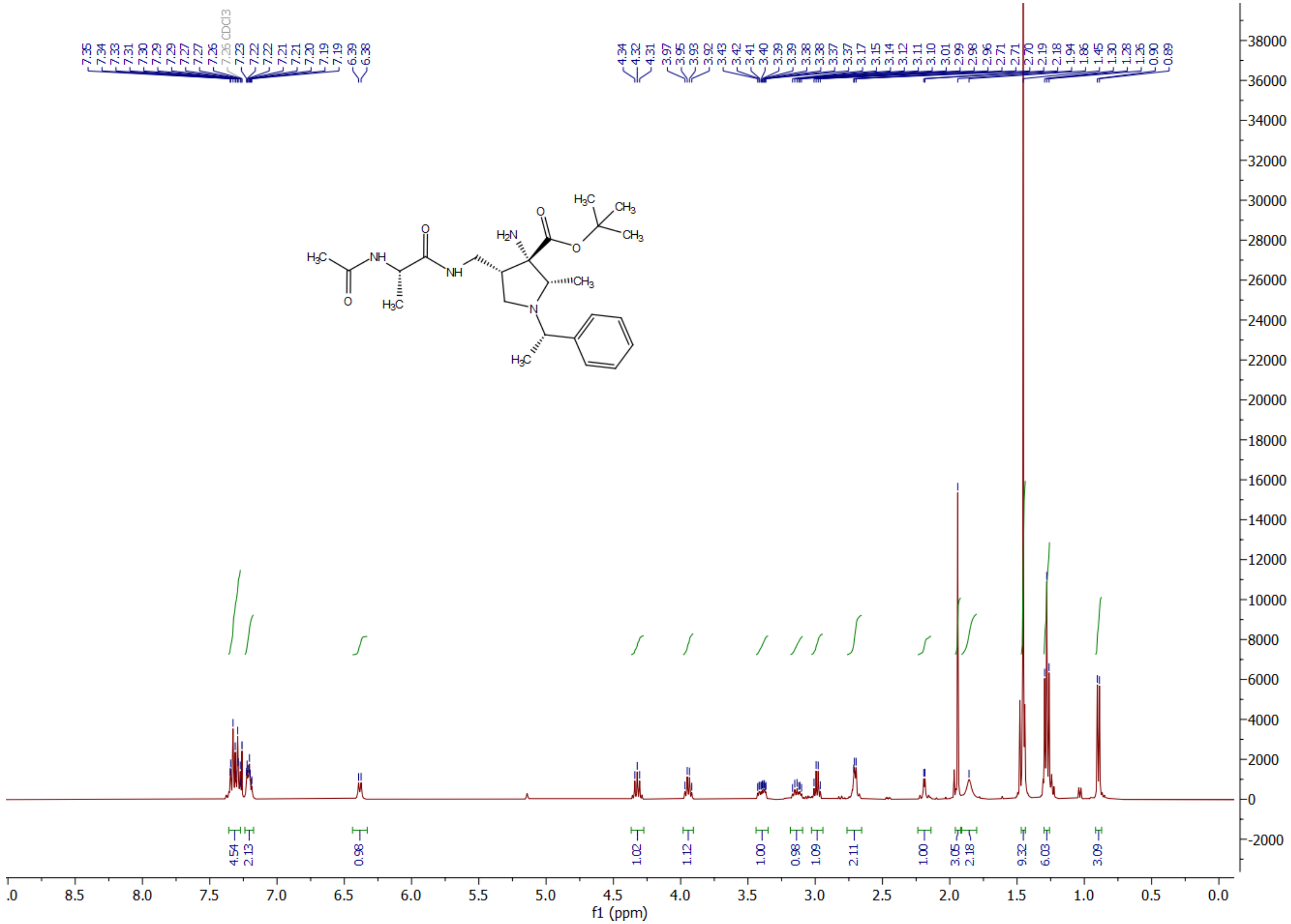

^{1}H-NMR spectrum of α/γ-peptide Ac(Ala-(*S*,*S*,*S*,*S*)[P]AAMP)O*t*Bu **Aoc-2AS[P]A** measured in $CDCl_3$ at 401 MHz.

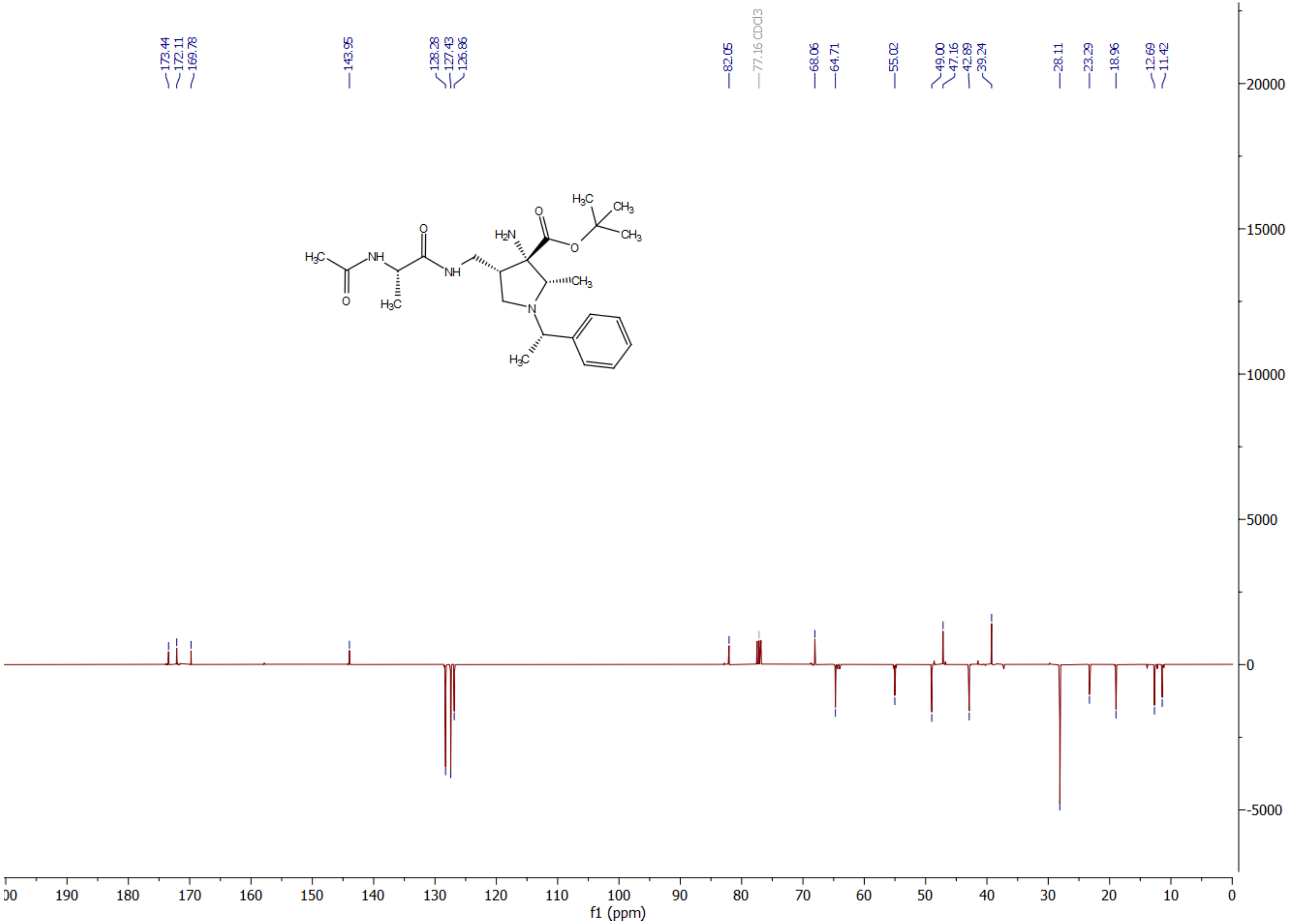

^{13}C-NMR spectrum of α/γ-peptide Ac(Ala-(*S*,*S*,*S*,*S*)^{P}AAMP)O*t*Bu **Aoc-2AS^{P}A** measured in $CDCl_3$ at 101 MHz.

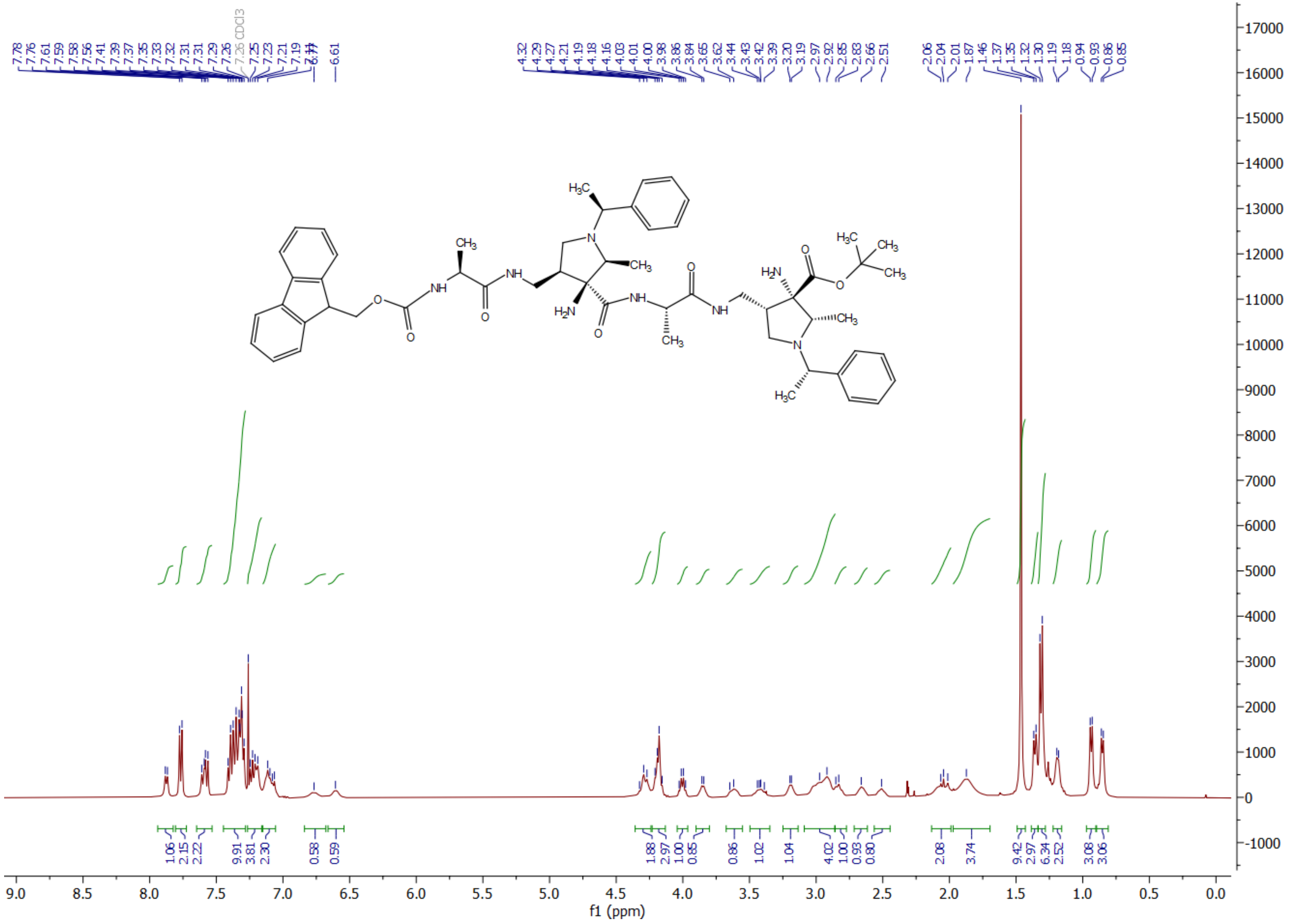

^{1}H-NMR spectrum of α/γ-peptide Fmoc(Ala-(*S*,*S*,*S*,*S*)[P]AAMP)$_2$O*t*Bu **Fmoc-4AS[P]A** measured in $CDCl_3$ at 400 MHz.

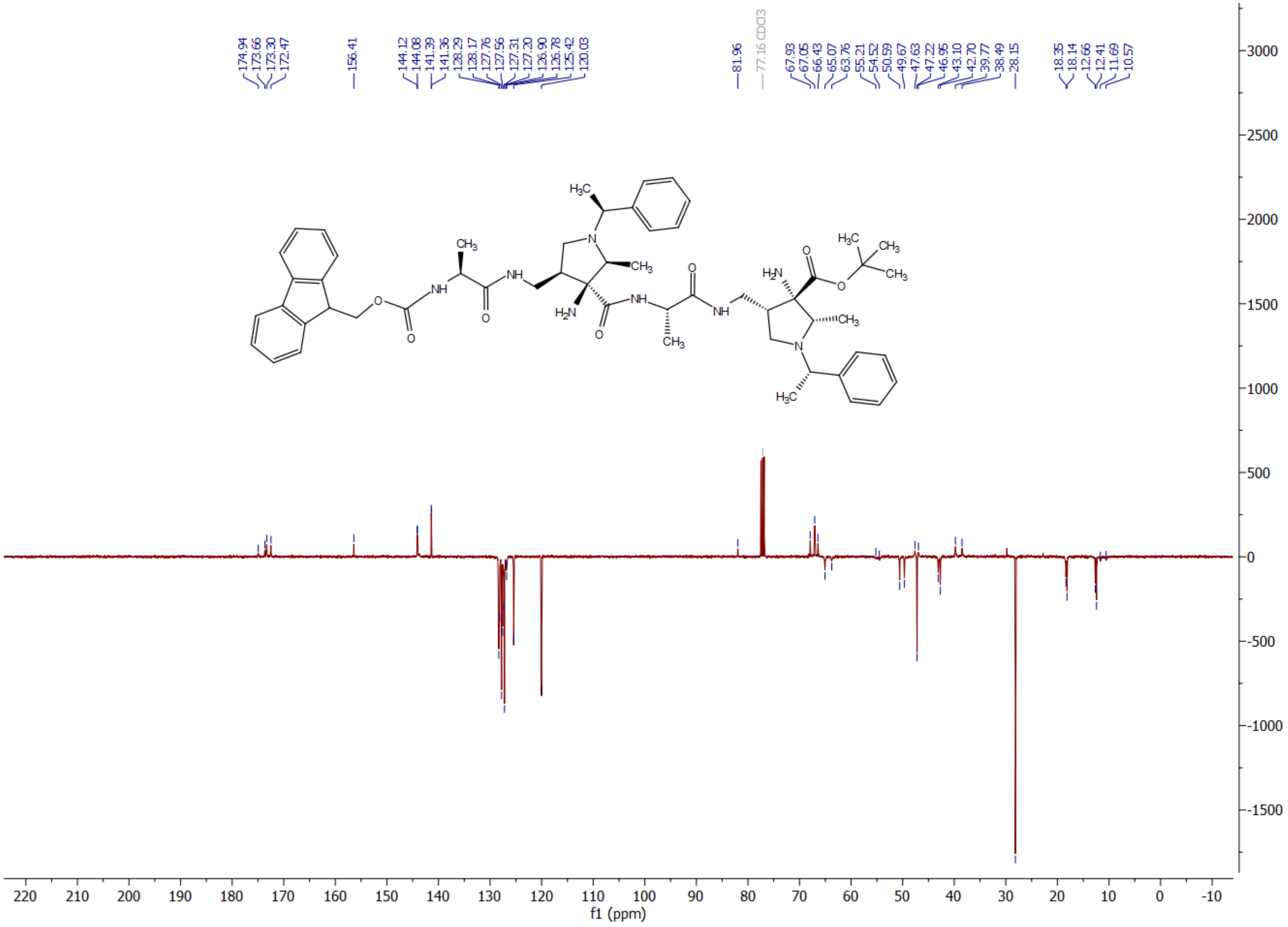

^{13}C-NMR spectrum of α/γ-peptide Fmoc(Ala-(*S*,*S*,*S*,*S*)[P]AAMP)$_2$O*t*Bu **Fmoc-4AS[P]A** measured in $CDCl_3$ at 126 MHz.

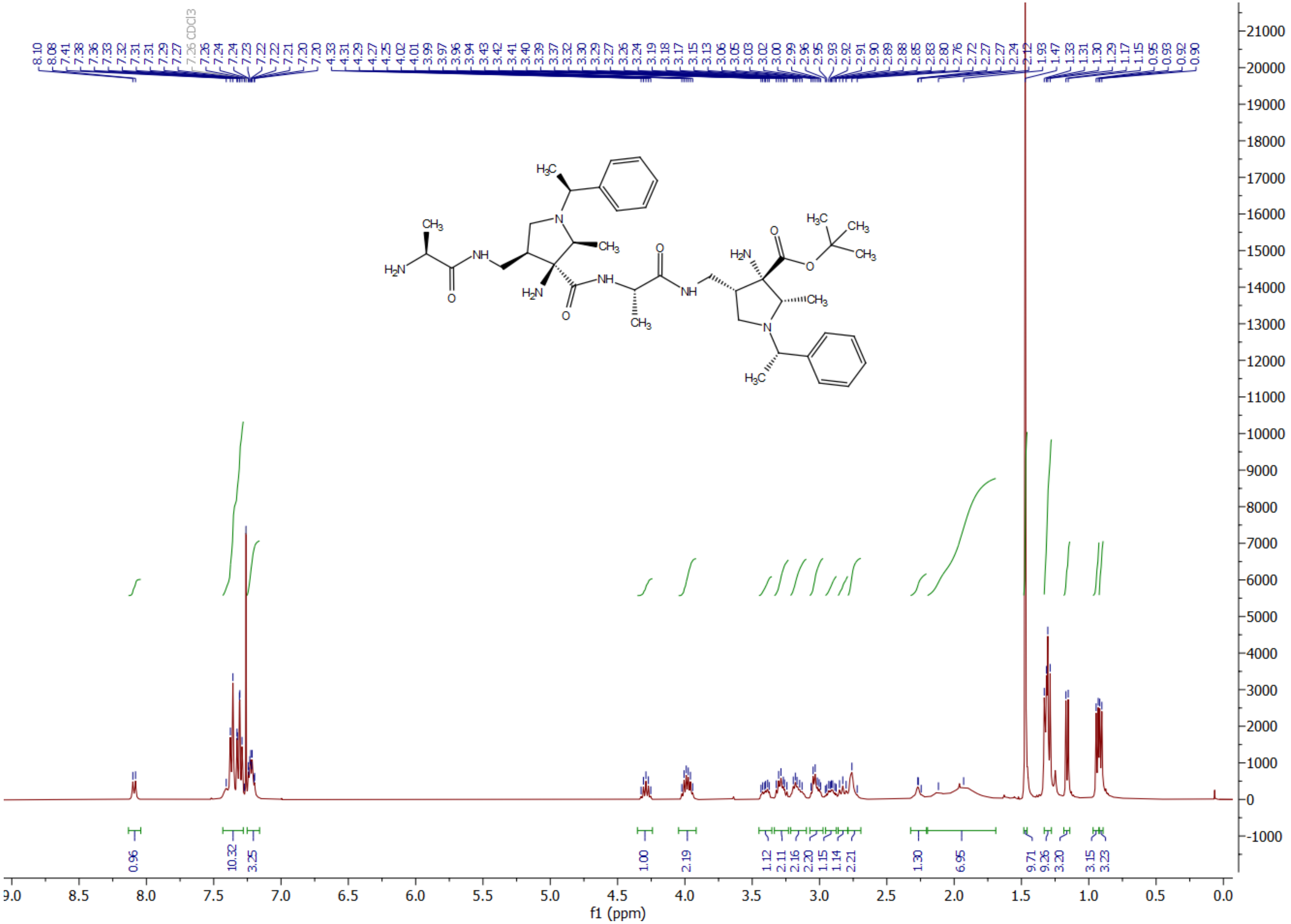

^{1}H-NMR spectrum of α/γ-peptide NH_2(Ala-(*S*,*S*,*S*,*S*)[P]AAMP)$_2$O*t*Bu **NH_2-4AS[P]A** measured in $CDCl_3$ at 401 MHz.

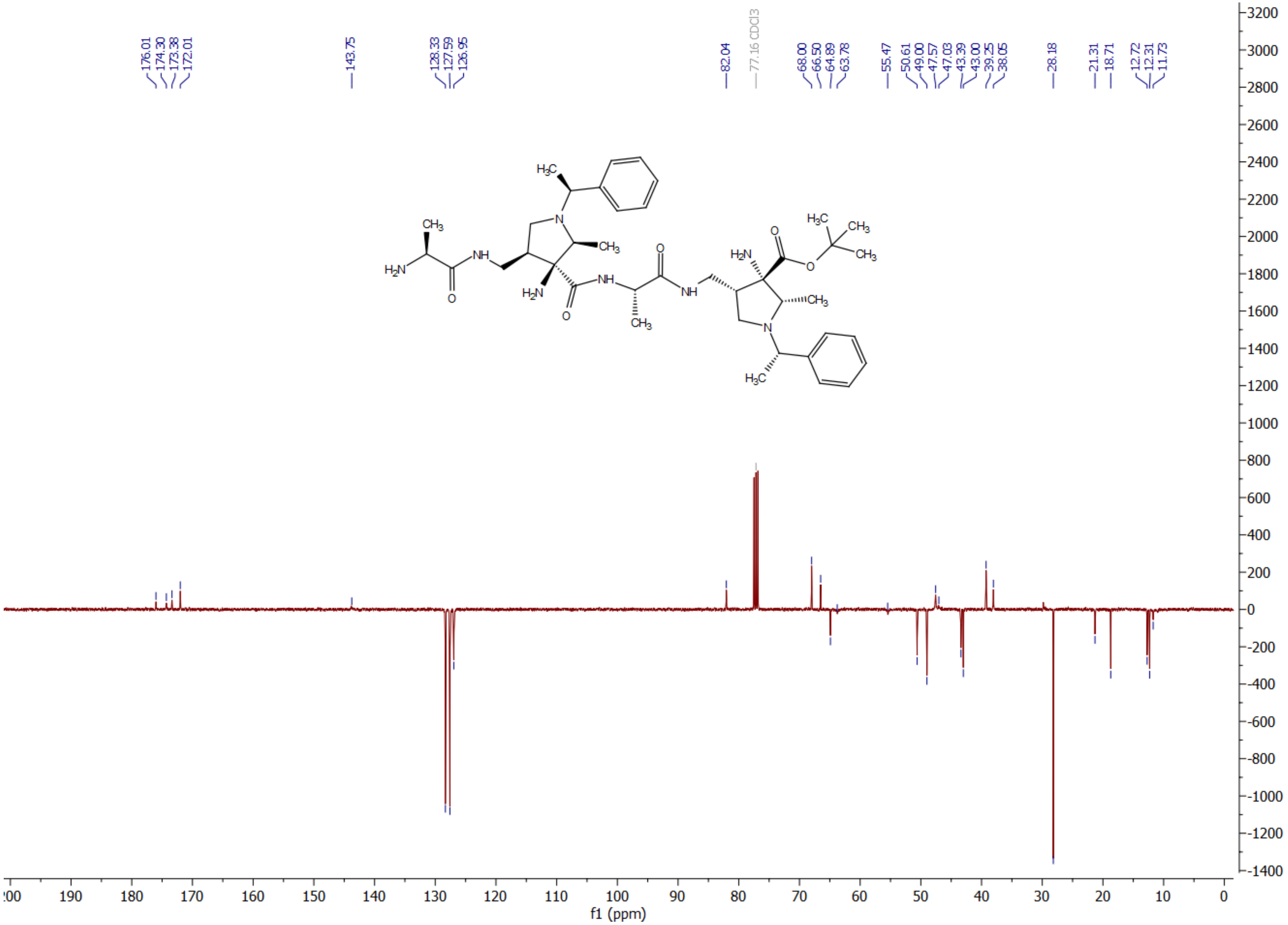

^{13}C-NMR spectrum of α/γ-peptide NH_2(Ala-(*S,S,S,S*)^{P}AAMP)$_2$O*t*Bu **NH_2c-4AS^{P}A** measured in $CDCl_3$ at 101 MHz.

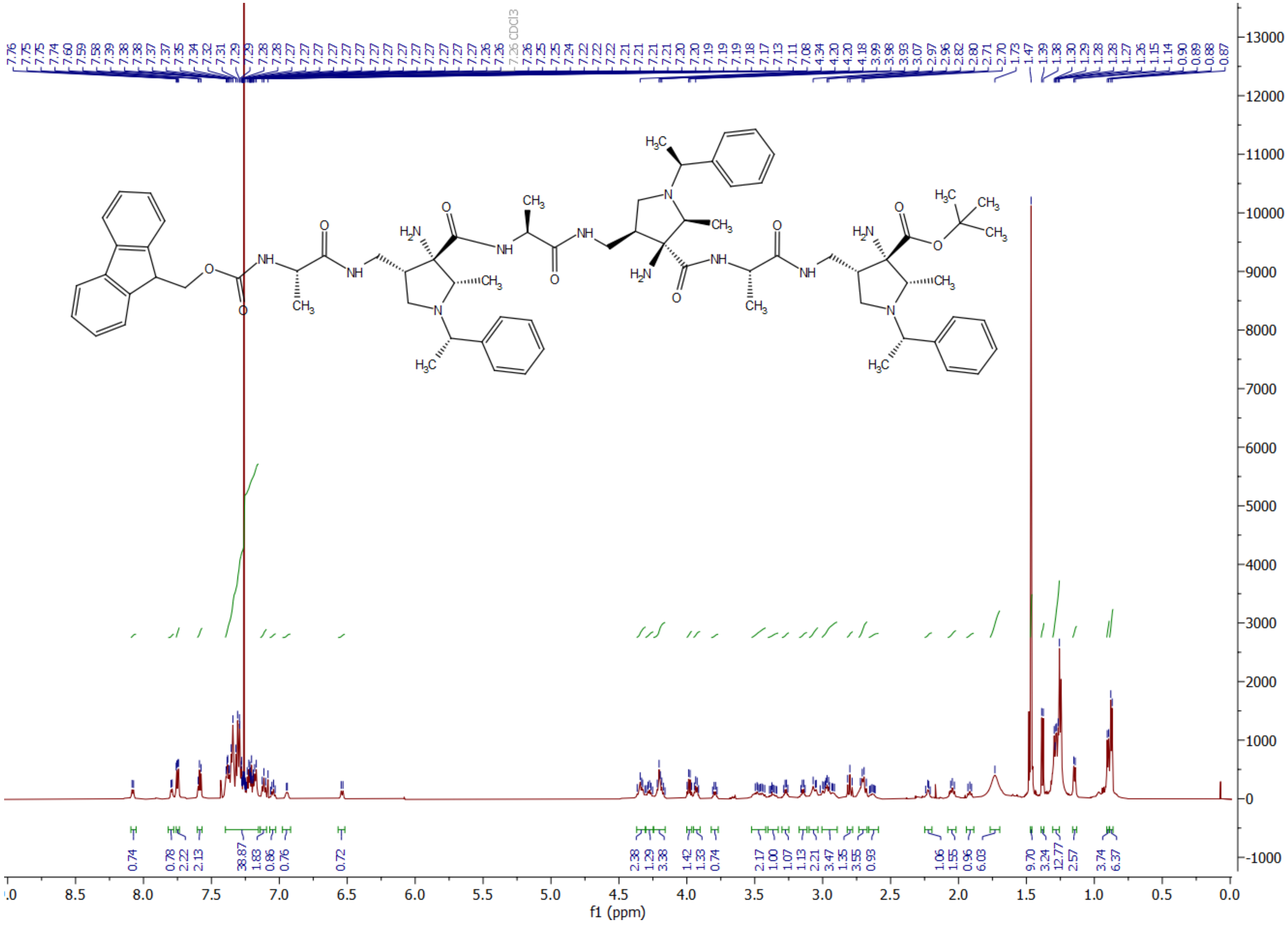

^{1}H-NMR spectrum of α/γ-peptide Fmoc(Ala-(*S*,*S*,*S*,*S*)^{P}AAMP)$_3$O*t*Bu **Fmoc-6AS^{P}A** measured in $CDCl_3$ at 600 MHz.

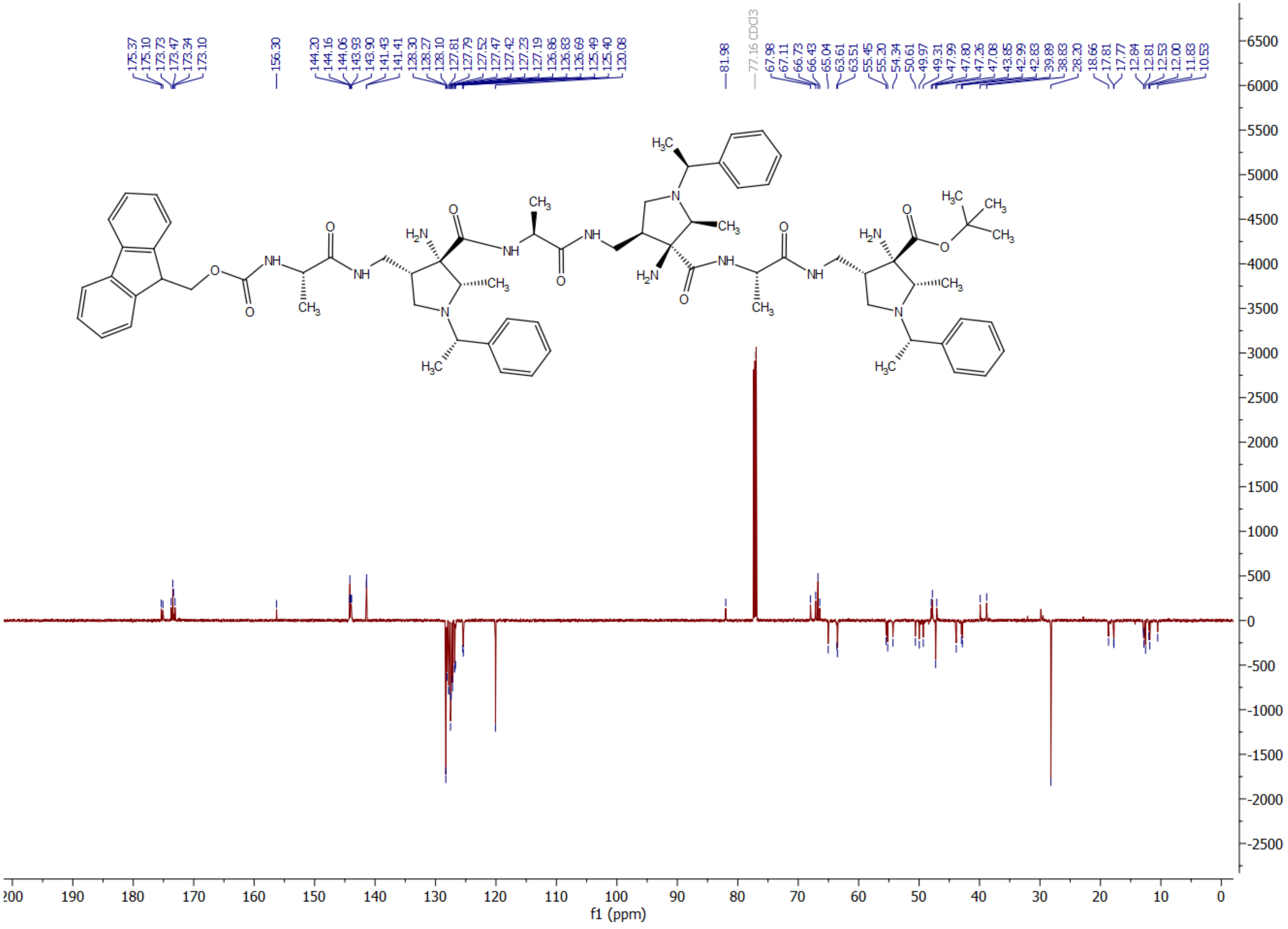

^{13}C-NMR spectrum of α/γ-peptide Fmoc(Ala-(*S*,*S*,*S*,*S*)^{P}AAMP)$_3$O*t*Bu **Fmoc-6AS^{P}A** measured in $CDCl_3$ at 151 MHz.

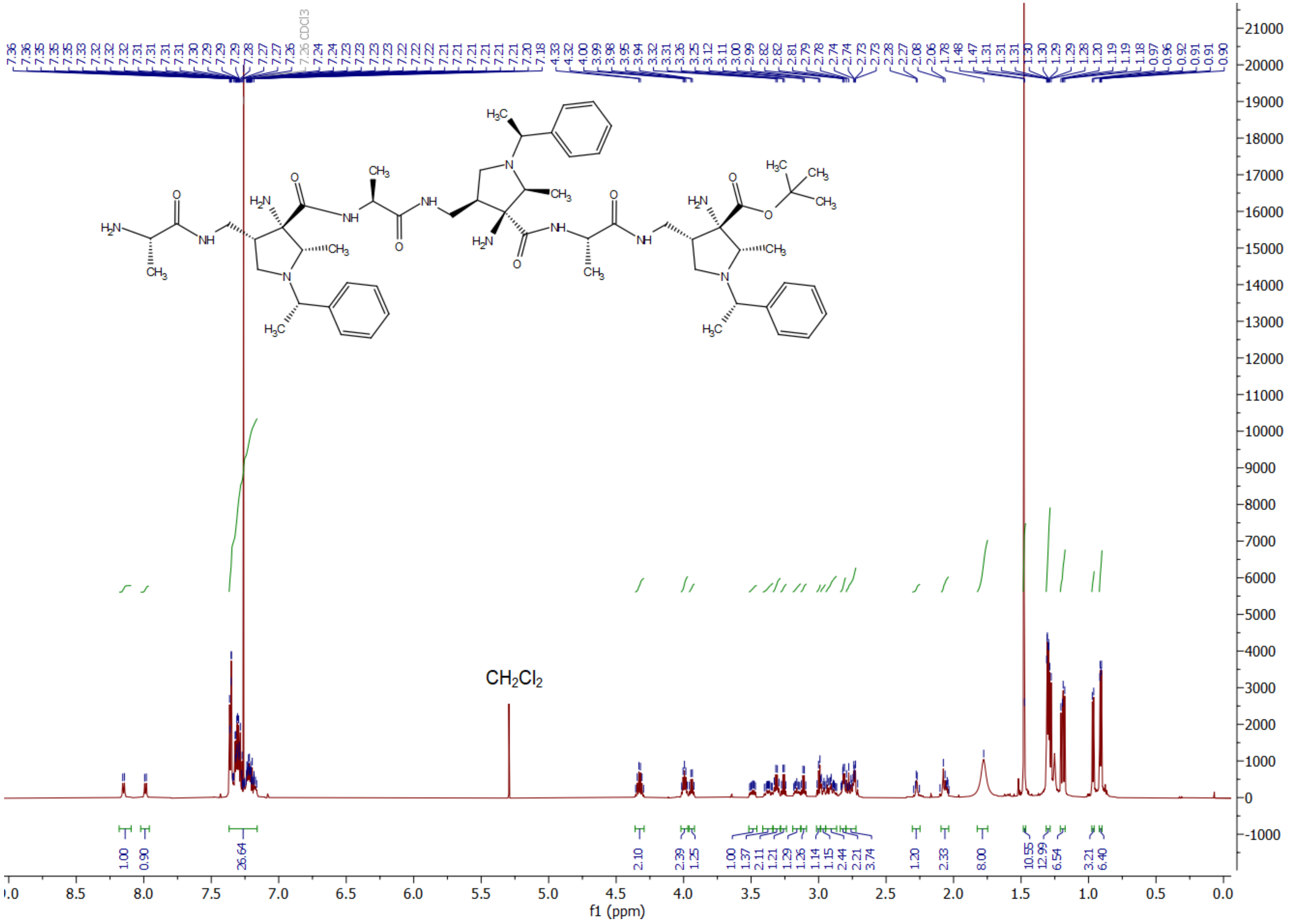

^{1}H-NMR spectrum of α/γ-peptide NH_2(Ala-(*S*,*S*,*S*,*S*)^{P}AAMP)$_3$O*t*Bu **NH_2-6AS^{P}A** measured in $CDCl_3$ at 600 MHz.

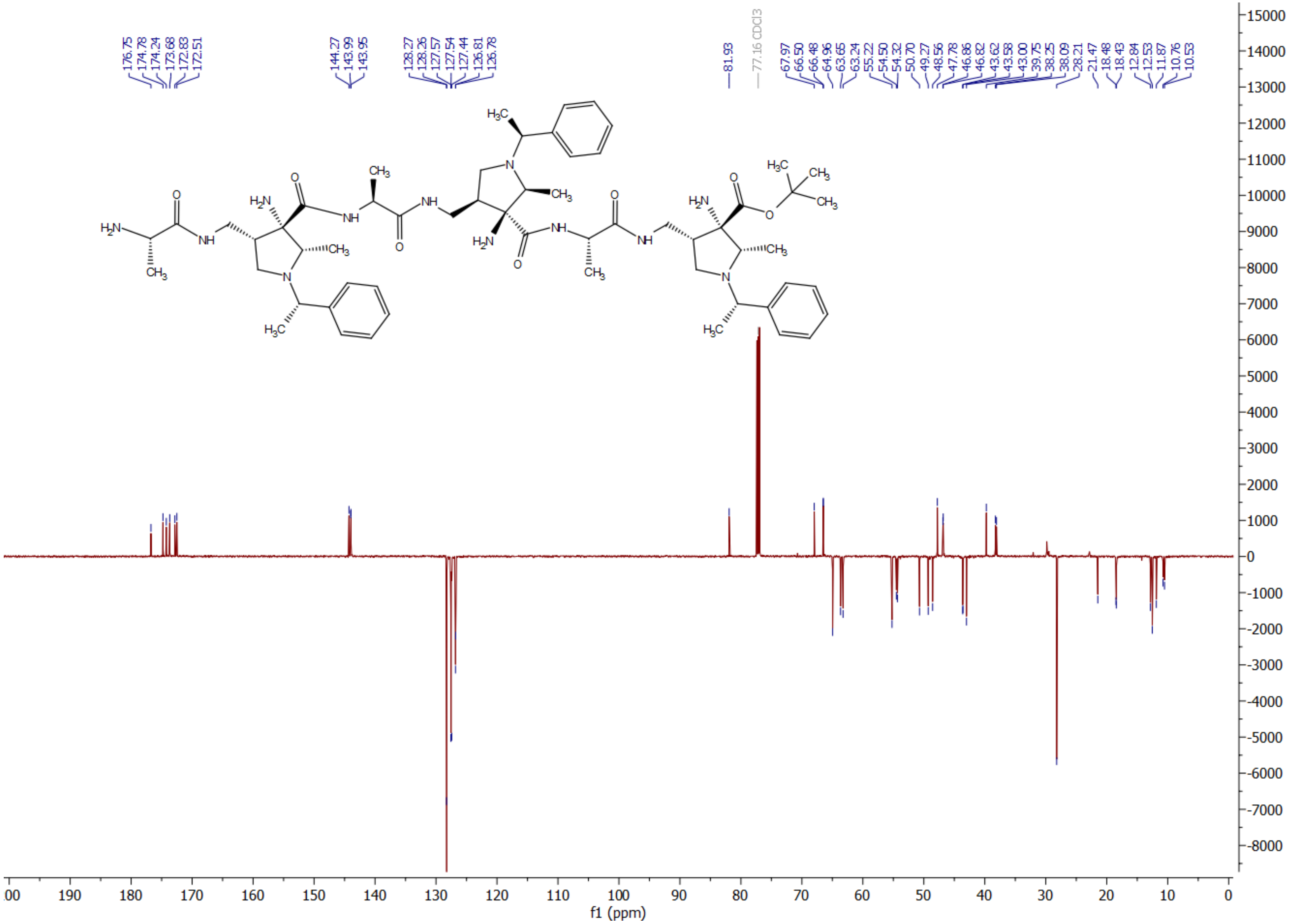

^{13}C-NMR spectrum of α/γ-peptide NH_2(Ala-(*S*,*S*,*S*,*S*)[P]AAMP)$_3$O*t*Bu **NH_2c-6AS[P]A** measured in $CDCl_3$ at 151 MHz.

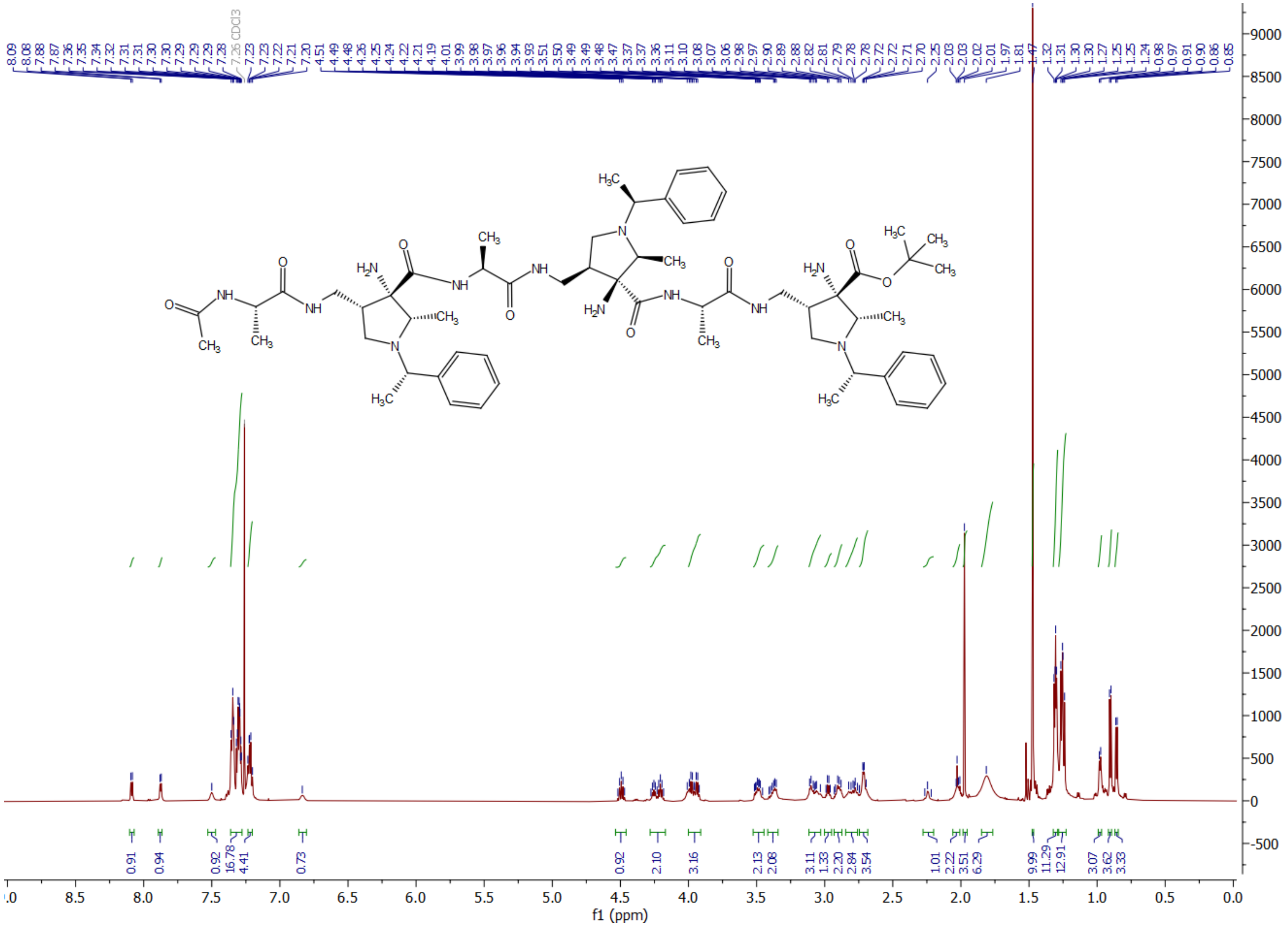

^{1}H-NMR spectrum of α/γ-peptide Ac(Ala-(*S*,*S*,*S*,*S*)[P]AAMP)$_3$O*t*Bu **Ac-6AS[P]A** measured in $CDCl_3$ at 600 MHz.

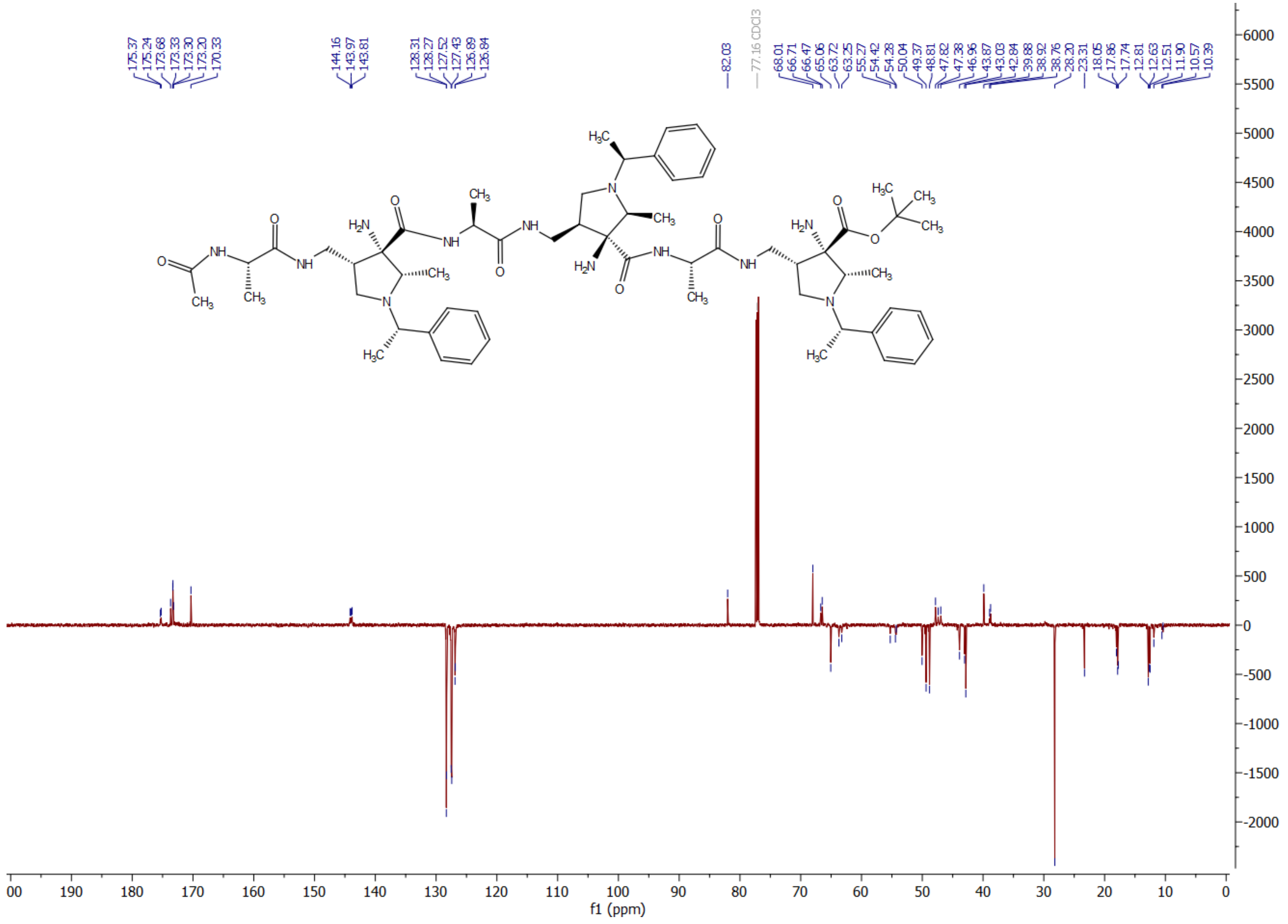

^{13}C-NMR spectrum of α/γ-peptide Ac(Ala-(*S*,*S*,*S*,*S*)^{P}AAMP)$_3$O*t*Bu **Ac-6AS^{P}A** measured in $CDCl_3$ at 151 MHz.

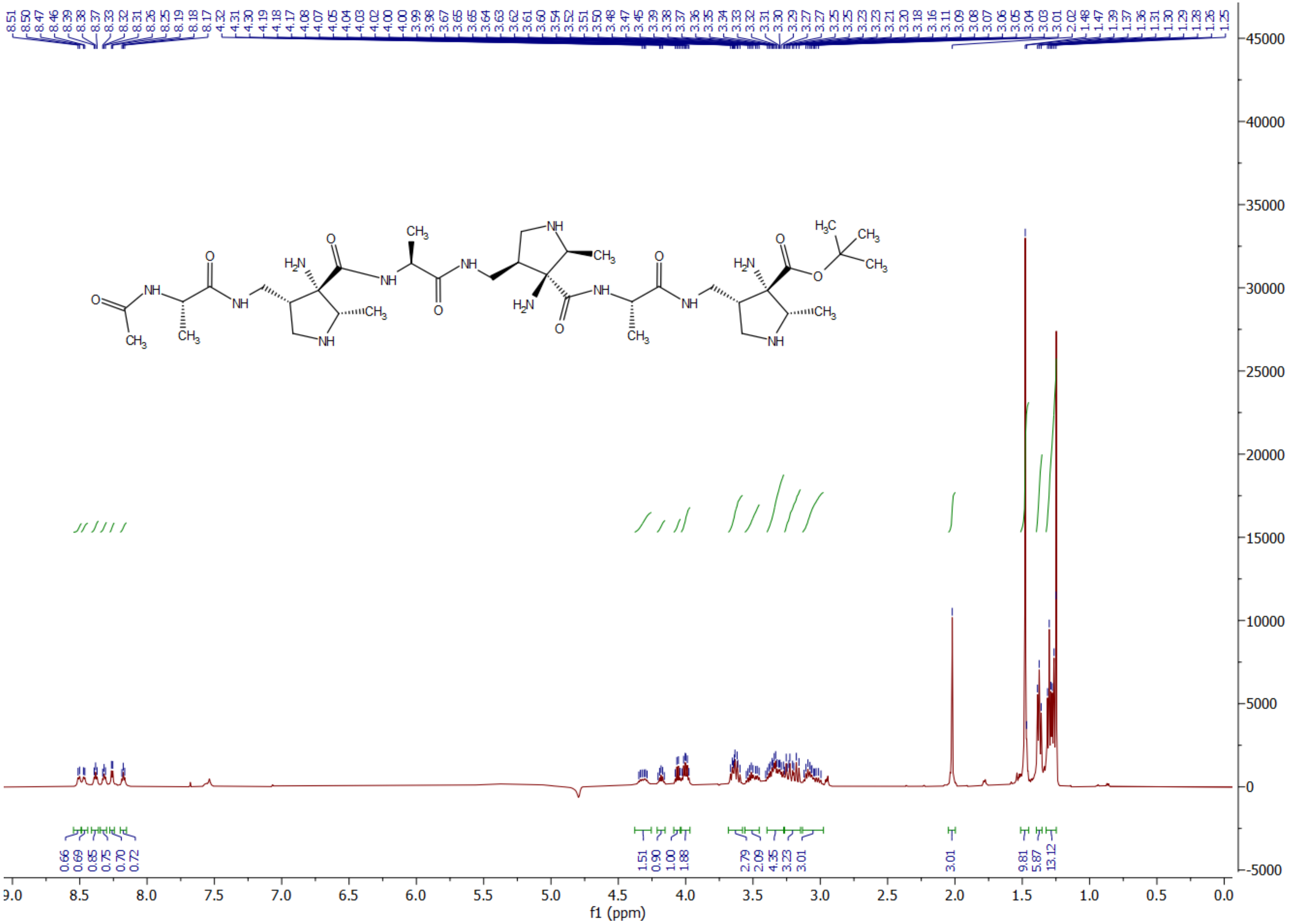

^{1}H-NMR spectrum of α/γ-peptide Ac(Ala-(*S*,*S*,*S*)AAMP)$_3$O*t*Bu **Ac-6AS^{H}A** measured in $H_2O:D_2O$ 9:1, acidified with CD_3COOD to pH of 4 at 600 MHz.

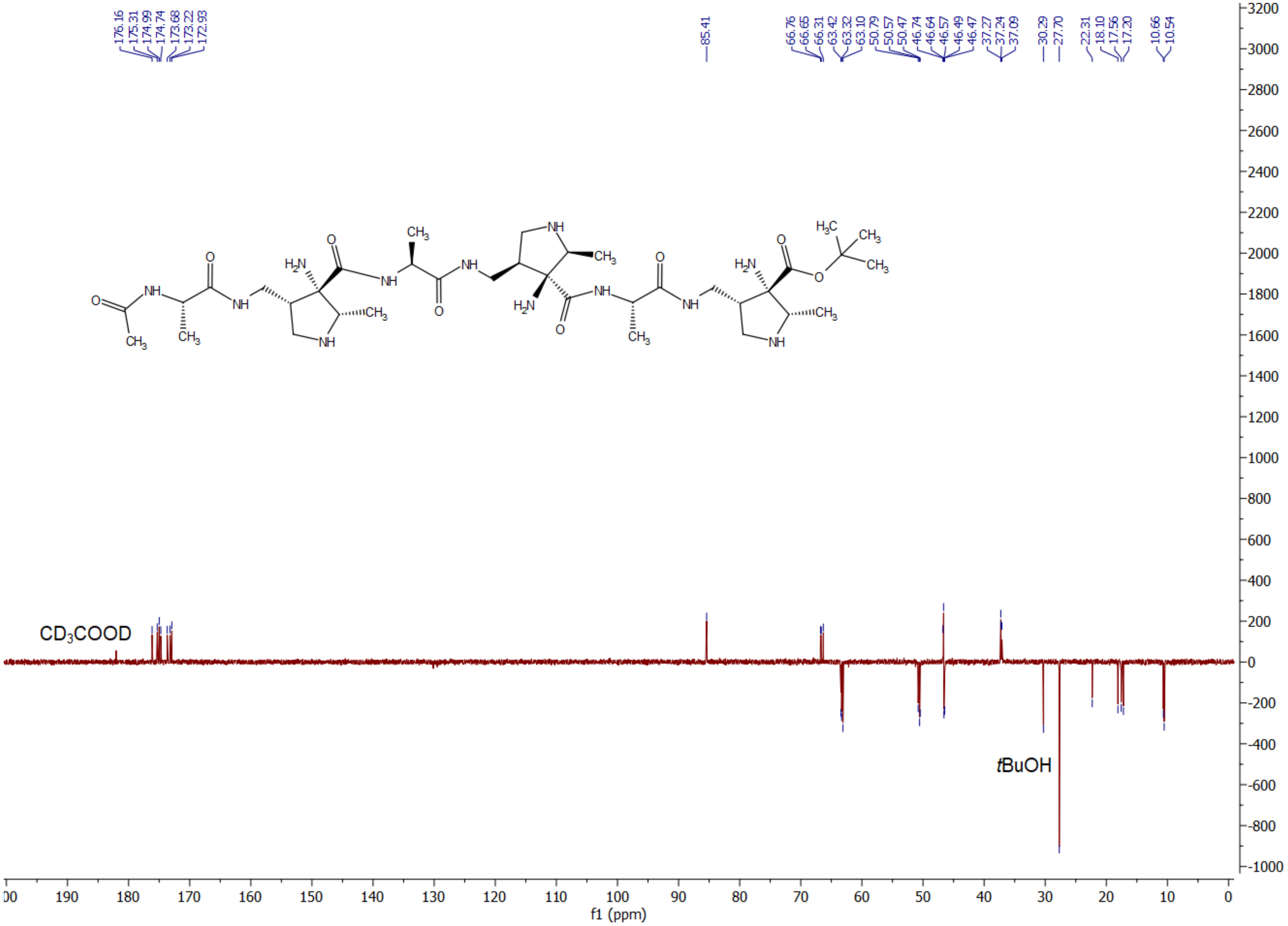

^{13}C-NMR spectrum of α/γ-peptide Ac(Ala-(*S*,*S*,*S*)AAMP)$_3$O*t*Bu **Ac-6AS^{H}A** measured in H_2O:D_2O 9:1, acidified with CD_3COOD to pH of 4 at 151 MHz.

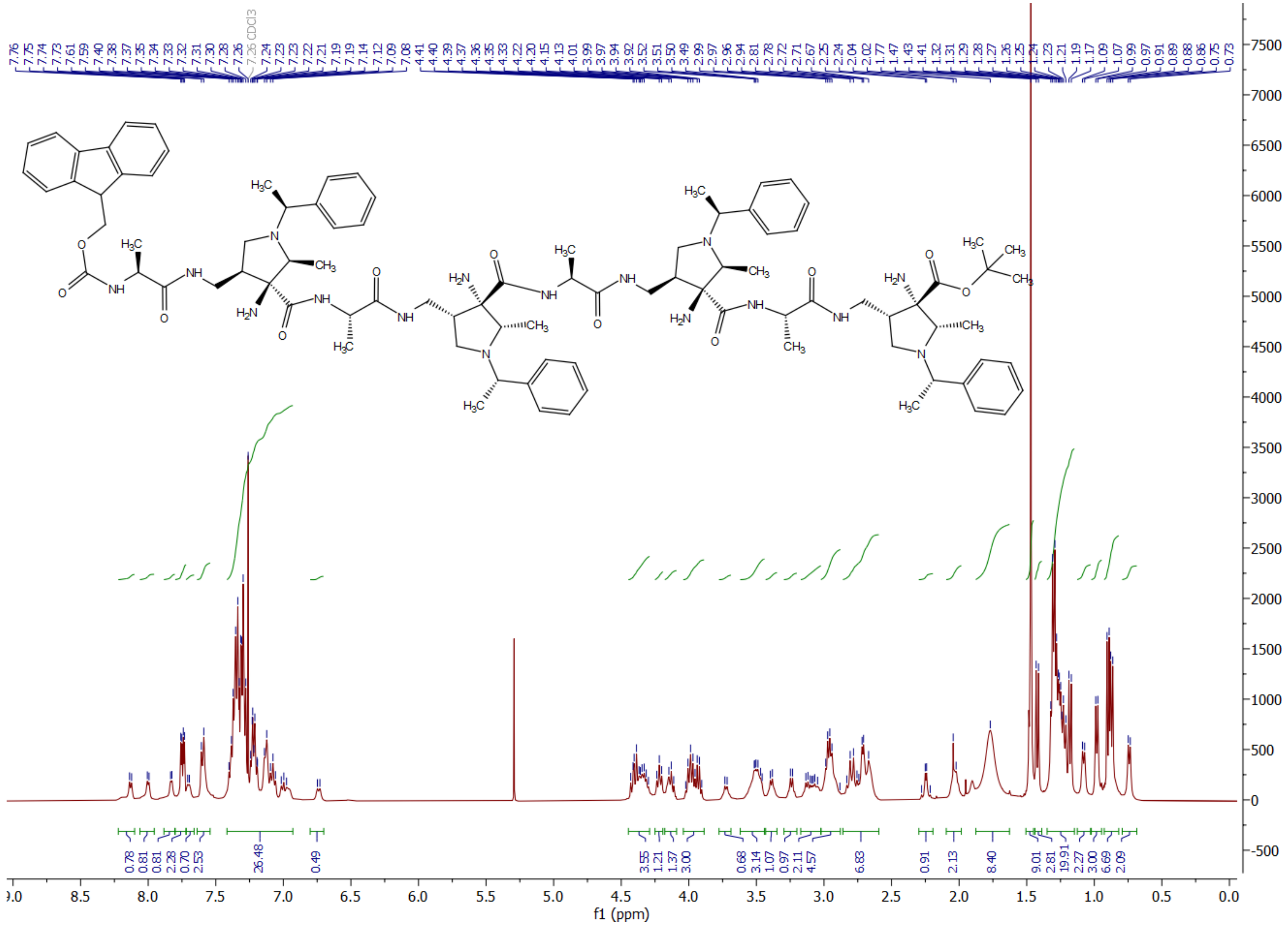

^{1}H-NMR spectrum of α/γ-peptide Fmoc(Ala-(*S*,*S*,*S*,*S*)[P]AAMP)$_4$O*t*Bu **Fmoc-8AS[P]A** measured in $CDCl_3$ at 401 MHz.

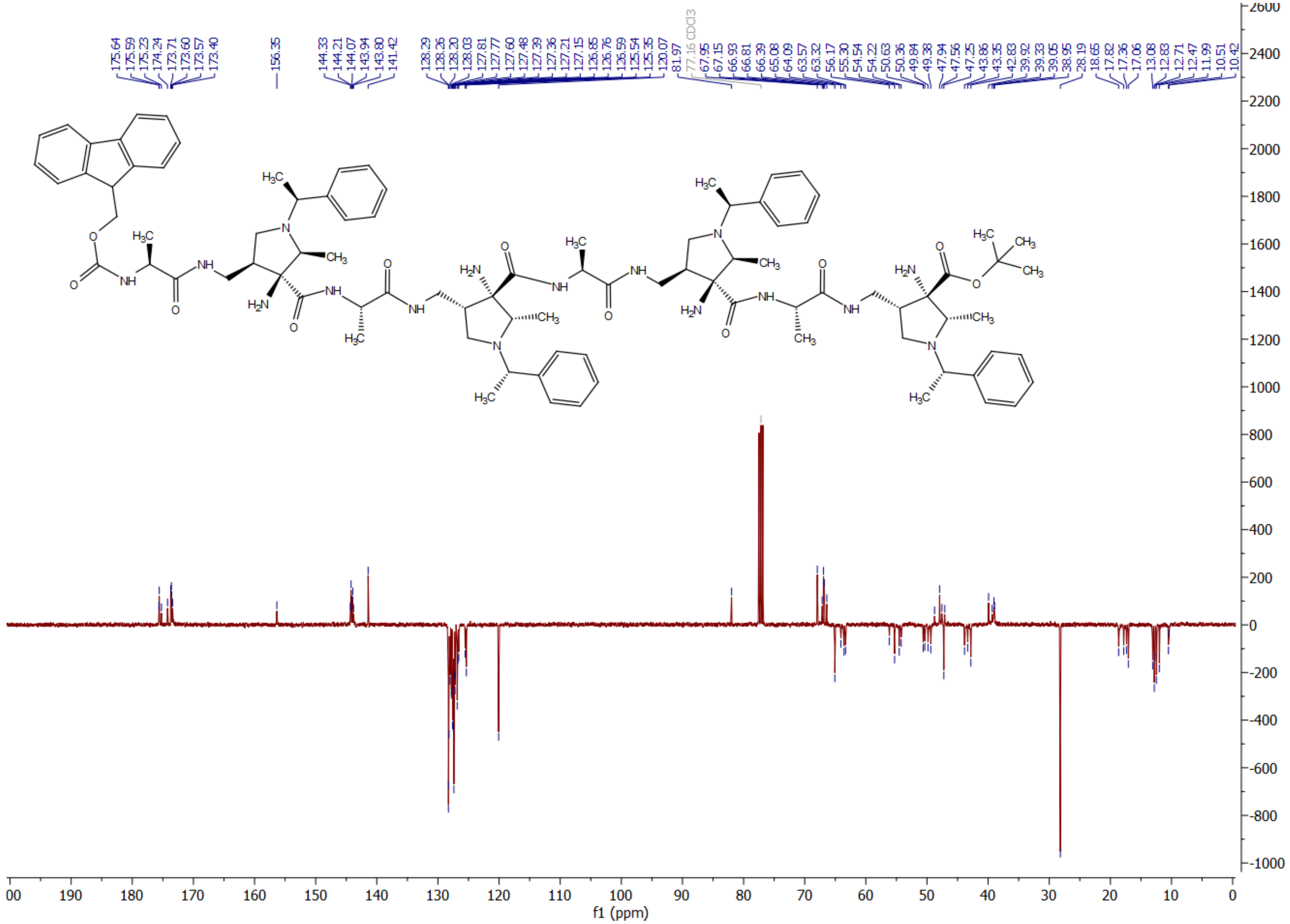

^{13}C-NMR spectrum of α/γ-peptide Fmoc(Ala-(*S*,*S*,*S*,*S*)[P]AAMP)$_4$O*t*Bu **Fmoc-8AS[P]A** measured in $CDCl_3$ at 101 MHz.

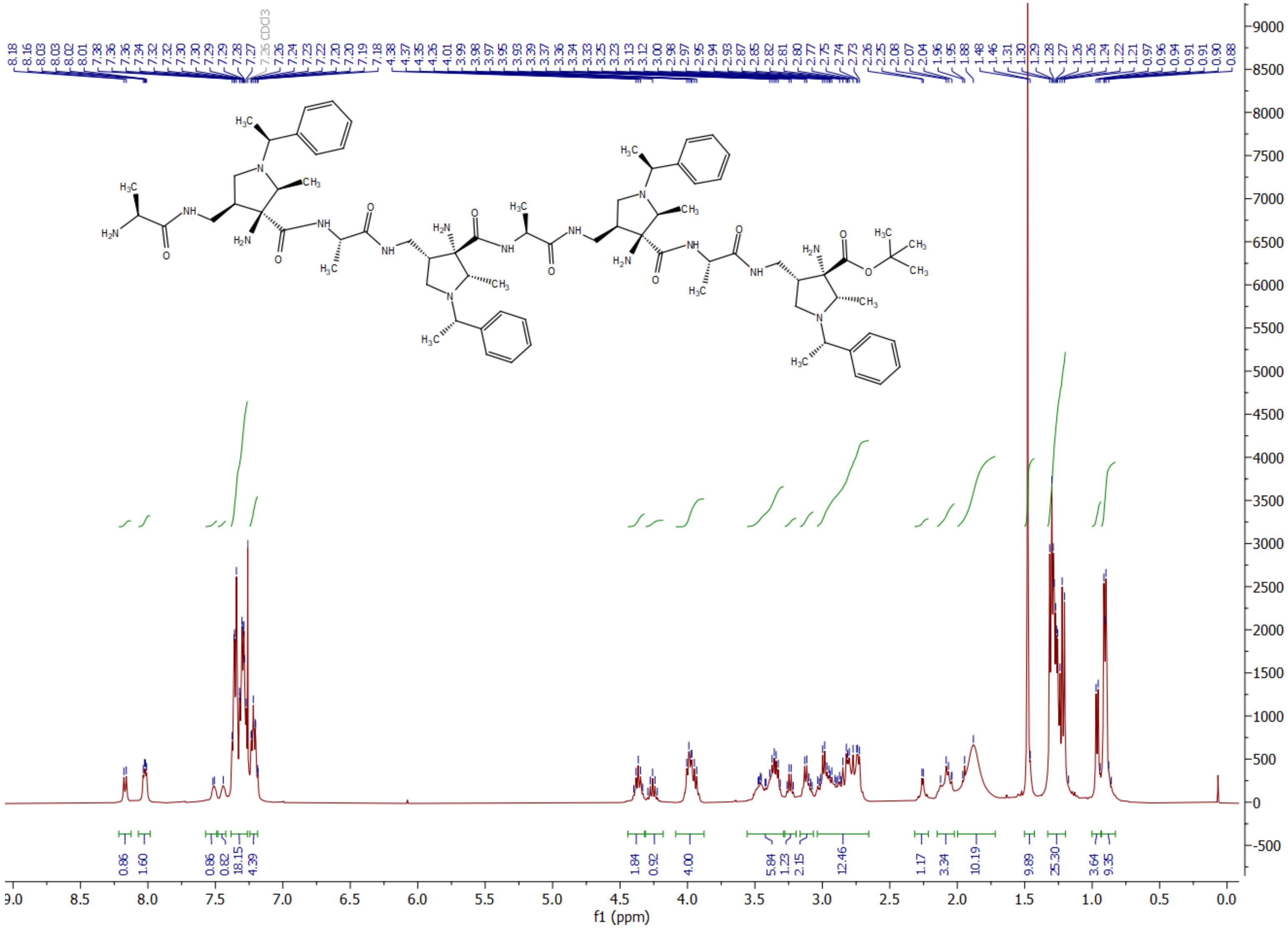

^{1}H-NMR spectrum of α/γ-peptide **NH_2**(Ala-(*S*,*S*,*S*,*S*)^{P}AAMP)$_4$O*t*Bu **NH_2-8AS^{P}A** measured in $CDCl_3$ at 401 MHz.

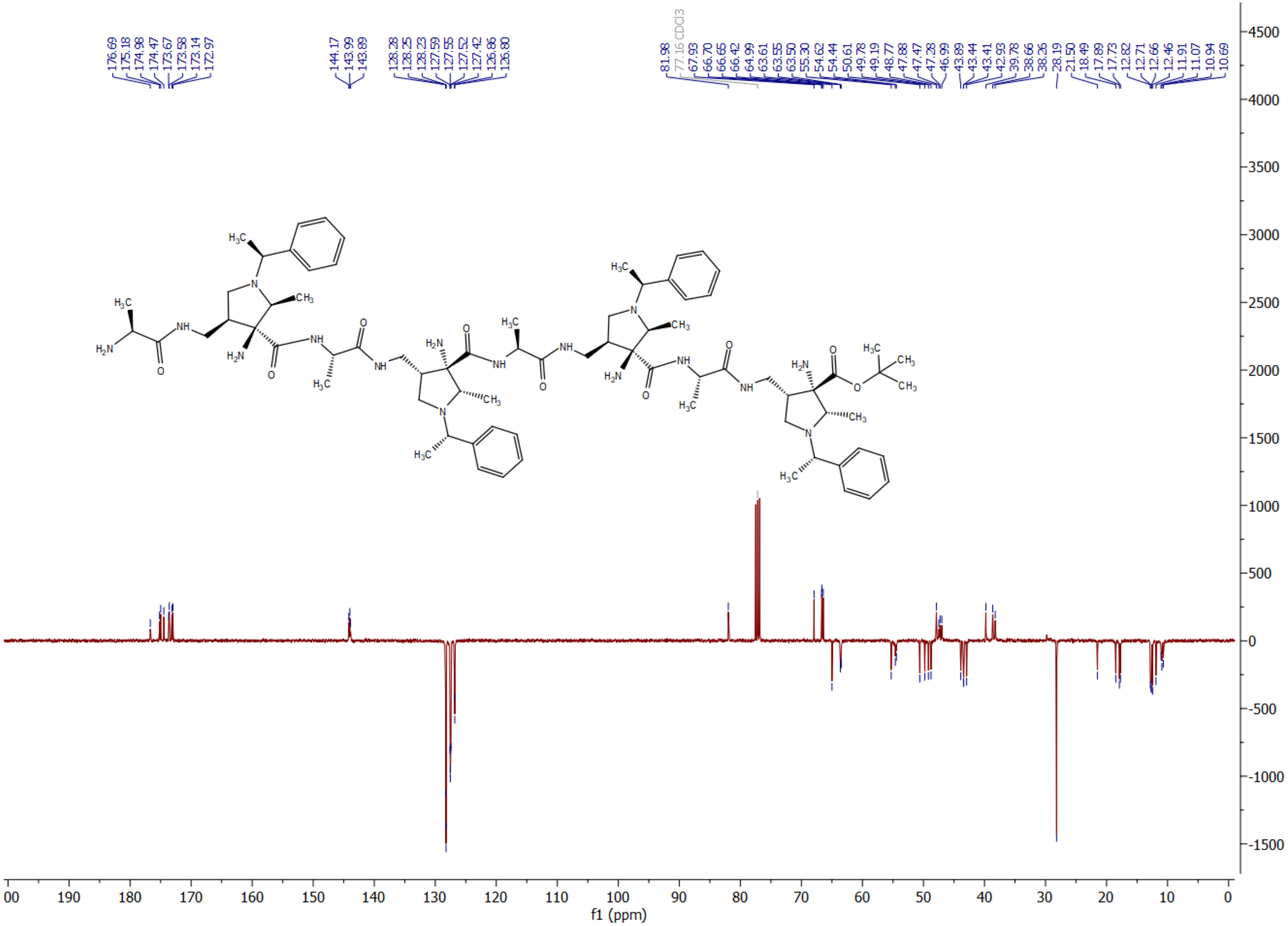

^{13}C-NMR spectrum of α/γ-peptide **NH₂**(Ala-(*S,S,S,S*)[P]AAMP)₄O*t*Bu **NH₂-8AS[P]A** measured in $CDCl_3$ at 101 MHz.

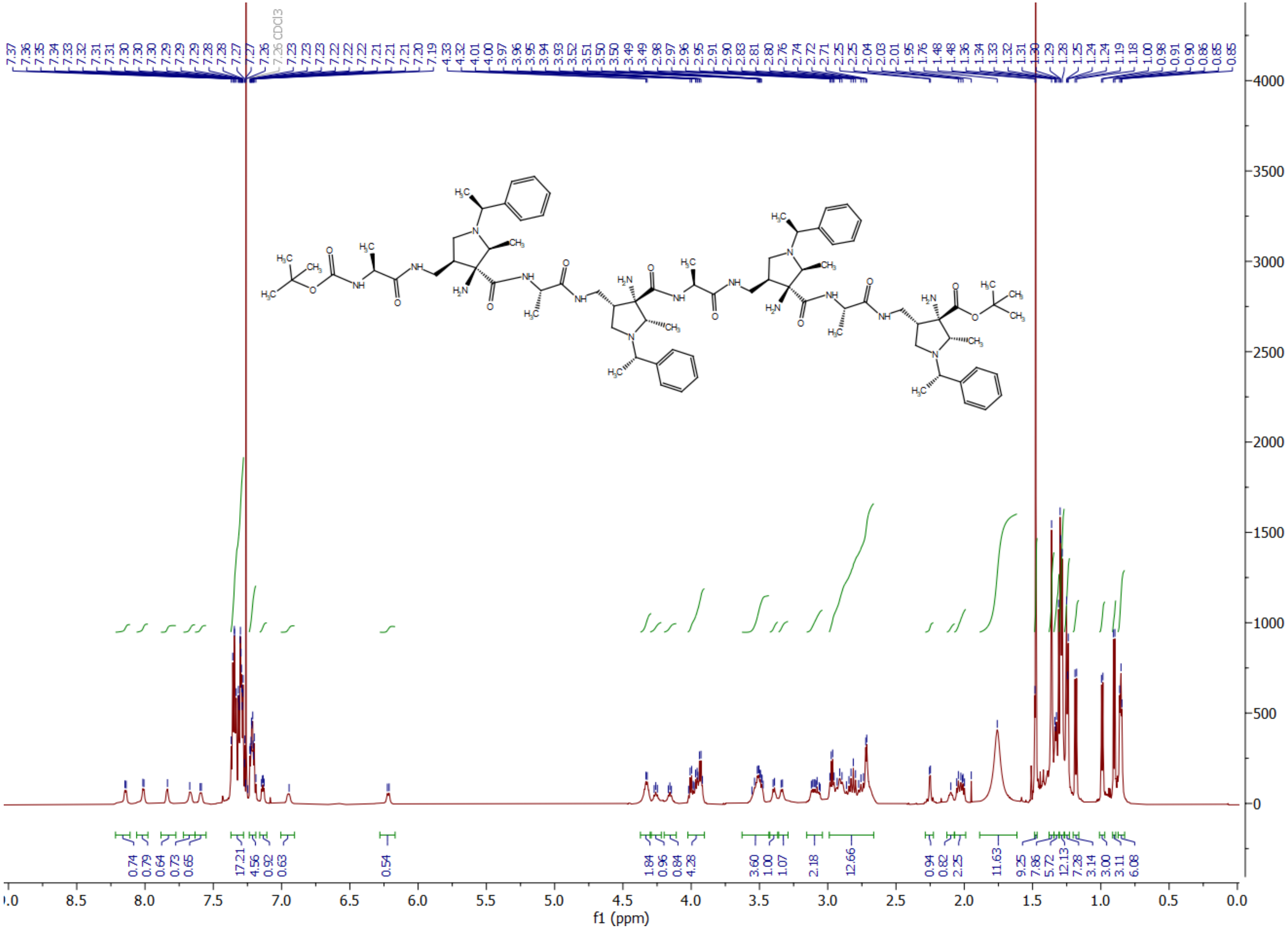

^{1}H-NMR spectrum of α/γ-peptide **Boc**(Ala-(*S*,*S*,*S*,*S*)^{P}AAMP)$_4$O*t*Bu **Boc-8AS^{P}A** measured in $CDCl_3$ at 600 MHz.

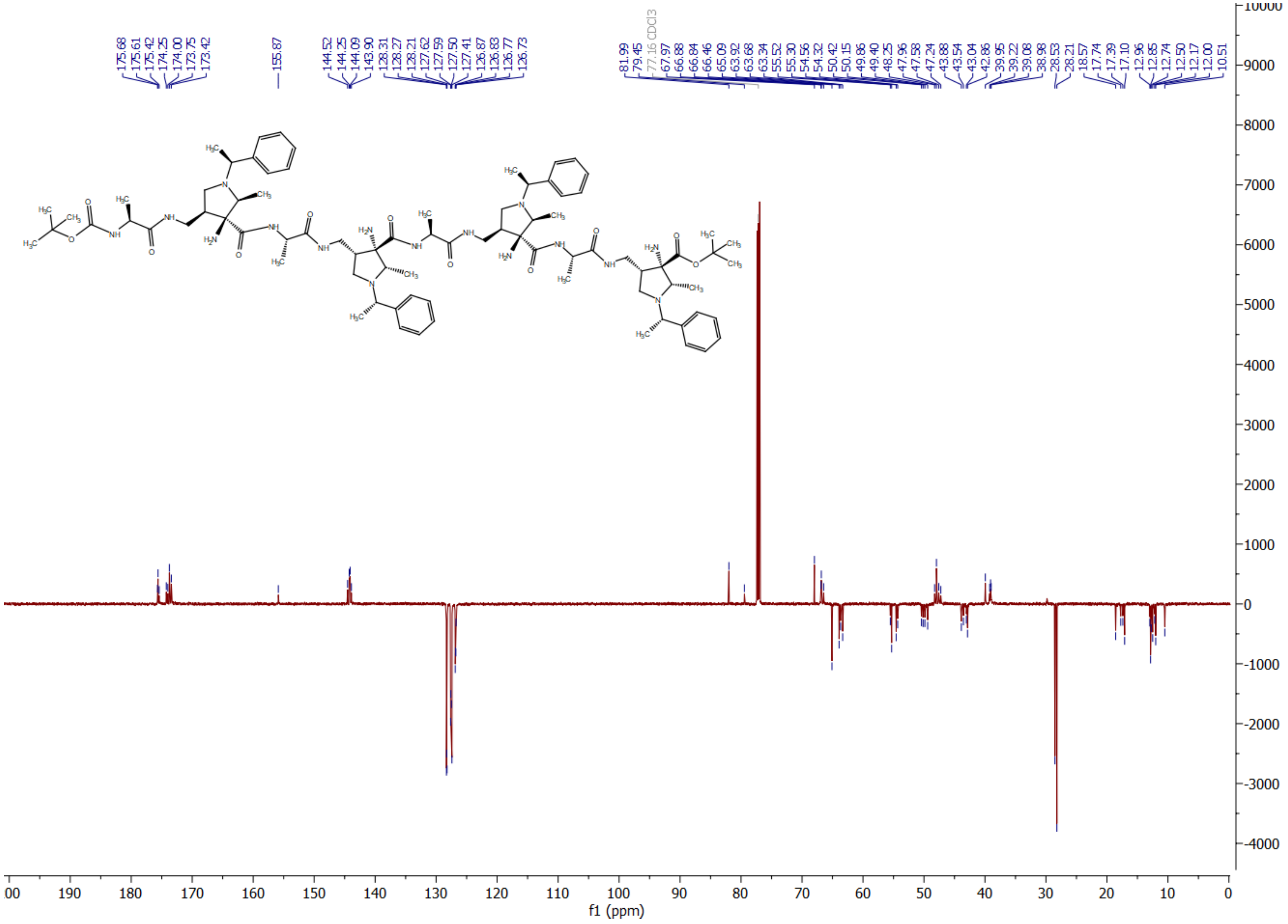

^{13}C-NMR spectrum of α/γ-peptide **Boc**(Ala-(*S*,*S*,*S*,*S*)^{P}AAMP)$_4$O*t*Bu **Boc-8AS^{P}A** measured in $CDCl_3$ at 151 MHz.

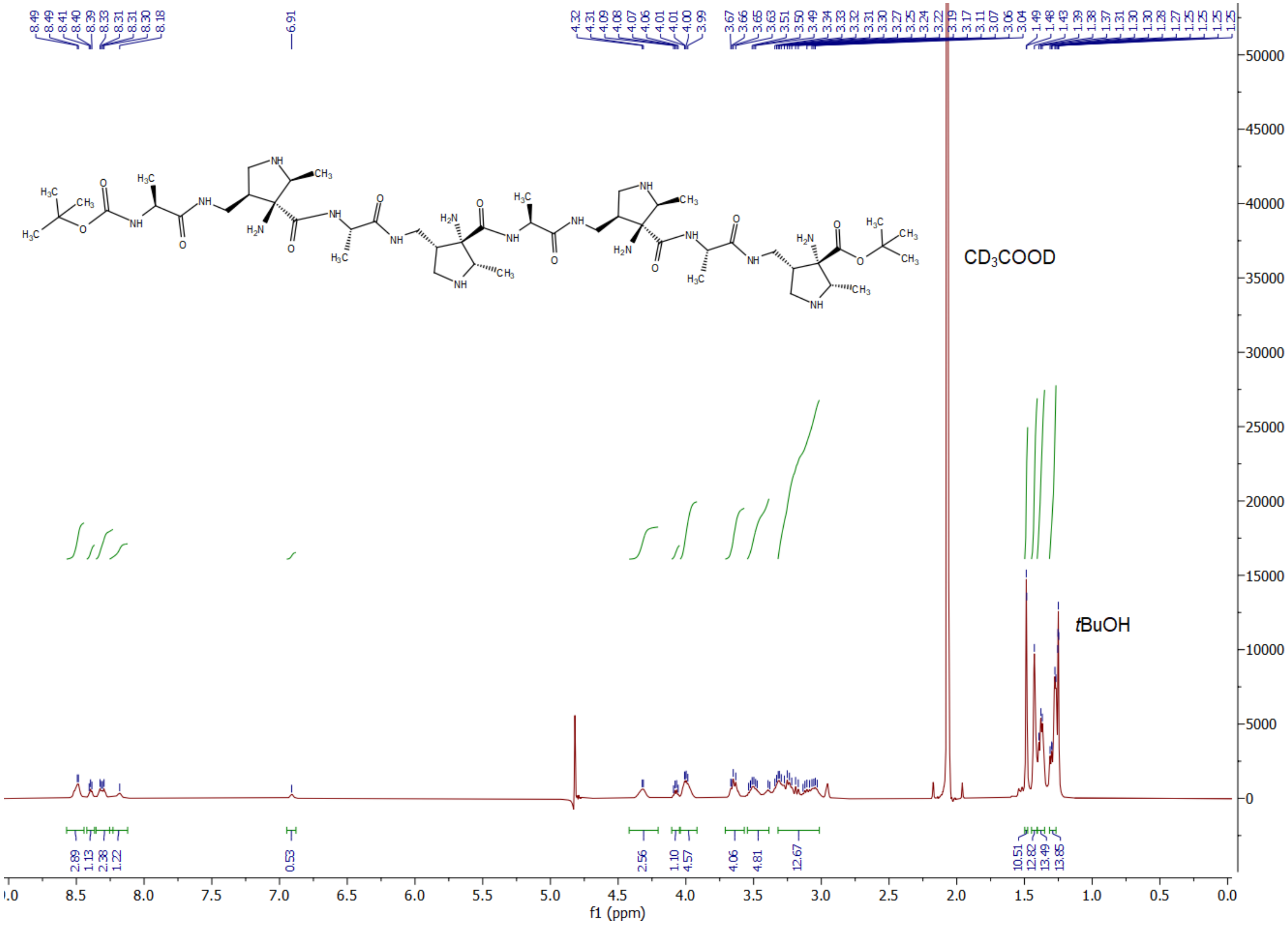

^{1}H-NMR spectrum of α/γ-peptide Boc(Ala-(*S*,*S*,*S*)AAMP)$_4$O*t*Bu **Boc-8AS^{H}A** measured in H_2O:D_2O 9:1, acidified with CD_3COOD to pH of 4 at 600 MHz.

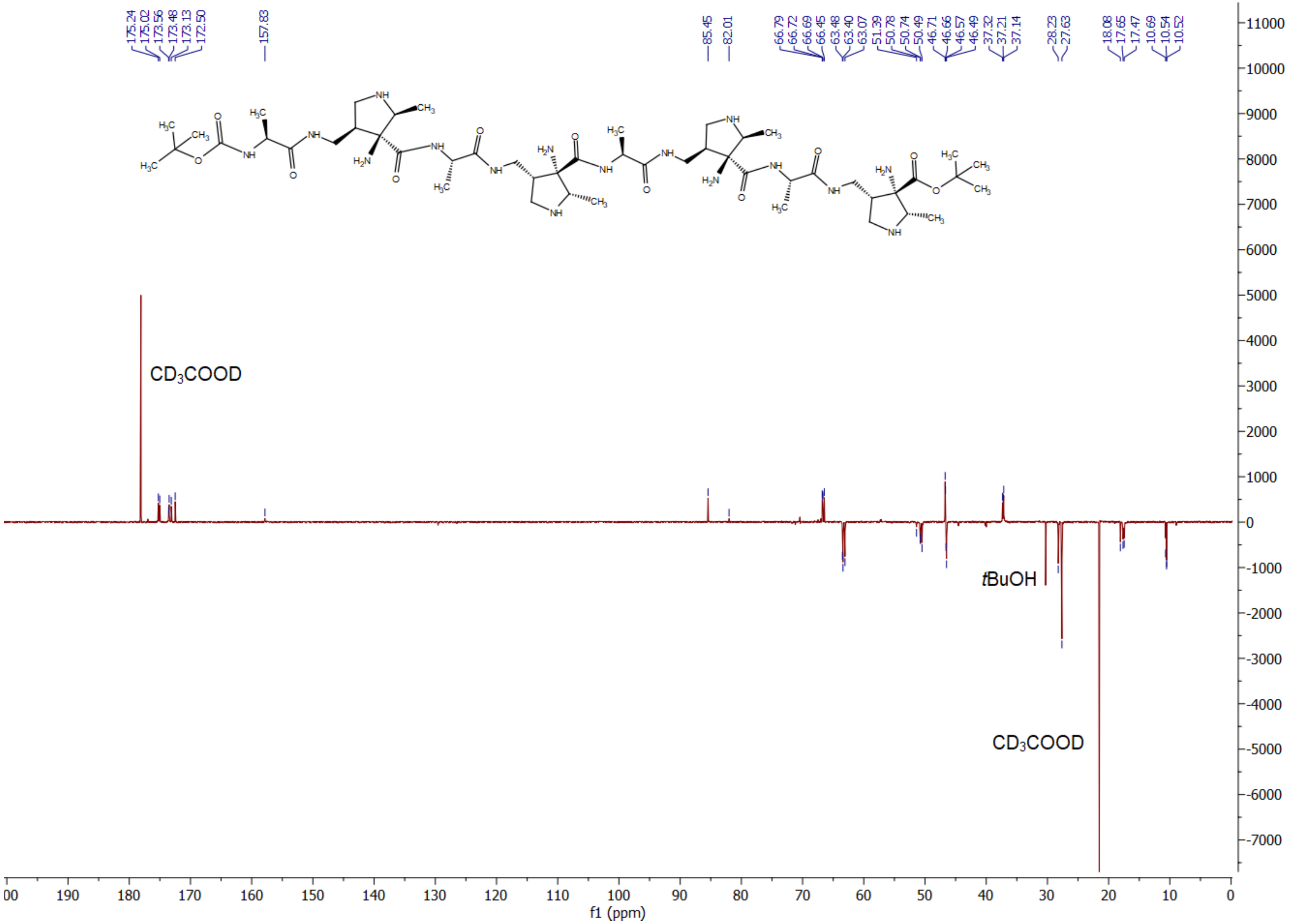

^{13}C-NMR spectrum of α/γ-peptide Boc(Ala-(*S*,*S*,*S*)AAMP)$_4$O*t*Bu **Boc-8AS^{H}A** measured in H_2O:D_2O 9:1, acidified with CD_3COOD to pH of 4 at 151 MHz.

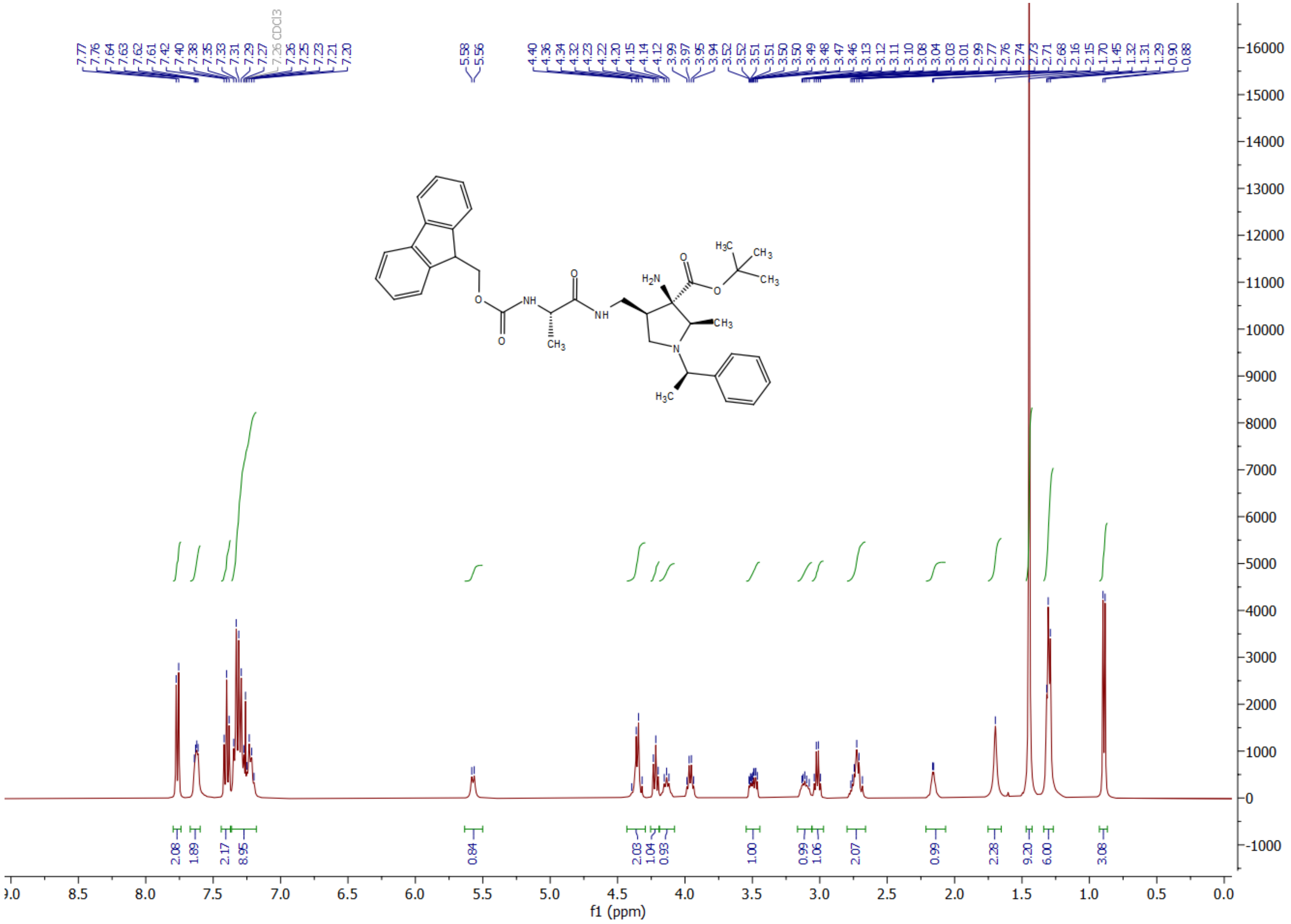

^{1}H-NMR spectrum of α/γ-peptide Fmoc(Ala-(*R*,*R*,*R*,*R*)[P]AAMP)O*t*Bu **Fmoc-2AR[P]A** measured in $CDCl_3$ at 401 MHz.

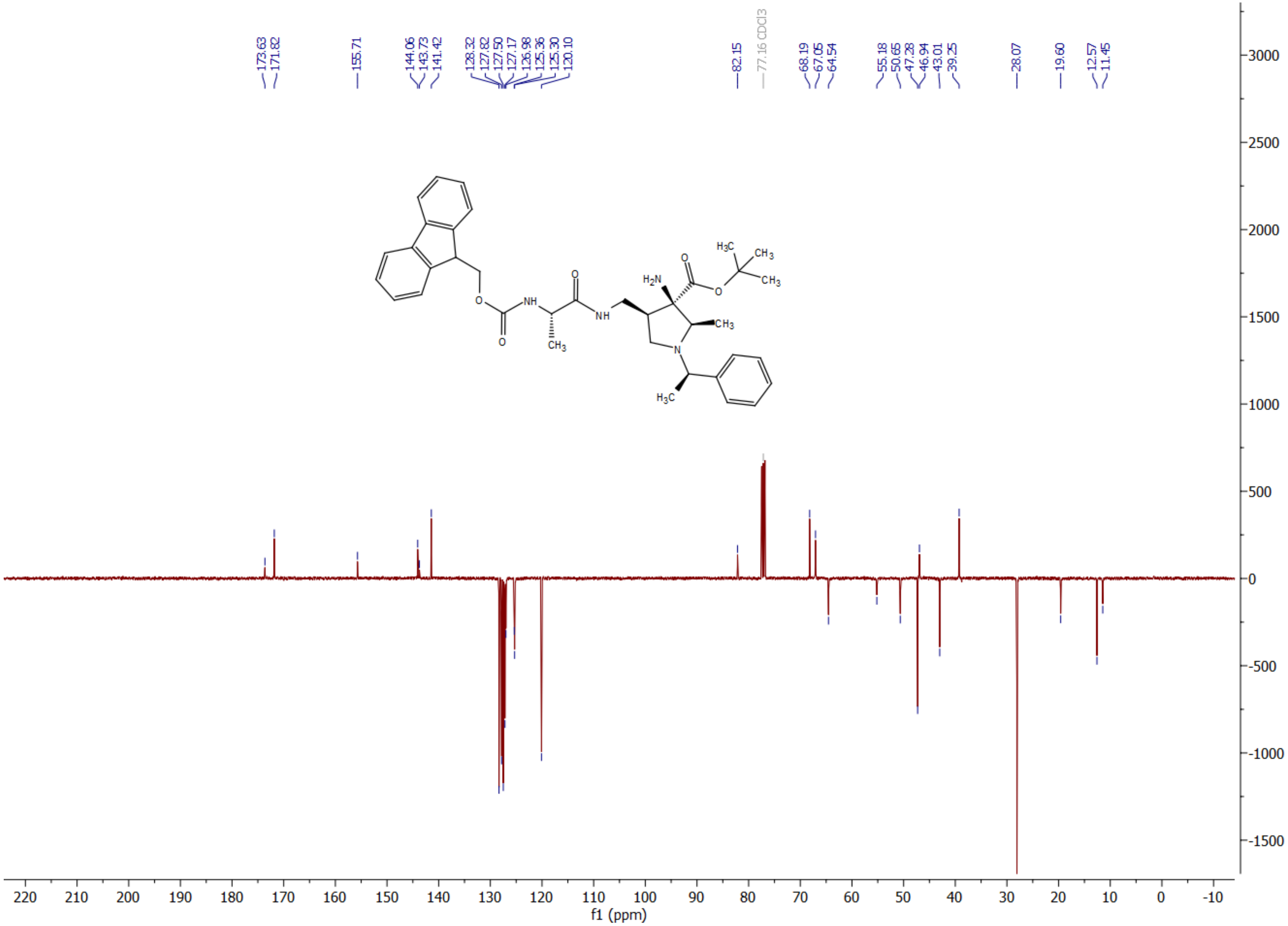

^{13}C-NMR spectrum of α/γ-peptide Fmoc(Ala-(*R*,*R*,*R*,*R*)^{P}AAMP)O*t*Bu **Fmoc-2AR^{P}A** measured in $CDCl_3$ at 101 MHz.

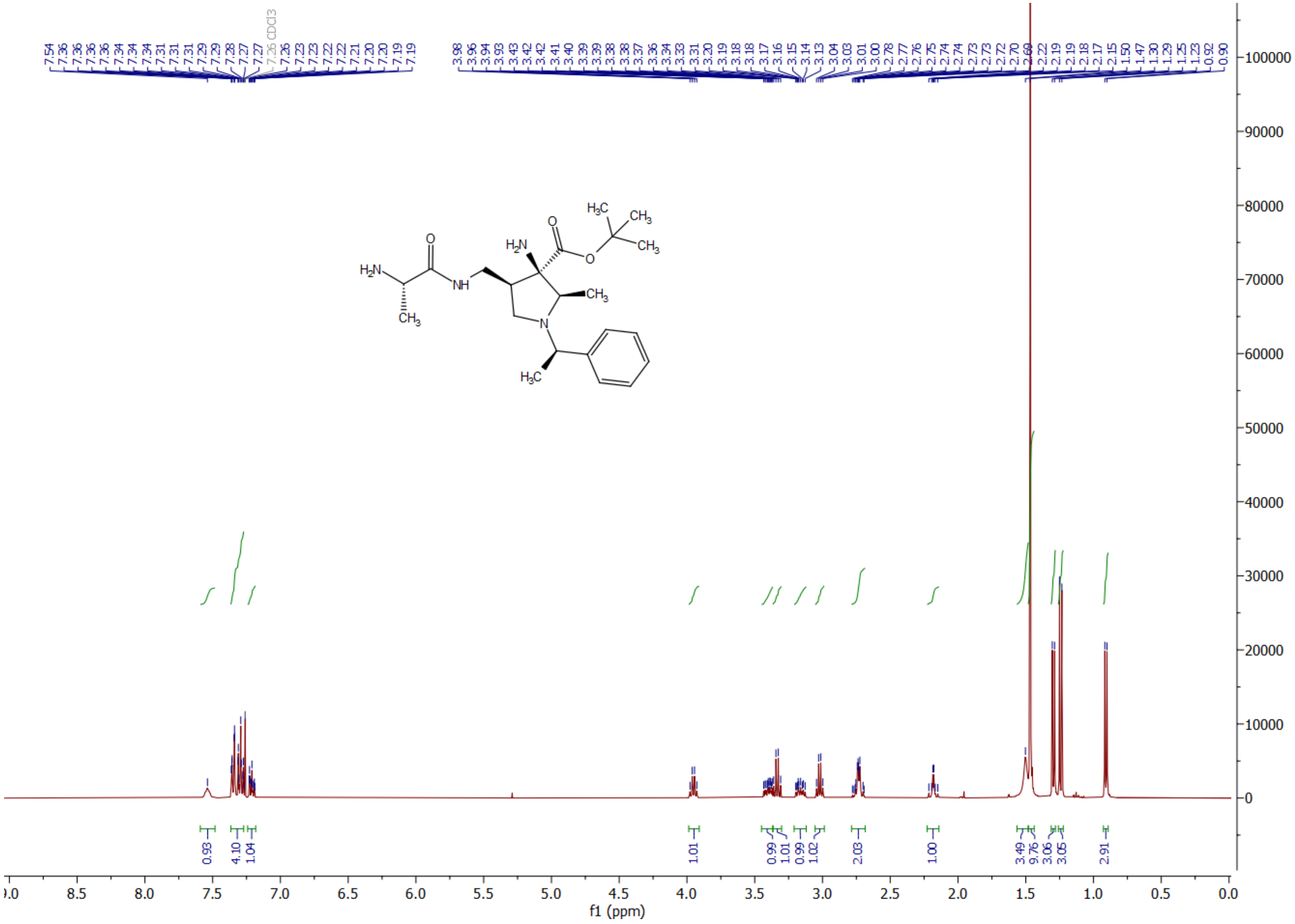

^{1}H-NMR spectrum of α/γ-peptide NH_2(Ala-(*R*,*R*,*R*,*R*)[P]AAMP)O*t*Bu **NH_2-2AR[P]A** measured in $CDCl_3$ at 400 MHz.

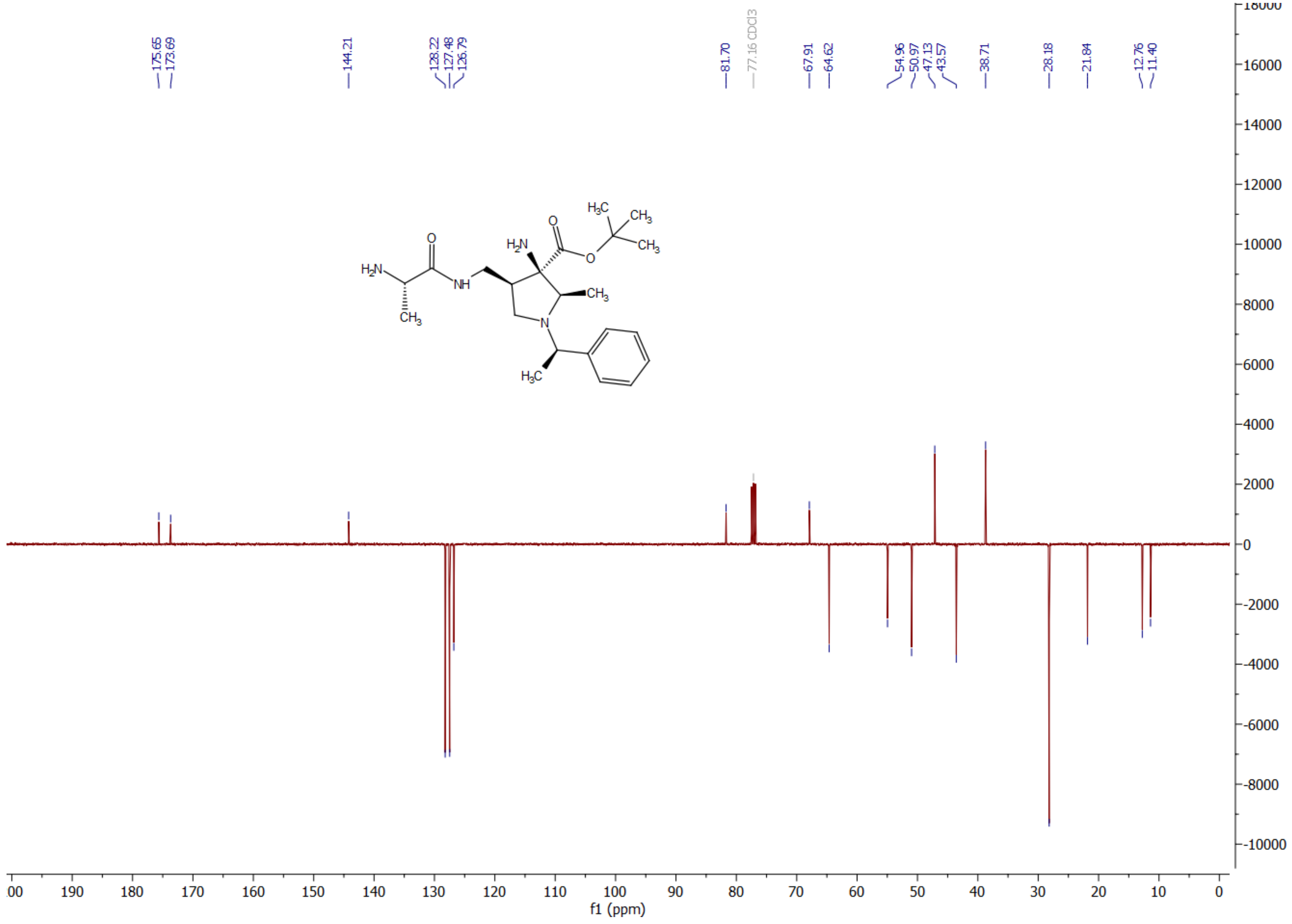

^{13}C-NMR spectrum of α/γ-peptide NH$_2$(Ala-(*R*,*R*,*R*,*R*)^{P}AAMP)O*t*Bu **NH$_2$-2AR^{P}A** measured in CDCl$_3$ at 101 MHz.

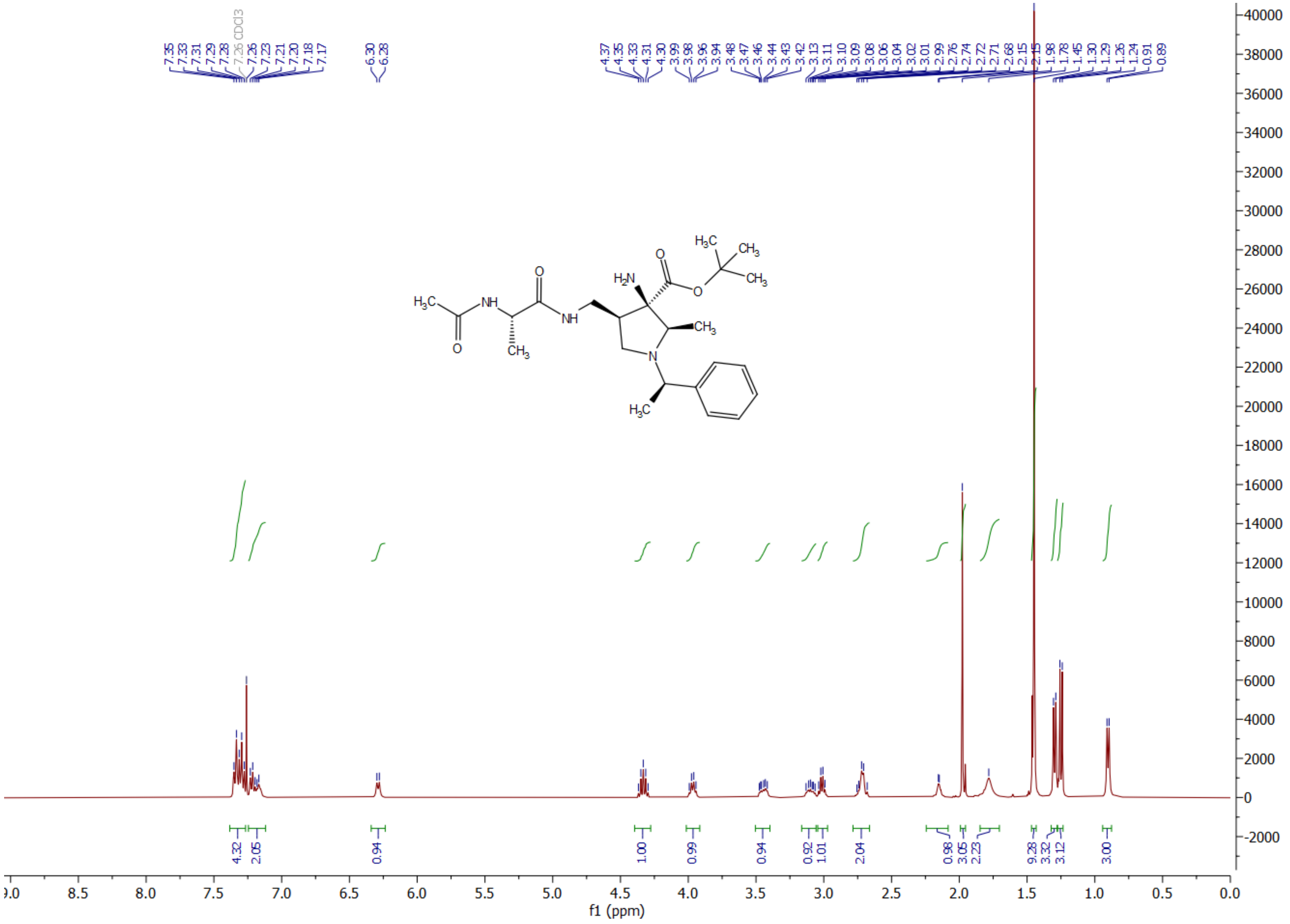

^{1}H-NMR spectrum of α/γ-Peptide Ac(Ala-(*R*,*R*,*R*,*R*)[P]AAMP)O*t*Bu) **Ac-2AR[P]A** measured in $CDCl_3$ at 401 MHz.

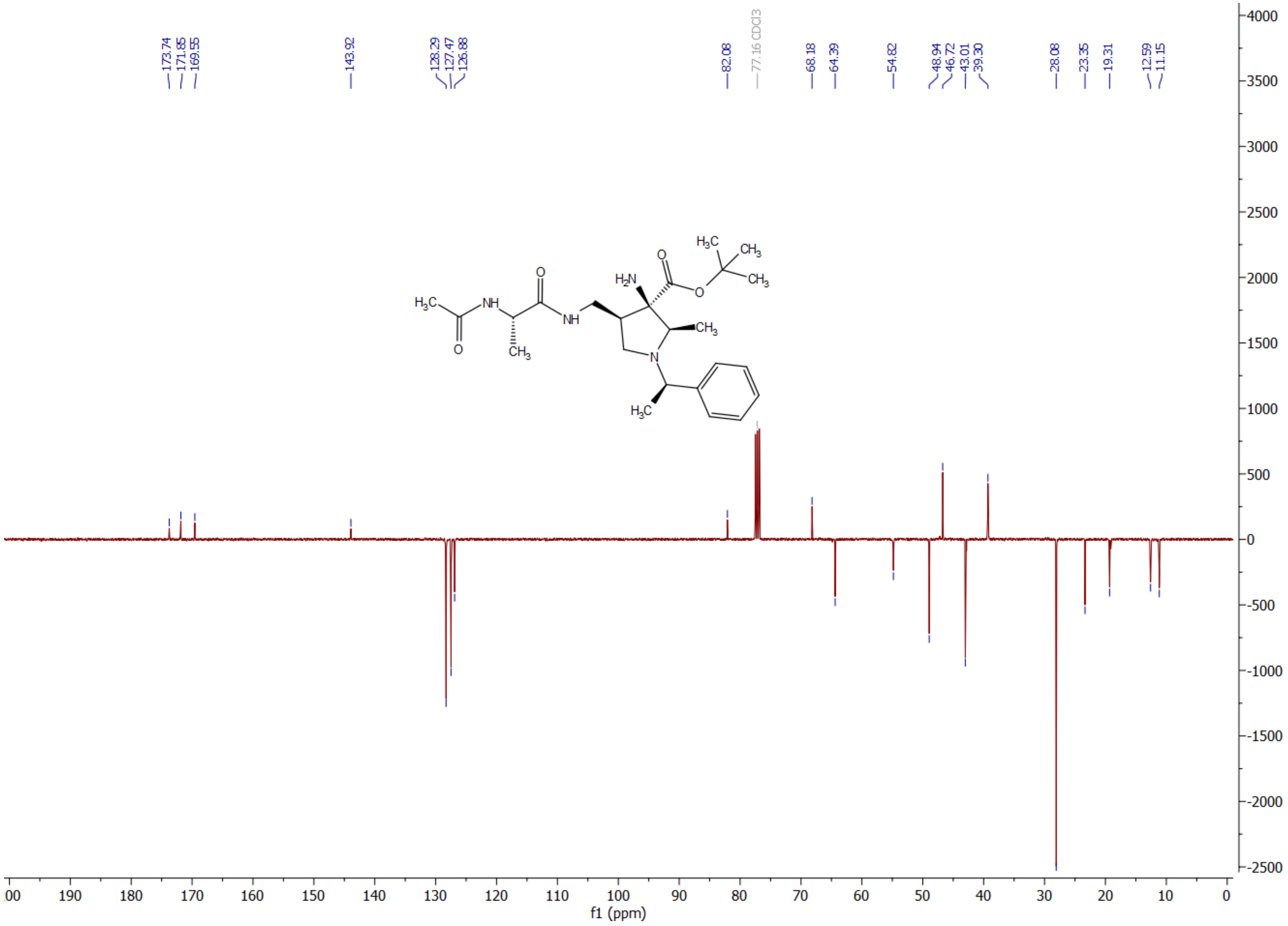

^{13}C-NMR spectrum of α/γ-peptide Ac(Ala-(*R*,*R*,*R*,*R*)^{P}AAMP)O*t*Bu **Ac-2AR^{P}A** measured in $CDCl_3$ at 101 MHz.

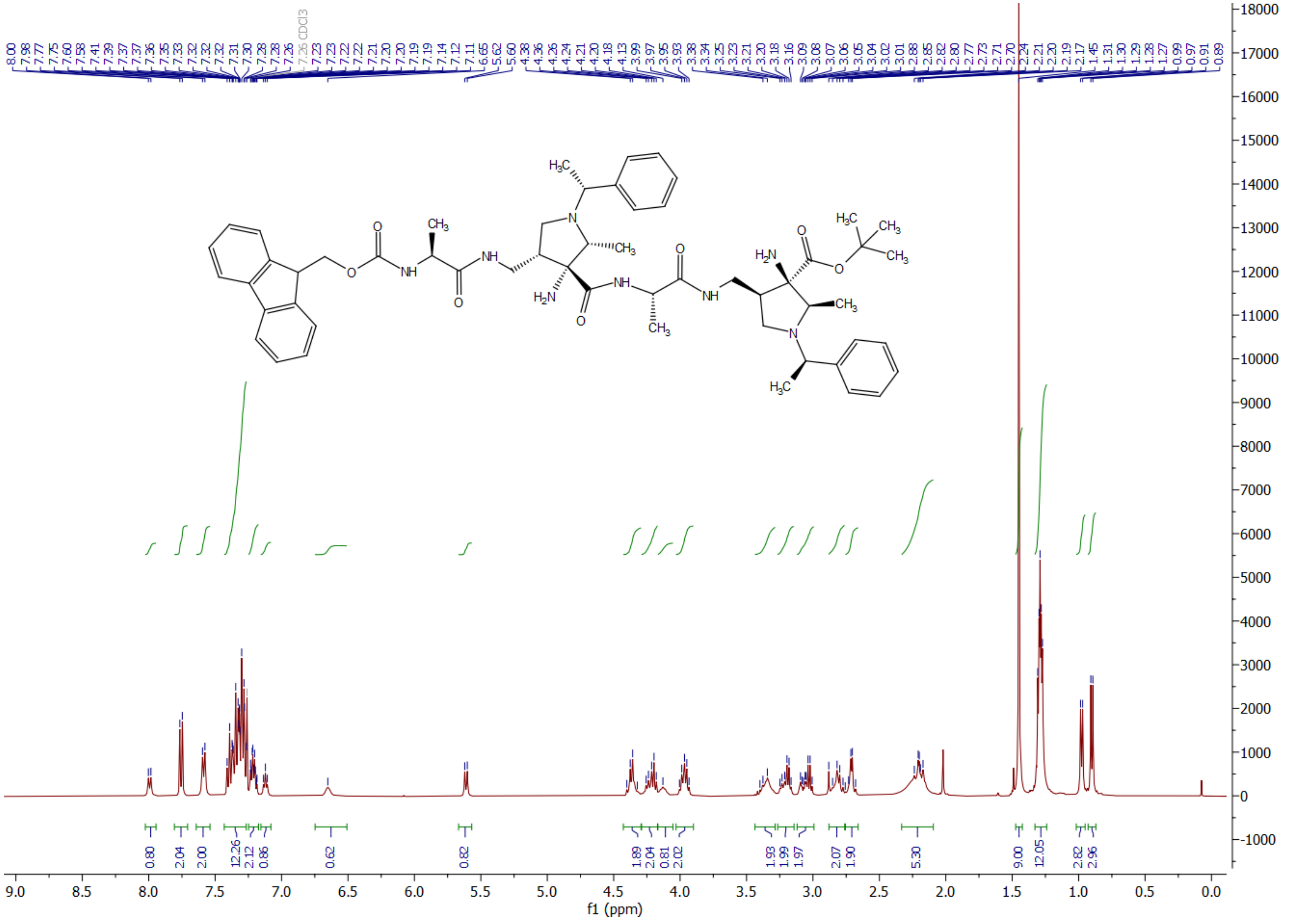

^{1}H-NMR spectrum of α/γ-Peptide Fmoc(Ala-(*R*,*R*,*R*,*R*)[P]AAMP)$_2$O*t*Bu) **Fmoc-4AR[P]A** measured in $CDCl_3$ at 401 MHz.

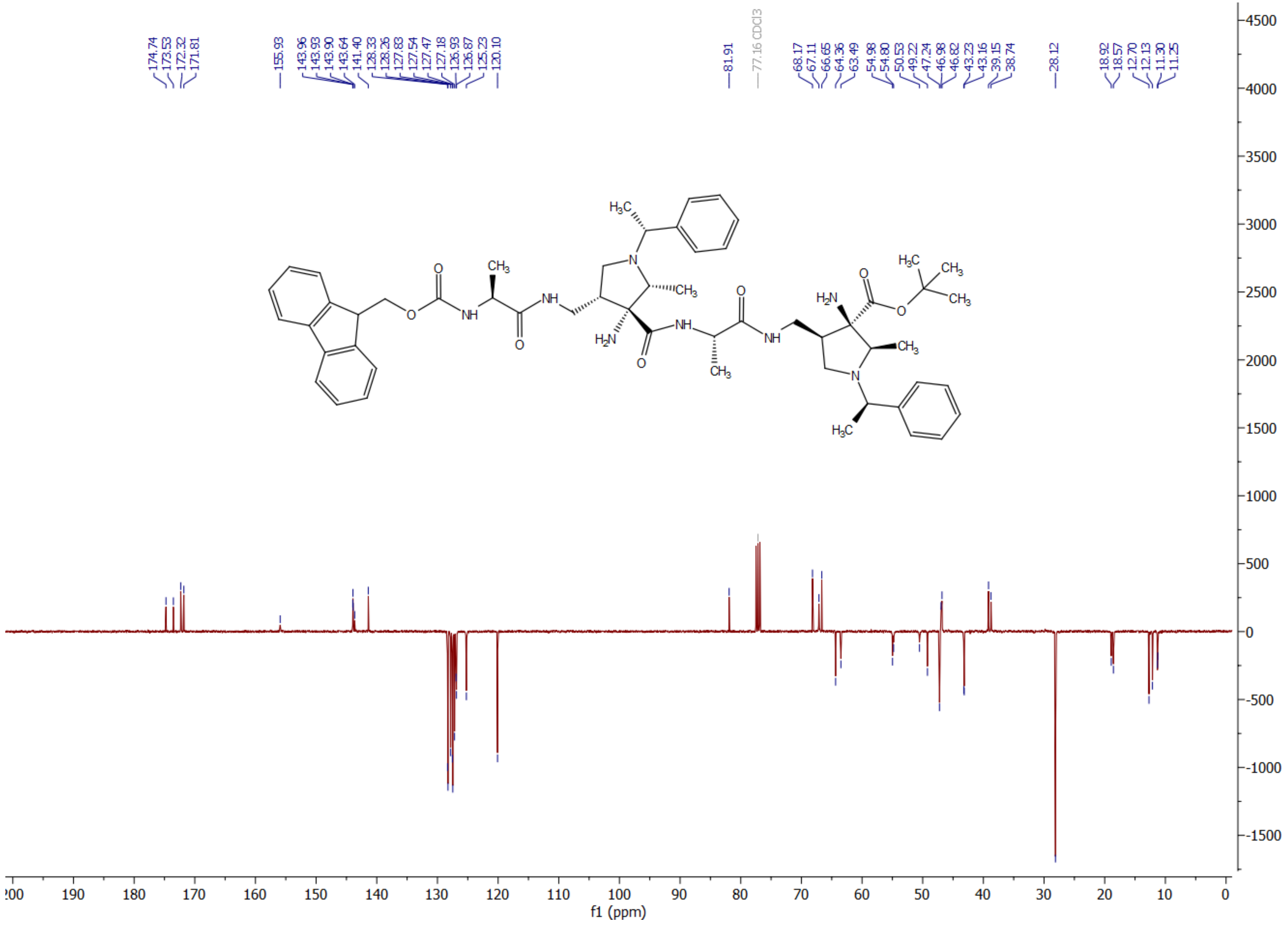

^{13}C-NMR spectrum of α/γ-peptide Fmoc(Ala-(*R*,*R*,*R*,*R*)^{P}AAMP)$_2$O*t*Bu **Fmoc-4AR^{P}A** measured in $CDCl_3$ at 101 MHz.

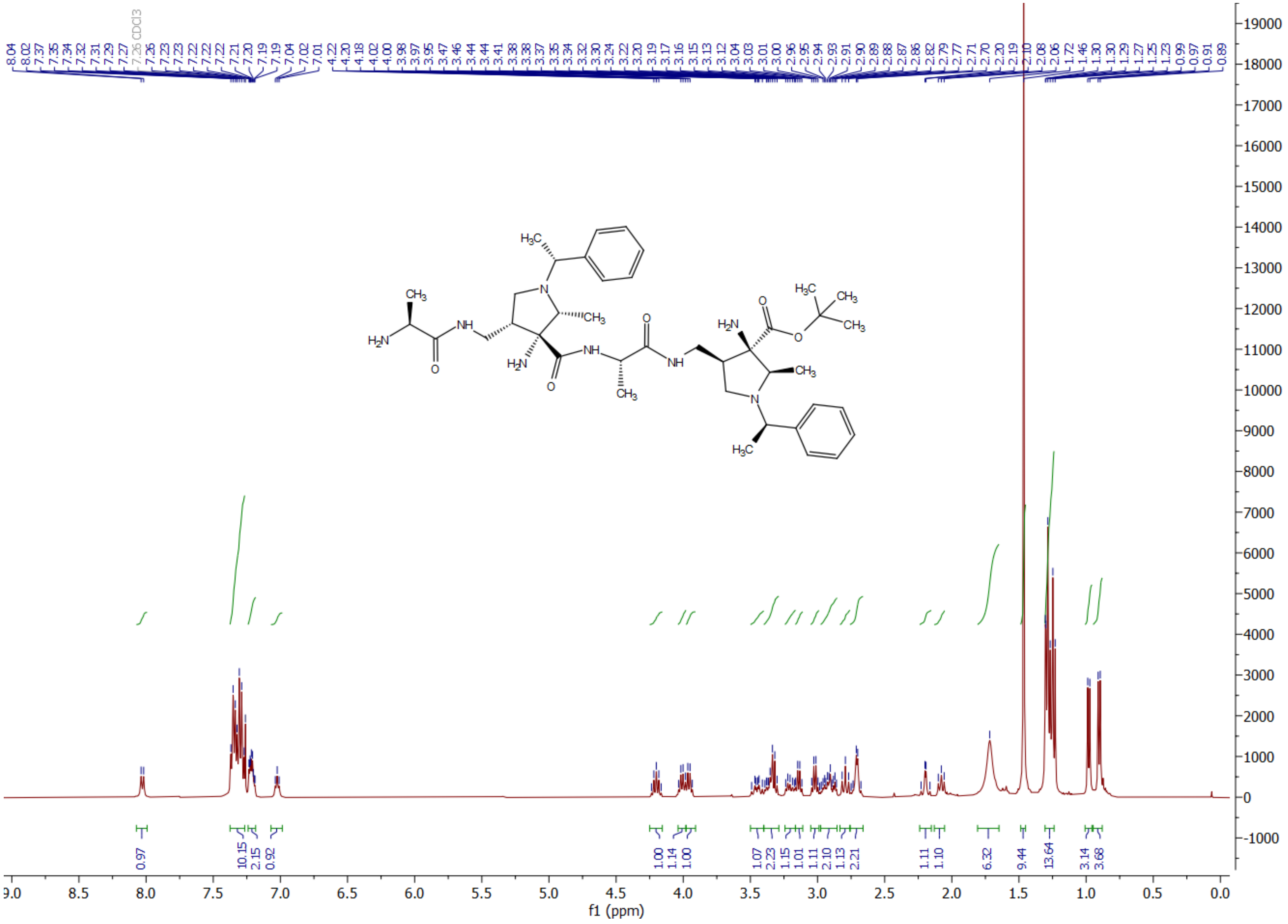

^{1}H-NMR spectrum of α/γ-Peptide NH_2(Ala-(*R*,*R*,*R*,*R*)[P]AAMP)$_2$O*t*Bu) **NH_2-4AR[P]A** measured in $CDCl_3$ at 401 MHz.

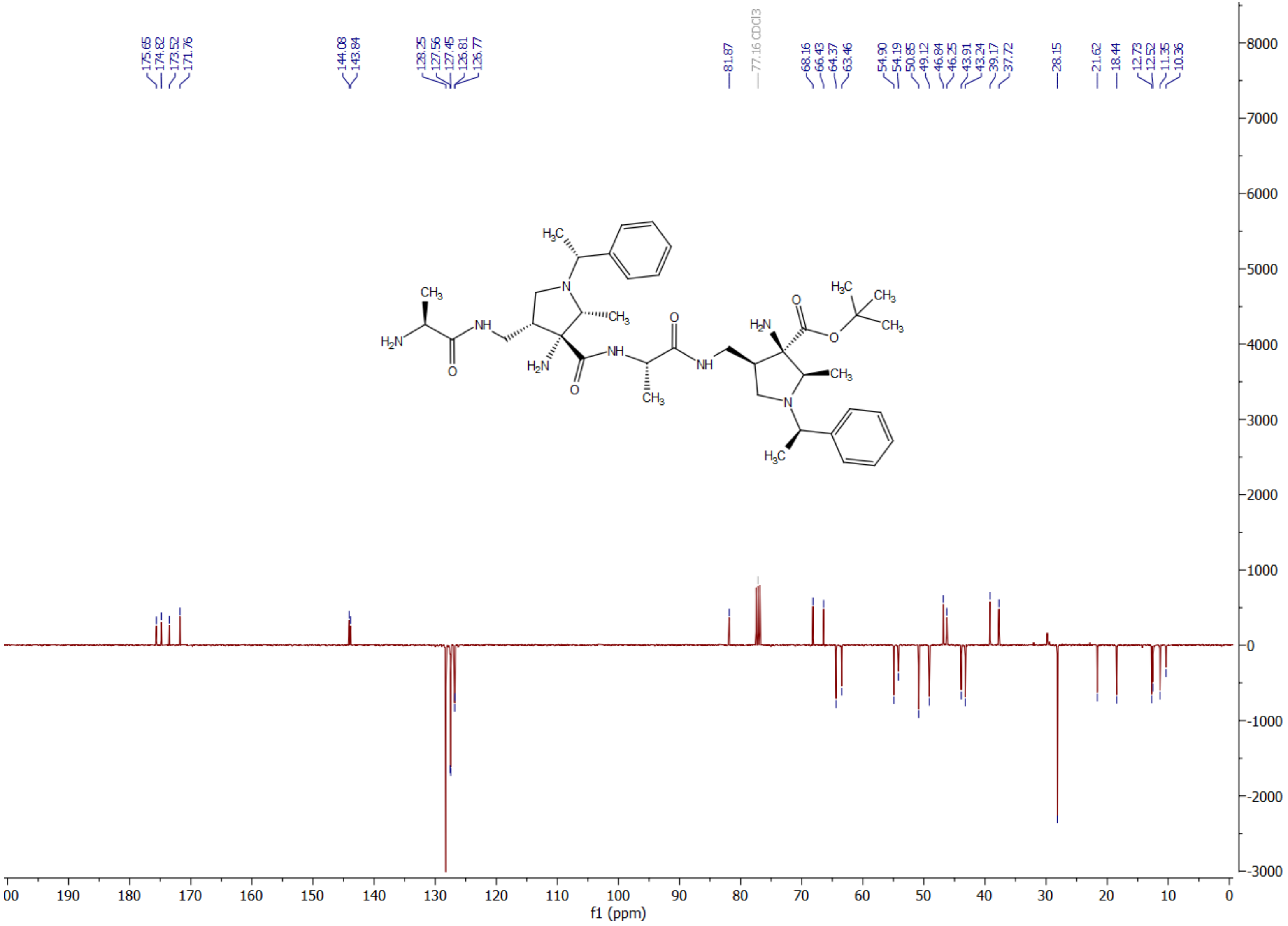

^{13}C-NMR spectrum of α/γ-Peptide NH_2(Ala-(*R*,*R*,*R*,*R*)[P]AAMP)$_2$O*t*Bu) **NH_2-4AR[P]A** measured in $CDCl_3$ at 101 MHz.

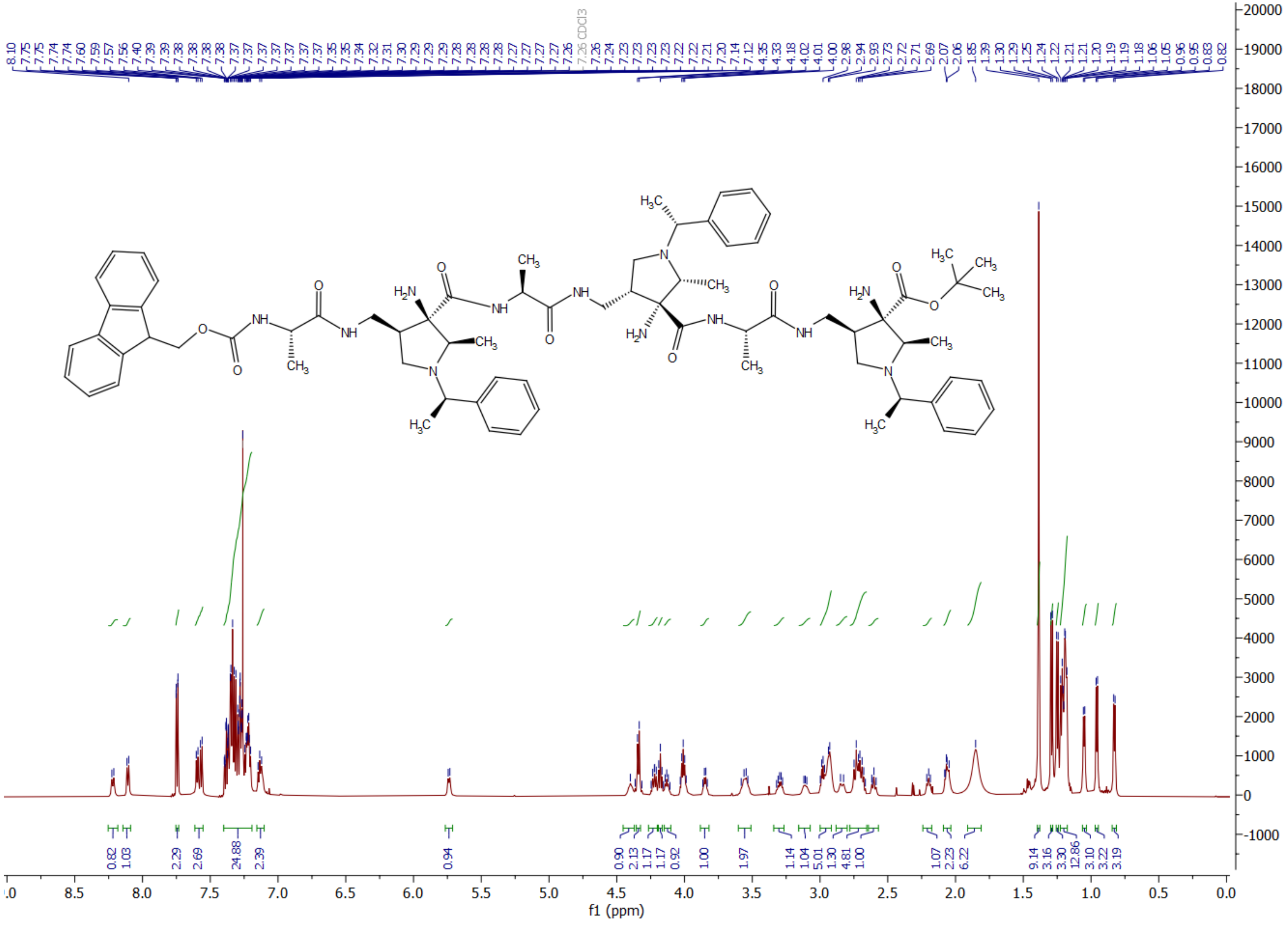

^{1}H-NMR spectrum of α/γ-Peptide Fmoc(Ala-(*R*,*R*,*R*,*R*)^{P}AAMP)$_3$O*t*Bu) **Fmoc-6AR^{P}A** measured in $CDCl_3$ at 600 MHz.

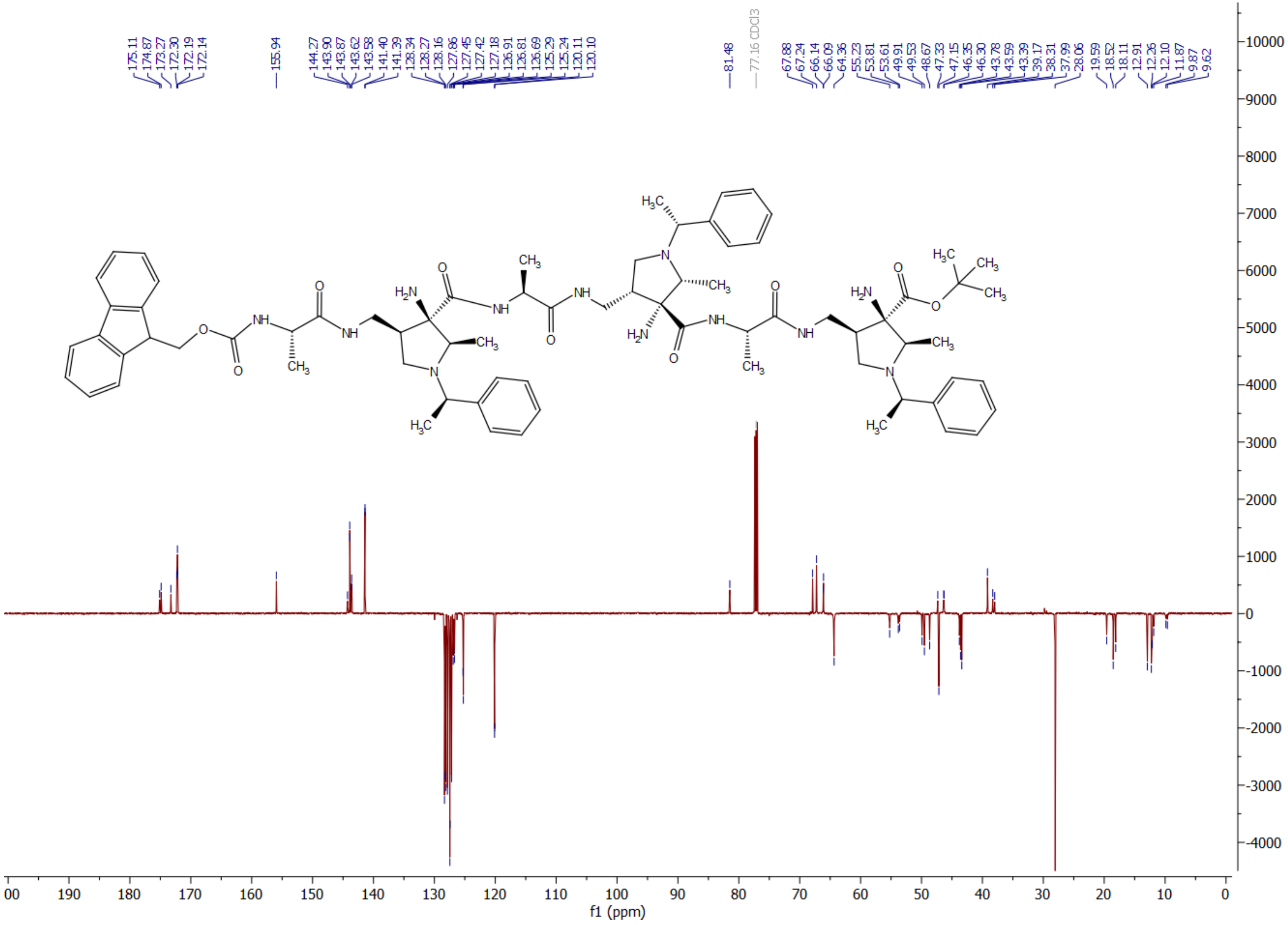

^{13}C-NMR spectrum of α/γ-Peptide Fmoc(Ala-(*R*,*R*,*R*,*R*)^{P}AAMP)$_3$O*t*Bu) **Fmoc-6AR^{P}A** measured in $CDCl_3$ at 151 MHz.

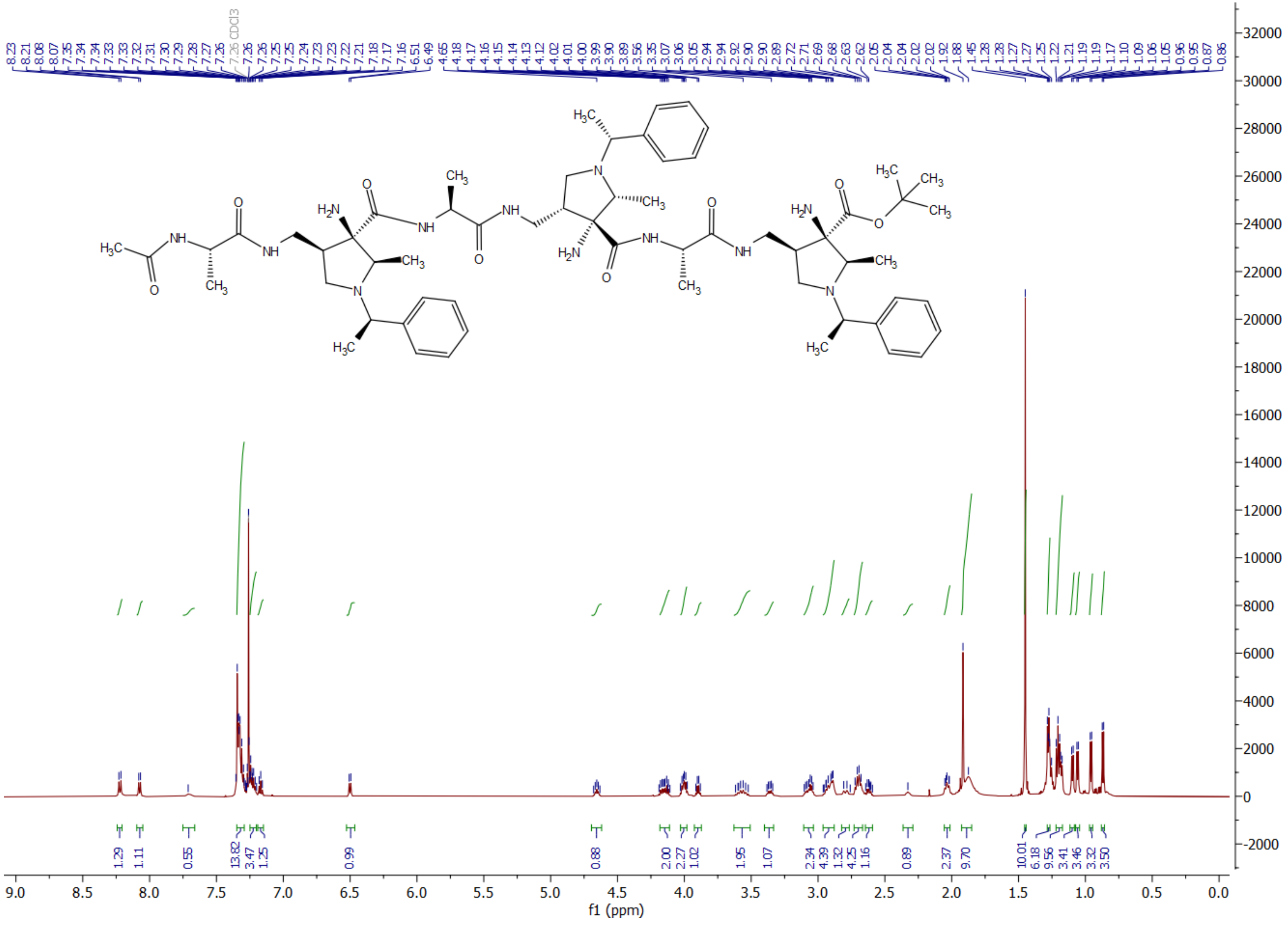

^{1}H-NMR spectrum of α/γ-Peptide Ac(Ala-(*R*,*R*,*R*,*R*)[P]AAMP)$_3$O*t*Bu) **Ac-6AR[P]A** measured in $CDCl_3$ at 600 MHz.

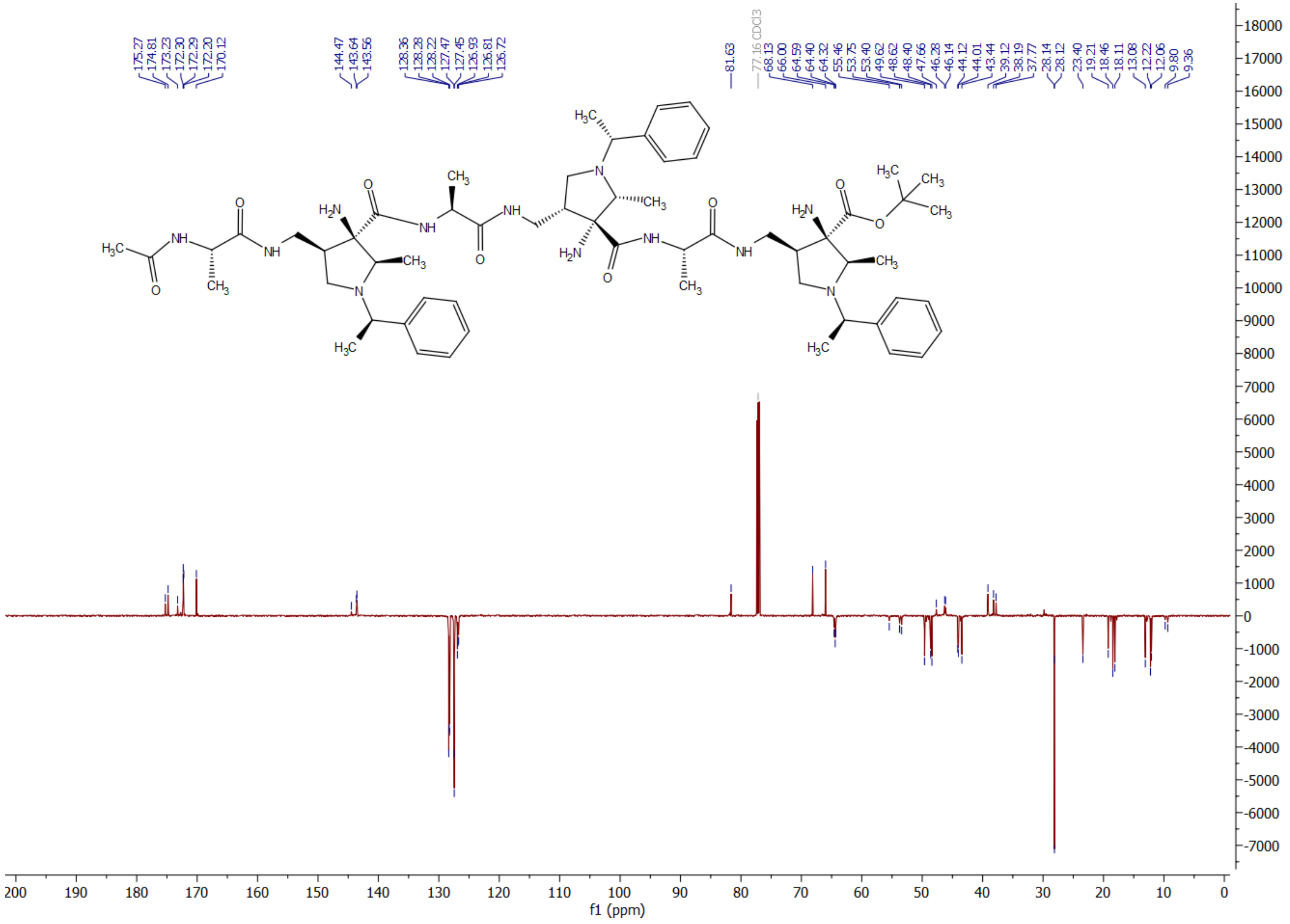

^{13}C-NMR spectrum of α/γ-Peptide Ac(Ala-(*R*,*R*,*R*,*R*)^{P}AAMP)$_3$O*t*Bu) **Ac-6AR^{P}A** measured in $CDCl_3$ at 151 MHz.

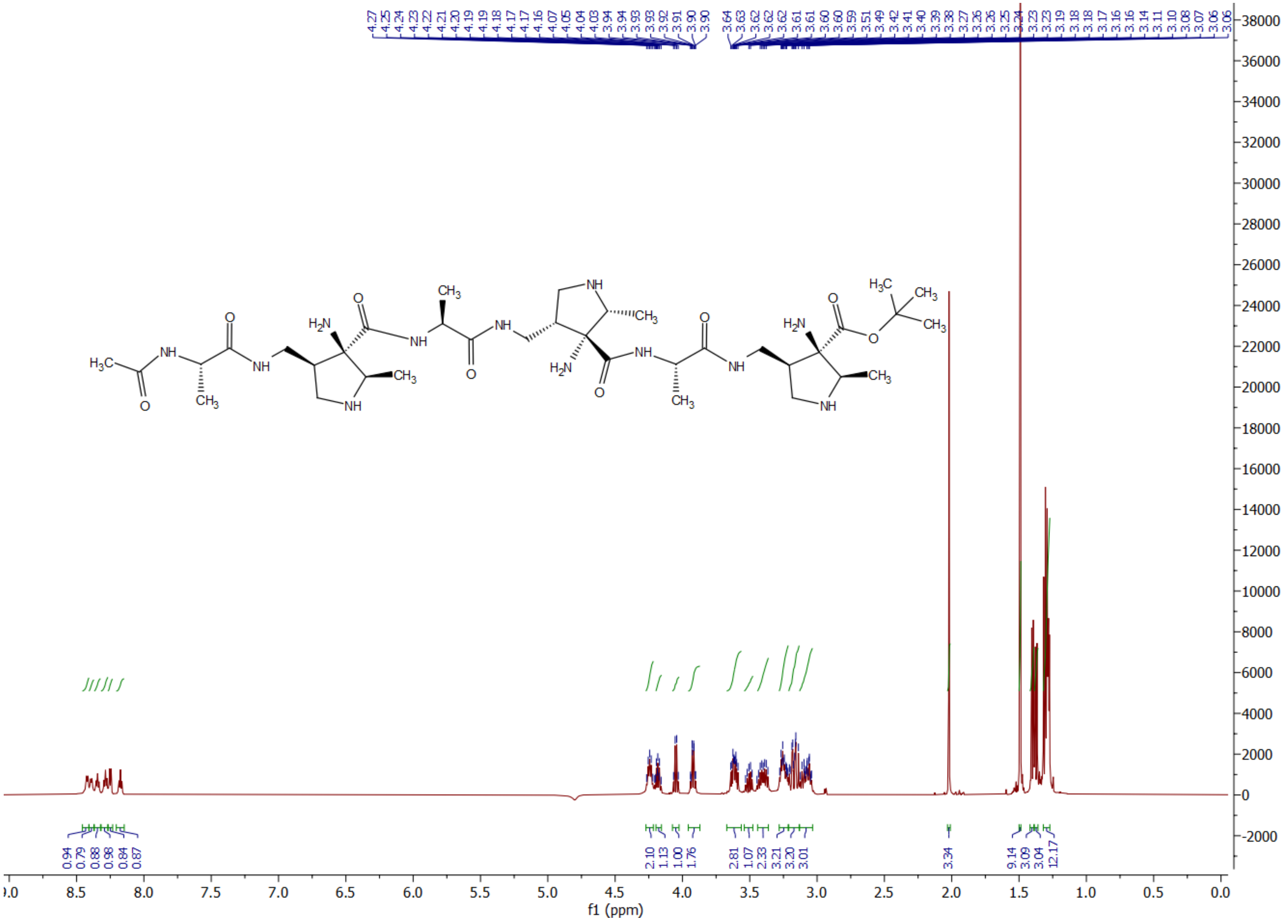

^{1}H-NMR spectrum of α/γ-peptide Ac(Ala-(*R*,*R*,*R*)AAMP)$_3$O*t*Bu **Ac-6AR^{H}A** measured in H_2O:D_2O 9:1, acidified with CD_3COOD to pH of 4 at 600 MHz.

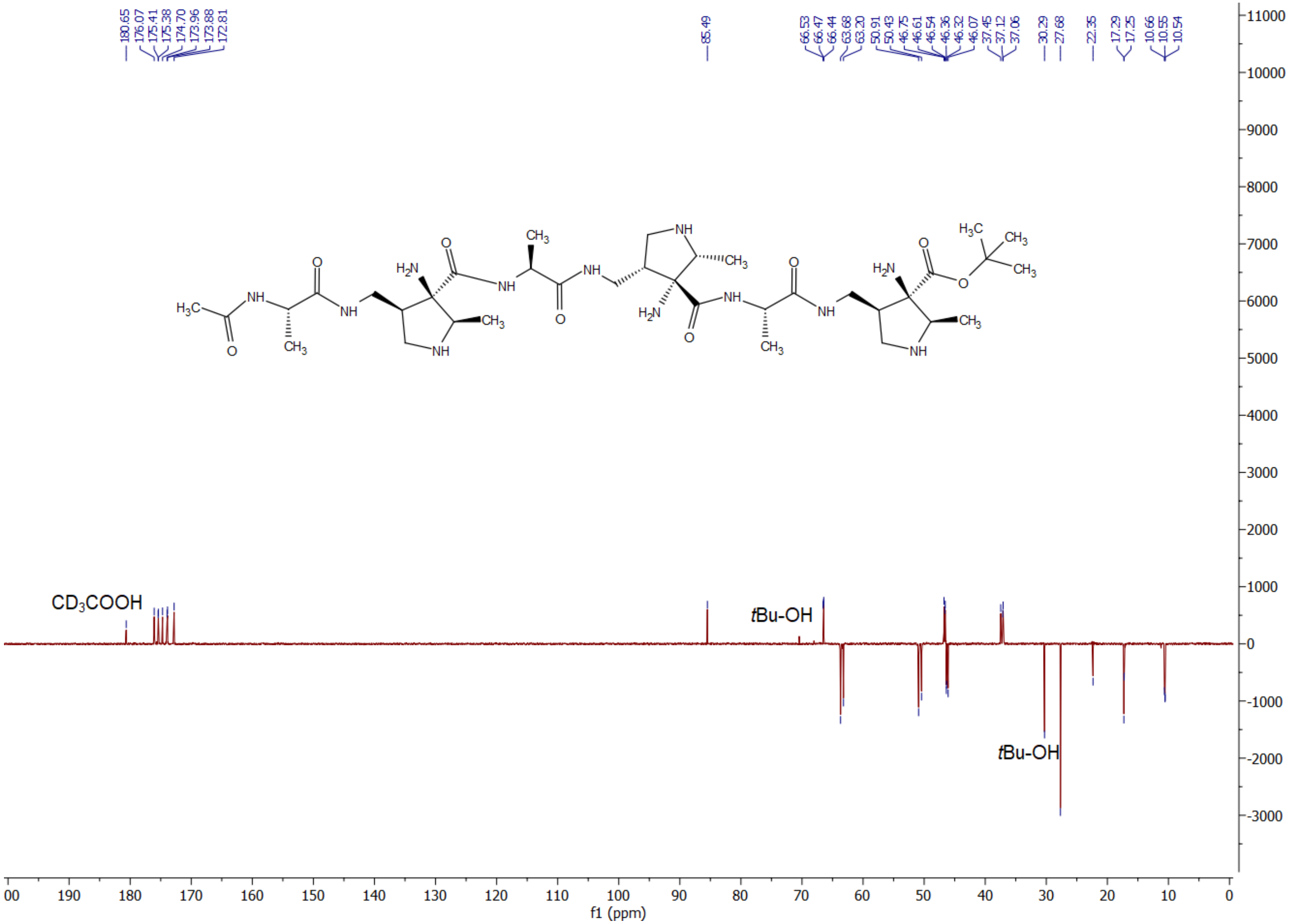

^{13}C-NMR spectrum of α/γ-peptide Ac(Ala-(*R*,*R*,*R*)AAMP)$_3$O*t*Bu **Ac-6AR^{H}A** measured in H_2O:D_2O 9:1, acidified with CD_3COOD to pH of 4 at 151 MHz.
